# China, the Democratic Republic of the Congo, and artisanal cobalt mining from 2000 through 2020

**DOI:** 10.1073/pnas.2212037120

**Published:** 2023-06-20

**Authors:** Andrew L. Gulley

**Affiliations:** ^a^U.S. Geological Survey, National Minerals Information Center, Minerals Intelligence Research Section, Reston, VA 20191

**Keywords:** artisanal cobalt mining, the Democratic Republic of the Congo, China, child labor, responsible sourcing

## Abstract

From 2000 through 2020, demand for cobalt to manufacture batteries grew 26-fold. Eighty-two percent of this growth occurred in China and China’s cobalt refinery production increased 78-fold. Diminished industrial cobalt mine production in the early-to-mid 2000s led many Chinese companies to purchase ores from artisanal cobalt miners in the Democratic Republic of the Congo (DRC), many of whom have been found to be children. Despite extensive research on artisanal cobalt mining, fundamental questions about its production remain unanswered. This gap is addressed here by estimating artisanal cobalt production, processing, and trade. The results show that, while total DRC cobalt mine production grew from 11,000 metric tons (t) in 2000 to 98,000 t in 2020, artisanal production only grew from 1,000 to 2,000 t in 2000 to 9,000 to 11,000 t in 2020 (with a peak of 17,000 to 21,000 t in 2018). Artisanal production’s share of world and DRC cobalt mine production peaked around 2008 at 18 to 23% and 40 to 53%, respectively, before trending down to 6 to 8% and 9 to 11% in 2020, respectively. Artisanal production was chiefly exported to China or processed within the DRC by Chinese firms. An average of 72 to 79% of artisanal production was processed at facilities within the DRC from 2016 through 2020. As such, these facilities may be potential monitoring points for artisanal production and its downstream consumers. This finding may help to support responsible sourcing initiatives and better address abuses related to artisanal cobalt mining by focusing local efforts at the artisanal processing facilities through which most artisanal cobalt production flows.

## Significance

The linkage between abuse to artisanal cobalt miners—including children—in the Democratic Republic of the Congo (DRC) and use of cobalt in advanced batteries has prompted global supply chain reviews, responsible sourcing initiatives, and research to remove cobalt from battery cathodes. However, no study has systematically evaluated how large of a role artisanal mining plays in world cobalt mine production. Doing so reveals artisanal supply trends and potential monitoring points for artisanal production to help provide more transparent cobalt supply chains. This study finds that artisanal cobalt mining’s share of world cobalt mining has generally decreased since 2008 and that most artisanal production was either processed in the DRC by Chinese firms or exported to China.

Lithium-ion batteries have become, and will likely continue to be, the dominant energy storage platform for emerging, sustainable, and strategic technologies including smartphones, electric vehicles, and battlefield communications (1). This—as well as China’s overseas foreign investments in mining assets (2)—has prompted concerns about competition for, and the supply risk of, battery materials and other critical mineral commodities (3–5). Around the turn of the century, the Chinese government began to implement plans to create domestically self-sufficient supply chains for battery-consuming industries of the early 2000s, such as mobile phones, laptops, and electric vehicles (6). Given that China has limited domestic cobalt mine production, state-owned Chinese firms began expanding China’s domestic cobalt refining capacity. Specifically, most of this capacity was to feed the production of battery-grade cobalt oxide (tricobalt tetroxide) for lithium cobalt oxide battery cathode materials (7), initially for laptops and mobile phones. Later, the expansion of battery-grade cobalt chemical production also proved crucial to China’s long-term strategic plans of leapfrogging Western manufacturers of internal combustion engines to dominate the future manufacture of electric vehicles (6).

During a 42-fold increase in China’s cobalt refinery production from 1999 through 2005, the country that historically mined most of the world’s cobalt—the Democratic Republic of the Congo (DRC)—had just begun to rise from a socioeconomic collapse and two African wars over its territory and resources (8). Before these crises, DRC copper–cobalt mines were industrial (also known as large scale) mines organized by firms that provided large capital investments to build mechanized mining capacity on large mining claims (8). In the early 2000s, almost all industrial cobalt mines still in operation in the DRC and elsewhere were already owned by or under long-term supply agreements with established Western cobalt-refining firms (8). As a result, cobalt trading, concentrating, and processing firms (most of which were Chinese owned) came to the DRC to purchase cobalt ores/concentrates that were produced by individuals or groups using simple tools in unmechanized operations that were remote and outside of the formal economy (8–10). This type of cobalt ore production is known as artisanal cobalt mining and has become popularized by images of men hauling bags of high-grade cobalt ore out of hand-dug tunnels and pits, women washing that ore, and children picking through or transporting the washed ore (10).

Since 2000, research on artisanal cobalt mining has revealed exploitative practices, human rights abuses, and child labor (10–12). This has led many to call for the removal of cobalt from lithium-ion batteries, even though to do so would reduce many batteries’ performance and fire safety—which may delay or stem the growth of technologies key for a sustainable energy transition such as electric vehicles (13, 14). Largely due to a 2016 report on the subject (10), growing public and corporate awareness of these issues prompted a wide array of efforts to stem related abuses. Multigovernmental organizations such as the Organization for Economic Co-operation and Development (OECD) and the Chinese Chamber of Commerce for Metals, Minerals, and Chemicals have developed responsible sourcing guidelines (15, 16). Nongovernmental organizations (NGOs) have studied artisanal cobalt mining regions (9, 10, 12). Governmental organizations have developed regulations regarding artisanal cobalt mining (17). Research organizations have sought to develop methods for reducing (or even removing) cobalt in advanced batteries (14, 18, 19). And original equipment manufacturers (OEMs) have demanded traceable cobalt supply chains from their suppliers (15).

**Fig. 1.**
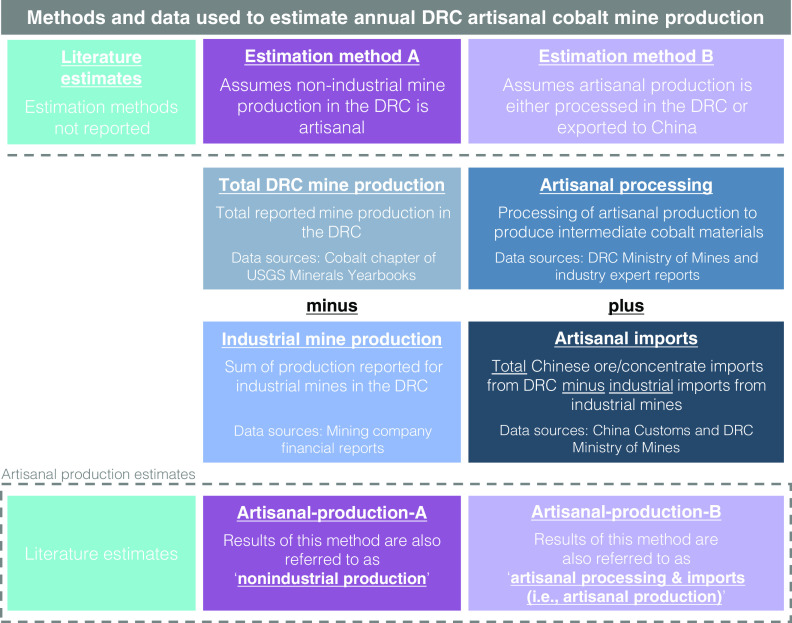
Methods and data used to estimate artisanal cobalt mine production in the Democratic Republic of the Congo (DRC): unknown methods for literature estimates in the left column, artisanal-production-A, also referred to as “nonindustrial production” in the middle column; artisanal-production-B, also referred to as “artisanal processing and imports (i.e., artisanal production)” in the right column.

The purpose of this paper is threefold: to transparently estimate artisanal cobalt production, to evaluate where this production is processed, and to determine where it is exported. To inform the initiatives above and to address these gaps in the literature, 53 artisanal production estimates are collected from the literature (Dataset S1) and two methods are developed to estimate artisanal production for the years from 2000 through 2020 (Fig. 1). The literature references do not report methods employed for their estimates of artisanal cobalt production, which is shown in Fig. 1 by the absence of an estimation methodology in the left column. Rather than relying on an estimation method, many literature references provide rough percentages of total DRC cobalt mine production. The lack of transparent estimation methods for literature estimates motivates the development of the two estimation methods presented below. Any use of trade, firm, or product names is for descriptive purposes only and does not imply endorsement by the U.S. government.

Fig. 1 presents method A and method B in the center and right columns, respectively. In method A, the portion of cobalt mine production in the DRC that cannot be accounted for by industrial mines (Dataset S2) is assumed to approximate artisanal production (Dataset S3). This method is referred to as “artisanal-production-A” or “nonindustrial production.” In method B, artisanal production is assumed to be comprised of one portion that is processed into cobalt-intermediate materials at facilities in the DRC (referred to as “artisanal processing,” see Dataset S4) and another portion that is imported into China (referred to as “artisanal imports,” see Dataset S5 through Dataset S9). This method is presented in the right column of Fig. 1 and is referred to as “artisanal-production-B” or “artisanal processing and imports (i.e., artisanal production).” See *SI Appendix*, *Supporting Discussion 1* for assumptions and variables that may impact the results of the two methods, as well as a comparison in the confidence of their results. See Datasets S10–S12 for numerical results.

The geographic scope of this analysis is limited to the copper belt of the DRC because artisanal cobalt production is limited to that region (9, 10, 12). The temporal scope of this analysis, from 2000 through 2020, covers the modern era of cobalt production in the DRC as it emerged from the collapse of its mining industry in the 1990s, which was the result of underinvestment in nationalized mining assets, as well as economic and civil turmoil (8). The year 2000 was also chosen as the start year because it corresponds to the beginning of both China’s economic expansion and growth of cobalt demand for batteries. By its nature, artisanal cobalt mining production is difficult to estimate because it is often disorganized, secretive, remote, and unsanctioned. The goal of this analysis is therefore not to ascertain the exact quantity of artisanal production each year. Rather, it is to gather all available estimates from the literature into a single analysis; compare the reported estimates to the results of the two estimation methods developed here (i.e., accounting estimates); compare multiple results within a single year; and identify artisanal production trends, destinations, and drivers.

**Fig. 2.**
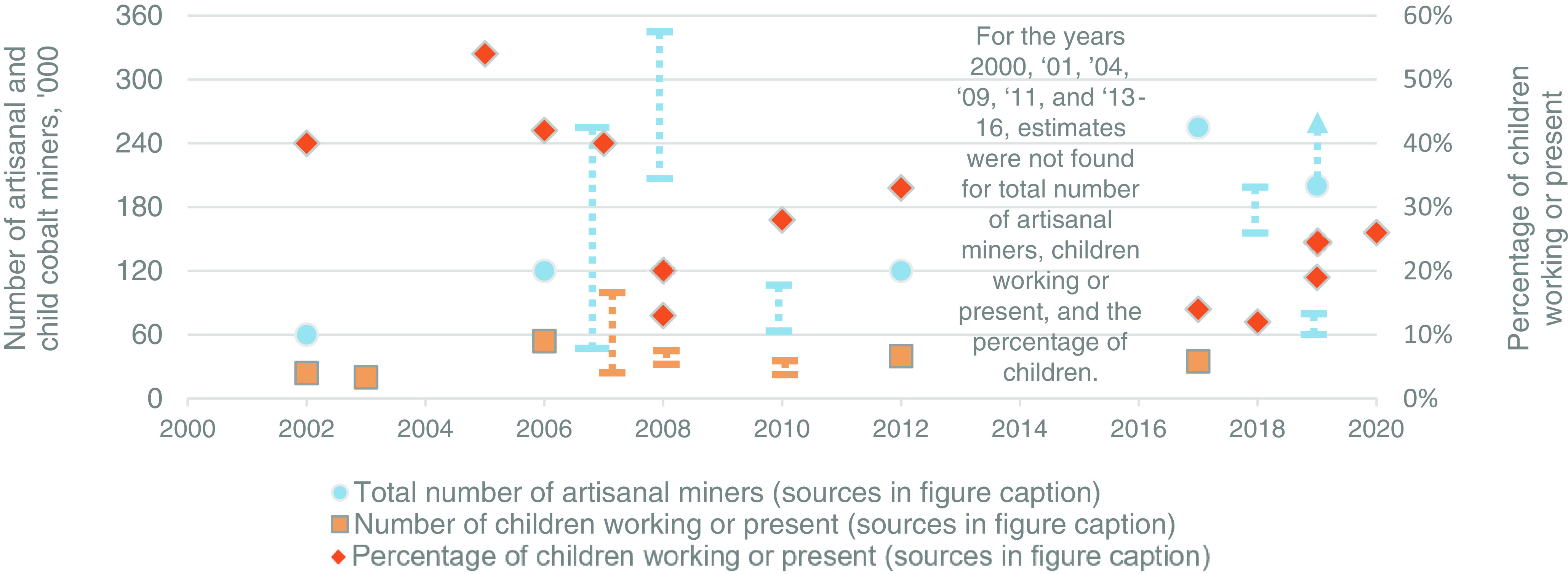
Literature estimates as found in original studies (as opposed to confirmed within this analysis) of the number of total artisanal cobalt miners (aqua dots), number of children working or present at artisanal cobalt mine sites (light orange squares), and these children as a percentage of the total number of artisanal cobalt miners (dark orange diamonds). Dotted bars indicate reported estimate ranges. Upward arrows indicate estimates reported to be “greater than” a certain number. The reference of each estimate can be found in the references based on the following abbreviations: 2002, Global Witness; 2003, Tshilobo; 2005, Groupe One; 2006, Tsurukawa; 2007, World Bank; 2008 (20% children estimate only), PACT; 2008 (all other estimates), Faber; 2010, Tsurukawa; 2012, UNICEF; 2017, Kara; 2018 (12% children estimate), Hanrui Cobalt; 2018 (150 to 200 thousand total miners), BGR-2019; 2019 (19% children estimate), BGR-2019; 2019 (60 to 80 thousand total miners), PACT; 2019 (greater than 200 thousand total miners and 25% children estimates), BGR-2021.

For context and general interest, estimates of the total number of artisanal miners, the number of children working or present at artisanal cobalt sites, and the share of children estimated to be at such sites are gathered from the literature. For the same reasons that it is difficult to estimate artisanal cobalt production, it is difficult to estimate how many people are working at artisanal mine sites. It is especially difficult to estimate the number of children who are digging, tunneling, washing ore, sorting ore, transporting ore, running errands, watching younger siblings, or being watched by parent miners (10, 20–25). The estimates from the literature are presented as found in the original studies in Fig. 2 with abbreviated references in the figure caption. Notably, these estimates have not been confirmed in this analysis. Complications in estimating the number of total and child artisanal cobalt miners include limited site visits, colocated living/mining spaces, and misreporting of combined estimates that include both artisanal cobalt miners in the southern copper–cobalt belt with artisanal tin, tungsten, tantalum (i.e., coltan), and gold miners in northern Katanga (10, 20–25). Unlike these so-called 3TG commodities, cobalt has not been designated as a conflict mineral (26) and recent studies have found no signs of forced labor (20, 23, 24, 27). See *SI Appendix*, *Supporting Discussion 2* for information about the studies that estimate the number of artisanal miners in the DRC and Dataset S13 for numerical estimates.

Artisanal cobalt mine site studies and interviews provide an indication of the types of work children may be involved in at such sites. These estimates are not confirmed here and are presented as found in the original studies. Of children working at cobalt mine sites, site studies have estimated that roughly 50% are fifteen to seventeen years old, roughly 40% are ten to fourteen, and the remainder are younger than ten (20, 22). Similarly, of children working at cobalt mine sites, roughly 20% clean ores that have been extracted, 25% sort cleaned ores, another 25% are working in other activities at the surface, and the remaining 30% are transporting ore or performing other tasks (20, 22). Of children who are excavating ore underground or at the surface, between 60% and 70% are 15 or older (22). Artisanal cobalt mine site studies have estimated that children numbered 24,000 or 40% of total artisanal cobalt miners in 2002 (9), 60,000 or 40% in 2007 (25), and 35,000 or 14% in 2017 (28).

## Results

### Comparison of Results and Literature Estimates.

**Fig. 3.**
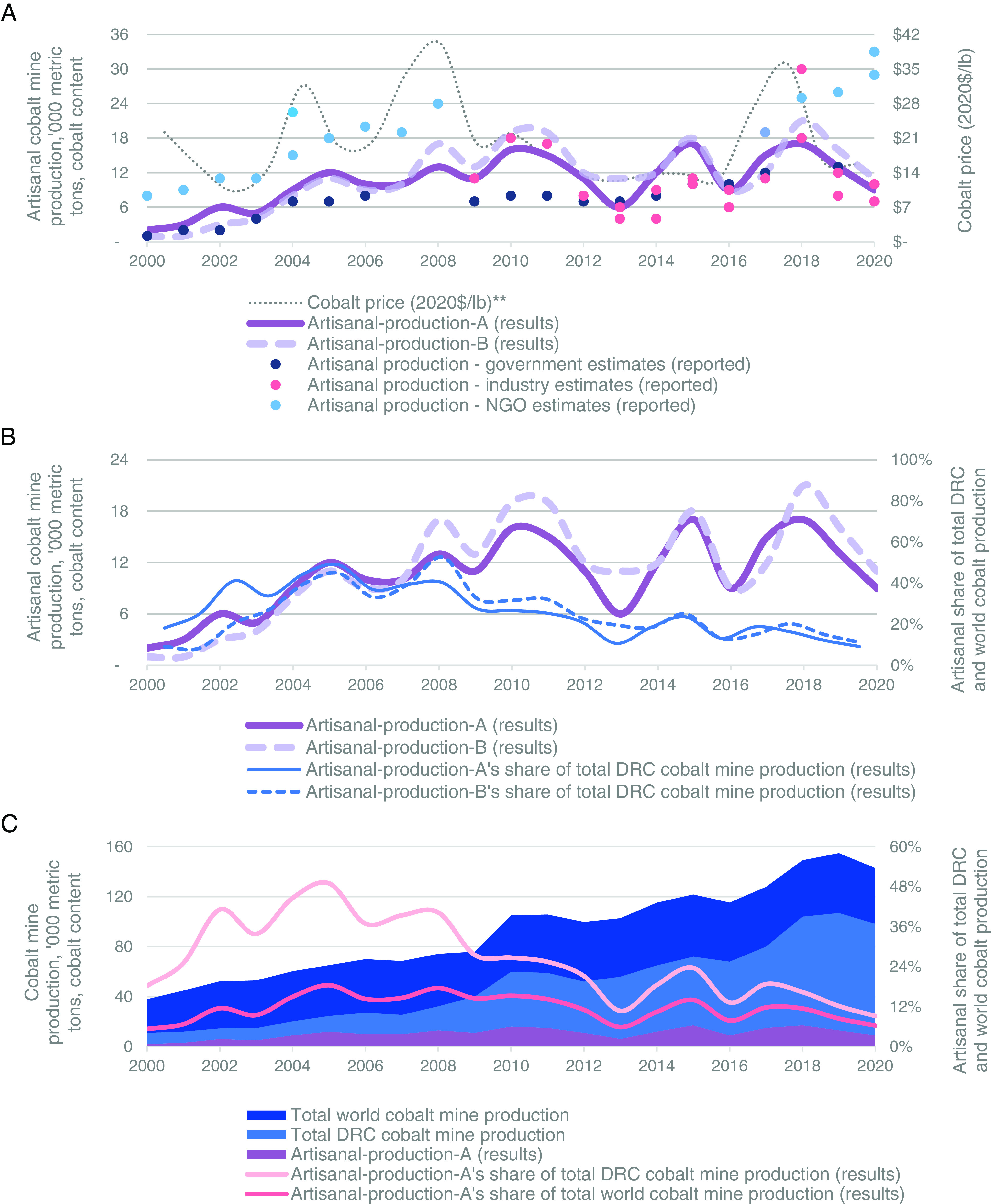
Estimates of artisanal cobalt mine production in the Democratic Republic of the Congo (DRC) and artisanal cobalt mine production’s share of world and DRC cobalt mine production. (*A*) Literature estimates of (dots) and results for (lines) artisanal cobalt mine production in the DRC. (*B*) Comparison of results for artisanal-production-A and artisanal-production-B by quantity (purple lines) and share of total DRC mine production (blue lines) (*C*) Artisanal cobalt mine production results for artisanal-production-A, dark purple area; total DRC cobalt mine production, light blue area; total world cobalt mine production, dark blue area. Artisanal-production-A as a percentage of world and DRC mine production, light and dark rose lines on the *Right* axis, respectively.

Fig. 3*A* shows the artisanal cobalt production estimates reported in the literature, the results generated by each method depicted in Fig. 1, and the annual average price of cobalt metal deflated to 2020 dollars (8). Fifty-three artisanal cobalt-mining estimates are available in the literature (23–25, 27, 29–41). Except for 2007 and 2008, multiple estimates are reported for each year. The reported estimates from the literature are presented visually as dots as a scatter plot in Fig. 3*A* by reporter type (government, industry, or NGO). Fig. 3*B* compares artisanal-production-A and artisanal-production-B by quantity and by their share of total DRC cobalt mine production. Fig. 3*C* then shows artisanal-production-A as a percentage of world and DRC cobalt mine production.

The results for artisanal-production-A (also referred to as nonindustrial production) are presented in Fig. 3*A* as the solid purple line. They indicate that artisanal-production-A increased from 2,000 metric tons (t) in 2000 to 12,000 t in 2005. This increase was largely due to increasing Chinese demand for cobalt refinery feed; at the same time, increases in industrial DRC cobalt mine production were hampered by a lack of foreign investment (8). Except a dip to 6,000 t in 2013, artisanal-production-A fluctuated between 9,000 and 17,000 t per annum (tpa) from 2005 through 2020. During this period, artisanal-production-A peaked at 16,000 t in 2010, then again at 17,000 t in 2015 and 2018. Only the 2018 peak corresponds with a spike in the cobalt price. But each peak roughly corresponds with the discovery of a surface (heterogenite) deposit that artisanal miners can exploit (31, 32, 42, 43) and an annual increase in Chinese refinery production of 8,000 t or more (8). From 2005 through 2020, artisanal production hit its lowest points at 6,000 t in 2013 and 9,000 t in 2016 and 2020. Although the causes of such declines are currently unknown, these years do correspond with troughs in the price of cobalt.

The results for artisanal-production-B (also referred to as artisanal processing and imports) are presented in Fig. 3 *A* and *B* as the dotted lavender line. Like artisanal-production-A, artisanal-production-B peaks in 2005, 2010, 2015, and 2018, as well as bottoming out in 2013, 2016, and 2020. The annual trends for artisanal-production-B were similar to those of artisanal-production-A, with the sign of annual change (positive or negative) for artisanal-production-A and -B being the same for 17 of the 20 y (Dataset S11d). From 2000 through 2020, the average difference between artisanal-production-A and -B was 800 tpa (1%) with a minimum difference of zero (0%) and a maximum of 5,000 tpa (67%) (Dataset S11d).

Similar to artisanal-production-A, artisanal-production-B increased from 2,000 t in 2000 to 11,000 t in 2005 due almost entirely to the increase in artisanal Chinese imports from 1,000 t to 10,000 t (Dataset S10). Artisanal-production-B then increased to 17,000 t in 2008, again due to an increase in artisanal Chinese imports to 14,000 t. From 2009 through 2020, this relation switched with artisanal processing exceeding artisanal Chinese imports for all but 1 y. For example, while artisanal Chinese imports fell 11,000 t from 2008 to 2009, artisanal processing increased from 3,000 t to 10,000 t as the result of investments by predominantly Chinese and Indian companies in processing (but not) mining facilities (Dataset S4b). The peaks in 2015 and 2018 were due to increases in both artisanal processing and artisanal Chinese imports, while nadirs in 2016 and 2020 were both due to decreases in artisanal processing and artisanal Chinese imports. Like the decline in artisanal-production-A from 2018 to 2020, artisanal-production-B fell from 21,000 t to 11,000 t. From 2000 through 2006, the results of artisanal-production-B were on average 1,400 tpa (31%) lower than those of artisanal-production-A. Given the low quantities for these early years, this difference may be immaterial. From 2007 to 2020, artisanal-production-B was equal to or greater than artisanal-production-A for all years except one, with an average of 1,900 tpa (17%) higher. This could be due to unreported Chinese imports from industrial operations, unreported artisanal feed for industrial mining operations, or some combination thereof.

As for the artisanal production estimates from the literature, the government estimates from 2000 through 2005 come from the U.S. Geological Survey (USGS) (36, 37). The estimate for 2006 is from the World Bank (25). No government estimates were located for 2007 or 2008. Estimates from 2009 through 2019 from the Bundesanstalt für Geowissenschaften und Rohstoffe or BGR (23, 24). The industry estimates from 2009 through 2020 are reported by CRU, a commodities research company (44, 45), as well as from Darton Commodities (a cobalt research and trading company) for the years 2013 through 2020. The NGO references providing artisanal production estimates include TRACeability of hEterogenite or TRACE (34), Global Witness (9), the OECD^1^ (27), PACT (35), and Save the Children (46). See Dataset S1 for details. Despite the absence of an estimation methodology, government, industry, and NGO estimates have (until now) been the only artisanal mine production estimates available to the public. Given that these estimates seek to measure the same quantity of artisanal cobalt production as the results of the two methods, these estimates are compared with the results below.

In relation to artisanal-production-A, the annual difference between production estimates reported by government references is 2,800 t (i.e., 27%) lower on average, with a range from 7,000 t (−67%) lower to 1,000 t (17%) higher. The largest differences were for the peak years of 2005, 2010/2011, and 2015. Annual changes for government estimates and artisanal-production-A are the same sign for 12 of the 15 available years, indicating similar overall trends. Industry references are 700 t (7%) lower on average than those of artisanal-production-A, with a range from 6,000 t (−42%) lower to 7,000 t (41%) higher. Like government estimates, the largest differences were for the peak years of 2015 and 2018. Annual changes for industry estimates and artisanal-production-A are the same sign for all 11 of the 11 available years. In contrast to government and industry references, no NGO references were lower than those of artisanal-production-A. Estimates from NGO references were 8,900 t (120%) higher on average, with a range from 4,000 t (27%) higher to 22,000 t (300%) higher.

In relation to artisanal-production-B, production estimates reported by government references were 18% lower on average, with a range from 58% lower to 100% higher. Industry references were 19% lower on average, with a range from 55% lower to 25% higher. NGOs were 209% higher on average, with a range from 41 to 800% higher. These results indicate that, on average, NGOs report cobalt production 2.2 to 3.1 times larger than that of other references. On the contrary, government, industry, artisanal-production-A, and artisanal-production-B estimates have no more than an average difference of 27%. The references above have the same sign of annual changes from 80 to 100% of available years, while NGO references range from 36 to 64%. These results may indicate that government and industry references are using similar methods of estimation to artisanal-production-A and -B. Alternatively, it may mean that government and industry have access to authoritative references whose production information has been approximated by artisanal-production-A and -B.

### Results and Literature Estimates as a Percentage of Total World and DRC Cobalt Mine Production.

Fig. 3*C* presents artisanal-production-A as estimated artisanal production in the context of total DRC and world cobalt mine production. The quantity of world, DRC, and artisanal-production-A is presented inFig. 3*C* as unstacked cobalt blue, sky blue, and dark purple areas, respectively, on the *Left* axis. Artisanal-production-A, as a percentage of total world and DRC production, is presented as dark and light rose lines, respectively, on the *Right* axis. Fig. 3*C* shows that artisanal-production-A’s share of total world and DRC cobalt mine production increased from 5% and 18% in 2000 to 18% and 49% in 2005, respectively (Dataset S11b and S11c). During this period, China’s cobalt refinery production increased more than ten-fold. Industrial mines were unable to keep pace with this demand, so most of the rise in total DRC mine production was comprised of artisanal mining. Then, increasing production from industrial mines in the DRC (and elsewhere) reduced artisanal-production-A’s percentage of world and DRC production, which fell from 18% and 49% in 2005 to 6% and 11% in 2013, respectively. With the discovery of new surface heterogenite deposits at the end of 2014, artisanal-production-A’s percentage increased to 14% and 24% in 2015 before trending down to 6% and 9% in 2020. The causes of this reduction were that industrial production nearly doubled from 2015 to 2020 and artisanal production fell from 17,000 t to 9,000 t.

In comparison, government estimates, as a percentage of total world and DRC production, peaked in 2004 at 12% and 35%, respectively. Government estimates then fell over time to 8% and 12% in 2019, which are the same percentages as artisanal-production-A and close to artisanal-production-B results of 10% and 15% in 2019. Industry estimates peaked at 17% and 30% in 2010 (as opposed to 10% and 18% for both artisanal-production-A and -B). In 2020, the average of industry estimates is 6% and 9%, equal to artisanal-production-A and like artisanal-production-B’s 7% and 10%. In other words, the trends and quantities of government and industry estimates were largely in line with the results for artisanal-production-A and -B.

In contrast, the average of NGO estimates, as a percentage of total world and DRC production, peaked in 2004 at 31% and 93%, respectively. The average of 93% is from two NGO estimates, one of 74% of DRC production (34) and the other of 111% (9). It is likely that these are overestimates, given that 65% (as opposed to 26%) of total DRC cobalt mine production in 2004 can be accounted for by just four industrial mining operations (i.e., 700 t from Gécamines, 2,900 t from Big Hill, 5,000 t from Luiswishi, and 4,000 t from Kababankola) (47–49). Neither of these references report a methodology associated with these estimates, so it is not possible to evaluate the causes of such wide variation between the two NGO estimates, the government estimate for the year (47), or the results for artisanal-production-A and -B. For the years from 2000 through 2006 (the years in the 2000s where NGO and government estimates are both available), NGO estimates are four times larger than government estimates on average. NGO estimates become available again from 2017 through 2020. In contrast to government estimates, industry estimates, and artisanal-production-A and -B, the average of NGO estimates increases from 15% of world and 24% of DRC cobalt mine production in 2017 to 22% and 32% in 2020. These may also be overestimates, given that over 90% (as opposed to only 68%) of cobalt mine production in the DRC can be accounted for by mechanized extraction at industrial mines.

### Artisanal Processing.

**Fig. 4.**
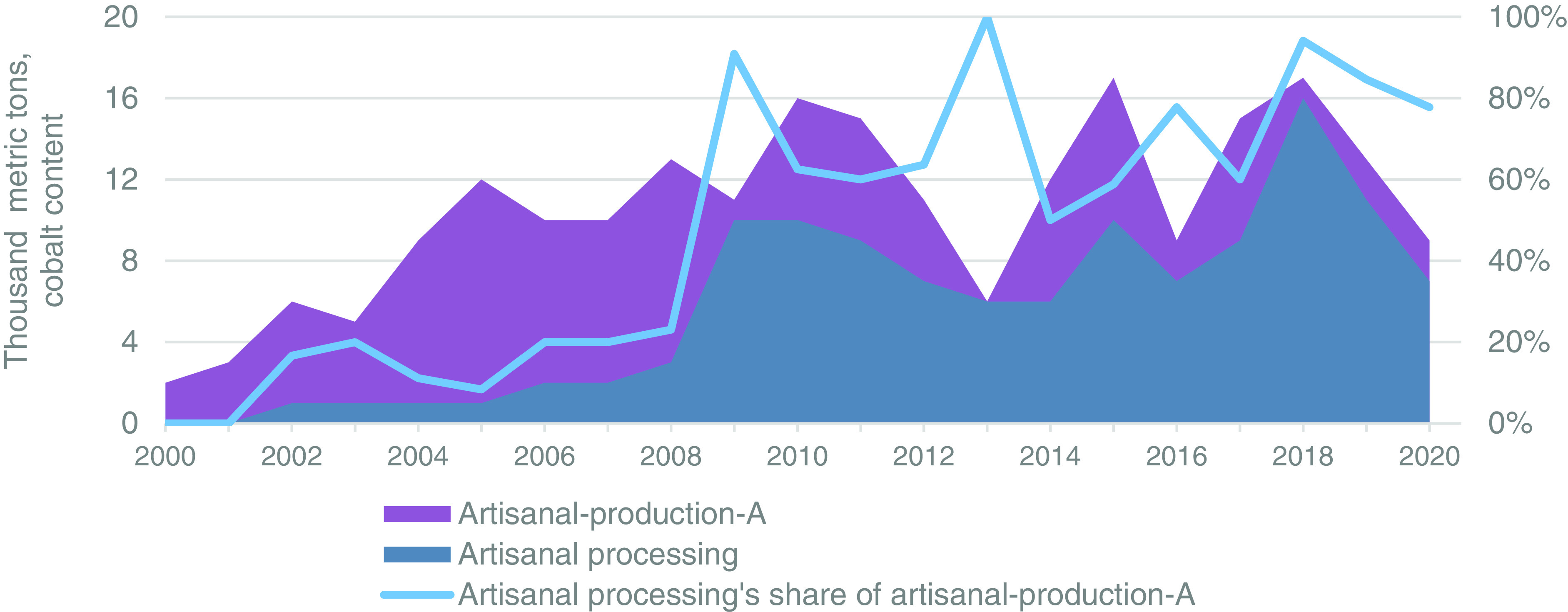
Artisanal-production-A in the Democratic Republic of the Congo (DRC), unstacked dark purple area on the *Left* axis; processing of artisanal production into intermediate cobalt materials (i.e., artisanal processing) in the DRC, blue gray unstacked area, *Left* axis; and artisanal processing as a percentage of artisanal-production-A, light blue line on the *Right* axis.

Fig. 4 shows the results of artisanal processing (unstacked blue gray area) in relation to artisanal-production-A (unstacked dark purple area), with the light blue line being artisanal processing’s percentage of artisanal-production-A. For the years 2000 and 2001, no facilities are reported to process artisanal ores in the DRC. Artisanal processing increases to 1,000 tpa from 2002 through 2005 due to the entry of a single Indian processing firm (Société Miniere du Katanga or SOMIKA). Between 2008 and 2009, artisanal processing increased from 3,000 t to 10,000 t as companies such as Congo Dongfang Mining, Groupe Bazano, Volcano Mining, and Bolfast began production. From 2009 through 2017, artisanal processing fluctuated between 6,000 t and 10,000 t as processing firms faced technical difficulties and responded to market prices. When prices and artisanal production spiked in 2018, processing firms briefly came online, resulting in a spike of artisanal processing to 16,000 t before falling to 7,000 t in 2020. See *Materials and Methods*, as well as Dataset S4a and S4b for methodology, calculations, processing facilities, percentage of artisanal feed sourcing, and references.

Artisanal processing, as a percentage of artisanal-production-A from 2000 through 2008, remained at or below 23% as most artisanal production was exported due to the lack of domestic processing facilities. With the increase in artisanal processing and the decrease in artisanal-production-A from 2008 to 2009, artisanal processing’s share jumped from 23 to 91%. Artisanal processing’s share then fluctuated between 50% and 64% from 2010 through 2015, except for a spike to 100% in 2013 when the result for artisanal-production-A dipped to 6,000 t. As domestic processing capacity expanded, Chinese imports shifted to crude cobalt hydroxide, and responsible sourcing initiatives sought to formalize the artisanal sector, artisanal processing’s share increased to a range of 78 to 94% from 2016 through 2020, except for a brief dip to 60% in 2017.

### Total, Industrial, and Artisanal Chinese Imports from the DRC.

**Fig. 5.**
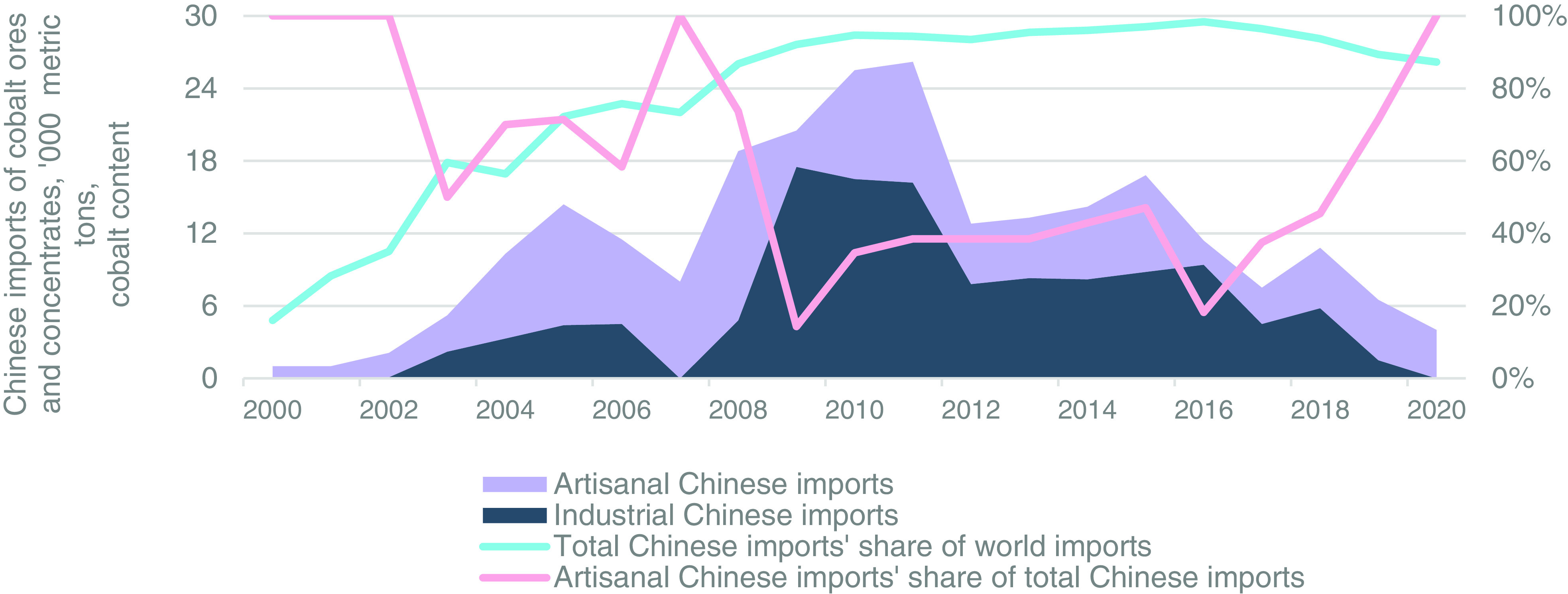
China’s imports of cobalt ores/concentrates from the DRC (imports). Industrial Chinese cobalt ore/concentrate imports, stacked dark blue gray area; artisanal (i.e., nonindustrial) Chinese cobalt ore/concentrate imports, stacked light purple area; total Chinese cobalt ore/concentrate imports’ share of world imports, light turquoise line on the *Right* axis; and artisanal imports’ share of total Chinese imports from the DRC, rose line on the *Right* axis.

China’s total imports of cobalt ores/concentrates from the DRC (referred to as “Chinese imports”) are presented in the stacked area graph of Fig. 5 as the total of industrial (indigo area) plus artisanal imports (light purple area). Fig. 5 also displays total Chinese imports from the world as a percentage of world trade (light turquoise line on *Left* axis) and artisanal imports as a percentage of total Chinese imports from the DRC (pink line on *Right* axis). The results show that to feed China’s growing cobalt refinery industry, total Chinese imports from the DRC grew from 1,200 t in 2000 (i.e., 16% of world cobalt ore/concentrate trade), to 6,400 t in 2003 (60% of world trade), to 15,100 t in 2005 (72% of world trade), and to 26,600 in 2010 (95% of world trade). Chinese ore/concentrate imports then fell from 2012 through 2016, averaging 14,100 tpa (96% of world trade). This decrease was predominantly due to increasing substitution of China’s refinery feed imports from ores/concentrates to crude cobalt hydroxide. From 2017 through 2020, Chinese imports fell further with an average of 7,300 tpa (92% of world trade). See *Materials and Methods* and Dataset S5 for details, references, and results.

Of total Chinese imports from the DRC in Fig. 5, the results demonstrate that industrial Chinese imports increased from 0 t in 2000 to 4,500 t in 2006, were officially banned by the DRC government in 2007, and increased from 4,800 t in 2008 to 17,500 t in 2010. Industrial Chinese imports then generally fell as industrial imports transitioned from ores/concentrates to intermediate cobalt products such as crude cobalt hydroxide. After declining to an average of 8,500 tpa from 2012 through 2016, these imports fell to 0 t in 2020. For references, numerical estimates, and the companies reported to provide industrial imports, see Dataset S8a for 2000 through 2008 and Dataset S9 for 2009 through 2020.

Artisanal (i.e., nonindustrial) imports, on the contrary, are comprised of total Chinese imports from the DRC minus industrial Chinese imports. The results for artisanal imports in Fig. 5 show an increase from 1,000 t in 2000 to 14,000 t in 2008, which reflects increasing Chinese demand for cobalt refinery inputs while the DRC’s industrial mine production struggled to recover. Artisanal imports then decreased to an average of 6,600 tpa from 2009 through 2015 before falling farther to an average of 3,800 tpa from 2016 through 2020. The decline in artisanal imports reflects both increased artisanal processing and increasing crude cobalt hydroxide imports from the newly recapitalized industrial mining industry. For references, calculations, assumptions, and numerical estimates, see Dataset S8 for 2000 through 2008 and Dataset S9 for 2009 through 2020.

As a percentage of total Chinese imports from the DRC, artisanal imports of the years 2000, 2007, and 2020 stand out. As mentioned, in 2000, the DRC’s mining industry was just restarting and little industrial concentrate supply was available, which is why Chinese imports for 2000 were artisanal. This trend continued from 2000 through 2007 as artisanal imports averaged 77% of total Chinese imports from the DRC. Artisanal imports’ share spiked again to 100% in 2007 because almost all exports from industrial operations were prevented during that year by the DRC raw material export ban. This share then fell to an average of 39% from 2008 through 2017 as industrial operations expanded ore/concentrate production. It then increased to 100% in 2020 as Boss Mining, Metal Mines, and COMIKA ceased industrial ore/concentrate exports, which were replaced by crude cobalt hydroxide from industrial mines in 2020. Again, for references, calculations, assumptions, and numerical estimates, see Dataset S8 for 2000 through 2008 and Dataset S9 for 2009 through 2020.

**Fig. 6.**
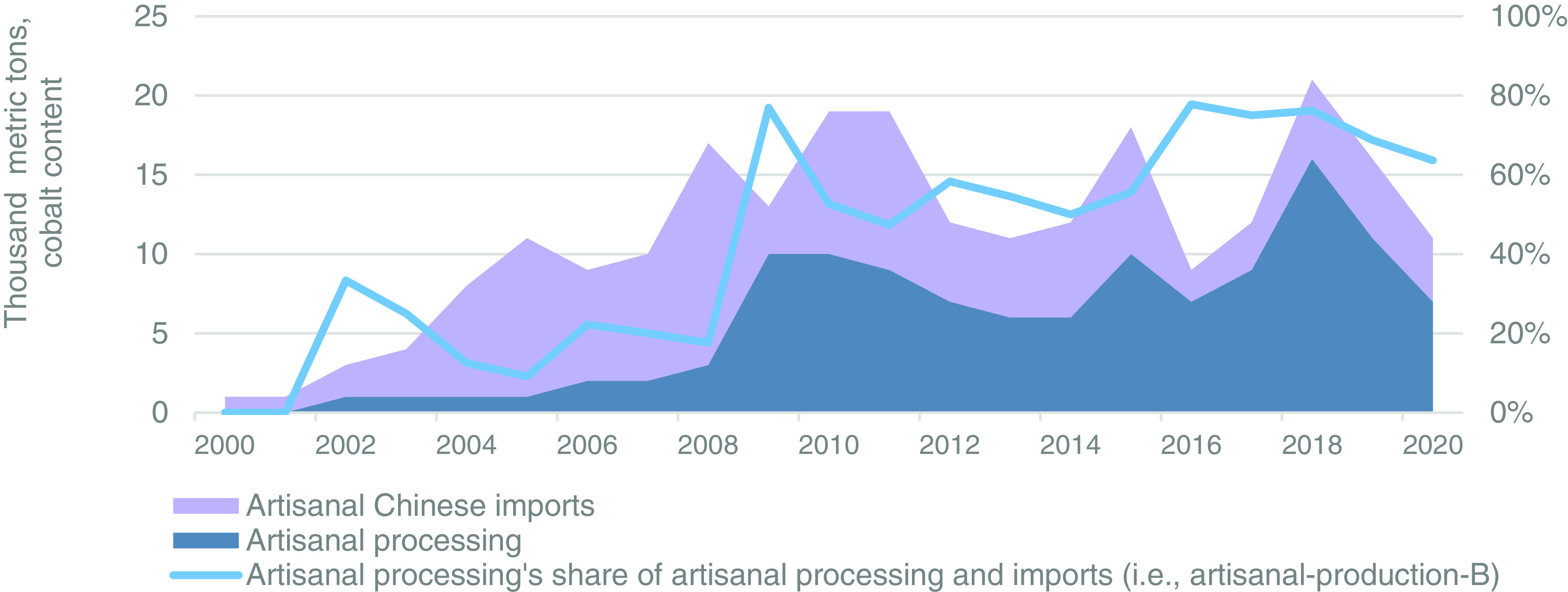
Artisanal processing, stacked blue gray area; artisanal Chinese ore and concentrate imports, stacked light purple area; and artisanal processing as a percentage of artisanal processing and imports (i.e., artisanal production), light blue line on the *Right* axis.

The artisanal-production-B results from the artisanal processing plus artisanal imports (i.e., artisanal production) are presented in Fig. 6, along with artisanal processing’s share of artisanal-production-B. Given that the results for artisanal-production-B and China’s artisanal imports have already been discussed, this section focuses on the percentage comprised of artisanal processing. From 2000 through 2008, artisanal processing’s share of artisanal-production-B averaged 16%, in comparison to 13% for artisanal-production-B. This share increased to 77% in 2009 as artisanal processing increased from 3,000 t to 10,000 t and artisanal imports fell from 14,000 to 3,000 t. From 2010 through 2015, artisanal processing and imports were balanced, even despite significant fluctuations, resulting in an average share for artisanal processing of 53%. These results reflect a transition period from artisanal imports to artisanal processing that is reflected in artisanal processing’s average share of 72% from 2016 through 2020.

### Key Data and Methodological Uncertainties.

Assumptions, uncertainty, and data issues that may impact the results of the two estimation methods are briefly presented here. For more detail, see *Materials and Methods* below, as well as *SI Appendix*, *Supporting Discussion 1*. Method A relies on: the accuracy of total historic DRC production estimates and of historic production estimates for industrial operations. Total DRC cobalt mine production data are from the cobalt chapter of the USGS Minerals Yearbook (MYB). In the field of minerals information, the USGS MYB is widely recognized as the best publicly available source of mineral production data and is routinely cited by prominent articles in the field. Industrial mine production data (whether referenced to USGS MYBs, Darton Commodities, or elsewhere) primarily originate from financial reports of multinational industrial mining companies. Rather than gathering each data point from each financial report, this analysis references several “aggregation reports”—such as the USGS MYB and Darton Commodities—that collect and rereport individual mine production data in a comprehensive and readily available format. See Datasets S1 and S2 and *SI Appendix*, *Supporting Discussion 1* for details and references.

As for method B, in contrast to industrial mine production data originally reported by large companies in financial statements, the processing production data chiefly originate from monthly or annual reports titled “Statistics of debit notes relating to the mining royalty issued” that are issued by the Katanga (now Lualaba and Haut Katanga) Division of the DRC Ministry of Mines. The balance of processing production data originates from industry expert estimates or sporadic reports from the companies themselves. Rather than gathering each data point from each report, this analysis again relies on several of the aggregation reports mentioned above. To facilitate replication of this analysis, and to make the DRC Ministry of Mines reports more available to the public, those that were found were provided at the end of *SI Appendix*.

This analysis multiplies each processing facility’s production data above by the portion of each facility’s ore/concentrate feed that comes from artisanal mining. It is difficult to corroborate processing production data (first subcomponent of artisanal processing), as well as the portion of artisanal feed (second subcomponent). This introduces uncertainty for the artisanal production component of method B that is not insignificant. As such, Dataset S4 presents the underlying data so that it may be replicated, updated, or revised by future researchers.

The second component of method B (Chinese artisanal imports) also relies upon two subcomponents. The first is total Chinese cobalt ore/concentrate imports from the DRC. The gross weight trade data come from China Customs. While China Customs serves as the best publicly available Chinese import data, the assumed cobalt content presents more uncertainty. Different mines have different cobalt contents that may vary year to year (Dataset S9b). A constant estimate of 7.6% was used based on reports from authoritative Chinese sources, average content of industrial mines, and reported content of artisanal production. See *SI Appendix*, *Supporting Discussion 1* for details and references. This introduces uncertainty into estimates of the cobalt content of Chinese ore/concentrate imports from the DRC.

The next subcomponent is the quantity of Chinese ore/concentrate imports from the DRC that were exported by industrial mines. For the accurate allocation of China’s imports from industrial operations in the DRC, there is less data available for 2000 through 2008 than for 2009 through 2020. From 2000 through 2008, there were relatively few industrial mines in the DRC and most supplied refining companies that were under the same parent company. This offsets the relative lack of data from 2000 through 2008. Beginning in 2009, the DRC Ministry of Mines began reporting the DRC’s exports of cobalt ores/concentrates by company and cobalt material type. This improved the detail and confidence with which Chinese imports from industrial mines in the DRC can be estimated from 2009 through 2020. To allow future researchers to replicate, update, or revise this piece of method B, Dataset S9 presents each company’s reported gross weight of exports, its cobalt content, whether it was industrial or artisanal, and the country assumed to import that material. If all imports from industrial mining operations were not properly accounted for, the estimate of artisanal cobalt imports would be higher than it should be. Potential underestimation of artisanal cobalt processing (discussed above) and potential overestimation of artisanal cobalt imports may help to balance each other during the early 2000s when reporting was less reliable.

Given that method A relies on total DRC mine production and industrial mine production data, while method B relies on processing production and import data, it would not be expected that the two methods would produce the same results. As discussed above, method A only has two components, both of which exhibit relatively lower uncertainty. Method B, on the contrary, has two components—which themselves each have two subcomponents. Three of the four subcomponents in method B have data issues which lead to uncertainties. As a result, method A may better approximate artisanal mine production than method B.

## Discussion

This analysis gathers 53 artisanal cobalt production estimates from 10 government, industry, and NGO references for the years from 2000 through 2020. This analysis also develops two methods of estimating artisanal cobalt production, which rely on different datasets, assumptions, and techniques. Despite these methodological differences, artisanal-production-A is only 1% less than artisanal-production-B on average from 2000 through 2020. The resulting estimates also reflect the literature estimates from government and industry references, which are on average 18% less than those of artisanal-production-A and -B from 2000 through 2020. NGO estimates, on the contrary, are 2.2 to 3.1 times larger than those of government, industry, artisanal-production-A, and artisanal-production-B. The methods developed in this analysis provide continuous datasets that may be used for future analyses of artisanal cobalt mining and related subjects. In addition to providing historical estimates, the methods allow the estimation of artisanal production for each new year. Future econometric analyses may use these results to evaluate predictors of artisanal cobalt production including price, time, and rainfall (all readily available), as well as deposit depletion, artisanal regulations, access to industrial concessions, world/middlemen price differentials, or responsible sourcing guideline implementation (not currently quantified or available).

To convey the key insights of this analysis, a metaphor is used where the cobalt supply chain is likened to a river and artisanal production with child labor (and other exploitative practices) is likened to polluted headsprings. The argument could be made that all artisanal cobalt mining involves some level of exploitation, and that the entire quantity of artisanal cobalt production is polluted^*^. Like streams flowing through water gauges that measure the quantity and quality of the water passing through, the results reveal several monitoring points immediately downstream of the headsprings from which artisanal production springs from the ground. Since 2000, an increasing portion of artisanal cobalt mine production is flowing through mostly Chinese-owned facilities in the DRC that process cobalt ores and concentrates into intermediate cobalt materials such as crude cobalt hydroxide (Figs. 3 and 5). From 2016 through 2020, between 72% and 79% of artisanal production (on average) passed through these facilities. From these facilities, one may track artisanal cobalt trade upstream to artisanal mine sites to evaluate questions such as where are the miners, what are the conditions, how many are there, and how many are children. In the other direction, one may track the sale of intermediate materials downstream to answer other questions such as what refiners are purchasing these materials, where are they, and who are their customers. Such an approach would be similar to that employed in US regulations regarding the DRC’s artisanal production of the so-called conflict minerals tin, tantalum, tungsten, and gold (26).

More broadly, the results show that the flow of total artisanal production (i.e., processed in the DRC and exported to China) comes mostly from “headwaters” chiefly controlled by Chinese processing and refining firms. Such firms may represent an additional gauge through which artisanal production passes. Chinese imports of cobalt ores and concentrates averaged 60% of world ore and concentrate trade from 2000 through 2009 and 94% from 2010 through 2020. Of China’s total cobalt ore and concentrate imports, 71% is estimated to have been artisanal from 2000 through 2009 and 47% from 2010 through 2020.

Seventy-nine percent of cobalt refined in China was used to produce battery-grade cobalt chemicals (40). These Chinese refiners, which produce nearly all of the world’s battery-grade cobalt chemicals (40), sell their output to battery cathode producers (often different subsidiaries of the same Chinese firms), then cell manufacturers, then pack manufacturers, then battery manufacturers, then OEMs (1). While no amount of child labor or other abuses related to artisanal mining in the DRC is acceptable, the likelihood of OEMs sourcing cobalt from such mining has been increasingly diluted by expanding industrial production in the DRC and elsewhere in the world. Artisanal cobalt mining, as a percentage of DRC cobalt mine production in 2020, was estimated to be between 9% and 11%. In other words, if a cobalt-refining firm were to randomly source cobalt mining material from the DRC, there was roughly a 90% chance that the material would be nonartisanal. If, as was estimated (24), one in four artisanal cobalt mine sites had children working—and all artisanal sites produced the same amount of cobalt—there would be a 98% chance that randomly selected cobalt mine material from the DRC was **not** linked to child labor in 2020.

Although these results will change with future artisanal production estimates and artisanal mine site studies, the current results suggest that, rather than being a “cobalt problem,” child labor in artisanal cobalt mining may be more of a “development problem.” Many downstream OEMs are finding it difficult or impossible to substitute cobalt without increasing the risk of batteries catching on fire or without reducing performance (such as long-range electric vehicles) to an extent that could impact full deployment (19, 41). Cobalt is often portrayed as being mined by child miners to produce the lithium-ion batteries in our smartphones and electric vehicles (42, 50–52)—resulting in calls to remove cobalt from batteries entirely (18). In terms of the river metaphor, this would be equivalent to trying to divert 63% (in 2020) of the water at the river’s estuary, rather than addressing the problem at its source.

## Materials and Methods

### Method A: Total DRC, Industrial, and Nonindustrial DRC Cobalt Mine Production.

Method A estimates artisanal production by subtracting the production of industrial mines from total reported DRC cobalt mine production (middle column of Fig. 1). The result is an estimate of nonindustrial production (i.e., artisanal-production-A). Total DRC cobalt mine production for year *t* is reported at the country level. Industrial production for year *t* is the sum of industrial mine *i*’s reported production—from its own deposits—for 1 to *n* industrial mines. Industrial production is calculated in Eq. **1**. The remaining portion of total DRC mine production is comprised of nonindustrial production for year *t.* Nonindustrial production is calculated in Eq. **2**, and presented as the middle column of Fig. 1.

[1] $$\begin{array}{c} {Industrial\;production}_{t}\\ =\sum_{i=1}^{n}{Reported\;production\;for\;DRC\;industrial\;cobalt\;mine}_{\mathrm{it}} \end{array}$$

[2] $$\begin{array}{c} Nonindustrial\;production_{t}=Artisanal\;production\;A_{t}\\ =total\;DRC\;mine\;production_{t}-Industrial\;production_{t} \end{array}$$

The mine-level data relied upon to estimate nonindustrial production are detailed by row in Dataset S2. In terms of data reliability, country-level cobalt mine production is reported annually in the USGS MYB, which has been published each year since at least 1932 (53). In addition to accounting for the sum of industrial mine production (reported in the financial reports of each mining company), the USGS MYB incorporates data on total mine production and exports reported by the DRC Ministry of Mines, total imports reported by the DRC’s trade partners, and mine production reported by cobalt market experts. The USGS MYB is often considered to be an authoritative reference of mineral production statistics, has been the data reference for seminal works within the mineral commodity field, has served as the data for methods of evaluating minerals critical to the United States, and is updated to reflect new information for 5 y after the original estimate was made (4, 54, 55). Given these factors, total DRC cobalt mine production may be viewed with confidence. Similarly, production at the level of individual mining operations, as well as major investments, developments, and production at industrial mine operations in the DRC, are reported in the DRC and Cobalt chapters of the USGS MYB, which collect most of this information from industrial mining company financials and data from the DRC Ministry of Mines. This mine-level information can often be corroborated by industry publications written by cobalt traders and analysts with a deep understanding of the market. Within this context, overestimation of artisanal-production-A could result if there were industrial mine production that was not captured in Dataset S2. Alternatively, underestimation could result if industrial mining operations supplemented their mined material with artisanal feed. Both possibilities are more likely for the earlier years (i.e., 2000 through 2008) than the later years (i.e., 2009 through 2020).

### Method B: Overview.

In method B, artisanal production is assumed to be comprised of one portion that is processed into intermediate cobalt materials at facilities in the DRC and another portion that is sent to China (right column of Fig. 1). Due to China’s rapidly increasing cobalt refinery production from 2000 through 2020, China imported most of the world’s cobalt ore and concentrate trade (56). At the beginning of this period in 2000, in contrast to the newly developing refiners in China, existing cobalt refiners in other countries had already established long-term supply agreements and did not need to seek out new feed supply sources. Given the lack of sufficient cobalt mine production in China and the dearth of available refinery feed supplies from operational industrial mines, Chinese trading firms came to the DRC in the early 2000s to secure cobalt materials and export them to China (25, 38). Due to the collapse of the DRC economy and mining industry in the 1990s, artisanal ores and concentrates were the most readily available cobalt feed available to be exported to China (25, 38). Government, industry, and NGO references all report artisanal production to predominantly be shipped to China (9, 10, 12, 25, 41).

### Method B: Artisanal Processing.

As mentioned above, many facilities in the DRC are reported to process artisanal cobalt ores and concentrates into intermediate cobalt materials including concentrate, cobalt copper alloy, crude cobalt carbonate, and crude cobalt hydroxide. To estimate artisanal processing, this analysis multiplies the quantity produced at the facility by the percentage of feed sourced from artisanal production (often 100%), as calculated in Eq. **3**,

[3] $$\begin{array}{c} {Artisanal\;processing}_{t}=\sum_{j=1}^{o}{(intermediate\;processing\;production}_{j}\;\\ {}*\;{artisanal\;feed\;share}_{j}) \end{array}$$

where *intermediate processing production_j_* represents intermediate cobalt production reported for DRC processing plant *j* and *artisanal feed share_j_* represents processing plant *j*’s share of feed from artisanal production from 1 to *o* number of processing plants. Processing-facility-level production data, artisanal feedstock share, and the resulting calculation of artisanal processing results are presented and referenced by facility in Datasets S4 and S8. As to the reliability of the data required to estimate artisanal processing, it is likely that some operations that were not reported to be processing artisanal ore were, in fact, processing artisanal ore. This is especially likely in the 2000s when there was less attention on artisanal cobalt mining in the DRC. This uncertainty would result in underestimating artisanal ore processed in the DRC which would cause underestimation of the results for artisanal-production-B.

### Method B: Total Chinese Imports from the DRC.

To estimate the cobalt content of total Chinese imports of cobalt ores/concentrates from the DRC, this method relies upon import data from China Customs (57). These data are reported in gross weight under the tariff code 260500 and were obtained from IHS’s Global Trade Atlas (57). To convert gross weight to cobalt content, these reported trade volumes are multiplied by the reported average cobalt content of 7.6% (2). China’s cobalt ore and concentrate imports from countries that neighbor the DRC, but do not have cobalt mine production, are assumed to come from the DRC. Examples of such “transit” countries are the Republic of the Congo (Brazzaville), Zambia, and South Africa. Chinese imports via transit countries were predominantly in the early 2000s. See Dataset S5 for trade data reported by China Customs.

Some specialists may be aware of skepticism about the accuracy of China’s official reported statistics. Such skepticism has largely pertained to official statistics regarding GDP growth (58–64), but to a lesser extent, some have also questioned trade statistics (61, 64). As such, references are provided here for the reader that may be of interest in this literature, which has shown that China’s trade statistics broadly align with trade statistics from other countries (61, 64). To illuminate this question within the context of this analysis, several Supporting Datasets are provided for specialists to compare China Customs’ data with that from other reporters. Dataset S6 presents reported cobalt ore/concentrate exports to China from the customs ministries of the DRC and likely transit countries. The DRC Customs Ministry has historically been underfunded (25) and was not able to publish such export data until 2015. Dataset S7 presents cobalt ore/concentrate imports from the DRC as reported by the customs ministries of the DRC’s trading partners. A comparison between the totals of Datasets S6a and S7a may indicate that the customs ministries of the DRC and transit countries have not accounted for all cobalt ore/concentrate exports to all their trading partners, let alone China. A final comparison of China Customs’ reported cobalt ore/concentrate imports from the DRC is provided in Dataset S9, which contains the DRC’s exports of cobalt ores/concentrates by company, as reported by the DRC Ministry of Mines provincial divisions for Katanga, Haut Katanga, and Lualaba. These data, however, are only available from 2009 through 2020. As a result, this analysis relies on import data reported by China Customs as the best publicly available information for calculating total Chinese imports of cobalt ores/concentrates from the DRC.

### Method B: Industrial and Artisanal Chinese Imports from the DRC.

Of China’s total ore/concentrate imports from the DRC, one portion is imported from industrial miners in the DRC. The only data available to estimate industrial Chinese imports from 2000 through 2008 come from refs. 36, 65, and 66 that report industrial concentrate production from Kamoto Copper Company (whose operations are now owned by Glencore) and from Boss Mining (now owned by Eurasian Resources Group) that were sent to China (Dataset S9a). However, more detailed data became available for this purpose beginning 2009. Industrial Chinese imports for the years 2009 through 2020 rely on: gross weight company-level export data reported by the DRC Ministry of Mines provincial divisions (Dataset S9a), cobalt content reported by the Extractive Industry Transparency Initiative (Dataset S9b and S9c), company type of production—industrial, artisanal, or artisanal processing—(Dataset S9d), and export destination (Dataset S9e). When China is the export destination in Dataset S9e and the company type of production is industrial in Dataset S9d, then the cobalt content of the reported export in Dataset S9c is assumed to be an industrial Chinese import. Summing the cobalt content of industrial exports to China results in the estimate of industrial Chinese imports.

Mathematically, Eq. **4** calculates industrial imports for year *t* as the sum of China’s imports from DRC industrial mine *i* from 1 to *p*. Eq. **5** shows that artisanal imports for year *t* are calculated as China’s total imports from the DRC for year *t* minus industrial imports for year *t*. Eq. **6** calculates artisanal-production-B as artisanal processing plus imports for year *t* as artisanal processing for year *t* plus artisanal imports for year *t*.

[4] $$\begin{array}{c} {Industrial\;imports}_{t}\\ =\sum_{i=1}^{p}{China^{'}s\;ore\;and\;concentrate\;import\;reported\;by\;DRC\;industrial\;miner}_{i} \end{array}$$

[5] $${Artisanal\;imports}_{t}={Total\;imports}_{t}-{Industrial\;imports}_{t}$$

[6] $$\begin{array}{c} Artisanal\;processing_{t}=\;Artisanal\;production\;B_{t}\\ =Artisanal\;processing_{t}+Artisanal\;imports_{t} \end{array}$$

## Supplementary Material

Appendix 01 (PDF)

Dataset S01 (XLSX)

Dataset S02 (XLSX)

Dataset S03 (XLSX)

Dataset S04 (XLSX)

Dataset S05 (XLSX)

Dataset S06 (XLSX)

Dataset S07 (XLSX)

Dataset S08 (XLSX)

Dataset S09 (XLSX)

Dataset S10 (XLSX)

Dataset S11 (XLSX)

Dataset S12 (XLSX)

Dataset S13 (XLSX)

## Data Availability

All study data are included in the article and/or Supporting Appendices and Datasets.

## Footnotes

^*^For example, per unit of production, artisanal cobalt mining has been found to produce higher levels of literal pollution than those of industrial mining (67–69). This often results in higher levels of birth defects and other illnesses near artisanal mine sites (67–69), an outcome which is found to be true for artisanal mining of other metals (67).
