# Supplementary material for: China, the Democratic Republic of the Congo, and artisanal cobalt mining from 2000 through 2020
Source: Proc Natl Acad Sci U S A. 2023 Jun 20;120(26):e2212037120. doi: 10.1073/pnas.2212037120 (PMC10293843; doi:10.1073/pnas.2212037120)
Supplement: Supplementary file 1 — Appendix 01 (PDF) [file pnas.2212037120.sapp.pdf]

Supporting Information for China, the Democratic Republic of the Congo, and artisanal cobalt mining from 2000 through 2020

Andrew L. Gulley
Email: agulley@usgs.gov

This PDF file includes:

Supporting text in Supporting Discussions 1-2
Supporting Discussion References
Supporting Datasets 1-13
Supporting data from Katanga, Haut Katanga, Lualaba provincial export, sales, production statistics from 2009 through 2020

Supporting Information Text

Supporting Discussion <u>1</u>: Assumptions and variables that may impact the results of the two estimation methods

Assumptions that may impact the results of the two estimation methods are presented here for context and clarity.

Method A

Method A relies on: the accuracy of total historic DRC production estimates, the assumption that all industrial mine operations are known, and the accuracy of historic production estimates for industrial operations. Total DRC cobalt mine production is reported and updated based on new information for five years after the fact. In addition to accounting for the sum of industrial mine production (reported in the financial reports of each mining company), the USGS MYB incorporates data on: total mine production and exports reported by the DRC Ministry of Mines (51), total imports reported by the DRC's trade partners (52), and experts on the world cobalt market (42, 50, 53, 54). In the field of minerals information, the USGS MYB is recognized as the best publicly-available source of minerals production data and is widely cited by prominent articles in the field (4, 55–59). Confidence in total DRC cobalt mine production reports from the USGS Minerals Yearbooks is therefore considered here to be high.

Similarly, production at the level of individual mining operations, as well as major investments, developments, and production at industrial mine operations in the DRC are reported in the DRC and cobalt chapters of the USGS Minerals Yearbooks. This facility-level information is corroborated by industry publications written by cobalt traders who have a deep understanding of the market. In addition to listing industrial mining operations in the DRC, the USGS Mineral Yearbooks and industry sources often report the feed sources for these operations. Given how regularly and reliably each component of method A is reported, overall confidence in the results of method A may be regarded to be high. As a result, method A's results serve as the estimation of artisanal production in Figure 2c—showing artisanal cobalt production as a share of total DRC and world cobalt mine production.

Method B: Artisanal processing

Method B relies on: the accurate estimation of artisanal processing, the accurate estimation of the cobalt content of Chinese imports of ores/concentrates, as well as the accurate identification and quantification of China's imports reported to be from industrial mining operations. It is likely that some operations that were not reported to be processing artisanal ore were, in fact, processing artisanal ore. This is especially likely in the 2000s when there was less attention on artisanal cobalt mining in the DRC. This uncertainty would result in underestimating artisanal ore processed in the DRC which would cause underestimation of the results for method B.

Method B: Total Chinese Imports

The estimation of China's imports of ores and concentrates depends on import data that is routinely reported by China Customs (1), which serves as the best publicly available Chinese import data. Skepticism regarding the reliability of China's official statistics and economic data has sparked a literature of its own since 2000 (2–8). These studies have focused almost exclusively on the estimation of China's gross domestic product (GDP) and China's growth rate, due to the importance of China's growth to the world economy. The studies in this literature were largely in response to assertions in the popular media that China was overstating or understating its growth due to the methods used to calculate GDP (8). A 2017 meta-analysis of this literature found 68% of the studies reviewed were critical of various official statistics used to calculate GDP,

while the other 32% concluded that China's official statistics were broadly accurate based on internal consistency (such as provincial versus national data), comparison with data reported by non-Chinese authorities, and adherence to international standards in the methodologies used (8).

However, this raises the question of whether criticisms of China's official statistics, used to calculate GDP, apply to trade data. Of the studies cited above only two address China's trade statistics (5, 8). Both found the statistics reported by China Customs to be broadly aligned with trade statistics reported by other countries and indicated that manipulation of trade data was less likely given that there is often another country calculating the same trade flow (5, 8). As discussed in the Methods section, Datasets S6 and S7 show that the exports reported by the DRC Customs Ministry, as well as those of potential transit nations, cannot be relied upon because they only begin in 2015 and they do not match up with what is known about DRC exports to China from 2015 through 2020. Therefore, no matter the potential issues, the import data from China Customs serve as the best publicly available source of Chinese cobalt ore/concentrate import data.

In addition to relying on the gross weight of imports reported by China Customs, the estimation of China's imports also relies upon the assumed cobalt content of these imports which is derived from reliable and routinely reported estimates from the authoritative data sources for China's cobalt industry (9–16). This estimate of the cobalt content of Chinese ore/concentrate imports also corresponds with the average of cobalt contents reported for industrial mining operations (such as Boss, Mutanda, Ruashi, and Tenke Fungurume), as well as reported cobalt contents for artisanal production (17–19).

Method B: Industrial and artisanal Chinese Imports

As for the accurate allocation of China's imports to industrial operations in the DRC, there is less data available for 2000 through 2008 than for 2009 through 2020. From 2000 through 2008 export destinations are not reported or quantified with the same dependability as the production and import estimates discussed above. During this period, however, there were relatively few industrial mines in the DRC and most supplied refining companies that were under the same parent company. In terms of uncertainty, it is not possible to tell if this is more likely to result in over or underestimation during these years. Beginning in 2009, however, the DRC Ministry of Mines provincial divisions for Katanga began reporting the DRC's exports of cobalt ores/ concentrates by company. This improves the detail and confidence with which industrial Chinese imports can be estimated from 2009 through 2020. See Dataset S9 for details.

If all imports from industrial mining operations were not properly accounted for, the estimate of artisanal cobalt imports would be higher than it should be. Potential underestimation of artisanal cobalt processing (discussed above) and potential overestimation of artisanal cobalt imports may help to balance each other during the early 2000s when reporting was less reliable. Nonetheless, the estimates and trends generated from this method correspond well with government and industry estimates of artisanal production—as well as the results for artisanal-production-A. This may indicate that artisanal-production-B is sound but should not be viewed with the same confidence as artisanal-production-A .

Supporting Discussion 2: Estimates of the number of child miners (child labor) involved in artisanal cobalt production in the Democratic Republic of the Congo (DRC) over time

Estimating the number of adults and children who are digging for ore, tunneling for ore, washing ore, sorting ore, transporting ore, and otherwise working at cobalt mine sites is complicated by many factors (17–23). Estimates of the number of child miners are extrapolations from interviews as study sites, the sample size of which range from 100 child miners at a few sites (24) to several hundred sites (17, 22). In each of these studies, sample sites could not be chosen randomly to represent the whole population of sites. For example, researchers' access to artisanal mine sites is often blocked by federal, provincial, local, or private security forces, as well as by the miners themselves for unknown reasons (17–23). It is difficult to tell whether the presence of security forces indicates higher levels of organization that reduce the participation of

child miners or if the denial of access indicates that larger numbers of child miners (and other potential abuses) are present. Such nuances in small samples of site visits will generate wide variances in broader extrapolations.

Another problem related to selection of sample sites is that children have been reported to be concentrated in a relatively small share of artisanal cobalt mine sites (22, 25). While child laborers may make up large shares of the artisanal miners at some sites, they are not present in all sites (17, 22). Further, there is often no separation between living and mining spaces at cobalt mine sites. This means that many children are present but not necessarily laboring. As a result, there are many activities that children participate in at artisanal cobalt mine sites including children mostly between 15 and 18 who are tunneling, digging, or transporting ore via bicycle (17–23) and children who are mostly between 8 and 14 who are washing or sorting ore (17–23). Other activities that may not fall into the category of labor are children who are being supervised by relations that are mining, children who are supervising their younger siblings, or children who are running menial errands for artisanal miners (18, 22, 25, 26).

Seasonality is another problem. Authors making estimates of the number of total and child miners rarely specify if their estimate is for the peak (dry) mining season or for the off (wet) season. For example, a study that does clarify this important variable estimates 67,000 to 79,000 permanent (wet or dry) total miners and 90,000 to 108,000 total miners during the peak (dry) season (19). For the low estimate, the dry season estimate is 34% higher than the wet season. For the high estimate, it is 37% higher.

Further, estimates of the number of artisanal cobalt miners (and related children) are often confused with larger numbers of artisanal miners producing all commodities in the former Katanga province including gold mined near the cobalt producing copper-belt, as well as tin, tungsten, tantalum (i.e., coltan), and gold farther to the north where cobalt is not mined. (Although the name ‘coltan’ gives the impression that it contains cobalt, due to the ‘co,’ it actually refers to a niobium—formerly called columbium—and tantalum containing mineral that is artisanally produced, largely for its tantalum content.) Unlike tin, tungsten, tantalum, and gold, cobalt is not a conflict mineral (27) and—despite the presence of security forces—recent studies with larger sample sizes (i.e., >100 sites) found no signs of forced labor (17, 19, 22, 25).

Additionally, proper citation is a common problem in this literature. Rather than ‘citing through’ to the primary study that generated the original estimate, authors often reference a later study that cites the original study (19, 23–25, 28–30). Presumably this is because, like this author, the studies’ authors were not able to find the primary studies. Further, for several of the studies within this literature, this author was not able to locate a reference or bibliography section (21, 31, 32). These issues have led to considerable confusion regarding estimates of the number of children present at artisanal cobalt mine sites. For example, when studies in this literature (and more popular forms of media such as newspaper and magazine articles) attempt to report the number of children mining cobalt artisanally, they may in fact be reporting an estimate of the number of children: present at all artisanal mine sites in the DRC, working at all artisanal mine sites in the DRC, present at all artisanal mine sites in the former Katanga province, working at all artisanal mine sites in the former Katanga province, present at artisanal cobalt mine sites, or working at artisanal cobalt mine sites.

Given the large number of difficulties in estimating the number of total and child artisanal cobalt miners, the goal of this section is not to identify the ‘real’ number of child artisanal cobalt miners. Rather the goal is to convey the reported estimate ranges and discuss how they may be changing over time. With these difficulties laid out, the following is a discussion of the studies that comprise the literature on child artisanal cobalt miners. These studies are discussed by year in the bulleted section below. The final bullet for a given year explains how the averaged estimates in Figure 2a were calculated.

- 1990s
    - In the 1990s, artisanal mining in the DRC is reported by some to have been relatively small (21). This seems unlikely for three reasons. First, the economic

collapse and hyper-inflation of the early 1990s in the DRC left much of the population scrambling for any method of income and subsistence (33). Second, the industrial cobalt mining industry in the DRC collapsed during the 1990s, falling from 66% of world production in 1986 to 5% in 1994. Given that this caused world production to fall from 50,000 t in 1986 to 18,000 t in 1994 (34), it is unlikely that artisanal miners would not produce cobalt in response to price increases from the supply shortfall. Third, there were two Great African Wars over the DRC and its resources in the late 1990s and early 2000s. During this period warring nations illegally exploited minerals in the DRC—much of which was extracted via artisanal mining (35–37).

  - No estimates presented in Figure 2a.

- 1999
  - The International Labor Organization (in collaboration with the World Bank and United Nations agencies) estimated that there were 150,000 artisanal miners (for all commodities) in the entire DRC (38). In this report, the DRC appears to only have been mentioned once and cobalt was not mentioned at all (38). Similarly, in the 2001 and 2002 UN reports of the panel of experts on the illegal exploitation of natural resources in the DRC (35–37), only gold and diamonds are mentioned as minerals produced by artisanal miners (36). Cobalt is only discussed in relation to large-scale mining (35–37).
  - No estimates were found for the number of artisanal or child cobalt miners so no estimate in Figure 2a.
- 2002
  - A 2004 Global Witness study on mineral trade in Southern Katanga estimated that the three largest cobalt ore trading houses[1] employed 5,000 total artisanal cobalt miners and that there were roughly 10,000 total artisanal cobalt miners at one site alone (Shinkolobwe) (39). This study also cited a 2004 ASADHO study on illegal resource exploitation (40)—which could not be found—stating there were roughly 60,000 young men and boys working as artisanal cobalt miners in the copper-cobalt belt of Katanga around the year 2002 (39). This number appears to be an estimate of all artisanal cobalt miners.
  - A 2007 CRONGD case study (on the human rights impacts of cobalt ore processing facility investments in the DRC by the Chinese firm SOMIKA (31)) refers to an uncited 2004 Nouvelle Dynamique Syndicale study that estimates nearly 60,000 artisanal cobalt miners in Katanga (the same estimate as the 2004 ASADHO study), 40% of which are children under 18 years of age (31). This provides a clear 2002 estimate of 24,000 child artisanal cobalt miners. It seems likely that the 2004 ASADHO and Nouvelle Dynamique Syndicale studies may be one in the same
    - This is close to a different estimate of 20,000 child miners for all commodities in all of the former Katanga province (19, 41). This may indicate that child miners in Katanga were almost entirely mining cobalt. It could also be due to the difficulty of making such estimates in the early 2000s.
  - As a result, Figure 2a displays 60,000 artisanal cobalt miners, 24,000 of whom are children, or 33%.
- The 1999 and 2002 estimates imply that in the early 2000s, of total miners in the DRC, 40% were mining cobalt, with 40% of artisanal cobalt miners being children.
- 2003
  - A 2011 Oeko-Institut study on socio-economic consequences of artisanal cobalt mining (19) cites a 2005 university study on child miners in artisanal mines in Katanga (41)—which also could not be found by this author—that references an unspecified 2003 UNICEF report stating there were 20,000 child miners in

[1] Bazano, Chemaf, and Societe Minere Du Katanga – also known as SOMIKA

Katanga in the early-2000s. Given that the 2011 Oeko-Institut study focuses on artisanal cobalt mining, this estimate is assumed to be for cobalt and is presented in Figure 2a.

- 2005
  - The 2011 Oeko-Institut study also cites a 2007 Groupe One study on child miners in Katanga (42)—which could not be found—estimating that out of 41,500 total miners in Lubumbashi only (a cobalt producing region of Katanga), 22,500 (54%) were children. This represents a minimum estimate for the mid-2000s because it is only for one portion of the larger cobalt producing region in southern Katanga.
  - Figure 2a shows only the estimate of 54% child miners.
- 2006
  - The 2011 Oeko-Institut study also cites a 2007 study by Group One where estimates there were 120,000 total miners in the copper-cobalt belt, 54,000 (42%) of which are children (19).
  - Figure 2a shows the numbers above.
- 2007
  - For the year 2007 a World Bank report aggregated previous studies. Given the range of estimates from 50,000 to 250,000 artisanal cobalt miners, it estimated 150,000 total with 40% being children (18).
    - The range of estimates appear to come from other studies but there are no citations to indicate which.
    - It is not clear if the 40% estimate is a recycled estimate from the 2004 Nouvelle Dynamique Syndicale study referenced by the 2007 CRONGD case study, or if this is a new estimate that is the same as the old one.
  - An uncited 2007 UNICEF briefing note is referenced in a 2009 academic study of artisanal mining in Katanga (24) which is stated to estimate 20,000 artisanal child miners in Katanga. This appears to be the same UNICEF estimate of 20,000 that was also uncited in the 2005 university study on child miners (41).
  - Figure 2a shows the World Bank's average of 50,000 to 250,000 total miners (150,000), where child miners equal this mean, times the stated percentage of 40% (60,000).
- 2008
  - A 2009 RAID study of Chinese mining operations in Katanga estimated there were one million total miners (all commodities) in Katanga as of August 2008 (20). The 2009 RAID study then estimates that, out of the one million total miners in Katanga, only 20,000 (2%) of them were children (20). While it is possible that this is a typo, 20,000 child artisanal miners in Katanga is the same as that estimated in the uncited 2003 and 2007 UNICEF references above.
  - A 2017 Berkeley study of site visits to artisanal mining communities (with no publicly available bibliography) referenced an uncited 2009 study (43) that estimated 13% (between 28,000 and 45,000) of total artisanal cobalt miners were children (21).
    - Nine-tenths of these are estimated to work at the surface.
    - While no estimate of a total number of artisanal cobalt miners was presented, the figures above suggest a range from 215,000 to 346,000. This range is at the top end of, and exceeding, the 2008 World Bank estimated range of 50,000 to 250,000 artisanal cobalt miners for 2007.
    - Assuming the 215,000 to 346,000 range are accurate, and that the 2009 RAID estimate of one million total miners for all commodities is accurate, that would mean that artisanal cobalt miners make up between 20% and 30% of total artisanal miners in Katanga. The other artisanal miners would presumably be mining gold (throughout Katanga) or tin, tungsten, and tantalum in northern Katanga.
  - A 2010 PACT study of three artisanal cobalt mines in and near Kolwezi estimated 30,000 artisanal cobalt miners and 4,000 children either directly or

indirectly affected by the mining for these three sites for the year 2008 (28). This study goes on to say that some of the mine sites visited had up to 40% child miners, which is the same as the 2004 ASAHDO and Nouvelle Dynamique Syndicale studies, as well as the 2008 World Bank study. The 2010 PACT study later states that, "reports on specific sites suggest that 20% of diggers in Kolwezi are children" (44).
    - The estimate presented in Figure 2a is calculated as follows: total number of miners is the average of 220,000 and 330,000 (275,000). The number of child miners is the average of 20,000 from one study and the mean of 30,000 and 50,000 estimated by another (30,000). The percentage is presented as the reported 13%.
- From the early 2000s to the mid-to-late-2000s the estimates indicate changes in: the number of artisanal cobalt miners from 60,000 to a mean of 150,000; the number of child miners from 25,000 to a mean of 50,000; and the percentage comprised of child miners from 40% to 35%. These means do not include 20,000 child miners in 2008. Based on the estimate of one million total miners for all of the former Katanga province and the mean estimates for 2007 and 2008, 20% of artisanal miners were mining cobalt—down from 40% in early 2000s. (The rest would most likely be mining tin, tungsten, tantalum, and gold in the north of the former Katanga province.)
- 2010
    - The 2011 Oeko-Institut study estimates, between 67,000 and 79,000 permanent full-time miners and 90,000 to 108,000 during the peak (dry) season (19). Twenty-eight percent or 20,000 to 30,000 were estimated to be children.
    - Figure 2a presents the average of the range 20,000 to 35,000 child miners (28,000) with the reported percentage of 28% and the resultant estimate of 100,000 total miners.
- 2012
    - A 2012 UNICEF article on getting child miners back into school refers to an uncited estimate that of 120,000 total miners in the copper-cobalt belt (same as the 2011 Oeko-Institut study), 40,000 (33%) were children (28).
    - Similarly, a 2013 World Vision study indicated that the only information they could gather on the percentage of miners in Kambove (one area of the copper-cobalt belt) was from a single interview estimating that of total artisanal cobalt miners, 33% were children (32).
    - Figure 2a shows 120,000 total, 40,000 children, and 33%.
- 2014
    - As of 2020, an often recycled estimate of 40,000 child cobalt miners comes from an uncited December 2014 UNICEF factsheet that this author was not able to find (45). Here, this is assumed to be the 2012 UNICEF estimate of 40,000 child miners in the former Katanga province above (45).
- 2016
    - A Berkeley report estimates 5,000 children working at cobalt mine sites from their cite visits, which serves as a minimum estimate for 2016 (21)
- 2017
    - A 2018 article in the Guardian, based on 31 artisanal mining sites in the south-east of the DRC, estimates that of 255,000 total cobalt miners, 35,000 (14%) were children in the year 2017 (46).
- 2018
    - A 2018 European policy brief cites an estimate that of total artisanal cobalt miners, 12% are children (47). This author was not able to find this reference.
    - Figure 2a presents the average of the range from 150,000 to 200,000 (175,000) total miners, and 17,000 child miners based on the reported percentage of 12%.
- 2019
    - A 2019 private/NGO report estimates that there are well over 200,000 total artisanal cobalt miners (48)

    - A BGR study mapping the artisanal cobalt supply chain found children working in 11 (19%) of the 59 sites visited (22). Based on the study's cite visits, it was estimated that there were at least 31,000 artisanal miners and at least 3,000 children working at cobalt mine sites.
    - A 2019 OECD study cited an unnamed study estimating children were present or working at 25% of artisanal cobalt mine sites studied (25).
    - A 2020 PACT briefing estimated total miners to be between 60,000 and 80,000 (49).
    - Figure 2a presents the average of 70,000 total miners and 13,000 child miners as the stated percentage of 19% of the total.
- 2020
    - A 2021 BGR study on artisanal cobalt mining conditions and trading networks found children working at 26% of sites visited (17).
    - Figure 2a presents the 26% above.

Any use of trade, firm, or product names is for descriptive purposes only and does not imply endorsement by the U.S. Government.

SI References

1. IHS Markit, Global Trade Atlas (2022).
2. A. Young, Gold into base metals: Productivity growth in the People's Republic of China during the reform period. *NBER Work. Pap. Ser.* (2000).
3. G. Chow, Are Chinese official statistics reliable ? *CESifo Econ. Stud.* **52** (2006).
4. L. R. Klein, S. Özmucur, The estimation of China's economic growth rate. *J. Econ. Soc. Meas.* **28** (2011).
5. X. Xu, Understanding China's official statistics. *China Econ. J.* **6** (2013).
6. J. N. Koch-weser, "The reliability of China ' s economic data : An analysis of national output" (2013).
7. J. Fernald, I. Malkin, M. Spiegel, "Economic Letter: On the reliability of Chinese output figures" (2013).
8. D. Plekhanov, Quality of China's official statistics : A brief review of academic perspectives. *Copenhagen J. Asian Stud.* **35**, 76–101 (2017).
9. L. Beijing Antaike Information Co., Precious and minor metals monthly: 2009 China Cobalt Trade Policy (2009).
10. A. Xu, "Minor metals monthly December 2012" (2012).
11. A. Xu, Minor metals monthly December 2013 (2013).
12. A. Xu, Minor metals monthly January 2015 (2015).
13. A. Xu, Minor metals monthly January 2016. 1–19 (2016).
14. A. Xu, Minor metals monthly January 2018 (2018).
15. A. Xu, Minor metals monthly January 2019 (2019).
16. A. Xu, *et al.*, "Lithium-ion battery metal materials monthly February 2021" (2021).
17. BGR, "Mining Conditions and Trading Networks in Artisanal Copper-Cobalt Supply Chains in the Democratic Republic of the Congo" (2021).
18. World Bank, "Democratic Republic of Congo: Growth with governance in the mining sector" (2008).
19. N. Tsurukawa, S. Prakash, A. Manhart, Social impacts of artisanal cobalt mining in Katanga, Democratic Republic of Congo. *Öko-Institut eV - Inst. Appl. Ecol. Freibg.* **49**, 65 (2011).
20. S. Goethals, J.-P. Okenda, R. Mbaya, "Chinese mining operations in Katanga" (2009).
21. B. Faber, B. Krause, R. S. De La Sierra, "Artisanal mining, livelihoods, and child labor in the cobalt supply chain of the Democratic Republic of Congo" (2017).
22. BGR, Mapping of the artisanal copper-cobalt mining sector in the provinces of Haut-Katanga and Lualaba in the Democratic Republic of the Congo (2019).

23. Amnesty International, “This is what we die for: Human rights abuses in the Democratic Republic of the Congo power the global trade in cobalt” (2016).
24. J. K. Musao, “The problem of artisanal mining in the province of Katanga (case of the district of Kolwezi) - in French,” Lubumbashi. (2009).
25. L. Maiotti, B. Katz, T. Gillard, H. Koep-Andrieu, “Interconnected supply chains: a comprehensive look at due diligence challenges and opportunities sourcing cobalt and copper from the Democratic Republic of the Congo” (2019).
26. Amnesty International, “Profits and loss: Mining and human rights in Katanga, Democratic Republic of the Congo” (2013).
27. SEC, “Disclosing the use of conflict minerals” (2017).
28. C. Walther, “In DR Congo , UNICEF supports efforts to help child labourers return to school” (2012).
29. P. Dolega, D. Schüler, “European policy brief: China’s approach towards responsible sourcing” (2018).
30. World Bank, “2020 State of the artisanal and small-scale mining sector” (2020).
31. CRONGD, “Democratic Republic of the Congo toxic cocktail: Protecting human rights amidst administrative confusion” (Rights and Democracy, 2007).
32. World Vision, “Child miners speak: Key findings on children and artisanal mining in Kambove, DRC” (2013).
33. G. Nzongola-Ntalaja, *The Congo from Leopold to Kabila: A People’s History* (Bloomsbury Publishing, 2002).
34. A. L. Gulley, One hundred years of cobalt production in the Democratic Republic of the Congo. *Resour. Policy* **79**, 103007 (2022).
35. UN Security Council, April 2001 report of the panel of experts on the illegal exploitation of natural resources and other forms of wealth of the Democratic Republic of the Congo. *UN Doc. S/2001/357 (Apr. 12, 2001)* (2001).
36. UN Security Council, “November 2001 Addendum to the report of the panel of experts on the illegal exploitation of natural resources and other forms of wealth of the Democratic Republic of the Congo” (2001).
37. UN Security Council, “October 2002 report of the panel of experts on the illegal exploitation of natural resources and other forms of wealth of the Democratic Republic of the Congo” (2002).
38. ILO, “Social and labour issues in small-scale mines: Report for discussion at the Tripartite Meeting on social and labour issues in small-scale mines” (1999).
39. Global Witness, “Rush and ruin: The devastating mineral trade in Southern Katanga, DRC” (2004).
40. ASADHO, “Katanga, Rapport préliminaire sur l’exploitation illégale des ressources naturelles en RD Congo” (2004).
41. E. Tshilobo, “Des milliers d’enfants exploités dans les mines du Katanga” (2005).
42. Groupe One, “Problématique du travail des enfants dans les mines artisanales au Katanga.” (2007).
43. Y. Vanbrabant, *et al.*, “TRACE (TRACeability of hEterogenite) Project Report.” (2009).
44. Pact, “PROMINES study - Artisanal mining in the Democratic Republic of Congo” (2010).
45. UNICEF, “Factsheet: enfants dans les mines” (2014).
46. S. Kara, Is your phone tainted by the misery of the 35,000 children in Congo’s mines? *Guard.* (2018).
47. Hanrui Cobalt, Industry to formally establish Responsible Cobalt Initiative, to take action on sustainable cobalt supply chain (2017).
48. S. J. De Silva, T. Strauss, N. Morisho, “The Mutoshi Pilot Project: Local economic impact of a project aimed at formalizing artisanal and small-scale mining” (2019).
49. PACT, “Confronting modern slavery in DRC-UK mineral supply chains” (2020).

Dataset S1: Price of refined cobalt (2020$/lb), artisanal cobalt mine production estimates from the literature (metric tons, cobalt content), total world cobalt mine production (metric tons, cobalt content), and total Democratic Republic of the Congo (DRC) cobalt mine production (metric tons, cobalt content) from 2000 through 202C

	Reporter Type	2000	2001	2002	2003	2004	2005	2006	2007	2008	2009	2010	2011	2012	2013	2014	2015	2016	2017	2018	2019	2020	References	Notes
Cobalt price (2020$/lb, rounded	Industry	$ 22	$ 15	$ 10	$ 15	$ 32	$ 20	$ 20	$ 34	$ 40	$ 20	$ 22	$ 17	$ 14	$ 12	$ 14	$ 14	$ 13	$ 27	$ 36	$ 16	$ 16	A. L. Gulley, One hundred years of cobalt production in the Democratic Republic of the Congo. Resour. Policy 79, 103007 (2022)	
USGS MYB, artisanal production	Government	1,000	2,000	2,000	4,000	7,000	7,000	-	-	-	-	-	-	-	-	-	-	-	-	-	-	-	2000-2003 K. B. Shedd, "Cobalt" in 2003 Minerals Yearbook, (U.S. Geological Survey, 2006); 2004-2005 K. B. Shedd, "Cobalt" in 2005 Minerals Yearbook, (US Geological Survey, 2008)	
Vanbrabant, artisanal productio	Non-governmental organization (NGO)	8,000	9,000	11,000	11,000	15,000	18,000	20,000	19,000	24,000	-	-	-	-	-	-	-	-	-	-	-	-	Y. Vanbrabant, et al., "TRACE (TRACeability of hEterogenite) Project Report." (2009).	
Darton, artisanal production	Industry	-	-	-	-	-	-	-	-	-	-	-	-	-	4,000	4,000	11,000	9,000	11,000	18,000	8,000	7,000	Darton, "Cobalt Market Review: 2014-2015" (2015).; Darton, "Cobalt Market Review: 2015-2016" (2016).; Darton, "Cobalt Market Review: 2016-2017" (2017).; Darton, "Cobalt Market Review: 2017-2018" (2018).; Darton, "Cobalt Market Review: 2018-2019" (2019).; Darton, "Cobalt Market Review: 2019-2020" (2020).; Darton, "Cobalt Market Review: 2020-2021" (202	
Global Witness, artisanal produc	Non-governmental organization (NGO)	-	-	-	-	22,500	-	-	-	-	-	-	-	-	-	-	-	-	-	-	-	-	Global Witness, "Rush and ruin: The devastating mineral trade in Southern Katanga, DRC" (2004).	60,000 t (of exported heterogenite) * 7.5% (average Co content in this report) * 5 (months in dry season) = 22,500 t Co content "A Katangan mining expert... calculated... 60,000 t... heterogenit... during dry season (~May - October)
World Bank, artisanal productio	Government	-	-	-	-	-	-	8,000	-	-	-	-	-	-	-	-	-	-	-	-	-	-	World Bank, "Democratic Republic of Congo: Growth with governance in the mining sector" (2008).	138,773*.8*.075 = 8,000 (nearest thousand) "The artisans... produce... 80 percent of the... 138,773 metric t.... officially exported from Katanga in 2006.
DERA/BGR, artisanal production	Government	-	-	-	-	-	-	-	-	-	7,000	8,000	8,000	7,000	7,000	8,000	10,000	10,000	12,000	18,000	13,000	-	S. Al Barazi, et al., Cobalt from the DRC – Potential, Risks and Significance for the Global Cobalt Market. DERA Commod. TopNews 53, 1–15 (2017).; BGR, Mapping of the Artisanal Copper-Cobalt Mining Sector in the Provinces of Haut-Katanga and Lualaba in the Democratic Republic of the Congo (2019	
CRU, artisanal production	Industry	-	-	-	-	-	-	-	-	-	11,000	18,000	17,000	8,000	6,000	9,000	10,000	6,000	19,000	30,000	12,000	10,000	G. Heppel, "Artisanal mining will balance the cobalt market," CRU (2018).	
Maiotti, artisanal production	Non-governmental organization (NGO)	-	-	-	-	-	-	-	-	-	-	-	-	-	-	-	-	-	19,000	25,000	26,000	-	L. Maiotti, B. Katz, T. Gillard, H. Koep-Andrieu, "Interconnected supply chains: a comprehensive look at due diligence challenges and opportunities sourcing	Average of 18% and 30% times DRC production for each year. "The percentage of artisanal production of cobalt ranges between 18% and 30% (of production).
PACT, artisanal production	Non-governmental organization (NGO)	-	-	-	-	-	-	-	-	-	-	-	-	-	-	-	-	-	-	-	-	29,000	PACT, "Confronting modern slavery in DRC-UK mineral supply chains" (2020).	
Save the Children, artisanal prod	Non-governmental organization (NGO)	-	-	-	-	-	-	-	-	-	-	-	-	-	-	-	-	-	-	-	-	33,000	C. Gaffar, I. Kaempfer, "Opportunities for Businesses to Promote Child Rights in Cobalt Artisanal and Small-Scale Mining" (2021).	
Total DRC mine production (rour	Government	11,000	12,000	14,600	14,800	20,200	24,500	27,100	25,400	32,300	40,000	60,000	59,000	52,000	56,000	65,000	72,000	68,000	80,000	104,000	107,000	98,200	K. B. Shedd, "Cobalt" in 2003 through 2019 Minerals Yearbooks, (U.S. Geological Survey, 2006 through 2021). https://www.usgs.gov/centers/national-minerals-information-center/cobalt-statistics-and-informatior	
Total world mine production (ro	Government	37,900	44,800	52,200	52,900	60,300	65,200	70,000	68,500	74,200	75,900	105,100	105,700	99,700	102,700	115,200	121,700	115,300	127,800	149,100	154,800	142,900	K. B. Shedd, "Cobalt" in 2003 through 2019 Minerals Yearbooks, (U.S. Geological Survey, 2006 through 2021). https://www.usgs.gov/centers/national-minerals-information-center/cobalt-statistics-and-informatior	
Government reported estimates	Government	1,000	2,000	2,000	4,000	7,000	7,000	8,000	N/A	N/A	7,000	8,000	8,000	7,000	7,000	8,000	10,000	10,000	12,000	18,000	13,000	N/A		Government reported estimates
Industry average reported estim	Industry	N/A	N/A	N/A	N/A	N/A	N/A	N/A	N/A	N/A	11,000	18,000	17,000	8,000	5,000	7,000	11,000	8,000	15,000	24,000	10,000	9,000		Average of industry reported estimates
NGO average reported estimates	Non-governmental organization (NGO)	8,000	9,000	11,000	11,000	19,000	18,000	20,000	19,000	24,000	N/A	N/A	N/A	N/A	N/A	N/A	N/A	N/A	19,000	25,000	26,000	31,000		Average of non-governmental organization reported estimates

Dataset S2: Democratic Republic of the Congo (DRC) industrial mine production 2000 through 2020 (metric tons, cobalt content)

Reported feed sources*	Contemporary mine/processing facility operator	Contemporary mine/processing facility name	Contemporary majority owner	Owner's headquarters	2000	2001	2002	2003	2004	2005	2006	2007	2008	2009	2010	2011	2012	2013	2014	2015	2016	2017	2018	2019	2020	References	Notes
Own mines	Glencore plc	Mutanda Mining	Glencore plc	Switzerland	-	-	-	-	-	-	-	-	-	7,900	8,900	7,800	8,400	13,700	14,500	16,500	24,600	23,900	27,300	25,100	-	Darton Commodities Ltd. Annual Cobalt Market Review	-
Own mines	Glencore plc	Kamoto / Katanga Mining / KCC (Kamoto Copper Company)	Glencore plc	Switzerland	-	-	-	-	-	-	400	1,000	1,200	2,500	3,400	2,600	2,100	2,600	2,200	2,900	-	-	11,100	17,100	23,900	2006-2007 USGS Cobalt MYB, 2008-2020 Darton Commodities Ltd. Annual Cobalt Market Review	-
Own mines	Eurasian Resources Group	RTR metalkol (aka: Kolwezi Cu/Co tailings project)	Eurasian Resources Group	Kazakhstan	-	-	-	-	-	-	-	-	-	-	-	-	-	-	-	-	-	-	-	8,400	15,500	Darton Commodities Ltd. Annual Cobalt Market Review	-
Own mines	China Molybdenum Co Ltd. (CMOC)	Tenke Fungurume	China Molybdenum Co Ltd. (CMOC)	China	-	-	-	-	-	-	-	-	-	2,600	8,700	11,300	11,300	12,100	13,500	15,200	15,800	16,800	18,800	16,100	15,400	2001-2011 S&P Global Metals & Mining mineral asset database, 2012-2020 Darton Commodities Ltd. Annual Cobalt Market Review	-
Own mines	Chemaf SARL	Etoile / Usoke	Shalina Resources	Dubai	-	-	-	-	-	-	2,000	2,600	2,400	2,800	2,700	2,400	2,200	2,200	1,500	2,300	1,900	3,700	7,200	8,100	6,200	2004-2008 USGS Cobalt MYB, 2009-2020 Darton Commodities Ltd. Annual Cobalt Market Review	-
Own mines	Ruashi Mining	Ruashi	Jinchuan Group International Resource Co. Ltd.	China	-	-	-	-	-	-	100	400	600	3,100	3,600	3,800	3,600	3,200	3,400	4,300	3,400	4,600	4,300	5,100	4,200	Darton Commodities Ltd. Annual Cobalt Market Review	-
Own mines	China Nonferrous Metals Corp. (CNMC)	Deziwa/Somidez	China Nonferrous Metals Corp. (CNMC)	China	-	-	-	-	-	-	-	-	-	-	-	-	-	-	-	-	-	-	-	-	4,200	Darton Commodities Ltd. Annual Cobalt Market Review	-
Own mines	La Générale des Carrières et des Mines (Gécamines)	Big Hill	Gécamines	DRC	1,500	1,800	1,900	3,100	2,900	2,300	4,300	5,500	5,500	4,600	4,200	4,500	3,200	4,300	5,200	4,200	3,600	2,600	-	-	3,600	2003-2007 Roskill Cobalt 11th edition (2007), 2008-2020 Darton Commodities Ltd. Annual Cobalt Market Review	-
Own mines	Compagnie Miniere de Kambove (COMIKA)	Kamoya	Wanbao Mining Limited	China	-	-	-	-	-	-	-	-	-	-	-	-	-	-	-	-	500	3,200	3,200	3,200	2,900	Darton Commodities Ltd. Annual Cobalt Market Review	-
Own mines	China Railways Resources Group	MKM Cu & Co mines	China Railways Resources Group	China	-	-	-	-	-	-	-	-	-	-	-	-	-	-	-	100	600	1,300	1,100	2,000	1,900	Darton Commodities Ltd. Annual Cobalt Market Review	-
Own mines	Zheijiang Huayou Cobalt	MIKAS (La Miniere de Kasombo)	Zheijiang Huayou Cobalt	China	-	-	-	-	-	-	-	-	-	-	-	-	-	-	-	-	-	-	2,800	2,100	1,600	Darton Commodities Ltd. Annual Cobalt Market Review	-
Own mines	Congo Dongfeng International Mining SPRL (CDM)	Luiswishi	Huayou Cobalt. Co. Ltd.	China	4,000	4,000	4,000	2,500	3,800	5,000	5,000	4,000	3,500	400	2,500	500	1,300	1,800	2,400	1,400	1,500	1,500	1,500	1,500	1,500	S&P Global Metals & Mining mineral asset database	From 2016-2020, Luiswishi's mine production (previously owned by Compagnie Miniere du Sud Katanga or CMSK) is reported together with CDM' Lubumbashi plant. Given the average from 2009-2015, Luiswishi's mine production is assumed to be 1,500 tons per annum. This is likely an overestimate, given that mine proc
Own mines	Pengxin International Mining	Shituru processing plant	Pengxin Group Co., Ltd.	China	-	-	-	-	-	-	-	-	-	-	-	-	-	-	-	-	-	-	-	-	1,100	Darton Commodities Ltd. Annual Cobalt Market Review	-
Own mines	Zijin Mining Group Co Ltd.	Compagnie Miniere de Musonoie Global SAS (COMMUS)	Zijin Mining Group	China	-	-	-	-	-	-	-	-	-	-	-	-	-	-	-	-	-	-	-	-	1,000	Darton Commodities Ltd. Annual Cobalt Market Review	-
Own mines	China Railways Resources Group	Sicomines and Luishia	China Railways Resources Group	China	-	-	-	-	-	-	-	-	-	-	-	-	-	-	-	-	800	900	500	700	900	2000-2008 S&P Global Metals & Mining mineral asset database, 2009-2020 Darton Commodities Ltd. Annual Cobalt Market Review	-
Own mines	Aurum Kisanfu Mining	KIMIN	Vinmart Group	China	-	-	-	-	-	-	-	-	-	-	-	-	-	-	-	-	-	-	-	-	500	Darton Commodities Ltd. Annual Cobalt Market Review	-
Own mines	China Nonferrous Metals Corp. (CNMC)	Huachin Metals Leach	China Nonferrous Metals Corp. (CNMC)	China	-	-	-	-	-	-	-	-	-	-	-	-	-	-	-	-	-	-	-	-	200	Darton Commodities Ltd. Annual Cobalt Market Review	-
Own mines	La Compagnie Minière de Luisha Sprl (COMILU)	Luisha mine	China Railway Engineering Corporation Group (CRECG)	China	-	-	-	-	-	-	-	-	-	-	-	-	-	-	-	-	-	-	-	-	200	Darton Commodities Ltd. Annual Cobalt Market Review	-
Own mines	Eurasian Resources Group	Boss Mining (Kababankola mine, Mukondo Mountain mine)	Eurasian Resources Group	Kazakhstan	-	-	1,000	3,000	4,000	5,000	5,000	1,000	5,000	4,600	7,800	10,000	8,300	9,100	8,700	6,300	4,500	3,300	3,300	200	-	2002-2008 S&P Global Metals & Mining mineral asset database, 2009-2020 Darton Commodities Ltd. Annual Cobalt Market Review	2002-2004 production is from Kababankola Mining Co. (KMC) SPRL; 2005-2006 production is the sum of KMC, Central African Mining & Exploration Company plc (CAMEC), and Boss Mining; Eurasian Resources Group later consolidated all of these mining operations under the name 'Boss Mining
Own mines	CuCo Resources Ltd	Kisanfu	CuCo Resources Ltd	Canada	-	-	-	-	-	-	-	-	-	-	1,500	-	-	-	-	-	-	-	-	-	-	2010 USGS Minerals Yearbook Congo (Kinshasa)	-
Own mines	La Générale des Carrières et des Mines (Gécamines)	Gécamines owned mines in Central and Western groups	Gécamines	DRC	3,400	3,200	2,100	1,200	700	600	600	600	300	400	800	700	900	500	100	200	100	100	100	-	-	2000-2008 USGS Minerals Yearbook Cobalt, 2009-2020 Darton Commodities Ltd. Annual Cobalt Market Review	Proxied by Gécamines refinery output.
Own mines	Metal Mines SARL	Metal Mines Katanga processing facility	Nanjing Hanrui Cobalt	China	-	-	-	-	-	-	-	-	-	-	-	-	-	-	1,100	1,700	2,000	2,900	5,900	4,200	4,300	Darton Commodities Ltd. Annual Cobalt Market Review	-
**Industrial DRC mine production (rounded to nearest 1,000)**					**9,000**	**9,000**	**9,000**	**10,000**	**11,000**	**13,000**	**17,000**	**15,000**	**19,000**	**29,000**	**44,000**	**44,000**	**41,000**	**50,000**	**53,000**	**55,000**	**59,000**	**65,000**	**87,000**	**94,000**	**89,000**		

Dataset S3: Artisanal-production-A results for nonindustrial (i.e., artisanal) cobalt mine production from 2000 through 2020 (metric tons, cobalt content, rounded to nearest thousand)

	2000	2001	2002	2003	2004	2005	2006	2007	2008	2009	2010	2011	2012	2013	2014	2015	2016	2017	2018	2019	2020	References	Notes
Total DRC cobalt mine production (reported)	11,000	12,000	14,600	14,800	20,200	24,500	27,100	25,400	32,300	40,000	60,000	59,000	52,000	56,000	65,000	72,000	68,000	80,000	104,000	107,000	98,200	2000-2019 K. B. Shedd, "Cobalt" in 2003 through 2019 Minerals Yearbooks, (U.S. Geological Survey, 2006 through 2021); 2020 K.B. Shedd, "Cobalt" in 2020	-
Total industrial mine production (see directly above)	9,000	9,000	9,000	10,000	11,000	13,000	17,000	15,000	19,000	29,000	44,000	44,000	41,000	50,000	53,000	55,000	59,000	65,000	87,000	94,000	89,000	-	-
**Artisanal-production-A, nonindustrial DRC cobalt mine production (results)**	**2,000**	**3,000**	**6,000**	**5,000**	**9,000**	**12,000**	**10,000**	**10,000**	**13,000**	**11,000**	**16,000**	**15,000**	**11,000**	**6,000**	**12,000**	**17,000**	**9,000**	**15,000**	**17,000**	**13,000**	**9,000**	-	**Artisanal-production-A results**
**Artisanal-production-A, nonindustrial DRC production's share of total DRC mine production (%, results)**	**18%**	**25%**	**41%**	**34%**	**45%**	**49%**	**37%**	**39%**	**40%**	**28%**	**27%**	**25%**	**21%**	**11%**	**18%**	**24%**	**13%**	**19%**	**16%**	**12%**	**9%**	-	
**Artisanal-production-A, nonindustrial DRC production's share of total world mine production (%, results)**	**5%**	**7%**	**11%**	**9%**	**15%**	**18%**	**14%**	**15%**	**18%**	**14%**	**15%**	**14%**	**11%**	**6%**	**10%**	**14%**	**8%**	**12%**	**11%**	**8%**	**6%**	-	

Dataset S4: Artisanal processing (i.e., first component of artisanal-production-B), Artisanal cobalt processing facilities, and artisanal sourcing from 2000 through 2020 (metric tons, cobalt content)

4a: Intermediate cobalt processing facility production in forms such as concentrate, crude carbonate, copper-cobalt alloys, and crude cobalt hydroxide

Reported feed sources*	Contemporary mine/processing facility operator	Contemporary mine/processing facility name	Contemporary majority owner	Owner's headquarters	2000	2001	2002	2003	2004	2005	2006	2007	2008	2009	2010	2011	2012	2013	2014	2015	2016	2017	2018	2019	2020	References	Notes
Artisanal and small-scale	Groupe Bazano	Unknown		Lebanon										2,000	1,800	1,200	600	200	1,000							Darton Commodities Ltd. Annual Cobalt Market Review	References report that Bazano purchased artisanal heterogenite from various sources.
Own mines and artisanal	Congo Dongfang Mining (CDM)	CDM Lubumbashi processing facility	Zheijiang Huayou Cobalt	China										900	1,100	1,500	1,900	1,600	5,300	5,400	6,700	6,300	8,100	8,200	4,400	2000-2012 S&P Global Metals & Mining mineral asset database, 2013-2022 Darton Commodities Ltd. Annual Cobalt Market Review	From 2014-2020 CDM reportedly sourced ~2,000 tpa of industrial mine feed from the Luiswishi operation, which the company reportedly purchased in 2014. Depending on the year, this indicates that between 41% and 82% was nonindustria
Own mines and artisanal	Societe Miniere Du Katanga (Somika SPRL)	Somika processing plant	Vinmart Group	India/China			500	500	500	1,000	1,000	1,000	2,500	2,500	2,500	2,000	2,000	2,100	1,500	2,200	200	2,300	3,500	1,400	200	2002-2008 USGS Minerals Yearbooks Congo (Kinshasa), 2009-2020 Darton Commodities Ltd. Annual Cobalt Market Review	2000-2020 SOMIKA is only reported to own a processing facility (no specific mines). It is also reported to be one of the three largest heterogenite trading houses in the DRC. SOMIKA was reported to be sourcing cobalt from artisanal miners as late as 2017. (Al Barazi et. al, 2017
Artisanal	Chengtun Mining	Kalongwe processing facility	Chengtun Mining	China																				800	3,100	Darton Commodities Ltd. Annual Cobalt Market Review	Chengtun reportedly "processes third party ore" and does not own a mine. It is reported that Chengtun will process material from "Jinchuan (Metorex's) Dilala West ore body in their Musonoi project," but there is no reported production for 2019 and Chengtun's production is 3 times that of Musonoi in 202
Artisanal	Jiana Energy (prev. Jiayuan Cobalt)	MJM processing facility		China																400	1,100	2,200	1,500			Darton Commodities Ltd. Annual Cobalt Market Review	Jiana is reported to process third party ore and no specific industrial sources are indicated
Artisanal	Feza Mining SPRL	Feza Likasi processing facility	Wanbao Mining Limited	China							500	500	500	500	500	500	500	500	500	500	500	-	-	-		Darton Commodities Ltd. Annual Cobalt Market Review	Feza is reported to own a copper/cobalt alloy processing facility, but no mines. There are no reports of industrial sources for the 500tpa of production, which the first reference indicates is fed by material from artisanal trading house:
Artisanal	Including (but not limited to) Congo Dongfang Mining (CDM), Groupe Bazano, Volcano Mining, and Bolfast													4,000	3,600	4,000	1,700	1,300	1,000	2,500	300	-	3,900	2,200	800	Darton Commodities Ltd. Annual Cobalt Market Review	References reporting this company's processing facility production do not indicate its feed is coming from industrial sources

4b: Intermediate cobalt processing facilities' reported percentage of feedstock sourced from artisanal mining

Reported feed sources*	Contemporary mine/processing facility operator	Contemporary mine/processing facility name	Contemporary majority owner	Owner's headquarters	2000	2001	2002	2003	2004	2005	2006	2007	2008	2009	2010	2011	2012	2013	2014	2015	2016	2017	2018	2019	2020	References	Notes
Artisanal and small-scale	Groupe Bazano	Unknown			0%	0%	0%	0%	0%	0%	0%	0%	0%	100%	100%	100%	100%	100%	100%	0%	0%	0%	0%	0%	0%	World Bank, "Democratic Republic of Congo: Growth with governance in the mining sector" (2008);	References report that Bazano purchased artisanal heterogenite from various sources.
Own mines and artisanal	Congo Dongfang Mining (CDM)	CDM Lubumbashi processing facility	Zheijiang Huayou Cobalt	China	0%	0%	0%	0%	0%	0%	0%	0%	0%	100%	100%	100%	100%	100%	41%	74%	78%	76%	81%	82%	66%	Darton Commodities Ltd. Annual Cobalt Market Review	From 2014-2020 CDM reportedly sourced ~2,000 tpa of industrial mine feed from the Luiswishi operation, which the company reportedly purchased in 2014. Depending on the year, this indicates that between 41% and 82% was nonindustria
Own mines and artisanal	Societe Miniere Du Katanga (Somika SPRL)	Somika processing plant	Vinmart Group	India/China	100%	100%	100%	100%	100%	100%	100%	100%	100%	100%	100%	100%	100%	100%	100%	100%	100%	100%	100%	100%	100%	2000-2004, Global Witness 2004 (Rush and ruin); 2005-2007, CRONGD 2007 (DRC toxic cocktail); 2008-2009, no change reported, 2010 (Darton 2010/2011)	2000-2020 SOMIKA is only reported to own a processing facility (no specific mines). It is also reported to be one of the three largest heterogenite trading houses in the DRC. SOMIKA was reported to be sourcing cobalt from artisanal miners as late as 2017. (Al Barazi et. al, 2017
Artisanal	Chengtun Mining	Kalongwe processing facility	Chengtun Mining	China	0%	0%	0%	0%	0%	0%	0%	0%	0%	0%	0%	0%	0%	0%	0%	0%	0%	0%	0%	100%	100%	Darton Commodities Ltd. Annual Cobalt Market Review	Chengtun reportedly "processes third party ore" and does not own a mine. It is reported that Chengtun will process material from "Jinchuan (Metorex's) Dilala West ore body in their Musonoi project," but there is no reported production for 2019 and Chengtun's production is 3 times that of Musonoi in 202
Artisanal	Jiana Energy (prev. Jiayuan Cobalt)	MJM processing facility		China	0%	0%	0%	0%	0%	0%	0%	0%	0%	0%	0%	0%	0%	0%	0%	100%	100%	100%	100%	0%	0%	Darton Commodities Ltd. Annual Cobalt Market Review	Jiana is reported to process third party ore and no specific industrial sources are indicated
Artisanal	Feza Mining SPRL	Feza Likasi processing facility	Wanbao Mining Limited	China	0%	0%	0%	0%	0%	0%	100%	100%	100%	100%	100%	100%	100%	100%	100%	100%	100%	0%	0%	0%	0%	S. Goethals, J.-P. Okenda, R. Mbaya, "Chinese mining operations in Katanga" (2009); Darton Commodities Ltd. Annual Cobalt Market Review	Feza is reported to own a copper/cobalt alloy processing facility, but no mines. There are no reports of industrial sources for the 500tpa of production, which the first reference indicates is fed by material from artisanal trading house:
Artisanal	Including (but not limited to) Congo Dongfang Mining (CDM), Groupe Bazano, Volcano Mining, and Bolfast				0%	0%	0%	0%	0%	0%	0%	0%	0%	100%	100%	100%	100%	100%	100%	100%	100%	0%	100%	100%	100%	Darton Commodities Ltd. Annual Cobalt Market Review	References reporting this company's processing facility production do not indicate its feed is coming from industrial sources

4c: Artisanal processing (i.e., first component of artisanal-production-B)

Reported feed sources*	Contemporary mine/processing facility operator	Contemporary mine/processing facility name	Contemporary majority owner	Owner's	2000	2001	2002	2003	2004	2005	2006	2007	2008	2009	2010	2011	2012	2013	2014	2015	2016	2017	2018	2019	2020	References	Notes
Own mines and artisanal	Groupe Bazano	Unknown												2,000	1,800	1,200	600	200	1,000							Darton Commodities Ltd. Annual Cobalt Market Review	-
Own mines and artisanal	Congo Dongfang Mining (CDM)	CDM Lubumbashi processing facility	Zheijiang Huayou Cobalt	China										900	1,100	1,500	1,900	1,600	2,200	4,000	5,200	4,800	6,600	6,700	2,900	2000-2012 S&P Global Metals & Mining mineral asset database, 2013-2022 Darton Commodities Ltd. Annual Cobalt Market Review	For years where the artisanal feed share is less than 100%, the processing production quantity only represents the portion of production from artisanal feed sources (i.e., not total production for that facility for that year).
Own mines and artisanal	Societe Miniere Du Katanga (Somika SPRL)	Somika processing plant	Vinmart Group	India/China			500	500	500	1,000	1,000	1,000	2,500	2,500	2,500	2,000	2,000	2,100	1,500	2,200	200	2,300	3,500	1,400	200	2002-2008 USGS Minerals Yearbooks Congo (Kinshasa), 2009-2020 Darton Commodities Ltd. Annual Cobalt Market Review	
Artisanal	Chengtun Mining	Kalongwe processing facility	Chengtun Mining	China																				800	3,100	Darton Commodities Ltd. Annual Cobalt Market Review	-
Artisanal	Jiana Energy (prev. Jiayuan Cobalt)	MJM processing facility		China																400	1,100	2,200	1,500			Darton Commodities Ltd. Annual Cobalt Market Review	-
Artisanal	Feza Mining SPRL	Feza Likasi processing facility	Wanbao Mining Limited	China							500	500	500	500	500	500	500	500	500	500	500	-	-	-		Darton Commodities Ltd. Annual Cobalt Market Review	-
Artisanal	Including (but not limited to) Congo Dongfang Mining (CDM), Groupe Bazano, Volcano Mining, and Bolfast													4,000	3,600	4,000	1,700	1,300	1,000	2,500	300	-	3,900	2,200	800	Darton Commodities Ltd. Annual Cobalt Market Review	-
**Total DRC artisanal processing (rounded to nearest 1,000)**					**-**	**-**	**1,000**	**1,000**	**1,000**	**1,000**	**2,000**	**2,000**	**3,000**	**10,000**	**10,000**	**9,000**	**7,000**	**6,000**	**6,000**	**10,000**	**7,000**	**9,000**	**16,000**	**11,000**	**7,000**		

*Assumed to be full artisanal if no mine ownership, no reported feed source, or reported "third party including artisanal" feed

Dataset S5: China's cobalt ore/concentrate imports reported by China Customs (HTS code 2605, metric tons)

5a: Gross weight of imports (metric tons, gross weight)

	2000	2001	2002	2003	2004	2005	2006	2007	2008	2009	2010	2011	2012	2013	2014	2015	2016	2017	2018	2019	2020	References	Notes
Albania	-	-	-	-	-	-	-	-	-	-	-	-	-	-	-	-	-	60	-	-	-	IHS Markit, Global Trade Atlas (2022).	China's reported imports of HTS code 2605 (cobalt ores/concentrates).
Australia	1,035	1,442	2,140	1,557	1,456	2,382	297	205	1	-	-	-	-	5	-	-	-	-	1	2	-	IHS Markit, Global Trade Atlas (2022).	China's reported imports of HTS code 2605 (cobalt ores/concentrates).
Belgium	139	135	381	487	1,248	95	144	21	368	940	260	-	148	36	56	-	-	124	166	-	-	IHS Markit, Global Trade Atlas (2022).	China's reported imports of HTS code 2605 (cobalt ores/concentrates).
Brazil	-	-	-	-	-	-	-	-	-	-	-	-	-	-	-	-	-	18	-	-	-	IHS Markit, Global Trade Atlas (2022).	China's reported imports of HTS code 2605 (cobalt ores/concentrates).
Cameroon	-	-	-	-	-	-	-	-	-	-	-	1	-	-	-	-	-	-	-	-	-	IHS Markit, Global Trade Atlas (2022).	China's reported imports of HTS code 2605 (cobalt ores/concentrates).
Canada	37	6	-	-	1,457	4,135	3,798	2,556	898	-	790	1,933	372	-	-	-	-	-	-	-	-	IHS Markit, Global Trade Atlas (2022).	China's reported imports of HTS code 2605 (cobalt ores/concentrates).
Congo	1,403	3,894	9,539	28,484	62,882	50,704	28,213	15,802	29,822	15,563	10,766	2,583	2,215	-	-	24	8	-	-	-	-	IHS Markit, Global Trade Atlas (2022).	China's reported imports of HTS code 2605 (cobalt ores/concentrates).
DRC	-	7,905	14,687	38,631	55,476	105,328	113,175	85,198	206,789	241,727	316,212	323,683	166,437	176,401	188,576	224,750	132,673	99,868	136,421	86,164	52,850	IHS Markit, Global Trade Atlas (2022).	China's reported imports of HTS code 2605 (cobalt ores/concentrates).
Côte d'Ivoire	-	-	-	-	-	-	508	103	-	-	-	-	-	-	-	-	-	-	-	-	-	IHS Markit, Global Trade Atlas (2022).	China's reported imports of HTS code 2605 (cobalt ores/concentrates).
Countries and territories not specified	-	-	-	-	-	-	17	-	63	95	-	101	-	-	-	-	-	-	-	-	-	IHS Markit, Global Trade Atlas (2022).	China's reported imports of HTS code 2605 (cobalt ores/concentrates).
Cuba	-	4,542	6,685	5,298	6,756	4,460	4,504	7,460	9,224	7,950	10,367	7,141	4,749	3,538	2,746	-	-	-	-	-	-	IHS Markit, Global Trade Atlas (2022).	China's reported imports of HTS code 2605 (cobalt ores/concentrates).
Egypt	-	56	-	-	-	-	-	-	-	-	-	-	-	-	-	-	-	-	-	-	-	IHS Markit, Global Trade Atlas (2022).	China's reported imports of HTS code 2605 (cobalt ores/concentrates).
Finland	-	-	-	-	-	0	-	-	-	-	-	-	-	-	-	-	-	-	-	-	-	IHS Markit, Global Trade Atlas (2022).	China's reported imports of HTS code 2605 (cobalt ores/concentrates).
France	-	30	-	-	15	-	-	-	-	-	-	-	-	-	-	-	-	-	-	-	-	IHS Markit, Global Trade Atlas (2022).	China's reported imports of HTS code 2605 (cobalt ores/concentrates).
Germany	306	-	-	-	-	38	-	167	-	-	19	5	-	-	-	-	-	-	-	-	-	IHS Markit, Global Trade Atlas (2022).	China's reported imports of HTS code 2605 (cobalt ores/concentrates).
Hong Kong	-	0	0	-	0	-	-	-	-	-	-	-	-	-	-	-	-	-	-	-	-	IHS Markit, Global Trade Atlas (2022).	China's reported imports of HTS code 2605 (cobalt ores/concentrates).
India	252	140	-	-	-	-	-	-	-	-	-	-	-	-	-	-	-	-	-	-	-	IHS Markit, Global Trade Atlas (2022).	China's reported imports of HTS code 2605 (cobalt ores/concentrates).
Indonesia	-	-	-	-	-	-	-	0	-	-	-	-	-	-	-	-	-	-	-	-	-	IHS Markit, Global Trade Atlas (2022).	China's reported imports of HTS code 2605 (cobalt ores/concentrates).
Iran (Islamic Rep. of)	-	-	-	-	-	-	-	-	-	17	192	-	-	2	-	-	-	-	-	-	-	IHS Markit, Global Trade Atlas (2022).	China's reported imports of HTS code 2605 (cobalt ores/concentrates).
Israel	-	-	-	-	-	-	-	-	45	-	-	-	-	-	-	-	-	-	-	-	-	IHS Markit, Global Trade Atlas (2022).	China's reported imports of HTS code 2605 (cobalt ores/concentrates).
Italy	2	-	-	-	-	-	-	-	-	-	-	-	-	-	-	-	-	-	-	-	-	IHS Markit, Global Trade Atlas (2022).	China's reported imports of HTS code 2605 (cobalt ores/concentrates).
Japan	128	236	227	217	282	-	52	90	173	140	38	25	59	-	68	-	-	-	-	-	-	IHS Markit, Global Trade Atlas (2022).	China's reported imports of HTS code 2605 (cobalt ores/concentrates).
Kenya	-	-	-	-	-	-	-	-	170	-	-	-	-	-	-	-	-	-	-	-	-	IHS Markit, Global Trade Atlas (2022).	China's reported imports of HTS code 2605 (cobalt ores/concentrates).
Korea (Rep. of)	185	17	-	699	1,288	1,331	184	14	17	-	-	0	-	-	-	-	-	-	336	-	-	IHS Markit, Global Trade Atlas (2022).	China's reported imports of HTS code 2605 (cobalt ores/concentrates).
Malaysia	-	-	-	-	0	-	-	-	-	-	-	-	-	-	-	-	-	-	-	-	-	IHS Markit, Global Trade Atlas (2022).	China's reported imports of HTS code 2605 (cobalt ores/concentrates).
Mexico	-	-	-	-	612	-	175	313	369	248	396	-	-	-	-	-	-	-	-	-	-	IHS Markit, Global Trade Atlas (2022).	China's reported imports of HTS code 2605 (cobalt ores/concentrates).
Morocco	-	3,121	117	-	-	-	-	-	-	935	-	-	-	-	-	-	-	-	-	-	-	IHS Markit, Global Trade Atlas (2022).	China's reported imports of HTS code 2605 (cobalt ores/concentrates).
Netherlands	-	-	-	394	-	2,105	-	-	-	-	-	-	-	-	-	-	273	-	-	-	-	IHS Markit, Global Trade Atlas (2022).	China's reported imports of HTS code 2605 (cobalt ores/concentrates).
Pakistan	-	-	-	-	-	-	-	-	-	-	57	-	74	-	-	-	-	-	-	-	-	IHS Markit, Global Trade Atlas (2022).	China's reported imports of HTS code 2605 (cobalt ores/concentrates).
Philippines	-	-	-	156	-	-	-	-	-	-	-	-	-	-	-	-	87	-	-	-	-	IHS Markit, Global Trade Atlas (2022).	China's reported imports of HTS code 2605 (cobalt ores/concentrates).
Russian Federation	-	-	-	-	19	-	-	-	-	-	-	-	-	-	-	-	-	-	-	-	-	IHS Markit, Global Trade Atlas (2022).	China's reported imports of HTS code 2605 (cobalt ores/concentrates).
Saudi Arabia	-	-	-	32	60	-	-	-	-	-	-	-	-	-	-	-	-	-	-	-	-	IHS Markit, Global Trade Atlas (2022).	China's reported imports of HTS code 2605 (cobalt ores/concentrates).
Singapore	-	-	-	-	-	-	14	-	134	-	-	-	-	-	-	-	-	-	-	-	-	IHS Markit, Global Trade Atlas (2022).	China's reported imports of HTS code 2605 (cobalt ores/concentrates).
Slovakia	-	-	-	-	-	-	-	-	-	-	-	-	-	-	-	-	-	-	-	-	10	IHS Markit, Global Trade Atlas (2022).	China's reported imports of HTS code 2605 (cobalt ores/concentrates).
South Africa	9,630	4,780	4,521	6,247	10,639	26,603	13,642	4,707	4,070	12,658	9,235	11,890	2,014	-	711	-	27	47	19	2,489	-	IHS Markit, Global Trade Atlas (2022).	China's reported imports of HTS code 2605 (cobalt ores/concentrates).
Spain	2	-	-	-	37	23	119	-	-	-	-	-	-	-	-	-	-	-	-	-	-	IHS Markit, Global Trade Atlas (2022).	China's reported imports of HTS code 2605 (cobalt ores/concentrates).
Sudan	-	-	-	-	-	0	-	-	-	-	-	-	-	-	-	-	-	-	-	-	-	IHS Markit, Global Trade Atlas (2022).	China's reported imports of HTS code 2605 (cobalt ores/concentrates).
Taiwan	38	117	33	681	735	358	212	321	82	-	-	-	-	-	-	-	-	-	-	-	-	IHS Markit, Global Trade Atlas (2022).	China's reported imports of HTS code 2605 (cobalt ores/concentrates).
Tanzania (United Rep. of)	-	-	-	210	-	559	228	504	-	481	-	70	-	-	22	1,018	5,273	286	-	-	-	IHS Markit, Global Trade Atlas (2022).	China's reported imports of HTS code 2605 (cobalt ores/concentrates).
Thailand	-	-	-	-	-	-	-	-	-	-	-	-	-	-	-	-	-	218	-	-	-	IHS Markit, Global Trade Atlas (2022).	China's reported imports of HTS code 2605 (cobalt ores/concentrates).
Uganda	-	-	-	-	-	-	-	499	-	-	-	-	-	-	-	-	-	-	-	-	-	IHS Markit, Global Trade Atlas (2022).	China's reported imports of HTS code 2605 (cobalt ores/concentrates).
United Arab Emirates	-	-	-	-	-	62	-	-	-	18	-	-	-	-	-	-	-	-	-	-	-	IHS Markit, Global Trade Atlas (2022).	China's reported imports of HTS code 2605 (cobalt ores/concentrates).
United States	1,808	1,313	164	573	546	488	279	68	323	2,026	2,048	262	-	11	99	12	-	-	-	-	-	IHS Markit, Global Trade Atlas (2022).	China's reported imports of HTS code 2605 (cobalt ores/concentrates).
Viet-Nam	-	-	-	-	-	-	-	-	-	53	-	-	236	-	-	-	-	-	-	-	-	IHS Markit, Global Trade Atlas (2022).	China's reported imports of HTS code 2605 (cobalt ores/concentrates).
Zambia	1,073	1,489	2,629	-	86	402	135	327	2,187	522	214	1,261	306	215	122	1,936	11,155	111	1,023	1,642	0	IHS Markit, Global Trade Atlas (2022).	China's reported imports of HTS code 2605 (cobalt ores/concentrates).
**Total Chinese ore/concentrate imports from world (repo**	**16,038**	**29,222**	**41,123**	**83,666**	**143,594**	**199,072**	**165,696**	**118,353**	**254,736**	**283,373**	**350,594**	**348,956**	**176,610**	**180,208**	**192,400**	**227,740**	**149,497**	**100,732**	**137,966**	**90,297**	**52,860**	-	-
**Total world trade of cobalt ores/concentrates (gross we**	**100,359**	**103,411**	**117,483**	**140,512**	**254,434**	**275,758**	**218,633**	**161,403**	**293,498**	**307,591**	**370,232**	**369,887**	**188,930**	**188,804**	**200,391**	**234,827**	**151,991**	**104,422**	**147,487**	**100,966**	**60,081**	IHS Markit, Global Trade Atlas (2022).	All countries' reported imports of HTS code 2605 (cobalt ores/concentrates).
**Chinese imports' share of world ore/concentrate trade**	**16%**	**28%**	**35%**	**60%**	**56%**	**72%**	**76%**	**73%**	**87%**	**92%**	**95%**	**94%**	**93%**	**95%**	**96%**	**97%**	**98%**	**96%**	**94%**	**89%**	**88%**	-	China's total imports minus total world imports.

5b: Cobalt content of imports (metric tons, cobalt content)*

	2000	2001	2002	2003	2004	2005	2006	2007	2008	2009	2010	2011	2012	2013	2014	2015	2016	2017	2018	2019	2020	References	Notes
Albania	-	-	-	-	-	-	-	-	-	-	-	-	-	-	-	-	-	5	-	-	-	-	Calculated at 7.6% cobalt content
Australia	79	110	163	118	111	181	23	16	0	-	-	-	-	0	-	-	-	-	0	0	-	-	Calculated at 7.6% cobalt content
Belgium	11	10	29	37	95	7	11	2	28	71	20	-	11	3	4	-	-	9	13	-	-	-	Calculated at 7.6% cobalt content
Brazil	-	-	-	-	-	-	-	-	-	-	-	-	-	-	-	-	-	1	-	-	-	-	Calculated at 7.6% cobalt content
Cameroon	-	-	-	-	-	-	-	-	-	-	-	0	-	-	-	-	-	-	-	-	-	-	Calculated at 7.6% cobalt content
Canada	3	0	-	-	111	314	289	194	68	-	60	147	28	-	-	-	-	-	-	-	-	-	Calculated at 7.6% cobalt content
Congo	107	296	725	2,165	4,779	3,853	2,144	1,201	2,266	1,183	818	196	168	-	-	2	1	-	-	-	-	-	Calculated at 7.6% cobalt content
DRC	-	601	1,116	2,936	4,216	8,005	8,601	6,475	15,716	18,371	24,032	24,600	12,649	13,407	14,332	17,081	10,083	7,590	10,368	6,548	4,017	-	Calculated at 7.6% cobalt content
Côte d'Ivoire	-	-	-	-	-	-	39	8	-	-	-	-	-	-	-	-	-	-	-	-	-	-	Calculated at 7.6% cobalt content
Countries and territories not specified	-	-	-	-	-	-	1	-	5	7	-	8	-	-	-	-	-	-	-	-	-	-	Calculated at 7.6% cobalt content
Cuba	-	345	508	403	513	339	342	567	701	604	788	543	361	269	209	-	-	-	-	-	-	-	Calculated at 7.6% cobalt content
Egypt	-	4	-	-	-	-	-	-	-	-	-	-	-	-	-	-	-	-	-	-	-	-	Calculated at 7.6% cobalt content
Finland	-	-	-	-	-	0	-	-	-	-	-	-	-	-	-	-	-	-	-	-	-	-	Calculated at 7.6% cobalt content
France	-	2	-	-	1	-	-	-	-	-	-	-	-	-	-	-	-	-	-	-	-	-	Calculated at 7.6% cobalt content
Germany	23	-	-	-	-	3	-	13	-	-	1	0	-	-	-	-	-	-	-	-	-	-	Calculated at 7.6% cobalt content
Hong Kong	-	0	0	-	0	-	-	-	-	-	-	-	-	-	-	-	-	-	-	-	-	-	Calculated at 7.6% cobalt content
India	19	11	-	-	-	-	-	-	-	-	-	-	-	-	-	-	-	-	-	-	-	-	Calculated at 7.6% cobalt content
Indonesia	-	-	-	-	-	-	-	0	-	-	-	-	-	-	-	-	-	-	-	-	-	-	Calculated at 7.6% cobalt content
Iran (Islamic Rep. of)	-	-	-	-	-	-	-	-	-	1	15	-	-	0	-	-	-	-	-	-	-	-	Calculated at 7.6% cobalt content
Israel	-	-	-	-	-	-	-	-	3	-	-	-	-	-	-	-	-	-	-	-	-	-	Calculated at 7.6% cobalt content
Italy	0	-	-	-	-	-	-	-	-	-	-	-	-	-	-	-	-	-	-	-	-	-	Calculated at 7.6% cobalt content
Japan	10	18	17	17	21	-	4	7	13	11	3	2	4	-	5	-	-	-	-	-	-	-	Calculated at 7.6% cobalt content
Kenya	-	-	-	-	-	-	-	-	13	-	-	-	-	-	-	-	-	-	-	-	-	-	Calculated at 7.6% cobalt content
Korea (Rep. of)	14	1	-	53	98	101	14	1	1	-	-	0	-	-	-	-	-	-	26	-	-	-	Calculated at 7.6% cobalt content
Malaysia	-	-	-	-	0	-	-	-	-	-	-	-	-	-	-	-	-	-	-	-	-	-	Calculated at 7.6% cobalt content
Mexico	-	-	-	-	47	-	13	24	28	19	30	-	-	-	-	-	-	-	-	-	-	-	Calculated at 7.6% cobalt content
Morocco	-	237	9	-	-	-	-	-	-	71	-	-	-	-	-	-	-	-	-	-	-	-	Calculated at 7.6% cobalt content
Netherlands	-	-	-	30	-	160	-	-	-	-	-	-	-	-	-	-	21	-	-	-	-	-	Calculated at 7.6% cobalt content
Pakistan	-	-	-	-	-	-	-	-	-	-	4	-	6	-	-	-	-	-	-	-	-	-	Calculated at 7.6% cobalt content
Philippines	-	-	-	12	-	-	-	-	-	-	-	-	-	-	-	-	7	-	-	-	-	-	Calculated at 7.6% cobalt content
Russian Federation	-	-	-	-	1	-	-	-	-	-	-	-	-	-	-	-	-	-	-	-	-	-	Calculated at 7.6% cobalt content
Saudi Arabia	-	-	-	2	5	-	-	-	-	-	-	-	-	-	-	-	-	-	-	-	-	-	Calculated at 7.6% cobalt content
Singapore	-	-	-	-	-	-	1	-	10	-	-	-	-	-	-	-	-	-	-	-	-	-	Calculated at 7.6% cobalt content
Slovakia	-	-	-	-	-	-	-	-	-	-	-	-	-	-	-	-	-	-	-	-	1	-	Calculated at 7.6% cobalt content
South Africa	732	363	344	475	809	2,022	1,037	358	309	962	702	904	153	-	54	-	2	4	1	189	-	-	Calculated at 7.6% cobalt content
Spain	0	-	-	-	3	2	9	-	-	-	-	-	-	-	-	-	-	-	-	-	-	-	Calculated at 7.6% cobalt content
Sudan	-	-	-	-	-	0	-	-	-	-	-	-	-	-	-	-	-	-	-	-	-	-	Calculated at 7.6% cobalt content
Taiwan	3	9	3	52	56	27	16	24	6	-	-	-	-	-	-	-	-	-	-	-	-	-	Calculated at 7.6% cobalt content
Tanzania (United Rep. of)	-	-	-	16	-	42	17	38	-	37	-	5	-	-	2	77	401	22	-	-	-	-	Calculated at 7.6% cobalt content
Thailand	-	-	-	-	-	-	-	-	-	-	-	-	-	-	-	-	-	17	-	-	-	-	Calculated at 7.6% cobalt content
Uganda	-	-	-	-	-	-	-	38	-	-	-	-	-	-	-	-	-	-	-	-	-	-	Calculated at 7.6% cobalt content
United Arab Emirates	-	-	-	-	-	5	-	-	-	1	-	-	-	-	-	-	-	-	-	-	-	-	Calculated at 7.6% cobalt content
United States	137	100	12	44	41	37	21	5	25	154	156	20	-	1	8	1	-	-	-	-	-	-	Calculated at 7.6% cobalt content
Viet-Nam	-	-	-	-	-	-	-	-	-	4	-	-	18	-	-	-	-	-	-	-	-	-	Calculated at 7.6% cobalt content
Zambia	82	113	200	-	7	31	10	25	166	40	16	96	23	16	9	147	848	8	78	125	0	-	Calculated at 7.6% cobalt content
**Total Chinese ore/concentrate imports from world repo**	**1,200**	**2,200**	**3,100**	**6,400**	**10,900**	**15,100**	**12,600**	**9,000**	**19,400**	**21,500**	**26,600**	**26,500**	**13,400**	**13,700**	**14,600**	**17,300**	**11,400**	**7,700**	**10,500**	**6,900**	**4,000**	-	-

* Cobalt content assumed to equal 7.6% of the gross weight of imports.

5c: Cobalt content of Chinese ore/concentrate imports from the DRC, assumed transit countries, and rest of world (metric tons, cobalt content)

	2000	2001	2002	2003	2004	2005	2006	2007	2008	2009	2010	2011	2012	2013	2014	2015	2016	2017	2018	2019	2020	References	Notes
DRC	-	600	1,100	2,900	4,200	8,000	8,600	6,500	15,700	18,400	24,000	24,600	12,600	13,400	14,300	17,100	10,100	7,600	10,400	6,500	4,000	**0**	Condensation of Dataset S 5B to show DRC and transit countries.
Congo (Brazzaville)	100	300	700	2,200	4,800	3,900	2,100	1,200	2,300	1,200	800	200	200	-	-	-	-	-	-	-	-	-	Condensation of Dataset S 5B to show DRC and transit countries.
South Africa	700	400	300	500	800	2,000	1,000	400	300	1,000	700	900	200	-	100	-	-	-	-	200	-	-	Condensation of Dataset S 5B to show DRC and transit countries.
Zambia	100	100	200	-	-	-	-	-	200	-	-	100	-	-	-	100	800	-	100	100	-	-	Condensation of Dataset S 5B to show DRC and transit countries.
Rest of World	300	800	800	800	1,100	1,200	900	900	900	900	1,100	700	400	300	200	100	500	100	-	100	-	-	Condensation of Dataset S 5B to show DRC and transit countries.
**Chinese ore/concentrate imports from the DRC and tran**	**900**	**1,400**	**2,300**	**5,600**	**9,800**	**13,900**	**11,700**	**8,100**	**18,500**	**20,600**	**25,500**	**25,800**	**13,000**	**13,400**	**14,400**	**17,200**	**10,900**	**7,600**	**10,500**	**6,800**	**4,000**	-	**Base number for "China's ore and concentrate imports from the DRC"**
**DRC share of total Chinese imports (%)**	**0%**	**27%**	**35%**	**45%**	**39%**	**53%**	**68%**	**72%**	**81%**	**86%**	**90%**	**93%**	**94%**	**98%**	**98%**	**99%**	**89%**	**99%**	**99%**	**94%**	**100%**		
**DRC plus transit countries share of total Chinese imports**	**75%**	**64%**	**74%**	**88%**	**90%**	**92%**	**93%**	**90%**	**95%**	**96%**	**96%**	**97%**	**97%**	**98%**	**99%**	**99%**	**96%**	**99%**	**100%**	**99%**	**100%**		

Dataset S6: Cobalt ore/concentrate exports to China reported by the Customs Ministries of the Democratic Republic of the Congo, South Africa, Zambia, and Congo (Brazzaville) (HTS code 2605, metric tons)

6a: Gross weight of exports (metric tons, gross weight)

0.00%	2000	2001	2002	2003	2004	2005	2006	2007	2008	2009	2010	2011	2012	2013	2014	2015	2016	2017	2018	2019	2020	References	Notes
DRC																124,741	67,468	63,558	40,979	5,355	3,000	IHS Markit, Global Trade Atlas (2022).	-
South Africa			4,657	21,120	26,090	62,998	44,229	24,007	1,582	3,789	541	3,431	2,750	2,873	147	-	1,574	56	55	23	49	IHS Markit, Global Trade Atlas (2022).	-
Zambia										5,932	2,720	415	169	223	-	50	-	-	369	-	156	IHS Markit, Global Trade Atlas (2022).	-
Congo (Brazzaville)																						IHS Markit, Global Trade Atlas (2022).	-
**DRC, South Africa, Zambia, and Congo (Brazzaville) ore/c**	**-**	**-**	**4,657**	**21,120**	**26,090**	**62,998**	**44,229**	**24,007**	**1,582**	**9,721**	**3,261**	**3,846**	**2,919**	**3,096**	**147**	**124,791**	**69,042**	**63,614**	**41,403**	**5,378**	**3,205**	-	-

6b: Cobalt content of exports (metric tons, cobalt content)*

	2000	2001	2002	2003	2004	2005	2006	2007	2008	2009	2010	2011	2012	2013	2014	2015	2016	2017	2018	2019	2020	References	Notes
DRC			-	-	-	-	-	-	-	-	-	-	-	-	-	9,500	5,100	4,800	3,100	400	200	-	Calculated at 7.6% cobalt content
South Africa			400	1,600	2,000	4,800	3,400	1,800	100	300	-	300	200	200	-	-	100	-	-	-	-	-	Calculated at 7.6% cobalt content
Zambia			-	-	-	-	-	-	-	500	200	-	-	-	-	-	-	-	-	-	-	-	Calculated at 7.6% cobalt content
Congo (Brazzaville)			-	-	-	-	-	-	-	-	-	-	-	-	-	-	-	-	-	-	-	-	Calculated at 7.6% cobalt content
**DRC, South Africa, Zambia, and Congo (Brazzaville) ore/concentrate exports to Ch**			**400**	**1,600**	**2,000**	**4,800**	**3,400**	**1,800**	**100**	**800**	**200**	**300**	**200**	**200**	**-**	**9,500**	**5,200**	**4,800**	**3,100**	**400**	**200**	-	-

* Cobalt content assumed to equal 7.6% of the gross weight of imports.

Dataset S7: Cobalt ore/concentrate imports from the DRC reported by the importers' Customs Ministries (HTS code 2605, metric tons)

7a: Gross weight of imports (metric tons, gross weight)

	2000	2001	2002	2003	2004	2005	2006	2007	2008	2009	2010	2011	2012	2013	2014	2015	2016	2017	2018	2019	2020	References	Notes
Belgium	-	-	-	-	63	131	-	-	-	-	-	2	-	-	-	-	-	-	-	-	-	IHS Markit, Global Trade Atlas (2022).	-
Botswana	-	-	-	-	-	2,848	-	-	-	-	-	-	-	-	-	-	-	-	-	-	-	IHS Markit, Global Trade Atlas (2022).	-
Brazil	-	-	-	-	-	-	-	65	-	-	27	-	-	-	-	-	-	-	-	-	-	IHS Markit, Global Trade Atlas (2022).	-
China (People's Republic of)	-	7,905	14,687	38,631	55,476	105,328	113,175	85,198	206,789	241,727	316,212	323,683	166,437	176,401	188,576	224,750	132,673	99,868	136,421	86,164	52,850	IHS Markit, Global Trade Atlas (2022).	-
Finland	-	-	64,274	41,570	79,494	50,711	28,167	18,676	15,456	5,733	520	-	-	845	-	-	-	-	-	-	-	IHS Markit, Global Trade Atlas (2022).	-
Hong Kong	-	-	-	-	-	-	-	-	606	40	-	450	-	-	-	-	-	-	-	-	-	IHS Markit, Global Trade Atlas (2022).	-
India	-	-	-	-	-	-	-	3,572	4,704	4,542	5,086	168	133	-	-	30	20	-	-	-	-	IHS Markit, Global Trade Atlas (2022).	-
Japan	-	-	-	-	-	-	-	-	-	-	-	-	-	43	-	-	-	-	-	-	-	IHS Markit, Global Trade Atlas (2022).	-
Korea (Rep. of)	-	-	-	-	-	-	-	-	-	0	-	-	0	-	-	-	-	0	152	127	-	IHS Markit, Global Trade Atlas (2022).	-
Morocco	-	-	-	-	-	-	-	870	-	-	327	-	-	-	-	-	-	1,070	5,098	7,025	6,881	IHS Markit, Global Trade Atlas (2022).	-
Namibia (source: UN Comtrade)	-	-	-	25	-	-	-	-	-	-	-	-	-	-	-	-	-	-	-	-	-	IHS Markit, Global Trade Atlas (2022).	-
South Africa	-	-	4,457	5,027	10,491	5,234	8,824	6,375	4	131	154	29	-	-	1	32	2	0	-	8	0	IHS Markit, Global Trade Atlas (2022).	-
Taiwan	-	-	-	-	-	-	-	-	-	-	-	-	-	-	-	-	-	-	-	-	102	IHS Markit, Global Trade Atlas (2022).	-
Ukraine	-	-	-	-	-	-	-	-	-	-	-	22	-	-	-	-	-	-	-	-	-	IHS Markit, Global Trade Atlas (2022).	-
United Kingdom	-	-	-	-	17	-	-	-	-	-	-	-	-	-	-	-	-	-	-	-	-	IHS Markit, Global Trade Atlas (2022).	-
Zambia	-	-	-	-	-	-	-	-	-	41,107	71,328	50,862	75,383	61,417	84,464	93,945	61,625	59,585	48,251	-	-	IHS Markit, Global Trade Atlas (2022).	-
Total	-	7,905	83,418	85,253	145,540	164,253	150,166	114,756	227,560	293,280	393,653	375,215	241,953	238,706	273,041	318,757	194,320	160,524	189,922	93,324	59,833	-	-

7b: Cobalt content of imports (metric tons, gross weight)

	2000	2001	2002	2003	2004	2005	2006	2007	2008	2009	2010	2011	2012	2013	2014	2015	2016	2017	2018	2019	2020	References	Notes
Belgium	-	-	-	-	5	10	-	-	-	-	-	0	-	-	-	-	-	-	-	-	-	-	Calculated at 7.6% cobalt content
Botswana	-	-	-	-	-	216	-	-	-	-	-	-	-	-	-	-	-	-	-	-	-	-	Calculated at 7.6% cobalt content
Brazil	-	-	-	-	-	-	-	5	-	-	2	-	-	-	-	-	-	-	-	-	-	-	Calculated at 7.6% cobalt content
China (People's Republic of)	-	601	1,116	2,936	4,216	8,005	8,601	6,475	15,716	18,371	24,032	24,600	12,649	13,407	14,332	17,081	10,083	7,590	10,368	6,548	4,017	-	Calculated at 7.6% cobalt content
Finland	-	-	4,885	3,159	6,042	3,854	2,141	1,419	1,175	436	40	-	-	64	-	-	-	-	-	-	-	-	Calculated at 7.6% cobalt content
Hong Kong	-	-	-	-	-	-	-	-	46	3	-	34	-	-	-	-	-	-	-	-	-	-	Calculated at 7.6% cobalt content
India	-	-	-	-	-	-	-	271	358	345	387	13	10	-	-	2	2	-	-	-	-	-	Calculated at 7.6% cobalt content
Japan	-	-	-	-	-	-	-	-	-	-	-	-	-	3	-	-	-	-	-	-	-	-	Calculated at 7.6% cobalt content
Korea (Rep. of)	-	-	-	-	-	-	-	-	-	0	-	-	0	-	-	-	-	0	12	10	-	-	Calculated at 7.6% cobalt content
Morocco	-	-	-	-	-	-	-	66	-	-	25	-	-	-	-	-	-	81	387	534	523	-	Calculated at 7.6% cobalt content
Namibia (source: UN Comtrade)	-	-	-	2	-	-	-	-	-	-	-	-	-	-	-	-	-	-	-	-	-	-	Calculated at 7.6% cobalt content
South Africa	-	-	339	382	797	398	671	485	0	10	12	2	-	-	0	2	0	0	-	1	0	-	Calculated at 7.6% cobalt content
Taiwan	-	-	-	-	-	-	-	-	-	-	-	-	-	-	-	-	-	-	-	-	8	-	Calculated at 7.6% cobalt content
Ukraine	-	-	-	-	-	-	-	-	-	-	-	2	-	-	-	-	-	-	-	-	-	-	Calculated at 7.6% cobalt content
United Kingdom	-	-	-	-	1	-	-	-	-	-	-	-	-	-	-	-	-	-	-	-	-	-	Calculated at 7.6% cobalt content
Zambia	-	-	-	-	-	-	-	-	-	3,124	5,421	3,865	5,729	4,668	6,419	7,140	4,683	4,528	3,667	-	-	-	Calculated at 7.6% cobalt content
**Total**	**-**	**601**	**6,340**	**6,479**	**11,061**	**12,483**	**11,413**	**8,721**	**17,295**	**22,289**	**29,918**	**28,516**	**18,388**	**18,142**	**20,751**	**24,226**	**14,768**	**12,200**	**14,434**	**7,093**	**4,547**	-	Calculated at 7.6% cobalt content

Dataset S8: Industrial and artisanal Chinese imports, as well as artisanal-production-B results for 2000 through 2008

8a: Industrial Chinese imports of cobalt ores and concentrates from the DRC from 2000 through 2008 (based on production reported to be exported to China)

Reported feed sources*	Contemporary mine/processing facility operator	Contemporary mine/processing facility name	Predominant material	2000	2001	2002	2003	2004	2005	2006	2007	2008	2009*	2010*	2011*	2012*	2013*	2014*	2015*	2016*	2017*	2018*	2019*	2020*	References	Notes
Own mines	Glencore plc	Kamoto / Katanga Mining / KCC (Kamoto Copper Company)	Home processed concentrates	-	-	-	-	-	-	-	-	1,200													K. B. Shedd, "Cobalt" in 2008 Minerals Yearbook, (US Geological Survey, 2011).	
Own mines	Eurasian Resources Group	Boss Mining (Kababankola mine, Mukondo Mountain mine)	Home processed concentrates	-	-	100	2,200	3,300	4,400	4,500	-	3,600													K. B. Shedd, "Cobalt" in 2002 Minerals Yearbook, (US Geological Survey, 2005); K. B. Shedd, "Cobalt" in 2005 Minerals Yearbook, (US Geological Survey, 2008).	Export to China was the alternative to refining at Shituru. At the time, KMC as the only major mining operation running throughput through Shituru. So, this number is the leftover production from KMC that would have to be exported to China because it could not be refined at Shituru. '02MYB KMC had been toll refining at Shituru, but "considered other options because of problems at Shituru."
**Industrial Chinese ore/concentrate imports from DRC**				**-**	**-**	**100**	**2,200**	**3,300**	**4,400**	**4,500**	**-**	**4,800**														**China's industrial imports for the years 2009 through 2020 rely on a dataset, not published until 2009, by the provincial divisions of the DRC Ministry of Mines.**

8b: Artisanal Chinese imports (i.e., second component of artisanal-production-B) of cobalt ores and concentrates from the DRC from 2000 through 2008

				2000	2001	2002	2003	2004	2005	2006	2007	2008	2009*	2010*	2011*	2012*	2013*	2014*	2015*	2016*	2017*	2018*	2019*	2020*	References	Notes
Total China Customs imports from DRC				1,000	1,400	2,300	5,600	9,800	13,900	11,700	8,100	18,500														From Dataset S 5C
Industrial Chinese imports				-	-	100	2,200	3,300	4,400	4,500	-	4,800														From Dataset S 4C
**Artisanal Chinese imports from the DRC (rounded to the nearest thousand)**				**1,000**	**1,000**	**2,000**	**3,000**	**7,000**	**10,000**	**7,000**	**8,000**	**14,000**														**China's artisanal imports for the years 2009 through 2020 rely on a dataset, not published until 2009, by the provincial divisions of the DRC Ministry of Mines.**

8c: Artisanal-production-B results for artisanal processing and imports from 2000 through 2008

				2000	2001	2002	2003	2004	2005	2006	2007	2008	2009*	2010*	2011*	2012*	2013*	2014*	2015*	2016*	2017*	2018*	2019*	2020*	References	Notes
Artisanal processing (rounded to nearest thousand)				-	-	1,000	1,000	1,000	1,000	2,000	2,000	3,000														From Dataset S4B.
Artisanal imports (rounded to nearest thousand)				1,000	1,000	2,000	3,000	7,000	10,000	7,000	8,000	14,000														From Dataset S4D.
**Artisanal-production-B, artisanal processing and imports (results)**				1,000	1,000	3,000	4,000	8,000	11,000	9,000	10,000	17,000														**Artisanal-production-B results from 2000 through 2008**

* Data, assumptions, calculations and results from 2009 through 2020 are presented in Supporting Datasets 9a through 9h.

Dataset S9: Democratic Republic of the Congo (DRC) cobalt ore/concentrate exports by gross weight, cobalt content, production type, and destination, as well as artisanal-production-B results from 2009 (first year of reporting) through 2020

9a: Export of cobalt ores and concentrates by company, as reported by the DRC Ministry of Mines provincial divisions for Katanga, Haut Katanga, and Lualaba (metric tons, gross weight)

Company	Material	Measurement	2000*	2001*	2002*	2003*	2004*	2005*	2006*	2007*	2008*	2009	2010	2011	2012	2013	2014	2015	2016	2017	2018	2019	2020	References	Notes
Bazano	Cobalt concentrate	Metric tons of gross weight										16,952	31,503	51,474	8,537	3,273	-	-	-	-	-	-	-	Province du Katanga Division Provinciale de Mines, “Statist	-
Bolfast	Cobalt concentrate	Metric tons of gross weight										6,335	13,818	16,150	7,630	4,626	5,273	8,190	1,911	200	-	-	-	Province du Katanga Division Provinciale de Mines, “Statist	-
Boss	Cobalt concentrate	Metric tons of gross weight										44,720	50,213	46,713	21,077	26,396	3,311	30,941	7,838	45,533	34,647	-	-	Province du Katanga Division Provinciale de Mines, “Statist	-
CDM	Cobalt concentrate	Metric tons of gross weight										16,588	21,166	28,673	34,532	29,042	62,933	52,420	3,271	-	-	-	-	Province du Katanga Division Provinciale de Mines, “Statist	-
Cota Mining	Cobalt concentrate	Metric tons of gross weight										1,204	564	-	-	-	-	-	-	-	-	-	-	Province du Katanga Division Provinciale de Mines, “Statist	-
Eagle Group	Cobalt concentrate	Metric tons of gross weight										-	-	-	-	4,646	540	-	-	-	-	-	-	Province du Katanga Division Provinciale de Mines, “Statist	-
Exaco (Exploitation artisanale du Congo)	Cobalt concentrate	Metric tons of gross weight										11,681	10,283	2,833	1,093	-	-	-	-	-	-	-	-	Province du Katanga Division Provinciale de Mines, “Statist	-
FEZA	Cobalt concentrate	Metric tons of gross weight										-	-	-	-	-	-	11,270	1,202	-	-	-	-	Province du Katanga Division Provinciale de Mines, “Statist	-
Huachin Mining (CNMC)	Cobalt concentrate	Metric tons of gross weight										-	-	-	-	-	2,425	11,575	-	-	-	-	-	Province du Katanga Division Provinciale de Mines, “Statist	-
KMP (Katanga Minerals Processing)	Cobalt concentrate	Metric tons of gross weight										-	1,854	-	-	-	-	-	-	-	-	-	-	Province du Katanga Division Provinciale de Mines, “Statist	-
Metal Mines (MTM)	Cobalt concentrate	Metric tons of gross weight										15,018	12,774	5,766	9,275	14,300	27,375	32,600	15,810	19,734	14,799	159	-	Province du Katanga Division Provinciale de Mines, “Statist	-
MJM (Jiana Energy, formerly Macrolink Jiayuan Mining SARL)	Cobalt concentrate	Metric tons of gross weight										-	610	1,855	-	2,290	5,400	4,394	14,228	30,879	6,198	-	-	Province du Katanga Division Provinciale de Mines, “Statist	-
MuMi (Mutanda) Export	Cobalt concentrate	Metric tons of gross weight										100,182	5,835	-	-	-	-	-	-	-	-	-	-	Province du Katanga Division Provinciale de Mines, “Statist	-
ROQ Mining	Cobalt concentrate	Metric tons of gross weight										-	445	-	-	-	-	-	-	-	-	-	-	Province du Katanga Division Provinciale de Mines, “Statist	-
Rubamin	Cobalt concentrate	Metric tons of gross weight										4,158	7,261	650	-	-	-	-	-	-	-	-	-	Province du Katanga Division Provinciale de Mines, “Statist	-
SARDC	Cobalt concentrate	Metric tons of gross weight										850	-	-	-	-	-	-	-	-	-	-	-	Province du Katanga Division Provinciale de Mines, “Statist	-
SODIMINCO	Cobalt concentrate	Metric tons of gross weight										-	-	-	-	1,085	-	-	-	-	-	-	-	Province du Katanga Division Provinciale de Mines, “Statist	-
SOMIKA	Cobalt concentrate	Metric tons of gross weight										24,742	24,332	16,097	8,519	950	3,176	1,202	-	-	-	-	-	Province du Katanga Division Provinciale de Mines, “Statist	-
Tengyuan Co&Cu Resources (TCC SARL)	Cobalt concentrate	Metric tons of gross weight										-	-	-	-	-	-	-	-	-	2,103	-	-	Province du Katanga Division Provinciale de Mines, “Statist	-
Thomas Mining SARL	Cobalt concentrate	Metric tons of gross weight										-	-	-	-	-	-	-	-	-	17,697	-	-	Province du Katanga Division Provinciale de Mines, “Statist	-
Volcano	Cobalt concentrate	Metric tons of gross weight										13,064	35,309	35,166	16,416	462	-	-	-	-	-	-	-	Province du Katanga Division Provinciale de Mines, “Statist	-
Bazano	Copper cobalt concentrate	Metric tons of gross weight										-	-	7,141	1,262	-	-	-	-	-	-	-	-	Province du Katanga Division Provinciale de Mines, “Statist	-
Boss	Copper cobalt concentrate	Metric tons of gross weight										21,588	50,233	58,737	87,518	96,648	117,487	47,601	58,854	5,038	2,319	-	-	Province du Katanga Division Provinciale de Mines, “Statist	-
CDM	Copper cobalt concentrate	Metric tons of gross weight										-	-	-	-	-	-	-	-	-	-	32,270	21,095	Province du Katanga Division Provinciale de Mines, “Statist	-
CMSK (Compagnie Minière du Sud Katanga)	Copper cobalt concentrate	Metric tons of gross weight										6,510	45,908	40,738	-	42,646	41,653	-	-	-	-	-	-	Province du Katanga Division Provinciale de Mines, “Statist	-
COMIKA	Copper cobalt concentrate	Metric tons of gross weight										-	-	-	-	-	-	-	31,020	45,823	46,170	26,190	-	Province du Katanga Division Provinciale de Mines, “Statist	-
DCP (Democratic Republic of Congo Copper and Cobalt Project)	Copper cobalt concentrate	Metric tons of gross weight										12,916	4,299	-	-	-	-	-	-	-	-	-	-	Province du Katanga Division Provinciale de Mines, “Statist	-
KCC (Kamoto Copper Company)	Copper cobalt concentrate	Metric tons of gross weight										-	19,081	85,420	98,853	135,003	-	-	-	-	-	-	-	Province du Katanga Division Provinciale de Mines, “Statist	-
MuMi (Mutanda)	Copper cobalt concentrate	Metric tons of gross weight										19,373	130,251	81,466	38,643	20,147	10,960	-	-	-	-	-	-	Province du Katanga Division Provinciale de Mines, “Statist	-
SICOMINES	Copper cobalt concentrate	Metric tons of gross weight										-	-	-	-	-	-	1,536	-	-	-	-	-	Province du Katanga Division Provinciale de Mines, “Statist	-
SODIMINCO	Copper cobalt concentrate	Metric tons of gross weight										-	-	-	-	980	-	-	-	-	-	-	-	Province du Katanga Division Provinciale de Mines, “Statist	-
**Total gross weight of ore/concentrate exports reported by DRC Ministry of Mines provincial divisions**												**315,881**	**465,739**	**478,879**	**333,355**	**382,494**	**280,533**	**201,729**	**134,134**	**147,207**	**123,933**	**58,619**	**21,095**	-	**The sum of DRC exports to the world (reported by the DRC Ministry of Mines) is larger than the sum of imports reported by the Customs Ministries of countries importing cobalt ores and concentrates from the DRC. This is most likely due to water loss during transport. Other reasons for the discrepancy may be that the material was: recorded at the mine but not for export taxes at DRC customs, miscategorized as another HTS code, or not recorded at the point of entry.**

9b. Percentage of cobalt contained in exports (%)

Company	Material	Measurement	2000*	2001*	2002*	2003*	2004*	2005*	2006*	2007*	2008*	2009	2010	2011	2012	2013	2014	2015	2016	2017	2018	2019	2020	References	Notes
Bazano	Cobalt concentrate	Percentage of cobalt content										5.77%	5.77%	5.77%	5.77%	5.77%	0.00%	0.00%	0.00%	0.00%	0.00%	0.00%	0.00%	EITI, “Extractive Industries Transparency Initiative Reports	When ranges are provided, the average is presented here.
Bolfast	Cobalt concentrate	Percentage of cobalt content										8.82%	8.82%	8.82%	8.82%	8.82%	8.82%	8.82%	8.82%	8.82%	0.00%	0.00%	0.00%	EITI, “Extractive Industries Transparency Initiative Reports	When ranges are provided, the average is presented here.
Boss	Cobalt concentrate	Percentage of cobalt content										9.50%	9.50%	9.50%	9.50%	9.50%	9.50%	9.50%	9.50%	9.50%	9.50%	0.00%	0.00%	EITI, “Extractive Industries Transparency Initiative Reports	When ranges are provided, the average is presented here.
CDM	Cobalt concentrate	Percentage of cobalt content										5.40%	5.40%	5.40%	5.40%	5.40%	8.45%	8.45%	8.45%	8.45%	0.00%	0.00%	0.00%	EITI, “Extractive Industries Transparency Initiative Reports	When ranges are provided, the average is presented here.
Cota Mining	Cobalt concentrate	Percentage of cobalt content										6.45%	6.45%	0.00%	0.00%	0.00%	0.00%	0.00%	0.00%	0.00%	0.00%	0.00%	0.00%	EITI, “Extractive Industries Transparency Initiative Reports	When ranges are provided, the average is presented here.
Eagle Group	Cobalt concentrate	Percentage of cobalt content										0.00%	0.00%	0.00%	0.00%	7.00%	7.00%	0.00%	0.00%	0.00%	0.00%	0.00%	0.00%	EITI, “Extractive Industries Transparency Initiative Reports	When ranges are provided, the average is presented here.
Exaco (Exploitation artisanale du Congo)	Cobalt concentrate	Percentage of cobalt content										7.00%	7.00%	7.00%	7.00%	0.00%	0.00%	0.00%	0.00%	0.00%	0.00%	0.00%	0.00%	EITI, “Extractive Industries Transparency Initiative Reports	When ranges are provided, the average is presented here.
FEZA	Cobalt concentrate	Percentage of cobalt content										0.00%	0.00%	0.00%	0.00%	0.00%	0.00%	9.56%	9.56%	0.00%	0.00%	0.00%	0.00%	EITI, “Extractive Industries Transparency Initiative Reports	When ranges are provided, the average is presented here.
Huachin Mining (CNMC)	Cobalt concentrate	Percentage of cobalt content										0.00%	0.00%	0.00%	0.00%	0.00%	9.00%	10.00%	0.00%	0.00%	0.00%	0.00%	0.00%	EITI, “Extractive Industries Transparency Initiative Reports	When ranges are provided, the average is presented here.
KMP (Katanga Minerals Processing)	Cobalt concentrate	Percentage of cobalt content										0.00%	7.04%	0.00%	0.00%	0.00%	0.00%	0.00%	0.00%	0.00%	0.00%	0.00%	0.00%	EITI, “Extractive Industries Transparency Initiative Reports	When ranges are provided, the average is presented here.
Metal Mines (MTM)	Cobalt concentrate	Percentage of cobalt content										6.45%	6.45%	6.45%	6.45%	6.45%	9.00%	10.00%	10.00%	10.00%	10.00%	10.00%	10.00%	EITI, “Extractive Industries Transparency Initiative Reports	When ranges are provided, the average is presented here.
MJM (Jiana Energy, formerly Macrolink Jiayuan Mining SARL)	Cobalt concentrate	Percentage of cobalt content										0.00%	6.80%	6.80%	6.80%	6.80%	11.00%	11.00%	11.00%	11.00%	11.00%	0.00%	0.00%	EITI, “Extractive Industries Transparency Initiative Reports	When ranges are provided, the average is presented here.
MuMi (Mutanda) Export	Cobalt concentrate	Percentage of cobalt content										9.50%	9.50%	0.00%	0.00%	0.00%	0.00%	0.00%	0.00%	0.00%	0.00%	0.00%	0.00%	EITI, “Extractive Industries Transparency Initiative Reports	When ranges are provided, the average is presented here.
ROQ Mining	Cobalt concentrate	Percentage of cobalt content										0.00%	6.45%	0.00%	0.00%	0.00%	0.00%	0.00%	0.00%	0.00%	0.00%	0.00%	0.00%	EITI, “Extractive Industries Transparency Initiative Reports	When ranges are provided, the average is presented here.
Rubamin	Cobalt concentrate	Percentage of cobalt content										6.45%	6.45%	6.45%	0.00%	0.00%	0.00%	0.00%	0.00%	0.00%	0.00%	0.00%	0.00%	EITI, “Extractive Industries Transparency Initiative Reports	When ranges are provided, the average is presented here.
SARDC	Cobalt concentrate	Percentage of cobalt content										6.45%	0.00%	0.00%	0.00%	0.00%	0.00%	0.00%	0.00%	0.00%	0.00%	0.00%	0.00%	EITI, “Extractive Industries Transparency Initiative Reports	When ranges are provided, the average is presented here.
SODIMINCO	Cobalt concentrate	Percentage of cobalt content										0.00%	0.00%	0.00%	0.00%	5.34%	0.00%	0.00%	0.00%	0.00%	0.00%	0.00%	0.00%	EITI, “Extractive Industries Transparency Initiative Reports	When ranges are provided, the average is presented here.
SOMIKA	Cobalt concentrate	Percentage of cobalt content										6.10%	6.10%	6.10%	6.10%	9.50%	9.50%	9.50%	0.00%	0.00%	0.00%	0.00%	0.00%	EITI, “Extractive Industries Transparency Initiative Reports	When ranges are provided, the average is presented here.
Tengyuan Co&Cu Resources (TCC SARL)	Cobalt concentrate	Percentage of cobalt content										0.00%	0.00%	0.00%	0.00%	0.00%	0.00%	0.00%	0.00%	0.00%	10.08%	0.00%	0.00%	EITI, “Extractive Industries Transparency Initiative Reports	When ranges are provided, the average is presented here.
Thomas Mining SARL	Cobalt concentrate	Percentage of cobalt content										0.00%	0.00%	0.00%	0.00%	0.00%	0.00%	0.00%	0.00%	0.00%	9.80%	0.00%	0.00%	EITI, “Extractive Industries Transparency Initiative Reports	When ranges are provided, the average is presented here.
Volcano	Cobalt concentrate	Percentage of cobalt content										6.80%	6.80%	6.80%	6.80%	6.80%	0.00%	0.00%	0.00%	0.00%	0.00%	0.00%	0.00%	EITI, “Extractive Industries Transparency Initiative Reports	When ranges are provided, the average is presented here.
Bazano	Copper cobalt concentrate	Percentage of cobalt content										0.00%	0.00%	10.00%	10.00%	0.00%	0.00%	0.00%	0.00%	0.00%	0.00%	0.00%	0.00%	EITI, “Extractive Industries Transparency Initiative Reports	When ranges are provided, the average is presented here.
Boss	Copper cobalt concentrate	Percentage of cobalt content										9.14%	9.14%	9.14%	9.14%	9.14%	9.14%	9.14%	9.14%	9.14%	9.14%	0.00%	0.00%	EITI, “Extractive Industries Transparency Initiative Reports	When ranges are provided, the average is presented here.
CDM	Copper cobalt concentrate	Percentage of cobalt content										0.00%	0.00%	0.00%	0.00%	0.00%	0.00%	0.00%	0.00%	0.00%	0.00%	8.50%	8.50%	EITI, “Extractive Industries Transparency Initiative Reports	When ranges are provided, the average is presented here.
CMSK (Compagnie Minière du Sud Katanga)	Copper cobalt concentrate	Percentage of cobalt content										1.20%	1.20%	1.20%	1.20%	1.20%	5.60%	0.00%	0.00%	0.00%	0.00%	0.00%	0.00%	EITI, “Extractive Industries Transparency Initiative Reports	When ranges are provided, the average is presented here.
COMIKA	Copper cobalt concentrate	Percentage of cobalt content										0.00%	0.00%	0.00%	0.00%	0.00%	0.00%	0.00%	5.53%	5.53%	5.53%	5.53%	0.00%	EITI, “Extractive Industries Transparency Initiative Reports	When ranges are provided, the average is presented here.
DCP (Democratic Republic of Congo Copper and Cobalt Project)	Copper cobalt concentrate	Percentage of cobalt content										1.20%	1.20%	0.00%	0.00%	0.00%	0.00%	0.00%	0.00%	0.00%	0.00%	0.00%	0.00%	EITI, “Extractive Industries Transparency Initiative Reports	When ranges are provided, the average is presented here.
KCC (Kamoto Copper Company)	Copper cobalt concentrate	Percentage of cobalt content										0.00%	3.85%	6.51%	6.78%	5.17%	0.00%	0.00%	0.00%	0.00%	0.00%	0.00%	0.00%	EITI, “Extractive Industries Transparency Initiative Reports	When ranges are provided, the average is presented here.
MuMi (Mutanda)	Copper cobalt concentrate	Percentage of cobalt content										3.85%	3.85%	6.51%	6.78%	5.17%	7.37%	0.00%	0.00%	0.00%	0.00%	0.00%	0.00%	EITI, “Extractive Industries Transparency Initiative Reports	When ranges are provided, the average is presented here.
SICOMINES	Copper cobalt concentrate	Percentage of cobalt content										0.00%	0.00%	0.00%	0.00%	0.00%	0.00%	5.53%	0.00%	0.00%	0.00%	0.00%	0.00%	EITI, “Extractive Industries Transparency Initiative Reports	When ranges are provided, the average is presented here.
SODIMINCO	Copper cobalt concentrate	Percentage of cobalt content										0.00%	0.00%	0.00%	0.00%	5.34%	0.00%	0.00%	0.00%	0.00%	0.00%	0.00%	0.00%	EITI, “Extractive Industries Transparency Initiative Reports	When ranges are provided, the average is presented here.
**Average cobalt content of cobalt concentrate**												**7.1%**	**7.0%**	**6.9%**	**7.0%**	**7.3%**	**9.0%**	**9.6%**	**9.6%**	**9.6%**	**10.1%**	**10.0%**	**10.0%**		Calculated by averaging estimates presented above.
**Average cobalt content of artisanal cobalt concentrate**												**6.6%**	**6.6%**	**6.6%**	**6.6%**	**6.9%**	**9.0%**	**9.6%**	**9.6%**	**9.6%**	**10.2%**	**10.0%**	**10.0%**		Calculated by averaging estimates presented above.
**Average cobalt content of copper/cobalt concentrate**												**3.8%**	**3.8%**	**6.5%**	**6.8%**	**5.2%**	**7.4%**	**7.3%**	**7.3%**	**7.3%**	**7.3%**	**5.5%**	**8.5%**		Calculated by averaging estimates presented above.
**Average cobalt content of artisanal copper/cobalt concentrate**												**1%**	**1%**	**10%**	**10%**	**5%**	**N/A**	**N/A**	**N/A**	**N/A**	**N/A**	**9%**	**9%**		Calculated by averaging estimates presented above.

9c. Cobalt content of exports by company (metric tons, cobalt content)

Company	Material	Measurement	2000*	2001*	2002*	2003*	2004*	2005*	2006*	2007*	2008*	2009	2010	2011	2012	2013	2014	2015	2016	2017	2018	2019	2020	References	Notes
Bazano	Cobalt concentrate	Metric tons of cobalt content										978	1,818	2,970	493	189	-	-	-	-	-	-	-		Calculated by multiplying gross weight (8A) and cobalt content (8B).
Bolfast	Cobalt concentrate	Metric tons of cobalt content										559	1,219	1,424	673	408	465	722	169	18	-	-	-		Calculated by multiplying gross weight (8A) and cobalt content (8B).
Boss	Cobalt concentrate	Metric tons of cobalt content										4,248	4,770	4,438	2,002	2,508	315	2,939	745	4,326	3,291	-	-		Calculated by multiplying gross weight (8A) and cobalt content (8B).
CDM	Cobalt concentrate	Metric tons of cobalt content										896	1,143	1,548	1,865	1,568	5,318	4,429	276	-	-	-	-		Calculated by multiplying gross weight (8A) and cobalt content (8B).
Cota Mining	Cobalt concentrate	Metric tons of cobalt content										78	36	-	-	-	-	-	-	-	-	-	-		Calculated by multiplying gross weight (8A) and cobalt content (8B).
Eagle Group	Cobalt concentrate	Metric tons of cobalt content										-	-	-	-	325	38	-	-	-	-	-	-		Calculated by multiplying gross weight (8A) and cobalt content (8B).
Exaco (Exploitation artisanale du Congo)	Cobalt concentrate	Metric tons of cobalt content										818	720	198	77	-	-	-	-	-	-	-	-		Calculated by multiplying gross weight (8A) and cobalt content (8B).
FEZA	Cobalt concentrate	Metric tons of cobalt content										-	-	-	-	-	-	1,077	115	-	-	-	-		Calculated by multiplying gross weight (8A) and cobalt content (8B).
Huachin Mining (CNMC)	Cobalt concentrate	Metric tons of cobalt content										-	-	-	-	-	218	1,158	-	-	-	-	-		Calculated by multiplying gross weight (8A) and cobalt content (8B).
KMP (Katanga Minerals Processing)	Cobalt concentrate	Metric tons of cobalt content										-	130	-	-	-	-	-	-	-	-	-	-		Calculated by multiplying gross weight (8A) and cobalt content (8B).
Metal Mines (MTM)	Cobalt concentrate	Metric tons of cobalt content										969	824	372	598	922	2,464	3,260	1,581	1,973	1,480	16	-		Calculated by multiplying gross weight (8A) and cobalt content (8B).
MJM (Jiana Energy, formerly Macrolink Jiayuan Mining SARL)	Cobalt concentrate	Metric tons of cobalt content										-	41	126	-	156	594	483	1,565	3,397	682	-	-		Calculated by multiplying gross weight (8A) and cobalt content (8B).
MuMi (Mutanda) Export	Cobalt concentrate	Metric tons of cobalt content										9,517	554	-	-	-	-	-	-	-	-	-	-		Calculated by multiplying gross weight (8A) and cobalt content (8B).
ROQ Mining	Cobalt concentrate	Metric tons of cobalt content										-	29	-	-	-	-	-	-	-	-	-	-		Calculated by multiplying gross weight (8A) and cobalt content (8B).
Rubamin	Cobalt concentrate	Metric tons of cobalt content										268	468	42	-	-	-	-	-	-	-	-	-		Calculated by multiplying gross weight (8A) and cobalt content (8B).
SARDC	Cobalt concentrate	Metric tons of cobalt content										55	-	-	-	-	-	-	-	-	-	-	-		Calculated by multiplying gross weight (8A) and cobalt content (8B).
SODIMINCO	Cobalt concentrate	Metric tons of cobalt content										-	-	-	-	58	-	-	-	-	-	-	-		Calculated by multiplying gross weight (8A) and cobalt content (8B).
SOMIKA	Cobalt concentrate	Metric tons of cobalt content										1,509	1,484	982	520	90	302	114	-	-	-	-	-		Calculated by multiplying gross weight (8A) and cobalt content (8B).
Tengyuan Co&Cu Resources (TCC SARL)	Cobalt concentrate	Metric tons of cobalt content										-	-	-	-	-	-	-	-	-	212	-	-		Calculated by multiplying gross weight (8A) and cobalt content (8B).
Thomas Mining SARL	Cobalt concentrate	Metric tons of cobalt content										-	-	-	-	-	-	-	-	-	1,734	-	-		Calculated by multiplying gross weight (8A) and cobalt content (8B).
Volcano	Cobalt concentrate	Metric tons of cobalt content										888	2,401	2,391	1,116	31	-	-	-	-	-	-	-		Calculated by multiplying gross weight (8A) and cobalt content (8B).
Bazano	Copper cobalt concentrate	Metric tons of cobalt content										-	-	714	126	-	-	-	-	-	-	-	-		Calculated by multiplying gross weight (8A) and cobalt content (8B).
Boss	Copper cobalt concentrate	Metric tons of cobalt content										-	-	4,856	5,435	5,000	6,317	2,997	-	-	-	-	-		Calculated by multiplying gross weight (8A) and cobalt content (8B).
Boss	Copper cobalt concentrate	Metric tons of cobalt content										1,972	4,589	510	2,560	3,829	4,415	1,351	5,376	460	212	-	-		Calculated by multiplying gross weight (8A) and cobalt content (8B).
CDM	Copper cobalt concentrate	Metric tons of cobalt content										-	-	-	-	-	-	-	-	-	-	2,743	1,793		Calculated by multiplying gross weight (8A) and cobalt content (8B).
CMSK (Compagnie Minière du Sud Katanga)	Copper cobalt concentrate	Metric tons of cobalt content										78	551	489	-	512	2,333	-	-	-	-	-	-		Calculated by multiplying gross weight (8A) and cobalt content (8B).
COMIKA	Copper cobalt concentrate	Metric tons of cobalt content										-	-	-	-	-	-	-	1,715	2,534	2,553	1,448	-		Calculated by multiplying gross weight (8A) and cobalt content (8B).
DCP (Democratic Republic of Congo Copper and Cobalt Project)	Copper cobalt concentrate	Metric tons of cobalt content										155	52	-	-	-	-	-	-	-	-	-	-		Calculated by multiplying gross weight (8A) and cobalt content (8B).
KCC (Kamoto Copper Company)	Copper cobalt concentrate	Metric tons of cobalt content										-	734	5,560	6,701	6,976	-	-	-	-	-	-	-		Calculated by multiplying gross weight (8A) and cobalt content (8B).
MuMi (Mutanda)	Copper cobalt concentrate	Metric tons of cobalt content										745	5,008	5,302	2,619	1,041	807	-	-	-	-	-	-		Calculated by multiplying gross weight (8A) and cobalt content (8B).
SICOMINES	Copper cobalt concentrate	Metric tons of cobalt content										-	-	-	-	-	-	85	-	-	-	-	-		Calculated by multiplying gross weight (8A) and cobalt content (8B).
SODIMINCO	Copper cobalt concentrate	Metric tons of cobalt content										-	-	-	-	52	-	-	-	-	-	-	-		Calculated by multiplying gross weight (8A) and cobalt content (8B).
**Total cobalt content of ore/concentrates reported by DRC Ministry of Mines provincial divisions**												**23,733**	**26,572**	**31,923**	**24,784**	**23,666**	**23,585**	**18,617**	**11,542**	**12,708**	**10,164**	**4,207**	**1,793**		

9d: Exports by company's predominant production type (Artisanal [including small-scale], artisanal processing, or industrial)

Company	Material	Measurement	2000*	2001*	2002*	2003*	2004*	2005*	2006*	2007*	2008*	2009	2010	2011	2012	2013	2014	2015	2016	2017	2018	2019	2020	Referencess	Notes
Bazano	Cobalt concentrate	Type of production										Artisanal processing	Artisanal processing	Artisanal processing	Artisanal processing	Artisanal processing	-	-	-	-	-	-	-	Global Witness, “Rush and ruin: The devastating mineral tr	The company is reported to use artisanal/small-scale means of extraction at their own mines, as well as periodically processing third pary ore.
Bolfast	Cobalt concentrate	Type of production										Artisanal	Artisanal	Artisanal	Artisanal	Artisanal	Artisanal	Artisanal	Artisanal	Artisanal	-	-	-	Congomines.org, https://congomines.org/drc_companies/	The presence of exports, but lack of data on the company indicates it is sourcing from artisanally produced cobalt ores and concentrates. Darton includes this as a small, independent company in the 'others' group.
Boss	Cobalt concentrate	Type of production										Industrial	Industrial	Industrial	Industrial	Industrial	Industrial	Industrial	Industrial	Industrial	Industrial	-	-	K. B. Shedd, “Cobalt” in Minerals Yearbook, (US Geological	-
CDM	Cobalt concentrate	Type of production										Artisanal processing	Artisanal processing	Artisanal processing	Artisanal processing	Artisanal processing	Artisanal processing	Artisanal processing	Artisanal processing	Artisanal processing	-	-	-	Darton Commodities Ltd. Annual Cobalt Market Review	Through 2013, the company is reported to have their own mines, which are reported to be extracted via artisanal/small-scale means.
Cota Mining	Cobalt concentrate	Type of production										Artisanal	Artisanal	-	-	-	-	-	-	-	-	-	-	Congomines.org, https://congomines.org/drc_companies/	The presence of exports, but lack of data on the company indicates it is sourcing from artisanally produced cobalt ores and concentrates. Darton includes this as a small, independent company in the 'others' group.
Eagle Group	Cobalt concentrate	Type of production										-	-	-	-	Artisanal	Artisanal	-	-	-	-	-	-	Darton Commodities Ltd. Annual Cobalt Market Review	The presence of exports, but lack of data on the company indicates it is sourcing from artisanally produced cobalt ores and concentrates. Darton includes this as a small, independent company in the 'others' group.
Exaco (Exploitation artisanale du Congo)	Cobalt concentrate	Type of production										Artisanal	Artisanal	Artisanal	Artisanal	-	-	-	-	-	-	-	-	Darton Commodities Ltd. Annual Cobalt Market Review	The presence of exports, but lack of data on the company indicates it is sourcing from artisanally produced cobalt ores and concentrates. Darton includes this as a small, independent company in the 'others' group.
FEZA	Cobalt concentrate	Type of production										-	-	-	-	-	-	Artisanal	Artisanal	-	-	-	-	Darton Commodities Ltd. Annual Cobalt Market Review	-
Huachin Mining (CNMC)	Cobalt concentrate	Type of production										-	-	-	-	-	Industrial	Industrial	-	-	-	-	-	K. B. Shedd, “Cobalt” in Minerals Yearbook, (US Geological	China Nonferrous Metals Company
KMP (Katanga Minerals Processing)	Cobalt concentrate	Type of production										-	Artisanal	-	-	-	-	-	-	-	-	-	-		Produces concentrates from ores, but no evidence it owns its own mines.
Metal Mines (MTM)	Cobalt concentrate	Type of production										Industrial	Industrial	Industrial	Industrial	Industrial	Industrial	Industrial	Industrial	Industrial	Industrial	Industrial	-	Darton Commodities Ltd. Annual Cobalt Market Review	-
MJM (Jiana Energy, formerly Macrolink Jiayuan Mining SARL)	Cobalt concentrate	Type of production										-	Artisanal	Artisanal	Artisanal	Artisanal	Artisanal	Artisanal	Artisanal	Artisanal	Artisanal	-	-	Darton Commodities Ltd. Annual Cobalt Market Review	The company is reported to use artisanal/small-scale means of extraction at their own mines, as well as periodically processing third pary ore.
MuMi (Mutanda) Export	Cobalt concentrate	Type of production										Industrial	Industrial	-	-	-	-	-	-	-	-	-	-	K. B. Shedd, “Cobalt” in Minerals Yearbook, (US Geological	-
ROQ Mining	Cobalt concentrate	Type of production										-	Artisanal	-	-	-	-	-	-	-	-	-	-		Produces concentrates from ores, but no evidence it owns its own mines.
Rubamin	Cobalt concentrate	Type of production										Industrial	Industrial	Industrial	-	-	-	-	-	-	-	-	-	Congomines.org, https://congomines.org/drc_companies/	Congomines.org indicates that Rubamin has exploitation permits on concessions in the copper-cobalt belt. It is worth noting that the low level of production/exports would typically indicate artisanal (including small-scale) production.
SARDC	Cobalt concentrate	Type of production										Artisanal	-	-	-	-	-	-	-	-	-	-	-		Produces concentrates from ores, but no evidence it owns its own mines.
SODIMINCO	Cobalt concentrate	Type of production										-	-	-	-	Artisanal	-	-	-	-	-	-	-		Produces concentrates from ores, but no evidence it owns its own mines.
SOMIKA	Cobalt concentrate	Type of production										Artisanal processing	Artisanal processing	Artisanal processing	Artisanal processing	Artisanal processing	Artisanal processing	Artisanal processing	Artisanal processing	Artisanal processing	Artisanal processing	Artisanal processing	Artisanal processing	2000-2004 Global Witness 2004 (Rush and ruin: The devas	-
Tengyuan Co&Cu Resources (TCC SARL)	Cobalt concentrate	Type of production										-	-	-	-	-	-	-	-	-	Artisanal processing	-	-	Darton Commodities Ltd. Annual Cobalt Market Review 20	-
Thomas Mining SARL	Cobalt concentrate	Type of production										-	-	-	-	-	-	-	-	-	Industrial	-	-	http://www.zijinmining.com/upload/file/2020/09/14/4d3	-
Volcano	Cobalt concentrate	Type of production										Artisanal	Artisanal	Artisanal	Artisanal	Artisanal	-	-	-	-	-	-	-	Congomines.org, https://congomines.org/drc_companies/	The presence of exports, but lack of data on the company indicates it is sourcing from artisanally produced cobalt ores and concentrates. Darton includes this as a small, independent company in the 'others' group.
Bazano	Copper cobalt concentrate	Type of production										-	-	Artisanal	Artisanal	-	-	-	-	-	-	-	-	Global Witness, “Rush and ruin: The devastating mineral tr	The company is reported to use artisanal/small-scale means of extraction at their own mines, as well as periodically processing third pary ore.
Boss	Copper cobalt concentrate	Type of production										Industrial	Industrial	Industrial	Industrial	Industrial	Industrial	Industrial	Industrial	Industrial	Industrial	-	-	K. B. Shedd, “Cobalt” in Minerals Yearbook, (US Geological	-
Boss	Copper cobalt concentrate	Type of production										Industrial	Industrial	Industrial	Industrial	Industrial	Industrial	Industrial	Industrial	Industrial	Industrial	-	-	K. B. Shedd, “Cobalt” in Minerals Yearbook, (US Geological	-
CDM	Copper cobalt concentrate	Type of production										-	-	-	-	-	-	-	-	-	-	Artisanal processing	Artisanal processing	K. B. Shedd, “Cobalt” in Minerals Yearbook, (US Geological	Through 2013, the company is reported to have their own mines, which are reported to be extracted via artisanal/small-scale means.
CMSK (Compagnie Minière du Sud Katanga)	Copper cobalt concentrate	Type of production										Industrial	Industrial	Industrial	Industrial	Industrial	Industrial	-	-	-	-	-	-	K. B. Shedd, “Cobalt” in Minerals Yearbook, (US Geological	-
COMIKA	Copper cobalt concentrate	Type of production										-	-	-	-	-	-	-	Industrial	Industrial	Industrial	Industrial	-	K. B. Shedd, “Cobalt” in Minerals Yearbook, (US Geological	-
DCP (Democratic Republic of Congo Copper and Cobalt Project)	Copper cobalt concentrate	Type of production										Artisanal	Artisanal	-	-	-	-	-	-	-	-	-	-		Produces concentrates from ores, but no evidence it owns its own mines.
KCC (Kamoto Copper Company)	Copper cobalt concentrate	Type of production										-	Industrial	Industrial	Industrial	Industrial	Industrial	Industrial	Industrial	Industrial	-	-	-	K. B. Shedd, “Cobalt” in Minerals Yearbook, (US Geological	-
MuMi (Mutanda)	Copper cobalt concentrate	Type of production										Industrial	Industrial	Industrial	Industrial	Industrial	Industrial	-	-	-	-	-	-	K. B. Shedd, “Cobalt” in Minerals Yearbook, (US Geological	-
SICOMINES	Copper cobalt concentrate	Type of production										-	-	-	-	-	-	Industrial	-	-	-	-	-	K. B. Shedd, “Cobalt” in Minerals Yearbook, (US Geological	-
SODIMINCO	Copper cobalt concentrate	Type of production										-	-	-	-	Artisanal	-	-	-	-	-	-	-	-	Produces concentrates from ores, but no evidence it owns its own mines.

9e: Exports by destination (country)

Company	Material	Measurement	2000*	2001*	2002*	2003*	2004*	2005*	2006*	2007*	2008*	2009	2010	2011	2012	2013	2014	2015	2016	2017	2018	2019	2020	References	Notes
Bazano	Cobalt concentrate	Type of production										Zambia	Zambia	Zambia	Zambia	Zambia									
Bolfast	Cobalt concentrate	Type of production										China	China	China	China	China	China	China	China	China					
Boss	Cobalt concentrate	Type of production										China	China	China	China	China	China	China	China	Zambia	Zambia				
CDM	Cobalt concentrate	Type of production										China	China	China	China	China	China	China	China	China					
Cota Mining	Cobalt concentrate	Type of production										Unknown	Unknown												
Eagle Group	Cobalt concentrate	Type of production														Unknown	Unknown								
Exaco (Exploitation artisanale du Congo)	Cobalt concentrate	Type of production										Unknown	Unknown	Unknown	Unknown										
FEZA	Cobalt concentrate	Type of production																China	China						
Huachin Mining (CNMC)	Cobalt concentrate	Type of production															China	China							
KMP (Katanga Minerals Processing)	Cobalt concentrate	Type of production											Unknown												
Metal Mines (MTM)	Cobalt concentrate	Type of production										China	China	China	China	China	China	China	China	China	China	China			
MJM (Jiana Energy, formerly Macrolink Jiayuan Mining SARL)	Cobalt concentrate	Type of production											China	China	China	China	China	China	China	China	China				
MuMi (Mutanda) Export	Cobalt concentrate	Type of production										China	China												
ROQ Mining	Cobalt concentrate	Type of production											Unknown												
Rubamin	Cobalt concentrate	Type of production										India	India	India											
SARDC	Cobalt concentrate	Type of production										Unknown													
SODIMINCO	Cobalt concentrate	Type of production														Unknown									
SOMIKA	Cobalt concentrate	Type of production										China	China	China	China	China	China	China	China						
Tengyuan Co&Cu Resources (TCC SARL)	Cobalt concentrate	Type of production																							
Thomas Mining SARL	Cobalt concentrate	Type of production																			China				
Volcano	Cobalt concentrate	Type of production										China	China	China	China	China									
Bazano	Copper cobalt concentrate	Type of production												China	China										
Boss	Copper cobalt concentrate	Type of production												Zambia	Zambia	Zambia	Zambia	Zambia							

Boss	Copper cobalt concentrate	Gross weight										China	China	China	China	China	China	China	China	Zambia	Zambia				

9f: DRC cobalt ore/concentrate exports to the world by production type (metric tons, cobalt content)

			2000*	2001*	2002*	2003*	2004*	2005*	2006*	2007*	2008*	2009	2010	2011	2012	2013	2014	2015	2016	2017	2018	2019	2020	References	Notes
**Industrial exports to the world**												**17,800**	**17,500**	**21,600**	**19,900**	**20,800**	**16,900**	**11,800**	**9,400**	**9,300**	**9,300**	**1,500**	**-**	-	Sum of reported exports from industrial mining companies.
**Reported artisanal exports to the world**												**2,600**	**4,600**	**4,900**	**2,000**	**1,000**	**1,100**	**2,300**	**1,800**	**3,400**	**700**	**-**	**-**	-	Sum of reported exports from companies sourcing material from artisanal miners.
**Reported artisanal processing exports to the world**												**3,400**	**4,400**	**5,500**	**2,900**	**1,800**	**5,600**	**4,500**	**300**	**-**	**200**	**2,700**	**1,800**	-	Sum of reported exports from companies processing ores from artisanal miners into concentrates.
**Total DRC Ministry of Mines exports to World**												**23,800**	**26,500**	**32,000**	**24,800**	**23,600**	**23,600**	**18,600**	**11,500**	**12,700**	**10,200**	**4,200**	**1,800**	-	-

9g: DRC cobalt ore/concentrate exports to China by production type (metric tons, cobalt content)

			2000*	2001*	2002*	2003*	2004*	2005*	2006*	2007*	2008*	2009	2010	2011	2012	2013	2014	2015	2016	2017	2018	2019	2020	References	Notes
**Industrial exports to China**												**17,500**	**16,500**	**16,200**	**7,800**	**8,300**	**8,200**	**8,800**	**9,400**	**4,500**	**5,800**	**1,500**	**-**		Sum of reported exports to China from industrial mining companies.
**Reported artisanal exports to China**												**1,400**	**3,700**	**4,700**	**1,900**	**600**	**1,100**	**2,300**	**1,800**	**3,400**	**700**	**-**	**-**		Sum of reported exports to China from companies sourcing material from artisanal miners.
**Reported artisanal processing exports to the China**												**2,400**	**2,600**	**2,500**	**2,400**	**1,700**	**5,600**	**4,500**	**300**	**-**	**-**	**2,700**	**1,800**		Sum of reported exports to China from companies processing ores from artisanal miners into concentrates.
**Total DRC Ministry of Mines exports to China**												**21,300**	**22,800**	**23,400**	**12,100**	**10,600**	**14,900**	**15,600**	**11,500**	**7,900**	**6,500**	**4,200**	**1,800**		0

9h: Calculation of "artisanal imports" to estimate the second component of artisanal-production-B (i.e., artisanal processing plus imports) (metric tons, cobalt content)

			2000*	2001*	2002*	2003*	2004*	2005*	2006*	2007*	2008*	2009	2010	2011	2012	2013	2014	2015	2016	2017	2018	2019	2020	References	Notes
Total China Customs imports from DRC												20,600	25,500	25,800	13,000	13,400	14,400	17,200	10,900	7,600	10,500	6,800	4,000	-	From Supporting Dataset 6B.
Industrial Chinese imports												17,500	16,500	16,200	7,800	8,300	8,200	8,800	9,400	4,500	5,800	1,500	-	-	From Supporting Dataset 8G.
**Artisanal Chinese imports from the DRC (rounded to the nearest thousand)**												**3,000**	**9,000**	**10,000**	**5,000**	**5,000**	**6,000**	**8,000**	**2,000**	**3,000**	**5,000**	**5,000**	**4,000**	-	These estimates are predominantly larger than the sum of the "Artisanal exports to China" and "Artisanal processing exports to China" due to exports that were not reported (perhaps due to smuggling).
																								-	

9i: Artisanal processing and imports (i.e., artisanal-production-B, results from 2009 through 2020, metric tons, cobalt content)

			2000*	2001*	2002*	2003*	2004*	2005*	2006*	2007*	2008*	2009	2010	2011	2012	2013	2014	2015	2016	2017	2018	2019	2020	References	Notes
Artisanal processing (rounded to nearest thousand)												10,000	10,000	9,000	7,000	6,000	6,000	10,000	7,000	9,000	16,000	11,000	7,000	-	From Supporting Dataset 4B
Artisanal imports (rounded to nearest thousand)												3,000	9,000	10,000	5,000	5,000	6,000	8,000	2,000	3,000	5,000	5,000	4,000	-	From Supporting Dataset 7H
**Artisanal-production-B, also referred to as artisanal processing and imports (results)**												**13,000**	**19,000**	**19,000**	**12,000**	**11,000**	**12,000**	**18,000**	**9,000**	**12,000**	**21,000**	**16,000**	**11,000**	**-**	**Artisanal-production-B results from 2009 through 2020**

Supporting Dataset 10: Summary of results for artisanal processing, total Chinese cobalt ore/concentrate imports, industrial Chinese ore/concentrate imports and artisanal Chinese ore/concentrate imports(metric tons, cobalt content)

		2000	2001	2002	2003	2004	2005	2006	2007	2008	2009	2010	2011	2012	2013	2014	2015	2016	2017	2018	2019	2020				Notes
Artisanal processing		-	-	1,000	1,000	1,000	1,000	2,000	2,000	3,000	10,000	10,000	9,000	7,000	6,000	6,000	10,000	7,000	9,000	16,000	11,000	7,000				From Supporting Dataset 4B.
Total Chinese cobalt ore/concentrate imports from the DRC		1,000	1,400	2,300	5,600	9,800	13,900	11,700	8,000	18,500	20,600	25,500	25,800	13,000	13,400	14,400	17,200	10,900	7,600	10,500	6,800	4,000				From Supporting Dataset 5C.
Industrial Chinese ore/concentrate imports from DRC		-	-	100	2,200	3,300	4,400	4,500	-	4,800	17,500	16,500	16,200	7,800	8,300	8,200	8,800	9,400	4,500	5,800	1,500	-				The years from 2000 through 2008 are from Supporting Dataset 4C. The years from 2008 through 2020 are from Supporting Dataset 11G.
Artisanal Chinese imports from the DRC		1,000	1,000	2,000	3,000	7,000	10,000	7,000	8,000	14,000	3,000	9,000	10,000	5,000	5,000	6,000	8,000	2,000	3,000	5,000	5,000	4,000				The years from 2000 through 2008 are from Supporting Dataset 10a. The years from 2008 through 2020 are from Supporting Dataset 11h.
Artisanal-production-B, also referred to as Artisanal processing and imports		1,000	1,000	3,000	4,000	8,000	11,000	9,000	10,000	17,000	13,000	19,000	19,000	12,000	11,000	12,000	18,000	9,000	12,000	21,000	16,000	11,000				Artisanal production estimated as the sum of artisanal processing and artisanal imports.

Supporting Dataset 11: Results of artisanal cobalt production accounting methods (metric tons, cobalt content) and comparison to artisanal cobalt production estimates from the literature (metric tons, cobalt content), and price (2020$/lb)

11a: Cobalt price (2020$/lb), Artisanal-production-A (metric tons, cobalt content), and artisanal-production-B (metric tons, cobalt content)

	Reporter Type	2000	2001	2002	2003	2004	2005	2006	2007	2008	2009	2010	2011	2012	2013	2014	2015	2016	2017	2018	2019	2020				Notes
Cobalt price (2020$/lb, rounded to the nearest dollar)		$ 22	$ 15	$ 10	$ 15	$ 32	$ 20	$ 20	$ 34	$ 40	$ 20	$ 22	$ 17	$ 14	$ 12	$ 14	$ 14	$ 13	$ 27	$ 36	$ 16	$ 16				
Artisanal-production-A, also referred to as Nonindustrial (i.e., artisanal) production	Results	2,000	3,000	6,000	5,000	9,000	12,000	10,000	10,000	13,000	11,000	16,000	15,000	11,000	6,000	12,000	17,000	9,000	15,000	17,000	13,000	9,000				From Supporting Dataset 3.
Artisanal-production-B, also referred to as Artisanal processing and imports	Results	1,000	1,000	3,000	4,000	8,000	11,000	9,000	10,000	17,000	13,000	19,000	19,000	12,000	11,000	12,000	18,000	9,000	12,000	21,000	16,000	11,000				The years 2000 through 2008 are from Supporting Dataset 9b. The years 2009 through 2020 are from Supporting Dataset 10I.

11b: Artisanal-production-A, artisanal-production-B, and reported literature estimates as a percentage of total DRC cobalt mine production (%)

	Reporter Type	2000	2001	2002	2003	2004	2005	2006	2007	2008	2009	2010	2011	2012	2013	2014	2015	2016	2017	2018	2019	2020				Notes
Total DRC mine production (rounded to nearest 100)		11,000	12,000	14,600	14,800	20,200	24,500	27,100	25,400	32,300	40,000	60,000	59,000	52,000	56,000	65,000	72,000	68,000	80,000	104,000	107,000	98,200				
Artisanal-production-A share of total DRC cobalt mine production	Results	18%	25%	41%	34%	45%	49%	37%	39%	40%	28%	27%	25%	21%	11%	18%	24%	13%	19%	16%	12%	9%				Artisanal-production-A divided by total DRC mine production
Artisanal-production-B share of total DRC cobalt mine production	Results	9%	8%	21%	27%	40%	45%	33%	39%	53%	33%	32%	32%	23%	20%	18%	25%	13%	15%	20%	15%	11%				Artisanal-production-B divided by total DRC mine production
Government reported estimates share of total DRC cobalt mine production	Government	9%	17%	14%	27%	35%	29%	N/A	N/A	N/A	18%	13%	14%	13%	13%	12%	14%	15%	15%	17%	12%	N/A				Government reported estimates (from Dataset S1) divided by total DRC cobalt mine production
Industry average reported estimates share of total DRC cobalt mine production	Industry	N/A	N/A	N/A	N/A	N/A	N/A	N/A	N/A	N/A	28%	30%	29%	15%	9%	11%	15%	12%	19%	23%	9%	9%				Average of industry reported estimates (from Dataset S1) divided by total DRC cobalt mine production
NGO average reported estimates share of total DRC cobalt mine production	Non-governmental organization (NGO)	73%	75%	75%	74%	94%	73%	74%	75%	74%	N/A	N/A	N/A	N/A	N/A	N/A	N/A	N/A	24%	24%	24%	32%				Average of non-governmental organization reported estimates (from Dataset S1) divided by total DRC cobalt mine production

11c: Artisanal-production-A, artisanal-production-B, and reported literature estimates as a percentage of total world cobalt mine production (%)

		2000	2001	2002	2003	2004	2005	2006	2007	2008	2009	2010	2011	2012	2013	2014	2015	2016	2017	2018	2019	2020				Notes
Total world mine production (rounded to nearest 1,000)		38,000	45,000	52,000	53,000	60,000	65,000	70,000	69,000	74,000	76,000	105,000	106,000	100,000	103,000	115,000	122,000	115,000	128,000	149,000	155,000	143,000				
Artisanal-production-A share of total world cobalt mine production		5%	7%	12%	9%	15%	18%	14%	14%	18%	14%	15%	14%	11%	6%	10%	14%	8%	12%	11%	8%	6%				Artisanal-production-A divided by total world mine production
Artisanal-production-B share of total world cobalt mine production		3%	2%	6%	8%	13%	17%	13%	14%	23%	17%	18%	18%	12%	11%	10%	15%	8%	9%	14%	10%	8%				Artisanal-production-B divided by total world mine production
Government reported estimates share of total world cobalt mine production		3%	4%	4%	8%	12%	11%	N/A	N/A	N/A	9%	8%	8%	7%	7%	7%	8%	9%	9%	12%	8%	N/A				Government reported estimates (from Dataset S1) divided by total world cobalt mine production
Industry average reported estimates share of total world cobalt mine production		N/A	N/A	N/A	N/A	N/A	N/A	N/A	N/A	N/A	14%	17%	16%	8%	5%	6%	9%	7%	12%	16%	6%	6%				Average of industry reported estimates (from Dataset S1) divided by total world cobalt mine production
NGO average reported estimates share of total world cobalt mine production		21%	20%	21%	21%	32%	28%	29%	28%	32%	N/A	N/A	N/A	N/A	N/A	N/A	N/A	N/A	15%	17%	17%	22%				Average of non-governmental organization reported estimates (from Dataset S1) divided by total world cobalt mine production

11d: Comparison of artisanal-production-A and artisanal-production-B

		2000	2001	2002	2003	2004	2005	2006	2007	2008	2009	2010	2011	2012	2013	2014	2015	2016	2017	2018	2019	2020	Average di	Minimum	Maximum	Notes
Difference between artisanal-production-A and artisanal-production-B (tons per annum or tpa)		(1,000)	(2,000)	(3,000)	(1,000)	(1,000)	(1,000)	(1,000)	-	4,000	2,000	3,000	4,000	1,000	5,000	-	1,000	-	(3,000)	4,000	3,000	2,000	800	(3,000)	5,000	Artisanal-production-B minus artisanal-production-A
Difference between artisanal-production-A and artisanal-production-B (%)		-50%	-67%	-50%	-20%	-11%	-8%	-10%	0%	31%	18%	19%	27%	9%	83%	0%	6%	0%	-20%	24%	23%	22%	1%	0%	67%	(Artisanal-production-B minus artisanal-production-A) divided by artisanal-production-A
Absolute difference between artisanal-production-A and artisanal-production-B (tons per annum or tpa)		1,000	2,000	3,000	1,000	1,000	1,000	1,000	-	4,000	2,000	3,000	4,000	1,000	5,000	-	1,000	-	3,000	4,000	3,000	2,000	2,000	-	5,000	Absolute difference between artisanal-production-B minus artisanal-production-A
Annual change of artisanal-production-A (tons per annum or tpa)			1,000	3,000	(1,000)	4,000	3,000	(2,000)	-	3,000	(2,000)	5,000	(1,000)	(4,000)	(5,000)	6,000	5,000	(8,000)	6,000	2,000	(4,000)	(4,000)				Artisanal-production-A t+1 minus t
Annual change of artisanal-production-B (tons per annum or tpa)			-	2,000	1,000	4,000	3,000	(2,000)	1,000	7,000	(4,000)	6,000	-	(7,000)	(1,000)	1,000	6,000	(9,000)	3,000	9,000	(5,000)	(5,000)				Year t+1 minus year t
Annual change of artisanal-production-A (sign)			+	+	-	+	+	-	-	+	-	+	-	-	-	+	+	-	+	+	-	-				Positive or negative annual change
Annual change of artisanal-production-B (sign)			-	+	+	+	+	-	+	+	-	+	-	-	-	+	+	-	+	+	-	-				Positive or negative annual change
Comparison of sign of annual changes			different	same	different	same	same	same	different	same	same	same	same	same	same	same	same	same	same	same	same	same	Same for 1			

11e: Comparison of artisanal-production-A and government estimates from the literature

		2000	2001	2002	2003	2004	2005	2006	2007	2008	2009	2010	2011	2012	2013	2014	2015	2016	2017	2018	2019	2020	Average di	Minimum	Maximum	Notes
Difference between artisanal-production-A and government estimates (tons per annum or tpa)		(1,000)	(1,000)	(4,000)	(1,000)	(2,000)	(5,000)	(2,000)	N/A	N/A	(4,000)	(8,000)	(7,000)	(4,000)	1,000	(4,000)	(7,000)	1,000	(3,000)	1,000	-	N/A	(2,800)	(8,000)	1,000	Artisanal-production-A minus government estimates (tons per annum or tpa)
Difference between artisanal-production-A and government estimates (%)		-50%	-33%	-67%	-20%	-22%	-42%	-20%	N/A	N/A	-36%	-50%	-47%	-36%	17%	-33%	-41%	11%	-20%	6%	0%	N/A	-27%	-67%	17%	Artisanal-production-A minus government estimates (%)
Absolute difference between artisanal-production-A and government estimates (tons per annum or tpa)		1,000	1,000	4,000	1,000	2,000	5,000	2,000	N/A	N/A	4,000	8,000	7,000	4,000	1,000	4,000	7,000	1,000	3,000	1,000	-	N/A	3,100	-	8,000	Absolute difference (tons per annum or tpa)
Annual change of government estimates (tons per annum or tpa)			1,000	-	2,000	3,000	-	N/A	N/A	N/A	N/A	1,000	-	(1,000)	-	1,000	2,000	-	2,000	6,000	(5,000)	N/A				Year t+1 minus year t
Annual change of government estimates (sign)			+	-	+	+	-	N/A	N/A	N/A	N/A	+	-	-	-	+	+	-	+	+	-	N/A				Positive or negative annual change
Comparison of sign of annual changes between artisanal-production-A and government estimates			same	different	different	same	different	N/A	N/A	N/A	N/A	same	same	same	same	same	same	same	same	same	same	N/A	Same for 1			

11f: Comparison of artisanal-production-A and industry estimates from the literature

		2000	2001	2002	2003	2004	2005	2006	2007	2008	2009	2010	2011	2012	2013	2014	2015	2016	2017	2018	2019	2020	Average di	Minimum	Maximum	Notes
Difference between artisanal-production-A and average industry estimates (tons per annum or tpa)		N/A	N/A	N/A	N/A	N/A	N/A	N/A	N/A	N/A	-	2,000	2,000	(3,000)	(1,000)	(5,000)	(6,000)	(1,000)	-	7,000	(3,000)	-	(700)	(6,000)	7,000	Artisanal-production-A minus average industry estimates (tons per annum or tpa)
Difference between artisanal-production-A and average industry estimates (%)		N/A	N/A	N/A	N/A	N/A	N/A	N/A	N/A	N/A	0%	13%	13%	-27%	-17%	-42%	-35%	-11%	0%	41%	-23%	0%	-7%	-42%	41%	Artisanal-production-A minus average industry estimates (%)
Absolute difference between artisanal-production-A and average industry estimates (tons per annum or tpa)		N/A	N/A	N/A	N/A	N/A	N/A	N/A	N/A	N/A	-	2,000	2,000	3,000	1,000	5,000	6,000	1,000	-	7,000	3,000	-	2,500	-	7,000	Absolute difference (tons per annum or tpa)
Annual change of average industry estimates (tons per annum or tpa)		N/A	N/A	N/A	N/A	N/A	N/A	N/A	N/A	N/A		7,000	(1,000)	(9,000)	(3,000)	2,000	4,000	(3,000)	7,000	9,000	(14,000)	(1,000)				Year t+1 minus year t
Annual change of average industry estimates (sign)		N/A	N/A	N/A	N/A	N/A	N/A	N/A	N/A	N/A		+	-	-	-	+	+	-	+	+	-	-				Positive or negative annual change
Comparison of sign of annual changes between artisanal-production-A and average industry estimates		N/A	N/A	N/A	N/A	N/A	N/A	N/A	N/A	N/A		same	same	same	same	same	same	same	same	same	same	same	Same for 1			

11g: Comparison of artisanal-production-A and non-governmental organization (NGO) estimates from the literature

		2000	2001	2002	2003	2004	2005	2006	2007	2008	2009	2010	2011	2012	2013	2014	2015	2016	2017	2018	2019	2020	Average di	Minimum	Maximum	Notes
Difference between artisanal-production-A and average NGO estimates (tons per annum or tpa)		6,000	6,000	5,000	6,000	10,000	6,000	10,000	9,000	11,000	N/A	N/A	N/A	N/A	N/A	N/A	N/A	N/A	4,000	8,000	13,000	22,000	8,900	4,000	22,000	Artisanal-production-A minus average NGO estimates (tons per annum or tpa)
Difference between artisanal-production-A and average NGO estimates (%)		300%	200%	83%	120%	111%	50%	100%	90%	85%	N/A	N/A	N/A	N/A	N/A	N/A	N/A	N/A	27%	47%	100%	244%	120%	27%	300%	Artisanal-production-A minus NGO estimates (%)
Absolute difference between artisanal-production-A and average NGO estimates (tons per annum or tpa)		6,000	6,000	5,000	6,000	10,000	6,000	10,000	9,000	11,000	N/A	N/A	N/A	N/A	N/A	N/A	N/A	N/A	4,000	8,000	13,000	22,000	8,900	4,000	22,000	Absolute difference (tons per annum or tpa)
Annual change of average NGO estimates (tons per annum or tpa)			1,000	2,000	-	8,000	(1,000)	2,000	(1,000)	5,000	N/A	N/A	N/A	N/A	N/A	N/A	N/A	N/A	N/A	6,000	1,000	5,000				Year t+1 minus year t
Annual change of average NGO estimates (sign)			+	+	-	+	-	+	-	+	N/A	N/A	N/A	N/A	N/A	N/A	N/A	N/A	N/A	+	+	+				Positive or negative annual change
Comparison of sign of annual changes between artisanal-production-A and average NGO estimates			same	same	same	same	different	different	same	same	N/A	N/A	N/A	N/A	N/A	N/A	N/A	N/A	N/A	same	different	different	Same for 7			

11h: Comparison of artisanal-production-B and government estimates from the literature

		2000	2001	2002	2003	2004	2005	2006	2007	2008	2009	2010	2011	2012	2013	2014	2015	2016	2017	2018	2019	2020	Average di	Minimum	Maximum	Notes
Difference between artisanal-production-B and government estimates (tons per annum or tpa)		-	1,000	(1,000)	-	(1,000)	(4,000)	(1,000)	N/A	N/A	(6,000)	(11,000)	(11,000)	(5,000)	(4,000)	(4,000)	(8,000)	1,000	-	(3,000)	(3,000)	N/A	(3,300)	(11,000)	1,000	Artisanal-production-B minus government estimates (tons per annum or tpa)
Difference between artisanal-production-B and government estimates (%)		0%	100%	-33%	0%	-13%	-36%	-11%	N/A	N/A	-46%	-58%	-58%	-42%	-36%	-33%	-44%	11%	0%	-14%	-19%	N/A	-18%	-58%	100%	Artisanal-production-B minus government estimates (%)
Absolute difference between artisanal-production-B and government estimates (tons per annum or tpa)		-	1,000	1,000	-	1,000	4,000	1,000	N/A	N/A	6,000	11,000	11,000	5,000	4,000	4,000	8,000	1,000	-	3,000	3,000	N/A	3,600	-	11,000	
Comparison of sign of annual changes between artisanal-production-B and government estimates			different	different	same	same	different	N/A	N/A	N/A	N/A	same	same	same	same	same	same	same	same	same	same	N/A	Same for 1			

11i: Comparison of artisanal-production-B and industry estimates from the literature

		2000	2001	2002	2003	2004	2005	2006	2007	2008	2009	2010	2011	2012	2013	2014	2015	2016	2017	2018	2019	2020	Average di	Minimum	Maximum	Notes
Difference between artisanal-production-B and industry estimates (tons per annum or tpa)		N/A	N/A	N/A	N/A	N/A	N/A	N/A	N/A	N/A	(2,000)	(1,000)	(2,000)	(4,000)	(6,000)	(5,000)	(7,000)	(1,000)	3,000	3,000	(6,000)	(2,000)	(2,500)	(7,000)	3,000	Artisanal-production-B minus industry estimates (tons per annum or tpa)
Difference between artisanal-production-B and industry estimates (%)		N/A	N/A	N/A	N/A	N/A	N/A	N/A	N/A	N/A	-15%	-5%	-11%	-33%	-55%	-42%	-39%	-11%	25%	14%	-38%	-18%	-19%	-55%	25%	Artisanal-production-B minus industry estimates (%)
Absolute difference between artisanal-production-B and industry estimates (tons per annum or tpa)		N/A	N/A	N/A	N/A	N/A	N/A	N/A	N/A	N/A	2,000	1,000	2,000	4,000	6,000	5,000	7,000	1,000	3,000	3,000	6,000	2,000	3,500	-	7,000	
Comparison of sign of annual changes between artisanal-production-B and industry estimates		N/A	N/A	N/A	N/A	N/A	N/A	N/A	N/A	N/A		same	same	same	same	same	same	same	same	same	same	same	Same for 1			

11j: Comparison of artisanal-production-B and non-governmental organization (NGO) estimates from the literature

		2000	2001	2002	2003	2004	2005	2006	2007	2008	2009	2010	2011	2012	2013	2014	2015	2016	2017	2018	2019	2020	Average di	Minimum	Maximum	Notes
Difference between artisanal-production-B and NGO estimates (tons per annum or tpa)		7,000	8,000	8,000	7,000	11,000	7,000	11,000	9,000	7,000	N/A	N/A	N/A	N/A	N/A	N/A	N/A	N/A	7,000	4,000	10,000	20,000	8,900	4,000	20,000	Artisanal-production-B minus NGO estimates (tons per annum or tpa)
Difference between artisanal-production-B and NGO estimates (%)		700%	800%	267%	175%	138%	64%	122%	90%	41%	N/A	N/A	N/A	N/A	N/A	N/A	N/A	N/A	58%	19%	63%	182%	209%	41%	800%	Artisanal-production-B minus NGO estimates (%)
Absolute difference between artisanal-production-B and NGO estimates (tons per annum or tpa)		7,000	8,000	8,000	7,000	11,000	7,000	11,000	9,000	7,000	N/A	N/A	N/A	N/A	N/A	N/A	N/A	N/A	7,000	4,000	10,000	20,000	8,900	4,000	22,000	
Comparison of sign of annual changes between artisanal-production-B and NGO estimates			different	same	different	same	different	different	different	same	N/A	N/A	N/A	N/A	N/A	N/A	N/A	N/A	N/A	same	different	different	Same for 4			

Supporting Dataset 12: Artisanal processing as a percentage of artisanal-production-A and -B, as well as artisanal imports share of total Chinese imports

		2000	2001	2002	2003	2004	2005	2006	2007	2008	2009	2010	2011	2012	2013	2014	2015	2016	2017	2018	2019	2020				Notes
Artisanal processing as a percentage of artisanal-production-A		0%	0%	17%	20%	11%	8%	20%	20%	23%	91%	63%	60%	64%	100%	50%	59%	78%	60%	94%	85%	78%				Artisanal processing divided by artisanal-production-A
Artisanal Chinese imports as a percentage of total Chinese imports		100%	71%	87%	54%	71%	72%	60%	100%	76%	15%	35%	39%	38%	37%	42%	47%	18%	39%	48%	74%	100%				Artisanal Chinese imports divided by total Chinese imports
Artisanal processing as a percentage of artisanal-production-B		0%	0%	33%	25%	13%	9%	22%	20%	18%	77%	53%	47%	58%	55%	50%	56%	78%	75%	76%	69%	64%				Artisanal processing divided by artisanal-production-A

Supporting Dataset 13: Literature estimates of the number of artisanal miners and the number of children working or present in the DRC, the former Katanga province, the copper-cobalt belt, or specifically at cobalt mine sites from 1999 through 2020.

		2000	2002	2003	2005	2006	2007	2008	2008	2008	2010	2012	2012	2016	2017	2018	2018	2019	2019	2019	2019	2020
**DRC all commodities**	Total (k)	150																			2,000	
**Former Katanga all commodities**	Total (k)							1,000														
	Child (k)							20														
	%							2%														
**Copper-belt (mostly Co & Au)**	Total (k)					120						120										
	Child (k)			20		54						40										
	%					42%						33%	33%									
**Cobalt**	Total (k)		60	0	>40	0	50-250		215-346	>30	67-108				255		150-200	>200	>31	>200	60-80	
	Child (k)		24	0	>20	0	20-100		28-45	>4	20-35			>5	35				>3			>0.25
	%		40%	0	54%	0	40%		13%	20%	28%				14%	12%			19%	25%		26%
**Reference**		1	2	3	4	5	6	7	8	9	5	10	11	8	12	13	14	15	14	16	17	18

Units are either in thousands (k) of people or in percentage (%) of children working or present.

Greater than symbols (>) indicate the results of mine site visits, which have not been extrapolated to estimate all artisanal and child cobalt miners.

1. ILO, “Social and labour issues in small-scale mines: Report for discussion at the Tripartite Meeting on social and labour issues in small-scale mines” (1999).
2. Global Witness, “Rush and ruin: The devastating mineral trade in Southern Katanga, DRC” (2004).
3. E. Tshilobo, “Des milliers d’enfants exploités dans les mines du Katanga” (2005).
4. Groupe One, “Problématique du travail des enfants dans les mines artisanales au Katanga.” (2007).
5. N. Tsurukawa, S. Prakash, A. Manhart, Social impacts of artisanal cobalt mining in Katanga, Democratic Republic of Congo. Öko-Institut eV - Inst. Appl. Ecol. Freibg. 49, 65 (2011).
6. World Bank, “Democratic Republic of Congo: Growth with governance in the mining sector” (2008).
7. S. Goethals, J.-P. Okenda, R. Mbaya, “Chinese mining operations in Katanga” (2009).
8. B. Faber, B. Krause, R. S. De La Sierra, “Artisanal mining, livelihoods, and child labor in the cobalt supply chain of the Democratic Republic of Congo” (2017).
9. Pact, “PROMINES study - Artisanal mining in the Democratic Republic of Congo” (2010).
10. C. Walther, “In DR Congo , UNICEF supports efforts to help child labourers return to school” (2012).
11. World Vision, “Child miners speak: Key findings on children and artisanal mining in Kambove, DRC” (2013).
12. S. Kara, Is your phone tainted by the misery of the 35,000 children in Congo’s mines? Guard. (2018).
13. Hanrui Cobalt, Industry to formally establish Responsible Cobalt Initiative, to take action on sustainable cobalt supply chain (2017).
14. BGR, Mapping of the artisanal copper-cobalt mining sector in the provinces of Haut-Katanga and Lualaba in the Democratic Republic of the Congo (2019).
15. S. J. De Silva, T. Strauss, N. Morisho, “The Mutoshi Pilot Project: Local economic impact of a project aimed at formalizing artisanal and small-scale mining” (2019).
16. L. Maiotti, B. Katz, T. Gillard, H. Koep-Andrieu, “Interconnected supply chains: a comprehensive look at due diligence challenges and opportunities sourcing cobalt and copper from the Democratic Republic of the Congo” (2019).
17. PACT, “Confronting modern slavery in DRC-UK mineral supply chains” (2020).
18. BGR, “Mining Conditions and Trading Networks in Artisanal Copper-Cobalt Supply Chains in the Democratic Republic of the Congo” (2021).

République Démocratique du Congo

PROVINCE DU KATANGA
DIVISION PROVINCIALE DES MINES
LUBUMBASHI
Le Chef de Division

STATISTIQUES DES EXPORTATIONS DE JANVIER A DECEMBRE/2009

N°	SOCIETES	MONTANTS REDEVANCE MINIERE (USD)	Produits Miniers par tonnes																							
			Conc.Cu (10-30%)	Conc.Co (4-15%)	Conc.CuCo	Cu.Noir (80-98%)	Cath.Cu (99%)	All.Blanc (30%Co ≤ 30%Cu≤	All.Rouge (±80% Cu	P-Zinc (89%Zn)	Mat-Cu (±40%)	Hetero Co (Brut)	Min Cu (Brut)	Hydro.Co (29%)	Cu.Pul (Cu 95%)	Cu.Bott (Cu 97%)	P.Plomb (P 30%)	Carb.Co (±25%)	Carb.Cu (±25%)	Nod.Cu (±97%)	Co.Electro (Co 99%)	Co-sep Mag (Co 55,25%)	Wire bars (Cu98,62%)	Anod.Sol	Scraps (Cu±30%)	Co en Granule (Co 96%)	Cassi (Sn ±55%)
1	AMCK	119 950,19	28 851,12																								
2	BAZANO	575 949,69	132,00			983,12																					
3	BOLFAST	327 491,18		16 952,00		333,00																					
4	BOSS MINING	2 750 634,35		6 335,00		1 814,00	32,00	1 632,00	2 046,00																		
5	CDM	894 437,27	20 249,30	44 720,50	21 588,75	67,50	8 806,76	350,00	647,00																		
6	CHEMAF	1 335 224,60		16 588,00		6 442,60																					
7	CMSK	253 982,49		315,00			12 666,50																				
8	CONGO LOYAL	603 579,99			6 510,07															877,45							
9	COTA MINING	111 515,98				12 407,00												14 634,50	30,00								
10	DCP	659 159,47		1 204,00		2 193,00																					
11	EXACO	364 464,34		11 681,00	12 915,92																						
12	FEZA MINING	217 307,23																									
13	FRONTIER	4 536 667,24	258 957,29			50,00																					
14	GCM	1 581 016,56						2 395,00	1 583,00									1 073,00									
5	GOLDEN AFRICAN	13 371,24				70,07	10 806,54	138,50	2 696,00	22 130,11	1 478,93																
6	GTL	0,00																									
7	HUACHIN	131 686,53					220,54							151,93	917,55												
8	JIAXING	322 519,80				3 611,96		21 923,70													215,52	171,00	342,87	248,20	194,19	66,80	
9	JMT	14 053,10				7 040,00																					
0	KATANGA METALS	44 446,34				264,00																					
1	KCC	4 050 457,80				773,28																					
2	KMP	23 544,23					38 524,62																				
3	LIDA AFRIMING	7 028,28				616,00		66,00																			
4	MAGMA	29 088,90				262,00															2 340,00						
5	MEHUL MINING	28 718,41				799,26																					
3	METALS MINES	257 057,63																									
7	MING YUE	30 139,51		15 018,00										209,00													
3	MMR	47 136,00				484,00																					
3	MUMI EXPORT	2 153 239,02	0,00	100 182,34	19 372,67																						
)	RUASHI MINING	2 433 134,90																									
	RUBAMIN	237 096,32																									420,00
	SARDC	34 779,69		4 158,09		3 114,30	19 260,31																				
	SODIMICO	13 892,88		850,00		396,00								1 227,95				16 864,96									
	SOMIKA	1 602 854,77				280,00																					
	TFM	5 396 951,59		24 742,00			1 106,00<br>66 688,79																				
	VOLCANO MINING	268 223,26		13 064,00										5 226,00<br>5 214,11	480,00			1 845,00									
	TAL	31 470 801,25	308189,71	255809,94	60387,41	42001,09	158112,05	26505,203	6972	22130,11	1478,93	0	0	12029	1397,6	0	0	34417,46	30	877,45	2555,52	171	342,87	248,2	194,19	66,8	420

total général est de: Trente un million quatre cent septante mille huit cent un, vingt cinq

20 Tonnes de Cement de Cuivre exportés par la GCM

15 Tonnes de Balayage Toiture exportés par la GCM

45,622 Tonnes de Cu.Scraps (Cu ± 99,02%) exporté par la GCM

19,148 tonnes Scraps All.Rouge (Cu ± 72%) exporté par la GCM

République Démocratique du Congo

Province du Katanga
Division Provinciale des Mines
BUREAU DES MINES

STATISTIQUES D'EXPORTATIONS DES PRODUITS MINIERS DE JANVIER A DECEMBRE 2010 (PRODUITS MINIERS PAR TONNES)

N°	SOCIETE	Conc.cu	Conc.co	Conc.cuco	Cu. Noir	Cath.cu	All.Bl	All.Rouge	Mat.Cu	P-Zinc	Hydro.co	Cu.Pul	Carb.co	Nodul.cu	Co.El	Co-sep Mag	Anod.Sol	Coltan	Cassi
1	AMCK	32154																	
2	BAZANO		31503,18				2750,01												
3	BOLFAST		13818		1560						64								
4	BOSS MINING		50213,26	50233,05		16318,25								2193,75					
5	CDM	6250,50	21165,75		14757,80				259,20										
6	CEPROD EV																		30
7	CHEMAF		504			15196,50							17055		40				
8	CIMCO					1929,30							356						
9	CLEPAD																		
10	CSMK			45907,86															25
12	COMISA					5511,30													
11	CONGO LOYAL				16 767														
12	COTA MINING		564		2 325														
13	DCP			4 298,71	337														
14	EXACO		10 283,38																
15	FEZA MINING						4 361												
16	FRONTIER	255 814,3																	
17	GCM					13 989,21	734,86	3 295,68	33	15 546	175	1 659		517,23	815	47	444,17		
18	GLOBAL MINING																		239,50
19	GOLDEN AFRICAN		32			1 090,66													
20	GTL						26 004,53												
21	HUACHIN				4 625														

N°	SOCIETE	Conc.cu	Conc.co	Conc.cuco	Cu. Noir	Cath.cu	All.Bl	All.Rouge	Mat.Cu	P-Zinc	Hydro.co	Cu.Pul	Carb.co	Nodul.cu	Co.El	Co-sep Mag	Anod.Sol	Coltan	Cassi
	**JD MINING**	**60**																	
**23**	**JIAXING**				**2 613**														
24	KATANGA METALS				567														
25	KCC			19 081,29		42 755,47								10 642,94	3 205				
26	KMP	458	1 854																
**27**	**MAGMA**				**894**														
28	MEHUL MINING										456,12								
29	METALS MINES		12 774																
30	MING YUE				60														
31	MJM		610																
32	MMR																		
33	MUMI EXPORT		5 835,41	130 251,01		1 060												**152,50**	**3 063,2**
34	PANJU																		
35	ROQ MINING	47,16	444,58								523								252,40
36	RUASHI MINING					30 814,61					33 476,12		13 266,8						
37	RUBAMIN		7 260,99		6 084,72														
38	SARDC				220														
39	SMK	3 172,25																	
40	SODIMICO				2 120														
41	SOMIKA		24 332			2 840					12 412								
42	TFM					122 446,57					57 541,83								
35	VOLCANO MINING		35 308,61		818														
**TOTAL**		**328779,2**	**221278,1**	**254871,7**	**53801,52**	**253951,86**	**33996,4**	**3295,68**	**292,2**	**15546**	**104648,1**	**1659**	**30677,8**	**13353,92**	**4060**	**47**	**444,17**	**159,72**	**3610,1**

Fait à Lubumbashi, le 05/01/2011

Chef de Bureau Provincial des Mines
Pierrot MALOBA KITUMBA

République Démocratique du Congo

Province du Katanga
Division Provinciale des Mines
BUREAU DES MINES

STATISTIQUES DES NOTES DE DEBIT RELATIVES A LA REDEVANCE MINIERE DE JANVIER A DECEMBRE 2010 (PRODUITS MINIERS PAR TONNES)

N°	SOCIETE	MONTANT (USD)	Conc.cu	Conc.co	Conc.cuco	Cu. Noir	Cath.cu	All.Bl	All.Rouge	Mat.Cu	P-Zinc	Hydro.co	Cu.Pul	Carb.co	Nodul.cu	Co.El	Co-sep Mag	Anod.Sol	Coltan	Cassi
1	AMCK	235938,36	32154																	
2	BAZANO	1029074,71		31503,18				2750,01												
3	BOLFAST	654244,43		13818		1560						64								
4	BOSS MINING	6448295,69		50213,26	50233,05		16318,25								2193,75					
5	CDM	1405044,35	6250,50	21165,75		14757,80				259,20										30
6	CEPRODEV	2476,00																		
7	CHEMAF	2990014,53		504			15196,50							17055		40				
8	CIMCO	248616,54					1929,30							356						25
9	CLEPAD	949,50																		
10	CSMK	1825873,83			45907,86															
12	COMISA	489713,87					5511,30													
11	CONGO LOYAL	1314200,40				16 767														
12	COTA MINING	117845,55		564		2 325														
13	DCP	105246,50			4 298,71	337														
14	EXACO	213798,67		10 283,38																
15	FEZA MINING	504626,46						4 361												
16	FRONTIER	6168879,01	255 814,3																	
17	GCM	3170408,61					13 989,21	734,86	3 295,68	33	15 546	175	1 659		517,23	815	47	444,17		239,50
18	GLOBAL MINING	14702,17																		
19	GOLDEN AFRICAN	121860,83		32			1 090,66													
20	GTL							26 004,53												
21	HUACHIN	325377,80				4 625														

N°	SOCIETE	MONTANT (USD)	Conc.cu	Conc.co	Conc.cuco	Cu. Noir	Cath.cu	All.Bl	All.Rouge	Mat.Cu	P-Zinc	Hydro.co	Cu.Pul	Carb.co	Nodul.cu	Co.El	Co-sep Mag	Anod.Sol	Coltan	Cassi
	**JD MINING**	**612,00**	**60**																	
**23**	**JIAXING**	**192783,58**				**2 613**														
24	KATANGA METALS	51155,34				567														
25	KCC	8544266,71			19 081,29		42 755,47								10 642,94	3 205				
26	KMP	41819,32	458	1 854																
**27**	**MAGMA**	**46859,80**				**894**														
28	MEHUL MINING	70432,89										456,12								
29	METALS MINES	301055,92		12 774																
30	MING YUE	5436,00				60														
31	MJM	14744,20		610																
32	MMR	374117,94																		
33	MUMI EXPORT	2262355,53		5 835,41	130 251,01		1 060												**152,50**	**3 063,2**
34	PANJU																			
35	ROQ MINING	34369,03	47,16	444,58								523								252,40
36	RUASHI MINING	6061623,61			-		30 814,61					33 476,12		13 266,8						
37	RUBAMIN	621846,02		7 260,99		6 084,72														
38	SARDC	16709,38				220														
39	SMK	13829,00	3 172,25																	
40	SODIMICO	200484,80				2 120														
41	SOMIKA	3050557,85		24 332			2 840					12 412								
42	TFM	23181771,61					122 446,57					57 541,83								
35	VOLCANO MINING	815001,09		35 308,61		818														
**TOTAL**		**73979960,58**	328779,2	221278,1	**254871,7**	**53801,52**	**253951,86**	**33996,4**	**3295,68**	**292,2**	**15546**	**104648,1**	**1659**	**30677,8**	**13353,92**	**4060**	**47**	**444,17**	**159,72**	**3610,1**

Fait à Lubumbashi, le 05/01/2011

République Démocratique du Congo

Province du Katanga
Division Provinciale des Mines
BUREAU DES MINES

STATISTIQUES DES NOTES DE DEBIT RELATIVES A LA REDEVANCE MINIERE
MOIS DE JANVIER A DECEMBRE 2011
(PRODUITS MINIERS PAR TONNES)

N°	SOCIETE	Montants (USD)	Conc Cu	Conc.co	Conc. cuco	Cu. Noir	Cath.cu	All. Bl	All. Rouge	P.Zin	Hydro. co	Carb.co	P. Plomb	Nodul. cu	Anod. Sol	Co.El	Mat Cu	Cu Pul	Co-sep	Hete Co	Min Cu	Colt an	Cassit
1	AMCK	2969 908,0	5 138				19571,9																
2	AMCK VENTE LOCAL	385 427,18	19848,6																				
3	BAZANO	1 332 543		51 474	7141,06			1440															
4	BOLFAST	1 004 021		16 150		324	597				512												
5	BOSS MINING	7 744 182		46 713	58736,9		24689,9							2 215,6									
6	CAM RESSOUR CES	26 664,73				206	48																
7	CDM	3 459 000		28672,6		27417,7	2287,1				360						934, 20						
8	CHEMAF	4 075 389		1 250			21 244					13695,7											
9	CIMCO	1 091 735					6 855					2 590											
10	CLEPAD	22 264,88																					308,35
11	CMSK	1 552 783			40737,5																		
12	CMSK VENTE LOCAL	13 676,51			1679,81																		
13	CONGO COPPER MILLS	41 960,02					357,38																
14	COPROCO	13 717,51																					156
15	CONGO LOYAL	2 197 895				21 045																	
16	COTA MINING	72 531,66				981,00																	
17	EXACO	87 198,19	3 543,1	2 832,7																			
18	FEZA MINING	235 063,4						2451															
19	GCM	2 734 097					11081,8	483	1 838	22513,5	412,50		1 460	32,08	459,77	760		859	50				

N°	SOCIETE	Montants (USD)	Conc Cu	Conc.co	Conc. cuco	Cu. Noir	Cath.cu	All. Bl	All. Rouge	P.Zin	Hydro. co	Carb.co	P. Plomb	Nodul. cu	Anod. Sol	Co.El	Mat Cu	Cu Pul	Co-sep	Hete. Co	Min Cu	Colt an	Cassit
20	GLOBAL MINING	10 081,70																					155
21	GOLDEN AFRICAN	195 578,21					1326																
22	GTL							31 022															
**23**	**HUACHIN**	**561 812 ,3**				**5 557**																	
24	ILUNGA BUSNESS (IBC)	13 383,40					108																
25	JMT	396 198,01				2984,04																	
26	KATANGA METALS	78 804,5				733																	
27	KCC	12528189,			85420		49292							6522,71		2615,68							
28	KISANFU MINING VENTE LOCAL	182 207,71																		66125,7			
29	MAGMA	324 142,05				3 396																	
30	METALS MINES	384 423		5 766			2036,99																
31	MING YUE	21 967,56				192																	
32	MJM	41 848,04		1 855																			
33	MMR	668 332,08																				329	3 639
34	MUMI EXPORT	9913535,8			81465,7		43421,7				21929,5												
35	MUMI VENTE LOCAL	189578,90			22525,3																		
36	PANJU	1447,19																					19,26
37	RUASHI MINING	8 048 065					37204,6				38994,2	352											
38	RUBAMIN	610 491,9		650,13		5773,8																	
39	SEK VENTE LOCAL	418 659,67	7 807,8																				
40	SMKK VENTE LOCAL	748 209,2																			36065,67		
41	SODIMICO	134337,10		25		1 240																	
42	SOMIKA	2376 149,1		16096,7			5 699				9279												
43	TFM	28 577931					126737				83 116												
44	TTT	4 097,92																				12,5	
45	VOLCANO MINING	1 144029,5	5024	35166		3154,90																	
**TOTAL**		**96633565,43**	**41361,652**	**206651,6**	**297706,52**	**73112,52**	**352450,46**	**35396,773**	**1838**	**22513,53**	**154603,7**	**16637,75**	**1460,1**	**8770,4**	**459,77**	**3375,6**	**934**	**859**	**50**	**66125,77**	**36065,67**	**342**	**4277**

Le total général est de : Nonante six millions six cent trente trois mille cinq cent soixante cinq, quarante trois

Fait à Lubumbashi, le 05/01/ 2012

Chef de Bureau Provincial des Mines

Pierrot MALOBA KITUMBA

République Démocratique du Congo

Province du Katanga
Division Provinciale des Mines
BUREAU DES MINES

STATISTIQUES DES NOTES DE DEBIT RELATIVES A LA REDEVANCE MINIERE
MOIS DE JANVIER 2012
(PRODUITS MINIERS PAR TONNES)

N°	SOCIETES	Montants (USD)	Conc cu	Conc.co	Conc. cuco	Cu. Noir	Cath.cu	All.Bl	All. Rouge	P.Zin	Mat. cu	Hydro. co	Cu.Pul	Min. cu	Hetero. Co	Carb.co	P. Plomb	Nodul. cu	Anod. sol	Co-sep	Co. El	Cass it
1	AMCK	867 202,79					6052,04 1															
2	BOLFAST	15 472,57		96			94															
3	BOSS MINING	591 351,50		3136,2	7935,91		2126,25											199,81				
4	CAM RESSOUR CES	2019,60				24																
5	CDM	332 317,48		2481,3		2718,5	277,7				162											
6	CHEMAF	230 063,67					1524									795						
7	CIMCO	82 029,08					311									490						
8	CONGO COPPER MILLS	14827,40					140															
9	CONGO LOYAL	164 703,00				1902																
10	EXACO	14460,06	1803																			
11	FEZA MINING	237 79,36						279														
12	GCM	235 857,56					1617,47		60	1518							783,51		60,815	10	60	
13	GOLDEN AFRICAN	122937,42					849,886						136,86									
14	GTL							26 398														

N°	SOCIETE	Montants (USD)	Conc. cu	Conc.co	Conc. cuco	Cu. Noir	Cath.cu	All.Bl	All. Rouge	P.Zin	Mat. cu	Hydro. co	Cu.Pul	Min. cu	Hetero. Co	Carb.co	P. Plomb	Nodul. cu	Anod. sol	Co-sep	Co. El	Cass it
15	HUACHIN	114 444,00				1 224																
16	KCC	718 119,9			7459,04		3619,58														112,82	
17	KISANFU MINING /VL	13 735,10												870,7	12657,8							
18	METALS MINES	31059,00		300			225															
19	MING YUE	6313,22				64																
20	MMR	12376,00																				
21	MUMI EXPORT	991200,99			2296,44		4560,21					4338,18										
22	RUASHI MINING	487832,03					2287					4083										
23	RUBAMIN	50074,58				575,868																
24	SEK/VL	100288,51	2099,17																			
25	SMKK/VL	45422,41												2572,4								
26	SODIMICO	7579,60				80																
27	SOMIKA	125558,04		1234,8			598					106										
28	TFM	2298609,9					13332,9					6933,84										
29	VOLCANO MINING	86136,06	2275	2867																		
**TOTAL**		**7785770,8**	**6177,2**	**10115**	**17691**	**6588,4**	**37615**	**26677**	**60**	**1518**	**162**	**15653**	**136,86**	**3443,1**	**12658**	**1285**	**783,51**	**199,81**	**60,815**	**10**	**172,82**	

Fait à Lubumbashi, le 05/02/ 2012
Chef de Bureau Provincial des Mines
Pierrot MALOBA KITUMBA

République Démocratique du Congo

Province du Katanga
Division Provinciale des Mines
BUREAU DES MINES

STATISTIQUES DES NOTES DE DEBIT RELATIVES A LA REDEVANCE MINIERE
MOIS DE FEVRIER 2012
(PRODUITS MINIERS PAR TONNES)

N°	SOCIETES	Montants (USD)	Conc cu	Conc. co	Conc. cuco	Cu. Noir	Cath.cu	All.Bl	All. Rouge	P.Zinc	Hydro. co	Cu.Pul	Min. cu	Hetero .Co	Carb.co	P. Plomb	Nodul. cu	Co. El	Cassit
1	AMCK	239400,28					1563,61												
2	BOLFAST	74699,85		1116			136												
3	BOSS MINING	665297,93		4320,3	5370,9		2936,25								63,28		66 932		
4	CDM	356082,37		3127		2018,5	923,5				72								
5	CHEMAF	131593,68					808								519,75				
6	CIMCO	71674,94					403								201				
7	CONGO COPPER MILLS	16921,98					144												
8	CONGO LOYAL	119163				1380													
9	EXACO	11102,00	1340																
10	GCM	266497,58					1814,92		240	1209,5						1274,1			
11	GLOBAL MINING	4181,67																	50,149
12	GOLDEN AFRICAN	44871,65					194,458					224,81							
13	GTL							2307											

14	HUACHIN	51425,00				550													
**N°**	**SOCIETE**	**Montants (USD)**	**Conc. cu**	**Conc. Co**	**Conc. cuco**	**Cu. Noir**	**Cath.cu**	**All.Bl**	**All. Rouge**	**P.Zin**	**Hydro. co**	**Cu.Pul**	**Min. cu**	**Hetero . Co**	**Carb. co**	**P. Plomb**	**Nodul. cu**	**Co. El**	**Cassit**
**15**	**ILUNGA BUSNES (IBS)**	**2520,62**				**29,13**													
**16**	**KATANGA METALS**	**9370,60**				**90**													
17	KCC	1012908,64			13224,5		3621,99											290,63	
18	KISANFU MINING /VL	2897,43												927					
19	MAGMA	18064,20				210													
20	METALS MINES	33171,26		600			175												
21	MMR	99778,58																	442
22	MUMI EXPORT	1022387,43			4816,05		4568,31				3552,6								
23	MUMI /VL	32531,16			5521,99														
24	RUASHI MINING	368325,87					1958				1575								
25	RUBAMIN	40380,64				444,76													
26	SEK/VL	85582,88	1920,5																
27	SMKK/VL	80496,17											4289						
28	SOMIKA	231796,01		2673,6			712				515								
29	TFM	2089923,25					11185,5				5809,8								
30	VOLCANO MINING	78879,20	3640	870		290													
**TOTAL**		**7261925,8**	**6900,53**	**12707**	**28933,4**	**5012,39**	**31144,5**	**2307**	**240**	**1209,5**	**11524**	**224,8**	**4289**	**927**	**784,03**	**1274**	**66,93**	290,63	**492,149**

Fait à Lubumbashi, le 05/03/ 2012

Chef de Bureau Provincial des Mines

Pierrot MALOBA KITUMBA

République Démocratique du Congo

Province du Katanga
Division Provinciale des Mines
BUREAU DES MINES

STATISTIQUES DES NOTES DE DEBIT RELATIVES A LA REDEVANCE MINIERE
MOIS DE MARS 2012
(PRODUITS MINIERS PAR TONNES)

N°	SOCIETES	Montants (USD)	Conc cu	Conc. co	Conc. cuco	Cu. Noir	Cath.cu	All. Bl	All. Rouge	P.Zinc	Hydro. co	Cu.Pul	Mat.cu	Min. cu	Co. El	Anod. Sol	Carb. co	P. Plomb	Nodul. cu	Conc. Etain	Conc. Tantal
1	AMCK	314304,12					2018,82														
2	BAZANO	41958,46			1262,4																
3	BAZANO /VL	23312,29	2739,3																		
4	BOLFAST	31784,34		448			66														
5	BOSS MINING	548991,83		2765,2	5747,5		2396,25										75,972		67,048		
6	CDM	348697,22		2322,8		2357,5	589				120										
7	CHEMAF	282373,43					1881										640,5				
8	CIMCO	88012,76					581										99				
9	CMSK/VL	21509,04			1386,9																
10	CONGO COPPER MILLS	9974,24					88														
11	CONGO LOYAL	84906,80				983															
12	EXACO	9797,45	961	30,5																	
13	FEZA MINING	49248,08						577													
14	GCM	343956,98					2073,22		120	1621,9		200	171		50	49,997		528,5	50		

N°	SOCIETES	Montants (USD)	Conc cu	Conc. co	Conc. cuco	Cu. Noir	Cath.cu	All. Bl	All. Rouge	P.Zinc	Hydro. co	Cu.Pul	Mat.cu	Min. cu	Co. El	Anod. Sol	Carb. co	P. Plomb	Nodul. cu	Conc. Etain	Conc. Tantal
15	GLOBAL MINING	3506,25																		75	
16	GOLDEN AFRICAN	29940,92					228,729														
17	GTL							634													
18	HUACHIN	60027,00				642															
19	KCC	814226,32			6947,2		3541,03								224,98						
20	MAGMA	5161,20				60															
21	LONG FEI	9456,93												7591,8							
22	METALS MINES	18483,24		725			25														
23	MMR	61861,57																		182	79,422
24	MUMI EXPORT	1276900,55			6560,9		5919,77				3427,3										
25	RUASHI MINING	437554,63					1972				3159										
26	RUBAMIN	60753,22				628,27															
27	SEK/VL	113357,53	2153																		
28	SMKK/VL	57421,94												2921,8							
29	SODIMICO	16903,20				160															
30	SOMIKA	197258,46		321,6			840				620										
31	TFM	2383362,76					12594,1				6268,7										
30	VOLCANO MINING	104843,85	3888	2647,5		95															
**TOTAL**		**7849846,6**	**9741**	**9260**	**21905**	**4926**	**34813,9**	**1211,7**	**120**	**1621,92**	**13595**	**200**	**171**	**10514**	**274,9**	**49,997**	**815,47**	**528,5**	**117,05**	**257**	79,422

Fait à Lubumbashi, le 05/04/ 2012

Chef de Bureau Provincial des Mines

Pierrot MALOBA KITUMBA

République Démocratique du Congo

Province du Katanga
Division Provinciale des Mines
BUREAU DES MINES

STATISTIQUES DES NOTES DE DEBIT RELATIVES A LA REDEVANCE MINIERE
MOIS D'AVRIL 2012
(PRODUITS MINIERS PAR TONNES)

N°	SOCIETES	Montants (USD)	Conc cu	Conc. co	Conc. cuco	Cu. Noir	Cath.cu	All. Rouge	P.Zinc	Hydro. co	Cu.Pul	Min. cu	Co. El	Carb. Co	P. Plomb	Nodul. cu	Conc. Etain	Conc. Tantal
1	AMCK	602531,01					4000,11											
2	BOLFAST	37659,32		416	134													
3	BOSS MINING	673937,46		7109,4	3198,886		2868,75							69,852		67,034		
4	CDM	448418, 38		2391,9		3117	694,5			264								
5	CHEMAF	194544,45					1124			153,75				423			50	
6	CDM	348697,22																
7	CIMCO	7674,48												99				
8	CONGO COPPER MILLS	28979,56					240											
9	CONGO LOYAL	167086,60				1759												
10	EXACO	6036,00	648															
11	GCM	217716,95					1369,78	120	1174,5				120		481,5			
12	GOLDEN AFRICAN	41821,46					262,367				85,549							
13	HUACHIN	39644,00				424												

14	KCC	858508,86			3929,35		4453,45						155, 4					
**N°**	**SOCIETES**	**Montants (USD)**	**Conc cu**	**Conc. co**	**Conc. cuco**	**Cu. Noir**	**Cath.cu**	**All. Rouge**	**P.Zinc**	**Hydro. co**	**Cu.Pul**	**Min. cu**	**Co. El**	**Carb. Co**	**P. Plomb**	**Nodul. cu**	**Conc. Etain**	**Conc. Tantal**
15	MAGMA	5161,20				60												
16	LONG FEI/VL	6103,78										5115						
17	METALS MINES	35283,50		1325			75											
18	MMR	56907,50															312	26
19	MUMI EXPORT	1494503,52			5425,29		6738,88			5273,89								
20	RUASHI MINING	629210,66					3140			3448								
21	RUBAMIN	57958,76				604,89												
22	SMKK/VL	65705,36										3329,7						
23	SODIMICO	5425,40		80		40												
24	SOMIKA	188909,87		30		252,5	833			298								
25	TFM	1954541,00					11147,7			4709,26								
26	VOLCANO MINING	93813,50	2830,4	3053														
**TOTAL**		**7928688,2**	**4749,47**	**14405**	**12553,5**	**6257**	**37081,5**	**120**	1174,5	**14146,9**	**85,549**	**8444,7**	**275,4**	**591,85**	**481,5**	**67,034**	**362**	26

Fait à Lubumbashi, le 05/05 2012
Chef de Bureau Provincial des Mines
Pierrot MALOBA KITUMBA

République Démocratique du Congo

Province du Katanga
Division Provinciale des Mines
BUREAU DES MINES

STATISTIQUES DES NOTES DE DEBIT RELATIVES A LA REDEVANCE MINIERE
MOIS DE MAI 2012
(PRODUITS MINIERS PAR TONNES)

N°	SOCIETES	Montants (USD)	Conc cu	Conc. co	Conc. cuco	Cu. Noir	Cath.cu	All. Blanc	P.Zinc	Hydro. co	Cu.Pul	Min. cu	Co. El	Carb. co	P. Plomb	Nodul. cu	Hetero .Co	Conc. Etain
1	AMCK	631181,78					2895,2											
2	BAZANO /VL	39181,86	1697,79	2814,9 3														
3	BOLFAST	54761,49		800			96											
4	BOSS MINING	518752,97		3422,8	5726,569		1957,5							48,628		67,034		
5	CDM	317811,54		2543,1		2322,5	210			312								
6	CHEMAF	226731,70					1318			558,75			12					
7	CDM	348697,22																
8	CIMCO	99307,20					688							64				
9	CONGO COPPER MILLS	22305,70					190											
10	COPROCO	2431,00																26
11	CONGO LOYAL	79302,00				808												
12	EXACO	22089,60	1062															
13	FEZA MINING	76475,96						890										
14	GCM	193325,28					1286,52		2110,8				70		671,02			

15	GOLDEN AFRICAN	33692,41					240				32,5							
**N°**	**SOCIETES**	**Montants (USD)**	**Conc cu**	**Conc. co**	**Conc. cuco**	**Cu. Noir**	**Cath.cu**	**All. Blanc**	**P.Zinc**	**Hydro. co**	**Cu.Pul**	**Min. cu**	**Co. El**	**Carb. co**	**P. Plomb**	**Nodul. cu**	**Hetero .Co**	**Conc. Etain**
16	GTL							1394,495										
17	HUACHIN	60739,20				648												
**18**	KCC	733967,43			4468,512		3769,65						112,73					
19	MAGMA	40912,80				468												
20	LONG FEI/VL	4449,99										3841,5						
21	METALS MINES	53728,50		1350			225											
22	MMR	45592,64																234
23	MUMI EXPORT	1722702,57			4026,5		8250,55			5557,54								
24	RUASHI MINING	474755,47					2251,5			3165								
25	SEK EXPORT	5760,00	360															
26	RUBAMIN	64800,18				691,63												
27	SEK/VL	147524,92	2762,56															
28	SMKK/VL	59380,41										3081,1						
29	SODIMICO	644,80		40														
30	SOMIKA	178471,36		1187		203	786			52								
31	TFM	2520732,83					13314,7			7754,88								
32	SODIMICO /VL	320,79															82	
33	VOLCANO MINING	58818,90	2714	1115		86												
**TOTAL**		**8490653,29**	**7534,34**	**11520**	**17036,5**	**5227**	**37478,6**	**2284,49**	**2110,82**	**17400,2**	**32,5**	**6922,6**	**194,73**	**112,63**	**671,02**	**67,034**	**82**	260

Fait à Lubumbashi, le 05/06/2012
Chef de Bureau Provincial des Mines
Pierrot MALOBA KITUMBA

République Démocratique du Congo

Province du Katanga
Division Provinciale des Mines
BUREAU DES MINES

STATISTIQUES DES NOTES DE DEBIT RELATIVES A LA REDEVANCE MINIERE
MOIS DE JUIN 2012
(PRODUITS MINIERS PAR TONNES)

N°	SOCIETES	Montants (USD)	Conc cu	Conc. co	Conc. cuco	Cu. Noir	Cath.cu	All.Bl	All. Rouge	P.Zinc	Hydro. co	Cu.Pul	Mat.cu	Min. cu	Co. El	Co-sep Mag	Carb. co	P. Plomb	Nodul. cu	Conc. Etain	Conc. Tantal
1	AMCK	488611,41					3713,93														
2	BAZANO /VL	13389,13	1376,8		1262,4																
3	BOLFAST	49144,96		694			96														
4	BOSS MINING	561587,94			9026,7		1890										55,428		200,86		
5	CDM	465679,64		4010,8		3175	883				72		375,3								
6	CHEMAF	252110,60					1966				267,75									25	
7	CIMCO	29447,75					221										34				
8	CONGO COPPER MILLS	11424,32					90					25									
9	CONGO LOYAL	126296,40				1396															
10	FEZA MINING	22655,00						260													
11	GCM	243165,71					1401,6		180	1928,2		250			80	10		122			
12	GOLDEN AFRICAN	18384,57					140					32,5									
13	GTL							3429,5													

14	HUACHIN	65076,00				696																
**N°**	**SOCIETES**	**Montants (USD)**	**Conc cu**	**Conc. co**	**Conc. cuco**	**Cu. Noir**	**Cath.cu**	**All.Bl**	**All. Rouge**	**P.Zinc**	**Hydro. co**	**Cu.Pul**	**Mat.cu**	**Min. cu**	**Co. El**	**Co-se Mag**	**Carb. co**	**P. Plomb**	**Nodul. cu**	**Conc. Etain**	**Conc. Tantal**
15	KATANGA METALS	17591,60				192															
16	KCC	733330,29			4431,2		3577,41								223,09						
17	KISANFU MINING/VL	39401,59												4564,6							
18	LONG FIE /VL	2440,22												2684,1							
19	METALS MINES	24985,74		825			75														
20	MMR	39913,10																		260	17
21	MUMI EXPORT	1055307,68			2597,9		6129,95				2758,3										
22	RUASHI MINING	404274,53					1777				3789										
23	SEK EXPORT	25376,00	1586																		
24	RUBAMIN	10606,96				117,94															
25	SEK/VL	23777,14	4506,4																		
26	SMKK/VL	86599,06												4686,1							
27																					
28	SODIMICO	7307,60				80															
29	SOMIKA	266812,09		1766,4		285,4	858				784,8										
30	TFM	1854145,45					10920,2				5507,5										
31	VOLCANO MINING	39434,50	4151																		
**TOTAL**		**7192275,98**	**11620,2**	**7296,2**	**16055,875**	**5942,34**	**33739,1**	**3689,54**	**180**	**1928,28**	**13179,4**	**307,5**	**375,3**	**11935**	**303,097**	**10**	**89,428**	**122**	**200,86**	**285**	**17**

Fait à Lubumbashi, le 05/07/ 2012

Chef de Bureau Provincial des Mines

Pierrot MALOBA KITUMBA

République Démocratique du Congo

Province du Katanga
Division Provinciale des Mines
BUREAU DES MINES

STATISTIQUES DES NOTES DE DEBIT RELATIVES A LA REDEVANCE MINIERE
MOIS DE JUILLET 2012
(PRODUITS MINIERS PAR TONNES)

N°	SOCIETES	Montants (USD)	Conc cu	Conc. co	Conc. cuco	Cu. Noir	Cath.cu	All.Bl	All. Rouge	P.Zinc	Hydro. co	Cu. Pul	Mat.cu	Min. Cu	Co. El	Carb. co	Nodul. cu	Conc. Etain	Conc. Tantal
1	AMCK	571473,03					4244,09												
2	BAZANO /VL	34322,72	1972,4																
3	BOLFAST	22816,53		96		30	128												
4	BOSS MINING	583374,29			9577,5		2261,25									60,968	235,2		
5	CDM	360126,33		3160,1		2236,5	859				216		129,6						
6	CHEMAF	381500,38					2410				1107,85							75	
7	CIMCO	107716,44					826,872									648			
8	CMSK/VL	36372,53			2233,2														
9	CONGO COPPER MILLS	4141,20					35												
10	COPROCO	2244,00																25	
11	CONGO LOYAL	151179,60				1815													
12	GCM	279764,93					1656,94		180	2130,9					50				
13	GOLDEN AFRICAN	12978,30					70					52							

14	GTL							2163,927											
**N°**	**SOCIETES**	**Montants (USD)**	**Conc cu**	**Conc. co**	**Conc. cuco**	**Cu. Noir**	**Cath.cu**	**All.Bl**	**All. Rouge**	**P.Zinc**	**Hydro. co**	**Cu. Pul**	**Mat.cu**	**Min. Cu**	**Co. El**	**Carb. co**	**Nodul. cu**	**Conc. Etain**	**Conc. Tantal**
15	HUACHIN	80036,00				856													
**16**	**HUACHIN METAL LEACH**	**10287,01**					**94,02**												
17	KATANGA METALS	14091,00				15O													
18	KCC	594172,98			2771,022		3455,92								158,0975				
19	LONG FIE /VL	1862,93												2412,3					
20	METALS MINES	31365,00		875			125												
21	MMR	33172,78																234	14
22	MUMI EXPORT	1353202,57			2689, 34		7468,13				5015,22								
23	RUASHI MINING	492207,64					2555,5				3495								
24	SEK EXPORT	76688,00	4793																
25	RUBAMIN	114006,31				1304,45													
26	SMKK/VL	79752,33												4616,7					
27	SODIMICO	22530,80				240													
28	SOMIKA	415379,97		634,8		312	746				574,4								
29	TFM	2483766,18					14719,2				8168,72								
30	VOLCANO MINING	14820,00	1550																
**TOTAL**		**8365351,75**	**8325,4**	**4765,9**	**17271**	**6943,95**	**41654,9**	**2163,92**	**180**	**2130,9**	**18577,2**	**52**	**129,6**	7029,1	**208,098**	**708,9**	**235,2**	**333**	**14**

Fait à Lubumbashi, le 05/08/ 2012
Chef de Bureau Provincial des Mines
Pierrot MALOBA KITUMBA

République Démocratique du Congo

Province du Katanga
Division Provinciale des Mines
BUREAU DES MINES

STATISTIQUES DES NOTES DE DEBIT RELATIVES A LA REDEVANCE MINIERE
MOIS D'AOUT 2012
(PRODUITS MINIERS PAR TONNES)

N°	SOCIETES	Montants (USD)	Conc cu	Conc.co	Conc. cuco	Cu. Noir	Cath.cu	All.Bl	All. Rouge	P.Zin	Mat. cu	Hydro. co	Cu.Pul	Min. cu	Carb.Co	Carb.Cu	P. Plomb	Nodul. cu	Co.El	Co-sep	Conc.Et ain	Conc.T antal
1	AMCK (MMG)	504917,32					3801,64															
2	BAZANO	114944,94		2416,1				838														
3	BOLFAST	106213,78		1922			32															
4	BOSS MINING	545276,67			9002,72		1687,5								53,593			904,39				
5	CDM	475326,12		4477,8		3383,5	912				356	120										
6	CHEMAF	235575,20					1824					318										
7	CIMCO	47040, 97					339								478							
8	CMSK/VL	70663,97			5187,19																	
9	CONGO LOYAK	67086,60				795																
10	CONCORDE POUR L'INDUSTRIE	3538,92														231						
11	FEZA MINING	9363,42						99														
12	GCM	204190,21					861,128		180	1600,82			300		70		289		100	10		
13	GOLDEN AFRICAN	5379,33										58,5										
14	GTL							2162,03														

15	HUACHIN	79157,76				836																
**N°**	**SOCIETES**	**Montants (USD)**	**Conc cu**	**Conc.co**	**Conc. cuco**	**Cu. Noir**	**Cath.cu**	**All.Bl**	**All. Rouge**	**P.Zin**	**Mat. cu**	**Hydro. co**	**Cu.Pul**	**Min. cu**	**Carb.Co**	**Carb.Cu**	**P. Plomb**	**Nodul. cu**	**Co.El**	**Co-sep**	**Conc.Etain**	**Conc.Tantal**
16	HUACHIN METAL LEACH	276714,56					2544,4															
17	KCC	759207,78			4362,66		3948,93											119,08	112,957			
18	MAGMA	25806,00				300																
19	LONG FEI /VL	1051,01												1477,2								
20	METALS MINES	42232,25		575			325															
21	MMR	48747,50																			390	12
22	MUMI EXPORT	1093368,2			3158,76		6710,58					2914,91										
23	RUASHI MINING	595418,40					3631,5					2687										
24	SEK EXPORT	60919,20	3852																			
25	RUBAMIN	10582,86				122,063																
26	SMKK/VL	71019,71												4010,5								
27	SODIMICO	27801,60		210		240																
28	SOMIKA	128682,57				438,5	486					202										
29	TFM	2201823,4					14195,1					6085,06										
30	VOLCANO MINING	66891,10	1480	2351																		
**TOTAL**		**7878941,49**	**5332**	**11952**	**21711,337**	**6115,06**	**41298,8**	**3099,035**	**180**	**1600,8**	**356,4**	**12385,5**	**300**	**5487,7**	**601,6**	**231**	**289**	**1023,5**	**212,957**	**10**	**390**	**12**

Fait à Lubumbashi, le 05/09/ 2012

Chef de Bureau Provincial des Mines

Pierrot MALOBA KITUMBA

République Démocratique du Congo

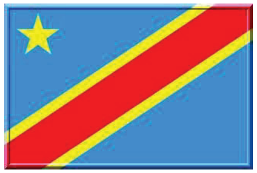

Province du Katanga
Division Provinciale des Mines
BUREAU DES MINES

STATISTIQUES DES NOTES DE DEBIT RELATIVES A LA REDEVANCE MINIERE
MOIS DE SEPTEMBRE 2012
(PRODUITS MINIERS PAR TONNES)

N°	SOCIETES	Montants (USD)	Conc cu	Conc.co	Conc. cuco	Cu. Noir	Cath.cu	All.Bl	All. Rouge	P.Zin	Mat. cu	Hydro. co	Cu.Pul	Min. cu	Carb.Co	Carb.Cu	P. Plomb	Nodul. cu	Co.El	Co-sep	Conc.Etain	Conc.Tantal
1	AMCK (MMG)	453980,90					3190,52															
2	BAZANO	81645,27		2689,6		343,05																
3	BOLFAST	63831,98		1136			30															
4	BOSS MINING	419093,91			6990,74		1350								11,308			671,23				
5	CAM RESSOURCES	11934,00					90															
6	CDM	378947,83		3984,7		2462	804				97,2	198										
7	CHEMAF	234159,68					1762					330										
8	CIMCO	33318,14					275															
9	CMSK	19459,80			1066,8																	
10	CONGO LOYAK	92156,20				1076																
11	CONCORDE POUR L'INDUSTRIE	11520,64														752						
12	GCM	330985,76					1851,81		60	1459,5					30		474,5		110	10		
13	GOLDEN AFRICAN	67612,15					465						125,2									

14	GTL							2958,80														
**N°**	**SOCIETES**	**Montants (USD)**	**Conc cu**	**Conc.co**	**Conc. cuco**	**Cu. Noir**	**Cath.cu**	**All.Bl**	**All. Rouge**	**P.Zin**	**Mat. cu**	**Hydro. co**	**Cu.Pul**	**Min. cu**	**Carb.Co**	**Carb.Cu**	**P. Plomb**	**Nodul. cu**	**Co.El**	**Co-sep**	**Conc.Etain**	**Conc.Tantal**
15	HUACHIN	59840,00				640																
16	HUACHIN METAL LEACH	117562,44					1039,66															
17	KCC	1065152,8			10439,1		4640,79												180,795			
18	MAGMA	5101,20				60																
19	LONG FEI /VL	1026,28												1617								
20	LUNA MINING /VL	48258,07	10819,9																			
21	METALS MINES	37530,50		825			200															
22	MMR	46704,78																			260	40
23	MUMI EXPORT	1054657,1			2172,33		6697,96					2772,41										
24	SINO KATANGA (STK)	3404,46																			22,27	
25	SHITURU MINING	151452,63					1169,29															
26	RUASHI MINING	339346,89					1471					3357										
27	SEK EXPORT	44856,00	2670																			
28	RUBAMIN	100816,83				1145,03																
29	SEK /VL	341764,22	7013,82																			
30	SMKK/VL	70195,45												4015,5								
31	SODIMICO	15575,20				160																
32	SOMIKA	111511,57		335,4		281	410					186										
33	TFM	1899937,5					12566,3					4093,55										
34	VOLCANO MINING	11856,60	720	224																		
**TOTAL**		**7725194, 94**	**21223, 7**	**9194,7**	**20668, 997**	**6167,0 8**	**38013, 4**	**2958, 809**	**160**	**1459,5**	**97,2**	**10937**	**125,2**	**5632,5**	**41,308**	**752**	**474,5**	**671,23**	**290, 795**	**10**	**282,27**	**40**

Fait à Lubumbashi, le 05/10/ 2012

Chef de Bureau Provincial des Mines

Pierrot MALOBA KITUMBA

République Démocratique du Congo

Province du Katanga
Division Provinciale des Mines
BUREAU DES MINES

STATISTIQUES DES NOTES DE DEBIT RELATIVES A LA REDEVANCE MINIERE
MOIS D'OCTOBRE 2012
(PRODUITS MINIERS PAR TONNES)

N°	SOCIETES	Montants (USD)	Conc cu	Conc. co	Conc. cuco	Cu. Noir	Cath.cu	All.Bl	All. Rouge	P.Zinc	Hydro. Co	Cu. Pul	Mat.cu	Co. El	Co-sep Mag	Anod. Sol	P. Plomb	Nodul. cu	Conc. Etain	Conc. Tantal
1	AMCK (MMG)	387712,70					2666,33													
2	BAZANO	16093,75				135,82														
3	BOLFAST	27001,14		456			32													
4	BOSS MINING	408407,82			4022,64		1687,5											603,63		
5	CDM	500389,07		2369,8		3568,5	852				378		129,6							
6	CHEMAF	211575,36					1339				495			10						
7	CIMCO	122490,27					942													
8	CMSK	41247,90			1308,56															
9	CONGO LOYAL	54268,60				632														
10	FEZA MINING	38236,68						384												
11	GCM	495440,19					2667,93		180	1366,5				110	10	120,04	641			
12	GOLDEN AFRICAN	28853,10					195,5					50								
13	GTL							3429,5												

14	HUACHIN	40596,00				408														
**N°**	**SOCIETES**	**Montants (USD)**	**Conc cu**	**Conc. co**	**Conc. cuco**	**Cu. Noir**	**Cath.cu**	**All.Bl**	**All. Rouge**	**P.Zinc**	**Hydro. Co**	**Cu. Pul**	**Mat.cu**	**Co. El**	**Co-se Mag**	**Anod. Sol**	**P. Plomb**	**Nodul. cu**	**Conc. Etain**	**Conc. Tantal**
**15**	**HUACHIN METAL LEACH**	**143721,39**					**1200,43**													
16	KATANGA METALS	18854,50				187														
17	KCC	1447739,48			16589,46		3387,77							178,22				3012,6		
18	KISANFU MINING/VL	52071,61																		
19	LUNA MINING/VL	32460,30																		
20	METALS MINES	29299,50		475			175													
21	OM METAL	8221,26					83,38													
22	MMR	56845,62																	390	18
23	MUMI EXPORT	1482306,16			1814,21		8960,2				3495,95									
24	SHITURU MINING	379258,92					2933,45													
25	RUASHI MINING	639422,82					3310				3594,08									
26	SEK EXPORT	11340,00	675																	
27	RUBAMIN	158270,28				1714,56														
28	SMKK/VL	43895,89																		
29	SODIMICO	32798,40				320														
30	SOMIKA	122410,86		297,7		336	312				384									
31	TFM	2250317,21					14645,9				3818,88									
32	VOLCANO MINING	16273,60	800	384																
**TOTAL**		**9297820,3**	**1475**	**3964,5**	**23734,8**	**7301,88**	**45390,3**	**1479,7**	**180**	**1366,5**	**12165,9**	**50**	**129,6**	**298,2**	**10**	120,04	**641**	**3616,2**	**390**	**18**

Fait à Lubumbashi, le 05/11/ 2012
Chef de Bureau Provincial des Mines
Pierrot MALOBA KITUMBA

République Démocratique du Congo

Province du Katanga
Division Provinciale des Mines
BUREAU DES MINES

STATISTIQUES DES NOTES DE DEBIT RELATIVES A LA REDEVANCE MINIERE
MOIS DE NOVEMBRE 2012
(PRODUITS MINIERS PAR TONNES)

N°	SOCIETES	Montants (USD)	Conc cu	Conc. co	Conc. cuco	Cu. Noir	Cath.cu	All.Bl	All. Rouge	P.Zinc	Hydro. co	Cu.Pul	Mat.cu	Min. cu	Co. El	Co-sep Mag	Hetero Co	P. Plomb	Nodul. cu	Conc.Etain	Conc.Tantal
1	AMCK (MMG)	462044,01					3367,82														
2	BAZANO	34823,92		1594,6																	
3	BOLFAST	29789,75		450			61														
4	BOSS MINING	580697,28		323,66	11778,48		1957,5												235,19		
5	CDM	182679,79		1600,3		58	1039				390		129,6								
6	COMIDE/VL	14665,98	340,73																		
7	CHEMAF	204945,88					1376				565,5										
8	CIMCO	112342,80					857														
9	CMSK	115244,96			4444,8																
10	CMSK/VL	15179,40	1689,5																		
11	CONGO LOYAL	52098,00				606															
12	FRONTIER	51000,00	2000																		
13	EXACO	2296,00	280																		
14	GCM	646469,45				2862	2012,6		60	288,66					50	10		332,5			
15	GOLDEN AFRICAN	16501,76					100					50,8									
**16**	**GTL**							756,11													
17	HUACHIN	67003,80				624															
18	HUACHIN METAL LEACH	26925,					238,546														

N°	SOCIETES	Montants (USD)	Conc cu	Conc. co	Conc. cuco	Cu. Noir	Cath.cu	All.Bl	All. Rouge	P.Zinc	Hydro. co	Cu.Pul	Mat.cu	Min. cu	Co. El	Co-sep Mag	Hetero Co	P. Plomb	Nodul. cu	Conc.Etain	Conc.Tantal
19	LOMAMINE	4335,82																		25	
20	KATANGA METALS	16872,00				180															
21	KCC	1663856,81			12711		1618,61								245,44				7113,3		
22	LUNA MINING /VL	19986,15	3569,2																		
23	METALS MINES	31875,00		775			150														
24	MMR	22544,22																		130	17
25	MUMI EXPORT	1213619,00			1485,4		7494,94				2999,1										
26	OM METAL	5831,32					59,84														
27	RUBAMIN/ VL	958,03															529,95				
28	RUASHI MINING	237442,95					1375				1018										
29	RUBAMIN	61527,74				639,27															
30	SEK EXPORT	35270,14	1821,1																		
31	SINO KATANGA (STK)	2984,12																		23,41	
32	SEK/VL	253994,64	5101,2																		
33	SHITURU MINING	189895,72					1449,35														
34	SMKK/VL	15426,92												819,32							
35	SODIMICO	28846,60		210		240															
36	SOMIKA	140661,95				87	676				396										
37	TFM	1757360,79					11756,9				3915,6										
38	VOLCANO MINING	58988,84	2440	1628																	
**TOTAL**		**8376086,8**	**17241,8**	**19293**	**17709**	**5296,33**	**35590,1**	**756,1**	**60**	**288,6**	**9284,33**	**50,8**	**129,6**	819,32	**295,442**	**10**	**529,95**	**332,5**	**7348,4**	**178,41**	**17**

Fait à Lubumbashi, le 05/12/ 2012
Chef de Bureau Provincial des Mines
Pierrot MALOBA KITUMBA

République Démocratique du Congo

Province du Katanga
Division Provinciale des Mines
BUREAU DES MINES

STATISTIQUES DES NOTES DE DEBIT RELATIVES A LA REDEVANCE MINIERE
MOIS DE DECEMBRE 2012
(PRODUITS MINIERS PAR TONNES)

N°	SOCIETES	Montants (USD)	Conc cu	Conc. co	Conc. cuco	Cu. Noir	Cath.cu	All.Bl	All. Rouge	P.Zinc	Hydro. Co	Cu. Pul	Mat.cu	Co. El	Co-sep Mag	Anod. Sol	P. Plomb	Nodul. cu	Conc. Etain	Conc. Tantal
1	AMCK (MMG)	696021,39					4896,13													
2	BAZANO	84941,18		1837		406														
3	BOSS MINING	386298,27			9139,221		1248,75											134,5		
4	CAM RESSOURCES	15254,02					129,36													
5	CDM	151972,02		2062			673				540		129,6							
6	CHEMAF	188129,50					1350				310,5								13	
7	CIMCO	97778,90					736													
8	CMSK	126727,30			4604,686															
9	FEZA MINING	14471,50						160												
10	GCM	566354,70				2358,04	1280,25		120	1322,3		300		50	10	59,9	233	68,943		
11	GOLDEN AFRICAN	40185,08					277					80								
12	GTL							743,193												
13	HUACHIN	31824,00				288														
14	MINES D'AFRIQUE	7557,92																	45,7	
15	HUACHIN METAL LEA	37059,02				330,38														

N°	SOCIETES	Montants (USD)	Conc cu	Conc. co	Conc. cuco	Cu. Noir	Cath.cu	All.Bl	All. Rouge	P.Zinc	Hydro. Co	Cu. Pul	Mat.cu	Co. El	Co-se Mag	Anod. Sol	P. Plomb	Nodul. cu	Conc. Etain	Conc. Tantal
**16**	**KCC**	**1909596,9**			**11520,5**		**1996,59**							**157,3**				**9495,9**		
17	LUNA MINING/VL	27620,22																		
18	METALS MINES	18976,25		625			75													
19	RUBAMIN /VL	546,87																		
20	MMR	45802,25																	312	13
21	MUMI EXPORT	1362878,87			1599,43		8520,79				2998,87									
22	SHITURU MINING	169100,80					1269,45													
23	RUASHI MINING	317483,45					1587				3042									
24	SEK EXPORT	23392,41	988,62																	
25	RUBAMIN	65351,83				697,408														
26	SMKK/VL	32972,19																		
27	SODIMICO	15735,20				160														
28	SOMIKA	52963,16		56			234				234									
29	TFM	2290890,18					16337,9				3088,84									
30	VOLCANO MINING	45453,00	1850,5	1276																
**TOTAL**		**8823339,4**	**2849**	**5856**	**26863,8**	**4239,82**	**40611,2**	**903,193**	**120**	**1322,32**	**10214,2**	**380**	**129,6**	**207,35**	**10**	**59,9**	**233**	**9699,3**	**370,7**	**13**

Fait à Lubumbashi, le 05/01/ 2013
Chef de Bureau Provincial des Mines
Pierrot MALOBA KITUMBA

République Démocratique du Congo

Province du Katanga
Division Provinciale des Mines
BUREAU DES MINES

STATISTIQUES D'EXPORTATIONS DES PRODUITS MINIERS
MOIS DE JANVIER 2012
(PRODUITS MINIERS PAR TONNES)

N°	SOCIETES	Conc cu	Conc.co	Conc. cuco	Cu. Noir	Cath.cu	All.Bl	All. Rouge	P.Zin	Mat. cu	Hydro. co	Cu.Pul	Carb. co	P. Plomb	Nodul. cu	Anod. sol	Co-sep	Co. El	Cas sit
1	AMCK					6052,041													
2	BOLFAST		96			94													
3	BOSS MINING		3136,2	7935,91		2126,25									199,81				
4	CAM RESSOURCES				24														
5	CDM		2481,3		2718,5	277,7				162									
6	CHEMAF					1524							795						
7	CIMCO					311							490						
8	CONGO COPPER MILLS					140													
9	CONGO LOYAL				1902														
10	EXACO	1803																	
11	FEZA MINING						279												
12	GCM					1617,47		60	1518					783,51		60,815	10	60	
13	GOLDEN AFRICAN					849,886						136,86							

N°	SOCIETE	Conc. cu	Conc.co	Conc. cuco	Cu. Noir	Cath.cu	All.Bl	All. Rouge	P.Zin	Mat. cu	Hydro. co	Cu.Pul	Carb.c o	P. Plomb	Nodul. cu	Anod. sol	Co-sep	Co. El	Cas sit
14	GTL						26 398												
15	HUACHIN				1 224														
16	KCC			7459,04		3619,58												112,82	
17	METALS MINES		300			225													
18	MING YUE				64														
19	MMR																		
20	MUMI EXPORT			2296,44		4560,21					4338,18								
21	RUASHI MINING					2287					4083								
22	RUBAMIN				575,868														
23	SODIMICO				80														
24	SOMIKA		1234,8			598					106								
25	TFM					13332,9					6933,84								
26	VOLCANO MINING	2275	2867																
**TOTAL**		**4 078,03**	**10115**	**17691**	**6588,4**	**37615**	**26677**	**60**	**1518**	**162**	**15653**	**136,86**	**1285**	**783,51**	**199,81**	**60,815**	**10**	**172,82**	

Fait à Lubumbashi, le 05/02/ 2012

Chef de Bureau Provincial des Mines

Pierrot MALOBA KITUMBA

République Démocratique du Congo

Province du Katanga
Division Provinciale des Mines
BUREAU DES MINES

STATISTIQUES D'EXPORTATIONS DES PRODUITS MINIERS
MOIS DE FEVRIER 2012
(PRODUITS MINIERS PAR TONNES)

N°	SOCIETES	Conc cu	Conc. co	Conc. cuco	Cu. Noir	Cath.cu	All.Bl	All. Rouge	P.Zinc	Hydro. co	Cu.Pul	Carb.co	P. Plomb	Nodul. cu	Co. El	Cassit
1	AMCK					1563,61										
2	BOLFAST		1116			136										
3	BOSS MINING		4320,3	5370,9		2936,25						63,28		66 932		
4	CDM		3127		2018,5	923,5				72						
5	CHEMAF					808						519,75				
6	CIMCO					403						201				
7	CONGO COPPER MILLS					144										
8	CONGO LOYAL				1380											
9	EXACO	1340														
10	GCM					1814,92		240	1209,5				1274,1			
11	GLOBAL MINING															50,149
12	GOLDEN AFRICAN					194,458					224,81					
13	GTL						2307									

N°	SOCIETE	Conc. cu	Conc. Co	Conc. cuco	Cu. Noir	Cath.cu	All.Bl	All. Rouge	P.Zin	Hydro. co	Cu.Pul	Carb. co	P. Plomb	Nodul. cu	Co. El	Cassit
14	HUACHIN				550											
**15**	**ILUNGA BUSNES (IBS)**				**29,13**											
**16**	**KATANGA METALS**				**90**											
17	KCC			13224,5		3621,99									290,63	
18	MAGMA				210											
19	METALS MINES		600			175										
20	MMR															442
21	MUMI EXPORT			4816,05		4568,31				3552,6						
22	RUASHI MINING					1958				1575						
23	RUBAMIN				444,76											
24	SOMIKA		2673,6			712				515						
25	TFM					11185,5				5809,8						
26	VOLCANO MINING	3640	870		290											
**TOTAL**		**4980,03**	**12707**	**23411,4**	**5012,39**	**31144,5**	**2307**	**240**	**1209,5**	**11524**	**224,8**	**784,03**	**1274**	**66,93**	290,63	**492,149**

Fait à Lubumbashi, le 05/03/ 2012
Chef de Bureau Provincial des Mines
Pierrot MALOBA KITUMBA

République Démocratique du Congo

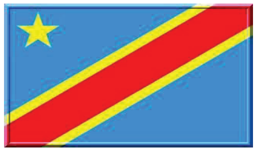

Province du Katanga
Division Provinciale des Mines
BUREAU DES MINES

STATISTIQUES D'EXPORTATIONS DES PRODUITS MINIERS
MOIS DE MARS 2012
(PRODUITS MINIERS PAR TONNES)

N°	SOCIETES	Conc cu	Conc. co	Conc. cuco	Cu. Noir	Cath.cu	All.Bl	All. Rouge	P.Zinc	Hydro. co	Cu.Pul	Mat .cu	Co. El	Anod.Sol	Carb. co	P. Plomb	Nodul. cu	Conc. Etain	Conc.Ta ntal
1	AMCK					2018,82													
2	BAZANO			1262,4															
3	BAZANO /VL	2739,3																	
4	BOLFAST		448			66													
5	BOSS MINING		2765,2	5747,5		2396,25									75,972		67,048		
6	CDM		2322,8		2357,5	589				120									
7	CHEMAF					1881									640,5				
8	CIMCO					581									99				
9	CMSK/VL			1386,9															
10	CONGO COPPER MILLS					88													
11	CONGO LOYAL				983														
12	EXACO	961	30,5																
13	FEZA MINING						577												
14	GCM					2073,22		120	1621,9		200	171	50	49,997		528,5	50		
15	GLOBAL MINING																	75	

N°	SOCIETES	Conc cu	Conc. co	Conc. cuco	Cu. Noir	Cath.cu	All.Bl	All. Rouge	P.Zinc	Hydro. co	Cu.Pul	Mat .cu	Co. El	Anod.Sol	Carb. co	P. Plomb	Nodul. cu	Conc. Etain	Conc.Tantal
16	GOLDEN AFRICAN					228,729													
17	GTL						634												
18	HUACHIN				642														
19	KCC			6947,2		3541,03							224,98						
20	MAGMA				60														
22	METALS MINES		725			25													
23	MMR																	182	79,422
24	MUMI EXPORT			6560,9		5919,77				3427,3									
25	RUASHI MINING					1972				3159									
26	RUBAMIN				628,27														
29	SODIMICO				160														
30	SOMIKA		321,6			840				620									
31	TFM					12594,1				6268,7									
30	VOLCANO MINING	3888	2647,5		95														
**TOTAL**		**7588**	**9260**	**21905**	**4926**	**34813,9**	**1211,7**	**120**	**1621,92**	**13595**	**200**	**171**	**274,9**	**49,997**	**815,47**	**528,5**	**117,05**	**257**	79,422

Fait à Lubumbashi, le 05/04/ 2012
Chef de Bureau Provincial des Mines
Pierrot MALOBA KITUMBA

République Démocratique du Congo

Province du Katanga
Division Provinciale des Mines
BUREAU DES MINES

STATISTIQUES D'EXPORTATIONS DES PRODUITS MINIERS
MOIS D'AVRIL 2012
(PRODUITS MINIERS PAR TONNES)

N°	SOCIETES	Conc cu	Conc. co	Conc. cuco	Cu. Noir	Cath.cu	All. Rouge	P.Zinc	Hydro. co	Cu.Pul	Co. El	Carb. co	P. Plomb	Nodul. cu	Conc. Etain	Conc. Tantal
1	AMCK					4000,11										
2	BOLFAST		416	134												
3	BOSS MINING		7109,4	3198,886		2868,75						69,852		67,034		
4	CDM		2391,9		3117	694,5			264							
5	CHEMAF					1124			153,75			423			50	
6	CDM															
7	CIMCO											99				
8	CONGO COPPER MILLS					240										
9	CONGO LOYAL				1759											
10	EXACO	648														
11	GCM					1369,78	120	1174,5			120		481,5			
12	GOLDEN AFRICAN					262,367				85,549						
13	HUACHIN				424											
14	KCC			3929,35		4453,45					155, 4					

N°	SOCIETES	Conc cu	Conc. co	Conc. cuco	Cu. Noir	Cath.cu	All. Rouge	P.Zinc	Hydro. co	Cu.Pul	Co. El	Carb. co	P. Plomb	Nodul. cu	Conc.Etain	Conc. Tantal
15	MAGMA				60											
16	METALS MINES		1325			75										
17	MMR														312	26
18	MUMI EXPORT			5425,29		6738,88			5273,89							
19	RUASHI MINING					3140			3448							
20	RUBAMIN				604,89											
21	SODIMICO		80		40											
22	SOMIKA		30		252,5	833			298							
23	TFM					11147,7			4709,26							
24	VOLCANO MINING	2830,4	3053													
**TOTAL**		**4749,47**	**14405**	**12553,5**	**6257**	**37081,5**	**120**	1174,5	**14146,9**	**85,549**	**275,4**	**591,85**	**481,5**	**67,034**	**362**	26

Fait à Lubumbashi, le 05/05 2012
Chef de Bureau Provincial des Mines
Pierrot MALOBA KITUMBA

République Démocratique du Congo

Province du Katanga
Division Provinciale des Mines
BUREAU DES MINES

STATISTIQUES D'EXPORTATIONS DES PRODUITS MINIERS
MOIS DE MAI 2012
(PRODUITS MINIERS PAR TONNES)

N°	SOCIETES	Conc cu	Conc. co	Conc. cuco	Cu. Noir	Cath.cu	All. Blanc	P.Zinc	Hydro. co	Cu.Pul	Co. El	Carb. co	P. Plomb	Nodul. cu	Conc.Eta in
1	AMCK					2895,2									
2	BOLFAST		800			96									
3	BOSS MINING		3422,8	5726,569		1957,5						48,628		67,034	
4	CDM		2543,1		2322,5	210			312						
5	CHEMAF					1318			558,75		12				
6	CDM														
7	CIMCO					688						64			
8	CONGO COPPER MILLS					190									
9	COPROCO														26
10	CONGO LOYAL				808										
11	EXACO	1062													
12	FEZA MINING						890								
13	GCM					1286,52		2110,8			70		671,02		
14	GOLDEN AFRICAN					240				32,5					

N°	SOCIETES	Conc cu	Conc. co	Conc. cuco	Cu. Noir	Cath.cu	All. Blanc	P.Zinc	Hydro. co	Cu.Pul	Co. El	Carb. co	P. Plomb	Nodul. cu	Conc.Etain
15	GTL						1394,495								
16	HUACHIN				648										
**17**	KCC			4468,512		3769,65					112,73				
18	MAGMA				468										
19	METALS MINES		1350			225									
20	MMR														234
21	MUMI EXPORT			4026,5		8250,55			5557,54						
22	RUASHI MINING					2251,5			3165						
23	SEK EXPORT	360													
24	RUBAMIN				691,63										
25	SODIMICO		40												
26	SOMIKA		1187		203	786			52						
27	TFM					13314,7			7754,88						
28	VOLCANO MINING	2714	1115		86										
**TOTAL**		**3 073,99**	**8705,07**	**17036,5**	**5227**	**37478,6**	**2284,49**	**2110,82**	**17400,2**	**32,5**	**194,73**	**112,63**	**671,02**	**67,034**	260

Fait à Lubumbashi, le 05/06/2012

Chef de Bureau Provincial des Mines

Pierrot MALOBA KITUMBA

République Démocratique du Congo

Province du Katanga
Division Provinciale des Mines
BUREAU DES MINES

STATISTIQUES D'EXPORTATIONS DES PRODUITS MINIERS
MOIS DE JUIN 2012
(PRODUITS MINIERS PAR TONNES)

N°	SOCIETES	Conc cu	Conc. co	Conc. cuco	Cu. Noir	Cath.cu	All.Bl	All. Rouge	P.Zinc	Hydro. co	Cu.Pul	Mat.cu	Co. El	Co-sep Mag	Carb. co	P. Plomb	Nodul. cu	Conc. Etain	Conc. Tantal
1	AMCK					3713,93													
2	BOLFAST		694			96													
3	BOSS MINING			9026,7		1890									55,428		200,86		
4	CDM		4010,8		3175	883				72		375,3							
5	CHEMAF					1966				267,75								25	
6	CIMCO					221									34				
7	CONGO COPPER MILLS					90					25								
8	CONGO LOYAL				1396														
9	FEZA MINING						260												
10	GCM					1401,6		180	1928,2		250		80	10		122			
11	GOLDEN AFRICAN					140					32,5								
12	GTL						3429,5												
13	HUACHIN				696														

N°	SOCIETES	Conc cu	Conc. co	Conc. cuco	Cu. Noir	Cath.cu	All.Bl	All. Rouge	P.Zinc	Hydro. co	Cu.Pul	Mat.cu	Co. El	Co-se Mag	Carb. co	P. Plomb	Nodul. cu	Conc. Etain	Conc. Tantal
14	KATANGA METALS				192														
15	KCC			4431,2		3577,41							223,09						
16	METALS MINES		825			75													
17	MMR																	260	17
18	MUMI EXPORT			2597,9		6129,95				2758,3									
19	RUASHI MINING					1777				3789									
20	SEK EXPORT	1586																	
21	RUBAMIN				117,94														
22	SODIMICO				80														
23	SOMIKA		1766,4		285,4	858				784,8									
24	TFM					10920,2				5507,5									
25	VOLCANO MINING	4151																	
**TOTAL**		**5737**	**7296,2**	**14793,475**	**5942,34**	**33739,1**	**3689,54**	**180**	**1928,28**	**13179,4**	**307,5**	**375,3**	**303,097**	**10**	**89,428**	**122**	**200,8**	**285**	**17**

Fait à Lubumbashi, le 05/07/ 2012
Chef de Bureau Provincial des Mines
Pierrot MALOBA KITUMBA

République Démocratique du Congo

Province du Katanga
Division Provinciale des Mines
BUREAU DES MINES

STATISTIQUES D'EXPORTATIONS DES PRODUITS MINIERS
MOIS DE JUILLET 2012
(PRODUITS MINIERS PAR TONNES)

N°	SOCIETES	Conc cu	Conc. co	Conc. cuco	Cu. Noir	Cath.cu	All.Bl	All. Rouge	P.Zinc	Hydro. co	Cu.Pul	Mat.cu	Co. El	Carb. co	Nodul. cu	Conc. Etain	Conc. Tantal
1	AMCK					4244,09											
2	BOLFAST		96		30	128											
3	BOSS MINING			9577,5		2261,25								60,968	235,2		
4	CDM		3160,1		2236,5	859				216		129,6					
5	CHEMAF					2410				1107,85						75	
6	CIMCO					826,872								648			
7	CONGO COPPER MILLS					35											
8	COPROCO															25	
9	CONGO LOYAL				1815												
10	GCM					1656,94		180	2130,9				50				
11	GOLDEN AFRICAN					70					52						
12	GTL						2163,927										

N°	SOCIETES	Conc cu	Conc. co	Conc. cuco	Cu. Noir	Cath.cu	All.Bl	All. Rouge	P.Zinc	Hydro. co	Cu.Pul	Mat.cu	Co. El	Carb. co	Nodul. cu	Conc.Etain	Conc.Tantal
13	HUACHIN				856												
**14**	**HUACHIN METAL LEACH**					**94,02**											
15	KATANGA METALS				15O												
16	KCC			2771,022		3455,92							158,0975				
17	METALS MINES		875			125											
18	MMR															234	14
19	MUMI EXPORT			2689, 34		7468,13				5015,22							
20	RUASHI MINING					2555,5				3495							
21	SEK EXPORT	4793															
22	RUBAMIN				1304,45												
23	SODIMICO				240												
24	SOMIKA		634,8		312	746				574,4							
25	TFM					14719,2				8168,72							
26	VOLCANO MINING	1550															
**TOTAL**		**6 353**	**4765,9**	**15037,8**	**6943,95**	**41654,9**	**2163,92**	**180**	**2130,9**	**18577,2**	**52**	**129,6**	**208,098**	**708,9**	**235,2**	**333**	**14**

Fait à Lubumbashi, le 05/08/ 2012

Chef de Bureau Provincial des Mines

Pierrot MALOBA KITUMBA

République Démocratique du Congo

Province du Katanga
Division Provinciale des Mines
BUREAU DES MINES

STATISTIQUES D'EXPORTATIONS DES PRODUITS MINIERS
MOIS D'AOUT 2012
(PRODUITS MINIERS PAR TONNES)

N°	SOCIETES	Conc cu	Conc.co	Conc. cuco	Cu. Noir	Cath.cu	All.Bl	All. Rouge	P.Zin	Mat. cu	Hydro. co	Cu.Pul	Carb.Co	Carb.Cu	P. Plomb	Nodul. cu	Co.El	Co-sep	Conc. Etain	Conc. Tantal
1	AMCK (MMG)					3801,64														
2	BAZANO		2416,1				838													
3	BOLFAST		1922			32														
4	BOSS MINING			9002,72		1687,5							53,593			904,39				
5	CDM		4477,8		3383,5	912				356	120									
6	CHEMAF					1824					318									
7	CIMCO					339							478							
8	CONGO LOYAK				795															
9	CONCORDE POUR L'INDUSTRIE													231						
10	FEZA MINING						99													
11	GCM					861,128		180	1600,82			300	70		289		100	10		
12	GOLDEN AFRICAN										58,5									
13	GTL						2162,03													
14	HUACHIN				836															

N°	SOCIETES	Conc cu	Conc.co	Conc. Cuco	Cu. Noir	Cath.cu	All.Bl	All. Rouge	P.Zin	Mat. cu	Hydro. co	Cu.Pul	Carb.Co	Carb.Cu	P. Plomb	Nodul. cu	Co.El	Co-sep	Conc. Etain	Conc.T antal
15	HUACHIN METAL LEACH					2544,4														
16	KCC			4362,66		3948,93										119,08	112,957			
17	MAGMA				300															
18	METALS MINES		575			325														
19	MMR																		390	12
20	MUMI EXPORT			3158,76		6710,58					2914,91									
21	RUASHI MINING					3631,5					2687									
22	SEK EXPORT	3852																		
23	RUBAMIN				122,063															
24	SODIMICO		210		240															
25	SOMIKA				438,5	486					202									
26	TFM					14195,1					6085,06									
27	VOLCANO MINING	1480	2351																	
**TOTAL**		**5332**	**11952**	**16524,147**	**6115,06**	**41298,8**	**3099,035**	**180**	**1600,8**	**356,4**	**12385,5**	**300**	**601,6**	**231**	**289**	**1023,5**	**212,957**	**10**	**390**	**12**

Fait à Lubumbashi, le 05/09/ 2012

Chef de Bureau Provincial des Mines

Pierrot MALOBA KITUMBA

République Démocratique du Congo

Province du Katanga
Division Provinciale des Mines
BUREAU DES MINES

STATISTIQUES D'EXPORTATIONS DES PRODUITS MINIERS
MOIS DE SEPTEMBRE 2012
(PRODUITS MINIERS PAR TONNES)

N°	SOCIETES	Conc cu	Conc.co	Conc. cuco	Cu. Noir	Cath.cu	All.Bl	All. Rouge	P.Zin	Mat. cu	Hydro. co	Cu.Pul	Carb.Co	Carb.Cu	P. Plomb	Nodul. cu	Co.El	Co-sep	Conc.Etain	Conc.Tantal
1	AMCK (MMG)					3190,52														
2	BAZANO		2689,6		343,05															
3	BOLFAST		1136			30														
4	BOSS MINING			6990,74		1350							11,308			671,23				
5	CAM RESSOURCES					90														
6	CDM		3984,7		2462	804				97,2	198									
7	CHEMAF					1762					330									
8	CIMCO					275														
9	CMSK			1066,8																
10	CONGO LOYAK				1076															
11	CONCORDE POUR L'INDUSTRIE													752						
12	GCM					1851,81		60	1459,5				30		474,5		110	10		
13	GOLDEN AFRICAN					465						125,2								
14	GTL						2958,80													

N°	SOCIETES	Conc cu	Conc.co	Conc. cuco	Cu. Noir	Cath.cu	All.Bl	All. Rouge	P.Zin	Mat. cu	Hydro. co	Cu.Pul	Carb.Co	Carb.Cu	P. Plomb	Nodul. cu	Co.El	Co-sep	Conc. Etain	Conc.T antal
15	HUACHIN				640															
16	HUACHIN METAL LEACH					1039,66														
17	KCC			10439,1		4640,79											180,795			
18	MAGMA				60															
19	METALS MINES		825			200														
20	MMR																		260	40
21	MUMI EXPORT			2172,33		6697,96					2772,41									
22	SINO KATANGA (STK)																		22,27	
23	SHITURU MINING					1169,29														
24	RUASHI MINING					1471					3357									
25	SEK EXPORT	2670																		
26	RUBAMIN				1145,03															
27	SODIMICO				160															
28	SOMIKA		335,4		281	410					186									
29	TFM					12566,3					4093,55									
30	VOLCANO MINING	720	224																	
**TOTAL**		**3389,9**	**9194,7**	**20668,997**	**6167,1**	**38013,4**	**2958,809**	**160**	**1459,5**	**97,2**	**10937**	**125,2**	**41,308**	**752**	**474,5**	**671,23**	**290,795**	**10**	**282,27**	**40**

Fait à Lubumbashi, le 05/10/ 2012

Chef de Bureau Provincial des Mines

Pierrot MALOBA KITUMBA

République Démocratique du Congo

Province du Katanga
Division Provinciale des Mines
BUREAU DES MINES

STATISTIQUES D'EXPORTATIONS DES PRODUITS MINIERS
MOIS D'OCTOBRE 2012
(PRODUITS MINIERS PAR TONNES)

N°	SOCIETES	Conc cu	Conc. co	Conc. cuco	Cu. Noir	Cath.cu	All.Bl	All. Rouge	P.Zinc	Hydro. Co	Cu. Pul	Mat.cu	Co. El	Co-sep Mag	Anod.Sol	P. Plomb	Nodul. cu	Conc. Etain	Conc. Tantal
1	AMCK (MMG)					2666,33													
2	BAZANO				135,82														
3	BOLFAST		456			32													
4	BOSS MINING			4022,64		1687,5											603,63		
5	CDM		2369,8		3568,5	852				378		129,6							
6	CHEMAF					1339				495			10						
7	CIMCO					942													
8	CMSK			1308,56															
9	CONGO LOYAL				632														
10	FEZA MINING						384												
11	GCM					2667,93		180	1366,5				110	10	120,04	641			
12	GOLDEN AFRICAN					195,5					50								
13	GTL						3429,5												
14	HUACHIN				408														

N°	SOCIETES	Conc cu	Conc. co	Conc. cuco	Cu. Noir	Cath.cu	All.Bl	All. Rouge	P.Zinc	Hydro. Co	Cu. Pul	Mat.cu	Co. El	Co-se Mag	Anod.Sol	P. Plomb	Nodul. cu	Conc. Etain	Conc. Tantal
**15**	**HUACHIN METAL LEACH**					**1200,43**													
16	KATANGA METALS				187														
17	KCC			16589,46		3387,77							178,22				3012,6		
18	METALS MINES		475			175													
19	OM METAL					83,38													
20	MMR																	390	18
21	MUMI EXPORT			1814,21		8960,2				3495,95									
22	SHITURU MINING					2933,45													
23	RUASHI MINING					3310				3594,08									
24	SEK EXPORT	675																	
25	RUBAMIN				1714,56														
26	SODIMICO				320														
27	SOMIKA		297,7		336	312				384									
28	TFM					14645,9				3818,88									
29	VOLCANO MINING	800	384																
**TOTAL**		**1475**	**3964,5**	**23734,8**	**7301,88**	**45390,3**	**1479,7**	**180**	**1366,5**	**12165,9**	**50**	**129,6**	**298,2**	**10**	120,04	**641**	**3616,2**	**390**	**18**

Fait à Lubumbashi, le 05/11/ 2012
Chef de Bureau Provincial des Mines
Pierrot MALOBA KITUMBA

République Démocratique du Congo

Province du Katanga
Division Provinciale des Mines
BUREAU DES MINES

STATISTIQUES D'EXPORTATIONS DES PRODUITS MINIERS
MOIS DE NOVEMBRE 2012
(PRODUITS MINIERS PAR TONNES)

N°	SOCIETES	Conc cu	Conc. co	Conc. cuco	Cu. Noir	Cath.cu	All.Bl	All. Rouge	P.Zinc	Hydro. co	Cu.Pul	Mat.cu	Co. El	Co-sep Mag	P. Plomb	Nodul. cu	Conc. Etain	Conc. Tantal
1	AMCK (MMG)					3367,82												
2	BAZANO		1594,6															
3	BOLFAST		450			61												
4	BOSS MINING		323,66	11778,48		1957,5										235,19		
5	CDM		1600,3		58	1039				390		129,6						
6	CHEMAF					1376				565,5								
7	CIMCO					857												
8	CMSK			4444,8														
9	CONGO LOYAL				606													
10	FRONTIER	2000																
11	EXACO	280																
12	GCM				2862	2012,6		60	288,66				50	10	332,5			
13	GOLDEN AFRICAN					100					50,8							
**14**	**GTL**						756,11											
15	HUACHIN				624													
16	HUACHIN METAL LEACH					238,546												

N°	SOCIETES	Conc cu	Conc. co	Conc. cuco	Cu. Noir	Cath.cu	All.Bl	All. Rouge	P.Zinc	Hydro. co	Cu.Pul	Mat.cu	Co. El	Co-sep Mag	P. Plomb	Nodul. cu	Conc. Etain	Conc. Tantal
17	LOMAMINE																25	
18	KATANGA METALS				180													
19	KCC			12711		1618,61							245,44			7113,3		
20	METALS MINES		775			150												
21	MMR																130	17
22	MUMI EXPORT			1485,4		7494,94				2999,1								
23	OM METAL					59,84												
24	RUASHI MINING					1375				1018								
25	RUBAMIN				639,27													
26	SEK EXPORT	1821,1																
27	SINO KATANGA (STK)																23,41	
28	SHITURU MINING					1449,35												
29	SODIMICO		210		240													
30	SOMIKA				87	676				396								
31	TFM					11756,9				3915,6								
32	VOLCANO MINING	2440	1628															
**TOTAL**		**6591,17**	**19293**	**17709**	**5296,33**	**35590,1**	**756,1**	**60**	**288,6**	**9284,33**	**50,8**	**129,6**	**295,442**	**10**	**332,5**	**7348,4**	**178,41**	**17**

Fait à Lubumbashi, le 05/11/ 2012

Chef de Bureau Provincial des Mines

Pierrot MALOBA KITUMBA

République Démocratique du Congo

Province du Katanga
Division Provinciale des Mines
BUREAU DES MINES

STATISTIQUES D'EXPORTATIONS DES PRODUITS MINIERS
MOIS DE DECEMBRE 2012
(PRODUITS MINIERS PAR TONNES)

N°	SOCIETES	Conc cu	Conc. co	Conc. cuco	Cu. Noir	Cath.cu	All.Bl	All. Rouge	P.Zinc	Hydro. Co	Cu.Pul	Mat.cu	Co. El	Co-sep Mag	Anod. Sol	P. Plomb	Nodul. cu	Conc. Etain	Conc. Tantal
1	AMCK (MMG)					4896,13													
2	BAZANO		1837		406														
3	BOSS MINING			9139,221		1248,75											134,5		
4	CAM RESSOURC ES					129,36													
5	CDM		2062			673				540		129,6							
6	CHEMAF					1350				310,5								13	
7	CIMCO					736													
8	CMSK			4604,686															
9	FEZA MINING						160												
10	GCM				2358,04	1280,25		120	1322,3		300		50	10	59,9	233	68,943		
11	GOLDEN AFRICAN					277					80								
12	GTL						743,193												
13	HUACHIN				288														
14	MINES D'AFRIQUE																	45,7	
15	HUACHIN METAL LEA				330,38														

N°	SOCIETES	Conc cu	Conc. co	Conc. cuco	Cu. Noir	Cath.cu	All.Bl	All. Rouge	P.Zinc	Hydro. Co	Cu.Pul	Mat.cu	Co. El	Co-se Mag	Anod. Sol	P. Plomb	Nodul. cu	Conc. Etain	Conc. Tantal
**16**	**KCC**			**11520,5**		**1996,59**							**157,3**				**9495,9**		
17	METALS MINES		625			75													
18	MMR																	312	13
19	MUMI EXPORT			1599,43		8520,79				2998,87									
20	SHITURU MINING					1269,45													
21	RUASHI MINING					1587				3042									
22	SEK EXPORT	988,62																	
23	RUBAMIN				697,408														
24	SODIMICO				160														
25	SOMIKA		56			234				234									
26	TFM					16337,9				3088,84									
27	VOLCANO MINING	1850,5	1276																
**TOTAL**		**2849**	**5856**	**26863,8**	**4239,82**	**40611,2**	**903,193**	**120**	**1322,32**	**10214,2**	**380**	**129,6**	**207,35**	**10**	**59,9**	**233**	**9699,3**	**370,7**	**13**

Fait à Lubumbashi, le 05/01/ 2013

Chef de Bureau Provincial des Mines

Pierrot MALOBA KITUMBA

République Démocratique du Congo

PROVINCE DU KATANGA
DIVISION PROVINCIALE DES MINES
LUBUMBASHI
Le Chef de Division

STATISTIQUES DES NOTES DE DEBIT RELATIVES A LA REDEVANCE MINIERE EMISES DE JANVIER A DECEMBRE/2013

Produits Miniers par tonnes

N°	SOCIETES	MONTANTS REDEVANCE MINIERE (USD)	Conc.Cu (10-30%)	Conc.Co (4-15%)	Conc.CuCo	Cu Noir (80-98%)	Cath.Cu (99%)	All.Blanc (30%Co ≤ 30%Cu≤	All.Rouge (±80% Cu	P-Zinc (89%Zn)	Mat-Cu (±40%)	Hetero Co (Brut)	Min Cu (Brut)	Hydro.Co (29%)	Cu.Pul (Cu 95%)	Cu.Bott (Cu 97%)	P.Plomb (P 30%)	Carb.Co (±25%)	Carb.Cu (±25%)	Nod.Cu (±97%)	Co.Electro (Co 99%)	Co-sep Mag (Co 55,25%)	Wire bars (Cu98,62 %)	Anod.Sol	Scraps (Cu±30 %)	Sable.Cobel	Conc.Etain	Conc.Tantal
1	ANVIL MINING CONGO (CMCC)																											
2	BAZANO	205 058,84		3 272,98	0,00	802,25																						
3	BAZANO VENTE LOCALE	2 508,77		1 466,43											30,00													
4	BOLFAST	241 056,58		4 626,00		53,00	960,00																					
	BOSS MINING	7 555 251,66		26 395,87	96 648,37	0,00	28 984,94											80,24		803,01								
5	CAM RESSOURCES	22 177,01					187,98																					
6	CDM	4 379 740,54		29 041,85	0,00	27 704,61	11 422,62	0,00	0,00	0,00	1 941,10	0,00	0,00	3 043,60														
	CEPRODEV	19 744,64		0,00			0,00							0,00							0,00						130,00	0,00
7	CHEMAF	2 786 125,15					19 139,00							6 139,50							10,16						127,00	31,00
8	CIMCO	1 161 877,95					10 216,00																					
9	EAGLE GROUP/EXPORT	12 714,77					0,00																					
10	CMSK	1 146 022,87			42 645,71	0,00	0,00																					
11	CCC VENTE LOCAL	201 107,55											10 598,22															
12	COMIDE/VENTE LOCALE	533 941,57	10 285,53																									
13	CONCORDE POUR L'INDUSTRIE	31 054,00	0,00																1 920,00									
14	CONGO COPPER MILLS	72 612,74	0,00				554,00																					
15	CONGO LOYAL	389 980,80	0,00		0,00	4 562,00	0,00																					
16	COPROCO	16 328,96	0,00		0,00	0,00	0,00																				94,80	
	EAGLE GROUP	81 722,86	3 712,70	4 645,88																							0,00	
17	EXACO	3 427,34	404,02																									
18	FEZA MINING	223 369,31	0,00					1 910,81																				
19	FRONTIER	6 805 677,73	230 799,52																									
20	GCM	4 580 705,48				16 071,94	15 456,37		1 023,34	18 479,92					1 085,05	0,00	2 179,00			495,43	470,00	30,00		277,81		170,00		
21	CONGO JIN JU CHENG	141 538,90					1 395,48																					
22	GOLDEN AFRICAN	326 747,84					2 577,00								428,00													
	GROUPE LA BONNE CONFIANCE	14 958,32																									91,50	
23	GTL	0,00						23 613,85																				
24	HUACHIN	514 022,17				5 160,00																						
25	HUACHIN METAL LEACH	1 083 628,27					10 405,85																					
26	BOSS MINING/VENTE LOCALE	2 476,50		338,97																							0,00	0,00

N°	SOCIETES	REDEVANCE MINIERE (USD)	Conc Cu (10-30%)	Conc.Co (4-15%)	Conc CuCo	Cu Noir (80-98%)	Cath.Cu (99%)	[illegible] (30%Co ≤ 30%Cus	All.Rouge (±80% Cu	P-Zinc (89%Zn)	Mat-Cu (±40%)	Hetero Co (Brut)	Min Cu (Brut)	Hydro.Co (29%)	Cu Pul (Cu 95%)	Cu.Bott (Cu 97%)	P.Plomb (P 30%)	Carb.Co (±25%)	Carb.Cu (±25%)	Nod.Cu (±97%)	Co.Electro (Co 99%)	Co-sep Mag (Co 55,25%)	Wire bars (Cu98,62 %)	Anod.Sol	Scraps (Cu±30 %)	Sable Cobel	Conc.Etain	Conc Tantal
27	RUASHI MINING/VENTE LOCALE	2 614,67					37,00																					
28	KATANGA METALS	85 667,58			0,00	962,00																					0,00	0,00
29	KCC	16 311 876,49			135 002,57	0,00	83 406,63								798,54					159,37	223,06							
30	KISANFU MINING/VENTE LOCALE	152 030,49													526,24					3 572,90	2 124,62							
31	LOMAMINES	12 410,00											18 617,80															
32	LONG FEI/VENTE LOCALE	12 237,69																									75,00	
33	LUNA MINING/VENTE LOCALE	314 015,40	44 225,87										15 078,04															
34	MIKAS/VENTE LOCALE	164 760,15	16 519,28	0,00	0,00																							
35	METALS MINES	709 588,26		14 300,00			2 900,00																					
36	MJM	58 555,65		2 290,18																								
37	MMG KINSEVERE	7 796 057,16					60 188,48																					
38	OPERA MINING	27 629,80																										
39	MMR	539 680,74																										
40	MUMI EXPORT	20 890 235,73			20 147,17		142 484,81																				3 102,00	243,11
41	MAGMA	52 203,90				642,00								51 581,32														
42	OM METAL	21 102,28					209,52																					
43	RUASHI MINING	6 366 283,69				0,00	39 769,55																					
44	RUBAMIN	1 436 529,91				15 073,27								22 721,99														
45	MKM	1 486 216,18					14 277,93																					
46	SEK EXPORT	1 566 264,91	54 580,00																									
47	SEK VENTE LOCAL	1 824 145,53	140 662,43																									
48	SHITURU MINING	2 711 142,23	0,00				22 636,01																					
49	SINO KATANGA (STK)	30 349,08																										
50	SMKK VENTE LOCALE	20 110,66																									171,45	
51	SODIMICO	201 592,49		1 085,00	980,00	1 320,00							1 082,42														0,00	
52	MINES D'AFRIQUE	42 801,92																										
53	SOMIKA	~1 067 585,04		949,90		453,00	7 168,00							1 968,10													281,00	
54	TFM	29 591 547,34		0,00		0,00	210 115,69							55 982,04														
55	VOLCANO MINING	10 270,26		462,00																								
56	TOUS AZIMUTS	1 133,45																										
57	PANCOM	14 872,27				150,41																						
TOTAL		126 077 090,11	501189,3	88875,1	295423,8	72954,5	684492,8	25524,7	1023,3	18479,9	1941,1	0,0	45376,5	141436,6	2867,8	0,0	2179,0	80,2	1920,0	5030,7	2827,8	30,0	0,0	277,8	0,0	170,0	4072,8	274,1

Le total général est de: Cent vingt six millions septante sept mille nonante dollars, onze

NB: 115,3 Tonnes de concentrés de wolframite exporté par MMR
NB: 230 Tonnes de Sulfure de Denickelage exporté par GCM
NB: 5.383,92 Tonnes de Plomb Affiné à 95% exporté par KCC
NB: 1.326 gr d'OR exporté par le Comptoir DEVELOPPEMENT TOUS ASIMUTS

Légende: Concentré de cuivre: (Conc.cu), concentré de cobalt : (Conc.co), concentré cupro-cobaltifère: (Conc.CuCo); Cathode de cuivre: (Cath.Cu); Matte de cuivre: (Matt.Cu); Hydroxyde de Cobalt: (Hydro.Co); Carbonate de cobalt (Carb.Co); Poussière de Zinc: (Pouss.Zinc); Poussière de Plomb: (P.Plomb); Nodule du cuivre :(Nod.Cu); Alliage Rouge: (All.Rouge), Alliage Blanc : (All.Blanc), Cuivre Pulverisé: (Cu.Pul); Cuivre Bottom: (Cu.Bott); Hétérogénite de cobalt: (Hetero.Co); Minerais de cuivre: (Min.Cu), Co en Granule: Cobalt en Granule; Cassi: Cassitérite

Fait à Lubumbashi, le 06 Janvier 2014
Emmanuel KIANDA KIBAWA

République Démocratique du Congo

PROVINCE DU KATANGA
DIVISION PROVINCIALE DES MINES
LUBUMBASHI
Le Chef de Division

STATISTIQUES DES NOTES DE DEBIT RELATIVES A LA REDEVANCE MINIERE EMISES DE JANVIER A DECEMBRE/2014

Produits Miniers par tonnes

N°	SOCIETES	MONTANTS REDEVANCE MINIERE (USD)	Conc.Cu (10-30%)	Conc.Co (4-15%)	Conc.CuCo	Cu.Noir (80-98%)	Cath.Cu (99%)	All.Blanc (30%Co ≤ 30%Cu≤	All.Rouge (±80% Cu	P-Zinc (89%Zn)	Mat-Cu (±40%)	Hetero Co (Brut)	Min Cu (Brut)	Hydro.Co (29%)	Cu.Pul (Cu 95%)	Cu.Bott (Cu 97%)	P.Plomb (P 30%)	Carb.Co (±25%)	Carb.Cu (±25%)	Nod.Cu (±97%)	Co.Electro (Co 99%)	Co-sep Mag (Co 55,25%)	Sable. Cobel	Anod.Sol	Scraps (Cu±30 %)	Conc.Wolframite	Conc.Etain	Conc.Tantal
1	ANVIL MINING CONGO (CMCC)	0,00																										
2	BAZANO	0,00																										
3	BAZANO VENTE LOCALE	0,00																										
4	BOLFAST	189 346,92	0,00	5 273,00	0,00	0,00	240,00										0,00	0,00	0,00	0,00	0,00	0,00	0,00					
5	BOSS MINING	7 514 658,86	0,00	3 311,25	117 487,38		31 517,50										0,00	647,65										
6	CAM RESSOURCES	0,00	0,00																									
7	CCC/VENTE LOCALE	161 818,42	827,09								0,00		8 843,00	0,00														
8	CDM	6 203 849,71	0,00	62 933,35	0,00	33 909,40	11 446,97				4 063,50		0,00	3 561,00														
9	CEPRODEV	3 179,53					0,00																				25,00	
10	CHEMAF	3 200 262,19					15 514,00							9 420,75													188,00	79,00
11	CIMCO	1 203 923,00					11 392,00							0,00														
12	COMILU	1 226 460,02					12 399,51																					
13	CMSK	1 185 573,80			41 652,61																							
14	CONGO JIN JU HENG	388 721,54	0,00				3 857,68																					
15	COMIDE/VENTE LOCALE	767 190,55	19 951,05				0,00																					
16	CONCORDE POUR L'INDUSTRIE	0,00	0,00				0,00																					
17	CROWN MINING	9 595,84																									52,00	
18	CONGO LOYAL	0,00																										
19	COPROCO	0,00																										
20	EAGLE GROUP	2 138,24		539,82									1 011,32															
21	DRAGON INTERNATIONAL	47 079,74				599,79																						
22	FEZA MINING	355 373,21		0,00				2 730,00																				
23	FRONTIER	8 170 225,59	291 780,46	0,00				0,00																				
24	GCM	2 316 735,65	4 237,00	0,00	0,00	3 108,32	11 558,56	0,00	840,00	19 430,68					300,00	0,00	2 680,90	125,02		122,16	75,00	10,00	21,00	63,97				
25	CNMC HUACHIN MINING MABENDE	1 358 879,75					12 869,94																					
26	GOLDEN AFRICAN	407 866,92					3 870,00																					
27	GROUPE LA BONNE FIANCE	25 132,40						0,00																			177,00	
28	GTL	0,00						27 518,42																			0,00	
29	HUACHIN METAL LEACH	847 948,56					7 980,84	0,00																				
30	HUACHIN MINING	803 805,43		2 425,00		7 024,18																						
31	KAI PENG MINING	570 696,04					5 538,23																					
32	KATANGA METALS	70 685,99				800,00															0,00							0,00
33	KCC	20 506 681,67				0,00	150 121,02	0,00												183,87	2 854,55							

N°	SOCIETES	MONTANTS REDEVANCE MINIERE (USD)	Conc.Cu (10-30%)	Conc.Co (4-15%)	Conc.CuCo	Cu.Noir (80-98%)	Cath.Cu (99%)	All.Blanc (30%Co ≤ 30%Cu≤	All.Rouge (±80% Cu	P-Zinc (89%Zn)	Mat-Cu (±40%)	Hetero Co (Brut)	Min Cu (Brut)	Hydro.Co (29%)	Cu.Pul (Cu 95%)	Cu.Bott (Cu 97%)	P.Plomb (P 30%)	Carb.Co (±25%)	Carb.Cu (±25%)	Nod.Cu (±97%)	Co.Electro (Co 99%)	Co-sep Mag (Co 55,25%)	Sable. Cobel	Anod.Sol	Scraps (Cu±30 %)	Conc.Wolframite	Conc.Etain	Conc.Tantal
34	KISANFU MINING/VENTE LOCALE	388 147,31	29 840,27																		0,00							
35	MAGMA	91 335,45				1 156,44																						
36	LONG FEI/VENTE LOCALE	1 392,57	0,00																									
37	LUNA MINING/VENTE LOCALE	282 283,12	41 259,00	0,00			0,00																					
38	MABENDE MINING	668,12	112,56	0,00																								0,00
39	METALS MINES	1 246 128,52	0,00	27 375,00			3 160,00																					
40	MINES D'AFRIQUE	52 872,04	0,00	0,00			0,00																			0,00	348,55	
41	MIKAS VENTE LOVALE	320 715,33	72 580,34																									
42	MJM	378 736,57	0,00	5 400,00	0,00	0,00	2 129,00																					
43	MKM	2 620 662,62	0,00	0,00	0,00	0,00	25 117,94	0,00	0,00	0,00	0,00	0,00	0,00	509,99														
44	MMG KINSEVERE	8 328 548,17					67 017,07							0,00														
45	MMR	509 319,50					0,00							0,00												17,50	3 041,81	208,15
46	MUMI EXPORT	28 042 847,37	801,96	0,00	10 959,95		196 332,62							52 009,85	0,00					1 524,87								0,00
47	OPERA MINING	84 279,90												0,00													468,00	
48	OM METAL	24 200,04				0,00	209,52																					
49	PANCOM	404 320,80				3 728,68																						
50	RUASHI MINING	5 832 980,77				0,00	36 487,27							17 492,74														
51	RUBAMIN	1 184 840,58				12 679,79	0,00							0,00														
52	MUMI/VENTE LOCALE	75 859,37			9 554,23									0,00														
53	SEK EXPORT	2 116 666,58	30 754,57				11 955,06							0,00														
54	SEK VENTE LOCAL	0,00	0,00											0,00														
55	SHITURU MINING	3 445 281,01					30 436,55							0,00													0,00	
56	SINO KATANGA (STK)	60 316,51																									374,57	
57	SMKK VENTE LOCALE	0,00																									0,00	
58	SODIMICO	56 412,80				640,00																						
59	SODIMICO/VENTE LOCALE	0,00																										
60	SOMIKA	1 953 026,15					12 114,00							4 746,70														
61	TFM	30 246 992,28					203 329,72							69 189,64														
62	TOUS AZIMUTS	0,00												0,00														
TOTAL		*145 286 663,07*	*492144,302*	*107257,4*	*179654,16*	*63646,61*	*866595*	*30248,42*	*840*	*19430,68*	*4063,5*	*0*	*9854,317*	*156930,7*	*300*	*0*	*2680,9*	*772,67*	*0*	*1830,893*	*2929,55*	*10*	*21*	*63,97*	*0*	*17,5*	*4674,93*	*287,15*

Le total général est de: Cent quarante cinq millions deux cent quatre vingt six mille six cent soixante trois dollars, sept centimes

Légende: Concentré de cuivre: (Conc.cu); concentré de cobalt : (Conc.co); concentré cupro-cobaltifère: (Conc.CuCo); Cathode de cuivre: (Cath.Cu); Matte de cuivre: (Matt.Cu); Hydroxyde de Cobalt: (Hydro.Co); Carbonate de cobalt: (Carb.Co); Poussière de Zinc: (Pouss.Zinc); Poussière de Plomb: (P.Plomb); Nodule du cuivre :(Nod.Cu); Alliage Rouge: (All.Rouge); Alliage Blanc : (All.Blanc); Cuivre Pulverisé: (Cu.Pul); Cuivre Bottom: (Cu.Bott); Hétérogénite de cobalt: (Hetero.Co); Minerais de cuivre: (Min.Cu), Co en Granule: Cobalt en Granule; Cassi: Cassitérite

Fait à Lubumbashi, le 02 Janvier 2015
Emmanuel KIANDA KIBAWA

République Démocratique du Congo

PROVINCE DU KATANGA
DIVISION PROVINCIALE DES MINES
LUBUMBASHI
Le Chef de Division

STATISTIQUES DES NOTES DE DEBIT RELATIVES A LA REDEVANCE MINIERE EMISES MOIS DE JANVIER /2015

Produits Miniers par tonnes

N°	SOCIETES	MONTANTS (USD)	Conc.Cu (10-30%)	Conc.Co (4-15%)	Conc.CuCo	Cu.Noir (80-98%)	Cath.Cu (99%)	All.Blanc (30%Co ≤ 30%Cu≤	All.Rouge (±80% Cu	P-Zinc (89%Zn)	Mat-Cu (±40%)	Hetero Co (Brut)	Min Cu (Brut)	Hydro.Co (±29%)	Cu.Pul (Cu 95%)	Cu.Bott (Cu 97%)	P.Plomb (P 30%)	Carb.Co (±25%)	Carb.Cu (±25%)	Nod.Cu (±99%)	Co.Electro (Co 99%)	Co-sep Mag (Co 55,25%)	Sable. Cobel	Anod.Sol	Scraps (Cu±30%)	Co en Granule (96%)	Conc
1	ANVIL MINING CONGO (CMCC)																										
2	BOLFAST	6770,05		215																							
3	BOSS MINING	704123,18			11307,355		2908,75											137,04									
4	CAMIS																										
5	CCC/VENTE LOCALE	16603,59										380,9	131,14														
6	CDM	491908,51		5115,15		2111,826	1223,503				62,1			495,6													
7	CEPRODEV																										
8	CHEMAF	393917,72					2464							850,5													
9	CIMCO	69791,80					763																				
10	CMSK	124255,34			4598,317																						
11	CNMC HUACHIN MINING MABENDE	212323,46					1917,678																				
12	COMIDE/VENTE LOCALE	120216,67	3471,37																								
13	COMILU	91936,48					901,338																				
14	CONGO JIN JU CHENG	18698,11					186,2905																				
15	CROWN MINING																										
16	DRAGON INTERNATIONAL	18737,40				220																					
17	EAGLE GROUP																										
18	FEZA MINING	58215,55						384																			
19	FRONTIER	1163793,52	46113																								
20	GCM	239864,37				73,697	1595,707			1156,5							269,5				70						
21	GOLDEN AFRICAN	28006,80					314																				
22	GROUPE LA BONNE FIANCE																										
23	GTL							1627,76																			
24	HUACHIN METAL LEACH	108093,67					977,418																				
25	HUACHIN MINING	43154,48		1450																							
26	KAI PENG MINING	82311,04					774,306																				
27	KATANGA METALS																										
28	KCC	1704938,92					12381,53													33,616	416						
29	KISANFU MINING/VENTE LOCALE	41133,36											9142,8														
30	LONG FEI/VENTE LOCALE																										

N°	SOCIETES	MONTANTS (USD)	Conc.Cu (10-30%)	Conc.Co (4-15%)	Conc.CuCo	Cu.Noir (80-98%)	Cath.Cu (99%)	All.Blanc (30%Co ≤ 30%Cu≤	All.Rouge (±80% Cu	P-Zinc (89%Zn)	Mat-Cu (±40%)	Hetero Co (Brut)	Min Cu (Brut)	Hydro.Co (±29%)	Cu.Pul (Cu 95%)	Cu.Bott (Cu 97%)	P.Plomb (P 30%)	Carb.Co (±25%)	Carb.Cu (±25%)	Nod.Cu (±99%)	Co.Electro (Co 99%)	Co-sep Mag (Co 55,25%)	Sable Cobel	Anod.Sol	Scraps (Cu±30%)	Co en Granule (96%)	Conc
31	LUNA MINING/VENTE LOCALE	16771,05	2292,94																								
32	MAGMA																										
33	METALS MINES	136050,94		3325			375																				
34	MIKAS VENTE LOVALE	28409,78	8333,49																								
35	MINES D'AFRIQUE																										
36	MJM	32347,34					333																				
37	MKM	263369,40					2413,176							135,826													
38	MMG KINSEVERE	764394,31					7456,255																				
39	MMR	43360,20																									
40	MUMI EXPORT	2259434,55					15813,6							2888,9						2110,61							
41	MUMI/VENTE LOCALE																										
42	OM METAL																										
43	OPERA MINING	4836,60																									
44	PANCOM	45948,10				474,254																					
45	RUASHI MINING	465019,54					2980,147							1649,333													
46	RUBAMIN	118338,09				1372,857																					
47	SEK	327679,87	2062,5				2706,342																				
48	SHITURU MINING	342616,01					3495,771																				
49	SINO KATANGA (STK)	7475,47																									50
50	SODIMICO																										
51	SOMIKA	164812,44					1202							314													
52	TFM	3364810,16					18947,08							7568,093													
	RUASHI MINING VENTE LOCALE																										
	SEK VENTE LOCALE																										
TOTAL		*14 124 467,87*	*62273,30*	*10105,15*	*15905,67*	*4252,63*	*82129,89*	*2011,76*	*0,00*	*1156,50*	*62,10*	*380,90*	*9273,89*	*13902,25*	*0,00*	*0,00*	*269,50*	*137,04*	*0,00*	*2144,23*	*486,00*	*0,00*	*0,00*	*0,00*	*0,00*	*0,00*	*30*

Le total général est de: Quatorze millions cent vingt quatre mille quatre cent soixante sept dollars, quatre vingt sept centimes

N.B:

Légende: Concentré de cuivre: (Conc.cu); concentré de cobalt : (Conc.co); concentré cupro-cobaltifère: (Conc.CuCo); Cathode de cuivre: (Cath.Cu); Matte de cuivre: (Matt.Cu); Hydroxyde de Cobalt: (Hydro.Co); Carbonate de cobalt: (Carb.Co); Poussière de Zinc: (Pouss.Zinc); Poussière de Plomb: (P.Plomb); Nodule du cuivre :(Nod.Cu); Alliage Rouge: (All.Rouge); Alliage Blanc : (All.Blanc); Cuivre Pulverisé: (Cu.Pul); Cuivre Bottom: (Cu.Bott); Hétérogénite de cobalt: (Hetero.Co); Minerais de cuivre: (Min.Cu)

Fait à Lubumbashi, le 07 Février 2015

Emmanuel KIANDA KIBAWA

République Démocratique du Congo

PROVINCE DU KATANGA
DIVISION PROVINCIALE DES MINES
LUBUMBASHI
Le Chef de Division

STATISTIQUES DES NOTES DE DEBIT RELATIVES A LA REDEVANCE MINIERE EMISES MOIS DE FEVRIER/2015

Produits Miniers par tonnes

N°	SOCIETES	MONTANTS (USD)	Conc.Cu (10-30%)	Conc.Co (4-15%)	Conc.CuCo	Cu.Noir (80-98%)	Cath.Cu (99%)	All.Blanc (30%Co ≤ 30%Cu≤	All.Rouge (±80% Cu	P-Zinc (89%Zn)	Mat-Cu (±40%)	Hetero Co (Brut)	Min Cu (Brut)	Hydro.Co (±29%)	Cu.Pul (Cu 95%)	Cu.Bott (Cu 97%)	P.Plomb (P 30%)	Carb.Co (±25%)	Carb.Cu (±25%)	Nod.Cu (±99%)	Co.Electro (Co 99%)	Co-sep Mag (Co 55,25%)	Sable. Cobel	Anod.Sol	Scraps (Cu±30%)	Co en Granule (96%)	Con
1	ANVIL MINING CONGO (CMCC)																										
2	BOLFAST	13594,50		450																							
3	BOSS MINING	446480,73			6990,187		2350,395							8,176													
4	CAMIS	1116,70																									
5	CCC/VENTE LOCALE																										
6	CDM	345510,44		5031,45		1023,108	1013,882				95,85			267,6													
7	CEPRODEV	2372,82																									
8	CHEMAF	259636,51					1400							866,25													
9	CIMCO	54015,80					641																				
10	CMSK	69487,29			2624,494																						
11	CNMC HUACHIN MINING MABENDE	137913,71					1263,694																				
12	COMIDE/VENTE LOCALE	61019,25	1942,43																								
13	COMILU	133667,01					1310,11																				
14	CONGO JIN JU CHENG																										
15	CROWN MINING	8325,80																									
16	DRAGON INTERNATIONAL	16168,02				198																					
17	EAGLE GROUP																										
18	FEZA MINING																										
19	FRONTIER																										
20	GCM	148917,23					759,635		520	1968							236										
21	GOLDEN AFRICAN	21797,00					258,5																				
22	GROUPE LA BONNE FIANCE																										
23	GTL							2682,87																			
24	HUACHIN METAL LEACH	131640,04					1204,728																				
25	HUACHIN MINING	137688,31		2000		948																					
26	KAI PENG MINING	55540,02					514,483																				
27	KATANGA METALS	11475,00				125																					
28	KCC	1523674,51					13016,28														208,54						
29	KISANFU MINING/VENTE LOCALE	24514,95											4375,7														
30	LONG FEI/VENTE LOCALE																										

N°	SOCIETES	MONTANTS (USD)	Conc.Cu (10-30%)	Conc.Co (4-15%)	Conc.CuCo	Cu.Noir (80-98%)	Cath.Cu (99%)	All.Blanc (30%Co ≤ 30%Cu≤	All.Rouge (±80% Cu	P-Zinc (89%Zn)	Mat-Cu (±40%)	Hetero Co (Brut)	Min Cu (Brut)	Hydro.Co (±29%)	Cu.Pul (Cu 95%)	Cu.Bott (Cu 97%)	P.Plomb (P 30%)	Carb.Co (±25%)	Carb.Cu (±25%)	Nod.Cu (±99%)	Co.Electro (Co 99%)	Co-sep Mag (Co 55,25%)	Sable. Cobel	Anod.Sol	Scraps (Cu±30%)	Co en Granule (96%)	Conc
31	LUNA MINING/VENTE LOCALE	42899,37	5601,97																								
32	MAGMA																										
33	METALS MINES	71263,98		1700			225																				
34	MIKAS VENTE LOVALE	29611,39	9371,44																								
35	MINES D'AFRIQUE	6885,00																									
36	MJM	20441,35					223																				
37	MKM	221616,23					2172,708																				
38	MMG KINSEVERE	821776,32					8150,988																				
39	MMR	69538,72																									
40	MUMI EXPORT	1802313,57					15874							3339,7													
41	MUMI/VENTE LOCALE																										
42	OM METAL	2899,72					29,92																				
43	OPERA MINING																										
44	PANCOM	45664,11				513,638																					
45	RUASHI MINING	486678,07					2993,683							2080,228													
46	RUBAMIN	153187,33				1955,916																					
47	SEK	251544,47	885				2435,097																				
48	SHITURU MINING	230319,34					2606,058																				
49	SINO KATANGA (STK)	7565,57																									[illegible]
50	SODIMICO																										
51	SOMIKA	138569,62		306			936							362,6													
52	TFM	2943584,32					16878,7							6422,644													
	RUASHI MINING VENTE LOCALE																										
	SEK VENTE LOCALE																										
TOTAL		*10 950 914,12*	*17800,84*	*9487,45*	*9614,68*	*4763,66*	*76257,86*	*2682,87*	*520,00*	*1968,00*	*95,85*	*0,00*	*4375,74*	*13347,20*	*0,00*	*0,00*	*236,00*	*0,00*	*0,00*	*0,00*	*208,54*	*0,00*	*0,00*	*0,00*	*0,00*	*0,00*	[illegible]

Le total général est de: Dix millions neuf cent cinquante mille neuf cent quatorze dollars, douze centimes

N.B:

Légende: Concentré de cuivre: (Conc.cu); concentré de cobalt : (Conc.co), concentré cupro-cobaltifère: (Conc.CuCo); Cathode de cuivre: (Cath.Cu); Matte de cuivre: (Matt.Cu); Hydroxyde de Cobalt: (Hydro.Co); Carbonate de cobalt: (Carb.Co); Poussière de Zinc: (Pouss.Zinc); Poussière de Plomb: (P.Plomb); Nodule du cuivre :(Nod.Cu); Alliage Rouge: (All.Rouge); Alliage Blanc : (All.Blanc); Cuivre Pulverisé: (Cu.Pul); Cuivre Bottom: (Cu.Bott); Hétérogénite de cobalt: (Hetero.Co); Minerais de cuivre (Min.Cu)

Fait à Lubumbashi, le 07 Mars 2015

Emmanuel KIANDA KIBAWA

République Démocratique du Congo

PROVINCE DU KATANGA
DIVISION PROVINCIALE DES MINES
LUBUMBASHI
Le Chef de Division

STATISTIQUES DES NOTES DE DEBIT RELATIVES A LA REDEVANCE MINIERE EMISES MOIS DE MARS/2015

Produits Miniers par tonnes

N°	SOCIETES	MONTANTS (USD)	Conc.Cu (10-30%)	Conc.Co (4-15%)	Conc.CuCo	Cu.Noir (80-98%)	Cath.Cu (99%)	All.Blanc (30%Co ≤ 30%Cu≤	All.Rouge (±80% Cu	P-Zinc (89%Zn)	Mat-Cu (±40%)	Hetero Co (Brut)	Min Cu (Brut)	Hydro.Co (±29%)	Cu.Pul (Cu 95%)	Cu.Bott (Cu 97%)	P.Plomb (P 30%)	Carb.Co (±25%)	Carb.Cu (±25%)	Nod.Cu (±99%)	Co.Electro (Co 99%)	Co-sep Mag (Co 55,25%)	Sable Cobel	Anod.Sol	Scraps (Cu±30%)	Co en Granule (96%)	Con
1	ANVIL MINING CONGO (CMCC)																										
2	BOLFAST	13770,30		450																							
3	BOSS MINING	521030,29		3935,853	4665,517		2920,5																				
4	CAMIS																										
5	CCC/VENTE LOCALE	14754,65										563,053	130,14														
6	CDM	497465,24		8071,65		1927,903	774,561				162			364,8													
7	CEPRODEV																										
8	CHEMAF	299116,84					1728							822,75													
9	CIMCO	37479,90					414																				
10	CMSK	4368,31			162,614																						
11	CNMC HUACHIN MINING MABENDE	104087,32					1107,256																				
12	COMIDE/VENTE LOCALE	39960,60											1340,4														
13	COMILU	105115,45					1187,725																				
14	CONGO JIN JU CHENG	11261,80					125,8985																				
15	CROWN MINING																										
16	DRAGON INTERNATIONAL	20454,40				256																					
17	EAGLE GROUP																										
18	FEZA MINING	73239,39						488																			
19	FRONTIER	691658,90	32222,5																								
20	GCM	91530,14					673,303			1183,5							603,5										
21	GOLDEN AFRICAN	16621,00					188																				
22	GROUPE LA BONNE FIANCE	12616,00																									
23	GTL							3095,79																			
24	HUACHIN METAL LEACH	84266,14					907,054																				
25	HUACHIN MINING	84459,77		2225		186																					
26	KAI PENG MINING	122917,38					1074,508																				
27	KATANGA METALS	11475,00				125																					
28	KCC	1342947,78					11563,52													29,919	260,36						
29	KISANFU MINING/VENTE LOCALE																										
30	LONG FEI/VENTE LOCALE																										

N°	SOCIETES	MONTANTS (USD)	Conc.Cu (10-30%)	Conc.Co (4-15%)	Conc.CuCo	Cu.Noir (80-98%)	Cath.Cu (99%)	All.Blanc (30%Co ≤ 30%Cu≤	All.Rouge (±80% Cu	P-Zinc (89%Zn)	Mat-Cu (±40%)	Hetero Co (Brut)	Min Cu (Brut)	Hydro.Co (±29%)	Cu.Pul (Cu 95%)	Cu.Bott (Cu 97%)	P.Plomb (P 30%)	Carb.Co (±25%)	Carb.Cu (±25%)	Nod.Cu (±99%)	Co.Electro (Co 99%)	Mag (Co 55,25%)	Sable. Cobel	Anod.Sol	Scraps (Cu±30%)	Granule (96%)	Conc.
31	LUNA MINING/VENTE LOCALE	4229,02	611,56																								
32	MAGMA																										
33	METALS MINES	94298,98		2450			225																				
34	MIKAS VENTE LOVALE	31484,66	10408,84																								
35	MINES D'AFRIQUE																										
36	MJM	51326,69		660			349																				
37	MKM	155985,35					1639,158							102,234													
38	MMG KINSEVERE	1005199,46					9525,166																				
39	MMR	56570,08																									
40	MUMI EXPORT	1891148,39					16645,6							4008,74													
41	MUMI/VENTE LOCALE																										
42	OM METAL	3136,50					30																				
43	OPERA MINING	3921,40																									
44	PANCOM	41004,60				474,214																					
45	RUASHI MINING	435811,34					2716,728							1714,94													
46	RUBAMIN	124743,32				1624,578																					
47	SEK	193085,24					1950,002																				
48	SHITURU MINING	268508,05					3059,894																				
49	SINO KATANGA (STK)																										
50	SODIMICO																										
51	SOMIKA	177295,98		210			1006							736,1													
52	TFM	2883661,63					17104,16							8162,053													
	RUASHI MINING VENTE LOCALE																										
	SEK VENTE LOCALE																										
TOTAL		***11 622 007,30***	***43242,90***	***18002,50***	***4828,13***	***4593,70***	***76915,03***	***3583,79***	***0,00***	***1183,50***	***162,00***	***563,05***	***1470,57***	***15911,62***	***0,00***	***0,00***	***603,50***	***0,00***	***0,00***	***29,92***	***260,36***	***0,00***	***0,00***	***0,00***	***0,00***	***0,00***	***5[illegible]***

Le total général est de: Onze millions six cent vingt deux mille sept dollars, trente centimes

N.B:

Légende: Concentré de cuivre: (Conc.cu); concentré de cobalt (Conc.co); concentré cupro-cobaltifère: (Conc.CuCo); Cathode de cuivre: (Cath.Cu); Matte de cuivre: (Matt.Cu); Hydroxyde de Cobalt: (Hydro.Co); Carbonate de cobalt: (Carb.Co); Poussière de Zinc: (Pouss.Zinc); Poussière de Plomb: (P.Plomb); Nodule du cuivre :(Nod.Cu); Alliage Rouge: (All.Rouge); Alliage Blanc : (All.Blanc); Cuivre Pulverisé: (Cu.Pul); Cuivre Bottom: (Cu.Bott); Hétérogénite de cobalt: (Hetero.Co); Minerais de cuivre: (Min.Cu)

Fait à Lubumbashi, le 06 Avril 2015
Emmanuel KIANDA KIBAWA

République Démocratique du Congo

PROVINCE DU KATANGA
DIVISION PROVINCIALE DES MINES
LUBUMBASHI
Le Chef de Division

STATISTIQUES DES NOTES DE DEBIT RELATIVES A LA REDEVANCE MINIERE EMISES MOIS D'AVRIL/2015

N°	SOCIETES	MONTANTS (USD)	Conc.Cu (10-30%)	Conc.Co (4-15%)	Conc.CuCo	Cu.Noir (80-98%)	Cath.Cu (99%)	All.Blanc (30%Co ≤ 30%Cu≤	All.Rouge (±80% Cu	P-Zinc (89%Zn)	Mat-Cu (±40%)	Hetero Co (Brut)	Min Cu (Brut)	Hydro.Co (±29%)	Cu.Pul (Cu 95%)	Cu.Bott (Cu 97%)	P.Plomb (P 30%)	Carb.Co (±25%)	Carb.Cu (±25%)	Nod.Cu (±99%)	Co.Electro (Co 99%)	Co-sep Mag (Co 55,25%)	Sable. Cobel	Anod.Sol	Scraps (Cu±30%)	Co en Granule (96%)	Conc
1	ANVIL MINING CONGO (CMCC)																										
2	BOLFAST	9215,75		325																							
3	BOSS MINING	376655,44		4418,749	3192,264		2067																				
4	CAMIS																										
5	CCC/VENTE LOCALE	2137,13										39,4	67,193														
6	CDM	396758,49		6299,1		1590,989	627,112				162			332,4													
7	CEPRODEV																										
8	CHEMAF	332752,80					2396							470,25													
9	CIMCO	60489,40					657																				
10	CMSK																										
11	CNMC HUACHIN MINING MABENDE	132097,91					1369,972																				
12	COMIDE/VENTE LOCALE	51472,39	1519,77																								
13	COMILU	27756,17					318,268																				
14	CONGO JIN JU CHENG	13107,00					150																				
15	CROWN MINING																										
16	DRAGON INTERNATIONAL	7910,10				99																					
17	EAGLE GROUP																										
18	FEZA MINING	19227,78						128																			
19	FRONTIER	422792,43	18312																								
20	GCM	263191,65			3716,776		927,833		300	1748							584,5				10		20				
21	GOLDEN AFRICAN	27693,00					312																				
22	GROUPE LA BONNE FIANCE																										
23	GTL							2796,1																			
24	HUACHIN METAL LEACH	118955,42					1237,92																				
25	HUACHIN MINING	109142,78		1875		683																					
26	KAI PENG MINING	121166,61					1037,394																				
27	KATANGA METALS	2017,64				24,726																					
28	KCC	1662941,19					13874,14														340,16						
29	KISANFU MINING/VENTE LOCALE	9005,30											9142,8														
30	LONG FEI/VENTE LOCALE																										

N°	SOCIETES	MONTANTS (USD)	Conc.Cu (10-30%)	Conc.Co (4-15%)	Conc.CuCo	Cu.Noir (80-98%)	Cath.Cu (99%)	All.Blanc (30%Co ≤ 30%Cu≤	All.Rouge (±80% Cu	P-Zinc (89%Zn)	Mat-Cu (±40%)	Hetero Co (Brut)	Min Cu (Brut)	Hydro.Co (±29%)	Cu.Pul (Cu 95%)	Cu.Bott (Cu 97%)	P.Plomb (P 30%)	Carb.Co (±25%)	Carb.Cu (±25%)	Nod.Cu (±99%)	Co.Electro (Co 99%)	Mag (Co 55,25%)	Sable. Cobel	Anod.Sol	Scraps (Cu±30%)	Granule (96%)	Conc.
31	LUNA MINING/VENTE LOCALE	12958,62	2436,08																								
32	MAGMA	109425,60				1350																					
33	METALS MINES	91549,24		2300			325																				
34	MIKAS VENTE LOVALE	14947,57	4750,23																								
35	MINES D'AFRIQUE																										
36	MJM	88362,31		2446			216																				
37	MKM	206064,68					1996,641							369,093													
38	MMG KINSEVERE	720976,21					6689,837																				
39	MMR	38463,18																									
40	MUMI EXPORT	1936783,75					16838,66							4683,6													
41	MUMI/VENTE LOCALE																										
42	OM METAL	3136,50					30																				
43	OPERA MINING																										
44	PANCOM	40636,20				470,12																					
45	RUASHI MINING	402095,41					2384,137							1713,7													
46	RUBAMIN	35740,95				452,863																					
47	SEK	210840,82					2064,567																				
48	SHITURU MINING	246520,39					2802,363																				
49	SINO KATANGA (STK)	11374,31																									7[illegible]
50	SODIMICO																										
51	SOMIKA	93188,30					886																				
52	TFM	2662852,44					17341,36							6312,601													
	RUASHI MINING VENTE LOCALE																										
	SEK VENTE LOCALE																										
TOTAL		11 092 402,88	27018,08	17663,85	6909,04	4670,70	76549,20	2924,10	300,00	1748,00	162,00	39,40	9209,94	13881,64	0,00	0,00	584,50	0,00	0,00	0,00	350,16	0,00	20,00	0,00	0,00	0,00	33[illegible]

Le total général est de: Onze millions six cent trente un mille six cent soixante cinq dollars, quarante cinq centimes

N.B:

Légende. Concentré de cuivre: (Conc.cu); concentré de cobalt : (Conc.co); concentré cupro-cobaltifère: (Conc.CuCo); Cathode de cuivre: (Cath.Cu), Matte de cuivre: (Matt.Cu), Hydroxyde de Cobalt: (Hydro.Co); Carbonate de cobalt: (Carb.Co); Poussière de Zinc. (Pouss.Zinc); Poussière de Plomb: (P.Plomb); Nodule du cuivre :(Nod.Cu); Alliage Rouge: (All.Rouge); Alliage Blanc : (All.Blanc); Cuivre Pulverisé: (Cu.Pul); Cuivre Bottom. (Cu.Bott); Hétérogénite de cobalt; (Hetero.Co); Minerais de cuivre' (Min Cu)

Fait à Lubumbashi, le 11 Mai 2015
Emmanuel KIANDA KIBAWA

République Démocratique du Congo

PROVINCE DU KATANGA
DIVISION PROVINCIALE DES MINES
LUBUMBASHI
Le Chef de Division

STATISTIQUES DES NOTES DE DEBIT RELATIVES A LA REDEVANCE MINIERE EMISES
MOIS DE MAI/2015

N°	SOCIETES	MONTANTS (USD)	Conc.Cu (10-30%)	Conc.Co (4-15%)	Conc.CuCo	Cu.Noir (80-98%)	Cath.Cu (99%)	All.Blanc (30%Co ≤ 30%Cu≤	All.Rouge (±80% Cu	P-Zinc (89%Zn)	Mat-Cu (±40%)	Hetero Co (Brut)	Min Cu (Brut)	Hydro.Co (±29%)	Cu.Pul (Cu 95%)	Cu.Bott (Cu 97%)	P.Plomb (P 30%)	Carb.Co (±25%)	Carb.Cu (±25%)	Nod.Cu (±99%)	Co.Electro (Co 99%)	Co-sep Mag (Co 55,25%)	Sable Cobel	Anod.Sol.	Scraps (Cu±30%)	Co en Granule (96%)	Conc[illegible]
1	ANVIL MINING CONGO (CMCC)																										
2	BOLFAST	25880,80		875																							
3	BOSS MINING	370626,18		6635,297	70,931		2332,5																				
4	CAMIS																										
5	CCC/VENTE LOCALE																										
6	CDM																										
7	CEPRODEV																										
8	CHEMAF	338387,71					1816							918													
9	CIMCO																										
10	CMSK																										
11	CNMC HUACHIN MINING MABENDE	89256,58					889,187																				
12	COMIDE/VENTE LOCALE	51277,99	1596,38																								
13	COMILU	89529,90					1026,598																				
14	CONGO JIN JU CHENG																										
15	CROWN MINING	22955,10																									
16	DRAGON INTERNATIONAL																										
17	EAGLE GROUP																										
18	FEZA MINING																										
19	FRONTIER	420341,91	18332																								
20	GCM	203910,35			1867,325		799,114			1528,5							537	130			40	10					
21	GOLDEN AFRICAN	19182,25					196,5																				
22	GROUPE LA BONNE FIANCE																										
23	GTL							1437,18																			
24	HUACHIN METAL LEACH	88448,58					835,998																				
25	HUACHIN MINING					160																					
26	KAI PENG MINING																										
27	KATANGA METALS																										
28	KCC	1507787,71					12136,96													30,031	313,62						
29	KISANFU MINING/VENTE LOCALE																										
30	LONG FEI/VENTE LOCALE																										

N°	SOCIETES	MONTANTS (USD)	Conc.Cu (10-30%)	Conc.Co (4-15%)	Conc.CuCo	Cu.Noir (80-98%)	Cath.Cu (99%)	All.Blanc (30%Co ≤ 30%Cu≤	All.Rouge (±80% Cu	P-Zinc (89%Zn)	Mat-Cu (±40%)	Hetero Co (Brut)	Min Cu (Brut)	Hydro.Co (±29%)	Cu.Pul (Cu 95%)	Cu.Bott (Cu 97%)	P.Plomb (P 30%)	Carb.Co (±25%)	Carb.Cu (±25%)	Nod.Cu (±99%)	Co.Electro (Co 99%)	Mag (Co 55,25%)	Sable. Cobel	Anod.Sol	Scraps (Cu±30%)	Granule (96%)	Conc
31	LUNA MINING/VENTE LOCALE																										
32	MAGMA																										
33	METALS MINES	153866,96		4725			225																				
34	MIKAS VENTE LOVALE	13585,87	4559,79																								
35	MINES D'AFRIQUE																										
36	MJM																										
37	MKM	98267,21					890,081							238,567													
38	MMG KINSEVERE	727730,02					6409,875																				
39	MMR	26016,46																									
40	MUMI EXPORT	2113291,91					17766,23							4852,83													
41	MUMI/VENTE LOCALE																										
42	OM METAL																										
43	OPERA MINING																										
44	PANCOM																										
45	RUASHI MINING	388476,58					2762,69							806,11													
46	RUBAMIN	37023,14				459,764																					
47	SEK	210346,89					1967,2																				
48	SHITURU MINING	220928,79					2443,169																				
49	SINO KATANGA (STK)																										
50	SODIMICO				32																						
51	SOMIKA	138528,12					1108							169,2													
52	TFM	3096718,41					17220,62							7929,911													
	RUASHI MINING VENTE LOCALE																										
	SEK VENTE LOCALE																										
TOTAL		10 452 365,43	24488,17	12235,30	1970,26	619,76	70825,72	1437,18	0,00	1528,50	0,00	0,00	0,00	14914,62	0,00	0,00	537,00	130,00	0,00	30,03	353,62	10,00	0,00	0,00	0,00	0,00	[illegible]

Le total général est de: Dix millions quatre cent cinquante deux mille trois cent soixante cinq dollars, quarante trois centimes

N.B:

Légende: Concentré de cuivre: (Conc.cu); concentré de cobalt : (Conc.co); concentré cupro-cobaltifère: (Conc.CuCo); Cathode de cuivre: (Cath.Cu); Matte de cuivre: (Matt.Cu); Hydroxyde de Cobalt: (Hydro.Co), Carbonate de cobalt: (Carb.Co); Poussière de Zinc: (Pouss.Zinc); Poussière de Plomb: (P.Plomb); Nodule du cuivre :(Nod.Cu); Alliage Rouge: (All.Rouge); Alliage Blanc (All.Blanc); Cuivre Pulverisé: (Cu.Pul); Cuivre Bottom: (Cu.Bott); Hétérogénite de cobalt: (Hetero.Co); Minerais de cuivre: (Min.Cu)

Fait à Lubumbashi, le 06 Juin 2015

Emmanuel KIANDA KIBAWA

République Démocratique du Congo

PROVINCE DU KATANGA
DIVISION PROVINCIALE DES MINES
LUBUMBASHI
Le Chef de Division

STATISTIQUES DES NOTES DE DEBIT RELATIVES A LA REDEVANCE MINIERE EMISES MOIS DE JUIN/2015

N°	SOCIETES	MONTANTS (USD)	Conc.Cu (10-30%)	Conc.Co (4-15%)	Conc.CuCo	Cu.Noir (80-98%)	Cath.Cu (99%)	All.Blanc (30%Co ≤ 30%Cu≤	All.Rouge (±80% Cu	P-Zinc (89%Zn)	Mat-Cu (±40%)	Hetero Co (Brut)	Min Cu (Brut)	Hydro.Co (±29%)	Cu.Pul (Cu 95%)	Cu.Bott (Cu 97%)	P.Plomb (P 30%)	Carb.Co (±25%)	Carb.Cu (±25%)	Nod.Cu (±99%)	Co.Electro (Co 99%)	Co-sep Mag (Co 55,25%)	Sable. Cobel	Anod.Sol	Scraps (Cu±30%)	Co en Granule (96%)	Co[illegible]
1	ANVIL MINING CONGO (CMCC)		5000																								
2	BOLFAST	35966,88		1050			60																				
3	BOSS MINING	395744,25		4726,934	1760,026		2116,5											185,99		33							
4	CAMIS																										
5	CCC/VENTE LOCALE																										
6	CDM	543113,07		6677,1		2519,032	1020,56				162			567													
7	CEPRODEV																										
8	CHEMAF	334190,49					2151							714													
9	CIMCO																										
10	CMSK																										
11	CNMC HUACHIN MINING MABENDE	133543,35					1369,49																				
12	COMIDE/VENTE LOCALE	71072,21	2164,32																								
13	COMILU	104625,58					1080,574																				
14	CONGO JIN JU CHENG																										
15	CROWN MINING																										
16	DRAGON INTERNATIONAL																										
17	EAGLE GROUP																										
18	FEZA MINING	73170,74		1056				282																			
19	FRONTIER	448288,68	18398																								
20	GCM	293783,72			1965,707		1350,448		680	2048,39							30,5										
21	GOLDEN AFRICAN	32186,40					345																				
22	GROUPE LA BONNE FIANCE																										
23	GTL							2889,66																			
24	HUACHIN METAL LEACH	100927,10					1068,554																				
25	HUACHIN MINING	92721,14		1225		636																					
26	KAI PENG MINING																										
27	KATANGA METALS																										
28	KCC	1685657,30					13375,06														286,72						
29	KISANFU MINING/VENTE LOCALE	53460,08											11679,72														
30	LONG FEI/VENTE LOCALE																										
31	LUNA MINING/VENTE LOCALE																										

N°	SOCIETES	MONTANTS (USD)	Conc.Cu (10-30%)	Conc.Co (4-15%)	Conc.CuCo	Cu.Noir (80-98%)	Cath.Cu (99%)	All.Blanc (30%Co ≤ 30%Cu≤	All.Rouge (±80% Cu	P-Zinc (89%Zn)	Mat-Cu (±40%)	Hetero Co (Brut)	Min Cu (Brut)	Hydro.Co (±29%)	Cu.Pul (Cu 95%)	Cu.Bott (Cu 97%)	P.Plomb (P 30%)	Carb.Co (±25%)	Carb.Cu (±25%)	Nod.Cu (±99%)	[illegible] (Co 99%)	[illegible] 55,25%)	Cobel	Anod.Sol	(Cu±30%)	(96%)	Con[illegible]
32	[illegible]AGMA																										
33	METALS MINES	172078,24		5200			225																				
34	MIKAS VENTE LOVALE	23474,68	7185,93																								
35	MINES D'AFRIQUE																										
36	MJM																										
37	MKM	157652,87					1525,326							100,283													
38	MMG KINSEVERE	607517,61					5812,035																				
39	MMR	30803,32																									
40	MUMI EXPORT	2266132,45					17988,56							4620,95													
41	MUMI/VENTE LOCALE																										
42	OM METAL																										
43	OPERA MINING																										
44	PANCOM																										
45	RUASHI MINING	414075,10					2628,19							1678,354													
46	RUBAMIN	37700,68				451,724																					
47	SEK	267330,24					2669,565																				
48	SHITURU MINING	252690,94					2661,003																				
49	SINO KATANGA (STK)	7446,53																									
50	SODIMICO				480																						
51	SOMIKA	187758,75		210			1278							386,8													
52	TFM	3013915,74					16765,37							7633,194													
53	SEK/VENTE LOCALE	678961,71	89675																								
TOTAL		*12 515 989,87*	*122423,25*	*20145,03*	*4205,73*	*3606,76*	*75490,24*	*3171,66*	*680,00*	*2048,39*	*162,00*	*0,00*	*11679,72*	*15700,58*	*0,00*	*0,00*	*30,50*	*185,99*	*0,00*	*33,00*	*286,72*	*0,00*	*0,00*	*0,00*	*0,00*	*0,00*	

Le total général est de:Douze millions cinq cent quinze mille neuf cent quatre vingt neuf, quatre vingt sept

N.B:

Légende: Concentré de cuivre: (Conc.cu); concentré de cobalt (Conc.co); concentré cupro-cobaltifère: (Conc.CuCo); Cathode de cuivre: (Cath.Cu); Matte de cuivre: (Matt.Cu); Hydroxyde de Cobalt: (Hydro.Co); Carbonate de cobalt: (Carb.Co), Poussière de Zinc: (Pouss.Zinc); Poussière de Plomb: (P.Plomb); Nodule du cuivre :(Nod.Cu); Alliage Rouge (All.Rouge); Alliage Blanc : (All.Blanc); Cuivre Pulverisé: (Cu.Pul); Cuivre Bottom: (Cu.Bott), Hétérogénite de cobalt: (Hetero.Co); Minerais de cuivre: (Min.Cu)

Fait à Lubumbashi, le 08 Juillet 2015
Emmanuel KIANDA KIBAWA

PROVINCE DU KATANGA
DIVISION PROVINCIALE DES MINES
LUBUMBASHI
Le Chef de Division

STATISTIQUES DES NOTES DE DEBIT RELATIVES A LA REDEVANCE MINIERE EMISES MOIS DE JUILLET/2015

N°	SOCIETES	MONTANTS (USD)	Conc.Cu (10-30%)	Conc.Co (4-15%)	Conc.CuCo	Cu.Noir (80-98%)	Cath.Cu (99%)	All.Blanc (30%Co ≤ 30%Cu≤	All.Rouge (±80% Cu	P-Zinc (89%Zn)	Mat-Cu (±40%)	Hetero Co (Brut)	Min Cu (Brut)	Hydro.Co (±29%)	Cu.Pul (Cu 95%)	Cu.Bott (Cu 97%)	P.Plomb (P 30%)	Carb.Co (±25%)	Carb.Cu (±25%)	Nod.Cu (±99%)	Co.Electro (Co 99%)	Co-sep Mag (Co 55,25%)	Sable. Cobel	Anod.Sol	Scraps (Cu±30%)	Co en Granule (96%)	Co
1	ANVIL MINING CONGO (CMCC)		11100																								
2	BOLFAST	46339,09		1200			120																				
3	BOSS MINING	522082,05		663,445	5130,554		3264											45,131									
4	CAMIS																										
5	CCC/VENTE LOCALE																										
6	CDM	611650,82		5304,15		3605,173	1201,06				129,6			866,4													
7	CEPRODEV																										
8	CHEMAF	265270,04					1403							852													
9	CIMCO																										
10	CMSK																										
11	CNMC HUACHIN MINING MABENDE	94347,70					1041,087																				
12	COMIDE/VENTE LOCALE	57306,88	1809,41																								
13	COMILU	45904,30					450,042																				
14	CONGO JIN JU CHENG																										
15	CROWN MINING																										
16	DRAGON INTERNATIONAL																										
17	EAGLE GROUP																										
18	FEZA MINING	94391,17		2304				128																			
19	FRONTIER	648501,54	28586,9																								
20	GCM	256573,67			879,645		1560,687		264	2177,94							30,5				50		10				
21	GOLDEN AFRICAN	27243,80					308																				
22	GROUPE LA BONNE FIANCE																										
23	GTL							1217,64																			
24	HUACHIN METAL LEACH	118360,00					1298,327																				
25	HUACHIN MINING	85429,68		1600		450																					
26	KAI PENG MINING																										
27	KATANGA METALS																										
28	KCC	1940002,70					16155,08													34,073	338						
29	KISANFU MINING/VENTE LOCALE																										
30	LONG FEI/VENTE LOCALE																										
31	LUNA MINING/VENTE LOCALE																										

N°	SOCIETES	MONTANTS (USD)	30%)	15%)	Conc.CuCo	98%)	(99%)	30%Cus	(±80% Cu	(89%Zn)	(±40%)	(Brut)	(Brut)	(±29%)	(Cu 95%)	(Cu 97%)	30%)	(±25%)	(±25%)	(±99%)	(Co 99%)	55,25%)	Cobel	Anod.Sol	(Cu±30%)	(96%)
32	MAGMA																									
33	METALS MINES	141958,48		4050			275																			
34	MIKAS VENTE LOVALE	1629,29	405,66																							
35	MINES D'AFRIQUE																									
36	MJM																									
37	MKM	191440,08					1855,468																			
38	MMG KINSEVERE	656750,53					6741,574																			
39	MMR	27362,02																								
40	MUMI EXPORT	2341452,46					19397,33							5231,04												
41	MUMI/VENTE LOCALE	83085,41			12226,474																					
42	OM METAL																									
43	OPERA MINING																									
44	PANCOM																									
45	RUASHI MINING	461165,58					3309,354							1545,402												
46	RUBAMIN	51130,72				643,013																				
47	SEK	234827,64					2474,328																			
48	SHITURU MINING	248177,45					2647,585																			
49	SINO KATANGA (STK)	6510,30																								
50	SODIMICO				640																					
51	SOMIKA	206647,69		476,4			1304							609,3												
52	TFM	2941648,30					18242,99							7107,637												
53	RUASHI MINING VENTE LOCALE	168,00					2,5																			
54	SEK/VENTE LOCALE																									
TOTAL		*12 407 357,39*	*41901,97*	*15598,00*	*18876,67*	*4698,19*	*83051,41*	*1345,64*	*264,00*	*2177,94*	*129,60*	*0,00*	*0,00*	*16211,78*	*0,00*	*0,00*	*30,50*	*45,13*	*0,00*	*34,07*	*388,00*	*0,00*	*10,00*	*0,00*	*0,00*	*0,00*

Le total général est de:Douze millions quatre cent sept mille trois cent cinquante sept, trente neuf

N.B: 514,32 Tonnes de Plomb Affiné à 99% exporter par KCC

Légende: Concentré de cuivre: (Conc.cu); concentré de cobalt : (Conc.co); concentré cupro-cobaltifère: (Conc.CuCo); Cathode de cuivre: (Cath.Cu); Matte de cuivre: (Matt.Cu); Hydroxyde de Cobalt: (Hydro.Co); Carbonate de cobalt: (Carb.Co); Poussière de Zinc: (Pouss.Zinc); Poussière de Plomb: (P.Plomb), Nodule du cuivre :(Nod.Cu); Alliage Rouge: (All.Rouge); Alliage Blanc : (All.Blanc); Cuivre Pulverisé: (Cu.Pul), Cuivre Bottom: (Cu.Bott); Hétérogénite de cobalt: (Hetero.Co); Minerais de cuivre: (Min.Cu)

Fait à Lubumbashi, le 05 Août 2015
Emmanuel KIANDA KIBAWA

PROVINCE DU KATANGA
DIVISION PROVINCIALE DES MINES
LUBUMBASHI
Le Chef de Division

STATISTIQUES DES NOTES DE DEBIT RELATIVES A LA REDEVANCE MINIERE EMISES MOIS D'AOUT/2015

N°	SOCIETES	MONTANTS (USD)	Conc.Cu (10-30%)	Conc.Co (4-15%)	Conc.CuCo	Cu.Noir (80-98%)	Cath.Cu (99%)	All.Blanc (30%Co ≤ 30%Cu≤	All.Rouge (±80% Cu	P-Zinc (89%Zn)	Mat-Cu (±40%)	Hetero Co (Brut)	Min Cu (Brut)	Hydro.Co (±29%)	Cu.Pul (Cu 95%)	Cu.Bott (Cu 97%)	P.Plomb (P 30%)	Carb.Co (±25%)	Carb.Cu (±25%)	Nod.Cu (±99%)	Co.Electro (Co 99%)	Co-sep Mag (Co 55,25%)	Sable. Cobel	Anod.Sol	Scraps (Cu±30%)	Co en Granule (96%)	C
1	ANVIL MINING CONGO (CMCC)																										
2	BOLFAST	61268,09		1125			330																				
3	BOSS MINING	295837,81		4905,286	1405,713		1760,25											46,008									
4	CAMIS																										
5	CCC/VENTE LOCALE																										
6	CDM	398093,69		3514,05		1668,483	630,857				64,8			1437,8													
7	CEPRODEV																										
8	CHEMAF	391032,56					2765							980,25													
9	CIMCO	122105,90					1628																				
10	CMSK																										
11	CNMC HUACHIN MINING MABENDE	144229,75					1595,462																				
12	COMIDE/VENTE LOCALE	22708,37	780,36																								
13	COMILU	76540,68					847,818																				
14	CONGO JIN JU CHENG																										
15	CROWN MINING																										
16	DRAGON INTERNATIONAL																										
17	EAGLE GROUP																										
18	FEZA MINING	65959,42		1466				121																			
19	FRONTIER	524238,90	23077,5																								
20	GCM	159110,08					1100,85		178	2306,97											30						
21	GOLDEN AFRICAN	21157,64					252,5																				
22	GROUPE LA BONNE FIANCE																										
23	GTL							3935,42																			
24	HUACHIN METAL LEACH	89316,83					988,018																				
25	HUACHIN MINING	46103,00		400		450																					
26	KAI PENG MINING																										
27	KATANGA METALS																										
28	KCC	1610250,71					13863,75													30,09	430,6						
29	KISANFU MINING/VENTE LOCALE	78608,36											18282,67														
30	LONG FEI/VENTE LOCALE																										
31	LUNA MINING/VENTE LOCALE																										

N°	SOCIETES	MONTANTS (USD)	Conc.Cu (10-30%)	Conc.Co (4-15%)	Conc.CuCo	Cu.Noir (80-98%)	Cath.Cu (99%)	(50%Co ≤ 30%Cu≤	All.Rouge (±80% Cu	P.Zinc (89%Zn)	Mat.Cu (±40%)	Hetero Co (Brut)	Min Cu (Brut)	Hydro.Co (±29%)	Cu.Pul (Cu 95%)	Cu.Bott (Cu 97%)	P.Plomb (P 30%)	Carb.Co (±25%)	Carb.Cu (±25%)	Nod.Cu (±99%)	Co.Electro (Co 99%)	Mag (Co 55,25%)	Sable. Cobel	Anod.Sol	Scraps (Cu±30%)	Granule (96%)	[illegible]
32	MAGMA																										
33	METALS MINES	121728,50		3725			150																				
34	MIKAS VENTE LOVALE	4118,82	1302,87																								
35	MINES D'AFRIQUE																										
36	MJM																										
37	MKM	117083,18					1050,497							238,17													
38	MMG KINSEVERE	509942,21					5723,937																				
39	MMR	20531,24																									
40	MUMI EXPORT	2102034,03					17800,33							5817,35													
41	MUMI/VENTE LOCALE																										
42	OM METAL																										
43	OPERA MINING																										
44	PANCOM																										
45	RUASHI MINING	385047,85					2773,609							1779,952													
46	RUBAMIN	48450,77				660,58																					
47	SEK	210142,96					2403,249																				
48	SHITURU MINING	199627,07					2394,929																				
49	SINO KATANGA (STK)	2980,90																									
50	SODIMICO				320																						
51	SOMIKA	164298,46					1412							354,3													
52	TFM	2086864,86					13552,97							4545,318													
53	RUASHI MINING VENTE LOCALE																										
54	SEK/VENTE LOCALE																										
TOTAL		*10 079 412,63*	*25160,73*	*15135,34*	*1725,71*	*2779,06*	*73024,03*	*4056,42*	*178,00*	*2306,97*	*64,80*	*0,00*	*18282,67*	*15153,14*	*0,00*	*0,00*	*0,00*	*46,01*	*0,00*	*30,09*	*460,60*	*0,00*	*0,00*	*0,00*	*0,00*	*0,00*	

Le total général est de: Dix millions septante neuf mille quatre cent douze dollars, soixante trois

N.B:

Légende: Concentré de cuivre: (Conc.cu); concentré de cobalt : (Conc.co); concentré cupro-cobaltifère: (Conc.CuCo); Cathode de cuivre: (Cath.Cu); Matte de cuivre: (Matt.Cu); Hydroxyde de Cobalt: (Hydro.Co); Carbonate de cobalt: (Carb.Co); Poussière de Zinc: (Pouss.Zinc); Poussière de Plomb: (P Plomb); Nodule du cuivre :(Nod.Cu); Alliage Rouge: (All.Rouge); Alliage Blanc : (All.Blanc); Cuivre Pulverisé: (Cu.Pul); Cuivre Bottom: (Cu.Bott); Hétérogénite de cobalt: (Hetero.Co); Minerais de cuivre: (Min Cu)

Fait à Lubumbashi, le 05 Septembre 2015
Emmanuel KIANDA KIBAWA

PROVINCE DU KATANGA
DIVISION PROVINCIALE DES MINES
LUBUMBASHI
Le Chef de Division

STATISTIQUES DES NOTES DE DEBIT RELATIVES A LA REDEVANCE MINIERE EMISES MOIS DE SEPTEMBRE/2015

N°	SOCIETES	MONTANTS (USD)	Conc.Cu (10-30%)	Conc.Co (4-15%)	Conc.CuCo	Cu.Noir (80-98%)	Cath.Cu (99%)	All.Blanc (30%Co ≤ 30%Cu≤	All.Rouge (±80% Cu	P-Zinc (89%Zn)		Hetero Co (Brut)	Min Cu (Brut)	Hydro.Co (±29%)	Cu.Pul (Cu 95%)	Cu.Bott (Cu 97%)	P.Plomb (P 30%)	Carb.Co (±25%)	Carb.Cu (±25%)	Nod.Cu (±99%)	Co.Electro (Co 99%)	Co-sep Mag (Co 55,25%)	Sable. Cobel	Anod.Sol	Scraps (Cu±30%)	Co en Granul[illegible] (96%)
1	ANVIL MINING CONGO (CMCC)		11100																							
2	BOLFAST	51075,88		550			420																			
3	BOSS MINING	366974,83		2167,225	4003,136		2224,5											92,484		33,75						
4	CAMIS																									
5	CCC/VENTE LOCALE																									
6	CDM	455566,27		7552,35		1177,014	863,419				32,4			1356,2												
7	CEPRODEV																									
8	CHEMAF	317557,47					1864							1105,5												
9	CIMCO	149006,70					1940																			
10	CMSK																									
11	CNMC HUACHIN MINING MABENDE	138244,40					1529,252																			
12	COMIDE/VENTE LOCALE	33845,51	1247,12																							
13	COMILU	59306,35					683,264																			
14	CONGO JIN JU CHENG																									
15	CROWN MINING	10661,04																								
16	DRAGON INTERNATIONAL																									
17	EAGLE GROUP																									
18	FEZA MINING	83392,68		2044				122																		
19	FRONTIER	787238,56	41693																							
20	GCM	290597,54				94,976	2271,741		160	1578,54					325,09						30					
21	GOLDEN AFRICAN	37775,68					449,5																			
22	GROUPE LA BONNE FIANCE																									
23	GTL																									
24	HUACHIN METAL LEACH	80754,04					892,412																			
25	HUACHIN MINING	22426,90		725		30																				
26	KAI PENG MINING	144185,37					1431,918																			
27	KATANGA METALS																									
28	KCC	1170059,21					10836,219													253,502	271,9					
29	KISANFU MINING/VENTE LOCALE	53120,86											15202,96													
30	LONG FEI/VENTE LOCALE																									

N°	SOCIETES	MONTANTS (USD)	30%)	15%)	Conc.CuCo	98%)	(99%)	30%Cu≤	(±80% Cu	(89%Zn)		(Brut)	(Brut)	(±29%)	(Cu 95%)	(Cu 97%)	30%)	(±25%)	(±25%)	(±99%)	Co.Electro (Co 99%)	Mag (Co 55,25%)	Sable. Cobel	Anod.Sol	Scraps (Cu±30%)	Granul (96%)
31	LUNA MINING/VENTE LOCALE																									
32	MAGMA																									
33	METALS MINES	26486,00		500			150																			
34	MIKAS VENTE LOVALE	7810,86	3245,51																							
35	MINES D'AFRIQUE																									
36	MJM																									
37	MKM	68762,89					627,984							171,622												
38	MMG KINSEVERE	522784,13					5775,739																			
39	MMR	22988,42																								
40	MUMI EXPORT	1840800,22					16630,179							6307,37												
41	MUMI/VENTE LOCALE																									
42	OM METAL																									
43	OPERA MINING																									
44	PANCOM																									
45	RUASHI MINING	436061,84					3081,764							2018,513												
46	RUBAMIN	30181,40				437,183																				
47	SEK	211661,55					2396,206																			
48	SHITURU MINING	149113,48					1841,714																			
49	SINO KATANGA (STK)	2893,02																								
50	SODIMICO				64																					
51	SOMIKA	152015,84					968							774,6												
52	TFM	3157565,73					17861,353							8880,773												
53	RUASHI MINING VENTE LOCALE																									
54	SEK/VENTE LOCALE																									
TOTAL		*10 880 914,66*	*57285,63*	*13538,58*	*4067,14*	*1739,17*	*74739,16*	*122,00*	*160,00*	*1578,54*	*32,40*	*0,00*	*15202,96*	*20614,58*	*325,09*	*0,00*	*0,00*	*92,48*	*0,00*	*287,25*	*301,90*	*0,00*	*0,00*	*0,00*	*0,00*	*0,0*

Le total général est de: Dix millions huit cent quatre vingt mille neuf cent quatorze, soixante six

N.B: 33,75 TONNES DE CUIVRE AUTREMENT PRESENTER EXPORTER PAR BOSS MINING
90 TONNES DE SULFURE DE DENICKELAGE EXPORTER PAR LA GCM

Légende: Concentré de cuivre: (Conc.cu); concentré de cobalt : (Conc.co); concentré cupro-cobaltifère: (Conc.CuCo); Cathode de cuivre: (Cath.Cu); Matte de cuivre: (Matt.Cu); Hydroxyde de Cobalt: (Hydro.Co); Carbonate de cobalt: (Carb.Co); Poussière de Zinc: (Pouss.Zinc); Poussière de Plomb: (P.Plomb); Nodule du cuivre :(Nod.Cu); Alliage Rouge: (All.Rouge); Alliage Blanc : (All.Blanc); Cuivre Pulverisé: (Cu.Pul); Cuivre Bottom: (Cu.Bott); Hétérogénite de cobalt: (Hetero.Co); Minerais de cuivre: (Min.Cu)

Fait à Lubumbashi, le 05 Octobre 2015
Emmanuel KIANDA KIBAWA

PROVINCE DU KATANGA
DIVISION PROVINCIALE DES MINES
LUBUMBASHI
Le Chef de Division

STATISTIQUES DES NOTES DE DEBIT RELATIVES A LA REDEVANCE MINIERE EMISES MOIS DE SEPTEMBRE/2015

N°	SOCIETES	MONTANTS (USD)	Conc.Cu (10-30%)	Conc.Co (4-15%)	Conc.CuCo	Cu.Noir (80-98%)	Cath.Cu (99%)	All.Blanc (30%Co ≤ 30%Cu≤	All.Rouge (±80% Cu	P-Zinc (89%Zn)		Hetero Co (Brut)	Min Cu (Brut)	Hydro.Co (±29%)	Cu.Pul (Cu 95%)	Cu.Bott (Cu 97%)	P.Plomb (P 30%)	Carb.Co (±25%)	Carb.Cu (±25%)	Nod.Cu (±99%)	Co.Electro (Co 99%)	Co-sep Mag (Co 55,25%)	Sable. Cobel	Anod.Sol	Scraps (Cu±30%)	Co e Granu (96%
1	ANVIL MINING CONGO (CMCC)		11100																							
2	BOLFAST	51075,88		550			420																			
3	BOSS MINING	366974,83		2167,225	4003,136		2224,5											92,484		33,75						
4	CAMIS																									
5	CCC/VENTE LOCALE																									
6	CDM	455566,27		7552,35		1177,014	863,419				32,4			1356,2												
7	CEPRODEV																									
8	CHEMAF	317557,47					1864							1105,5												
9	CIMCO	149006,70					1940																			
10	CMSK																									
11	CNMC HUACHIN MINING MABENDE	138244,40					1529,252																			
12	COMIDE/VENTE LOCALE	33845,51	1247,12																							
13	COMILU	59306,35					683,264																			
14	CONGO JIN JU CHENG																									
15	CROWN MINING	10661,04																								
16	DRAGON INTERNATIONAL																									
17	EAGLE GROUP																									
18	FEZA MINING	83392,68		2044				122																		
19	FRONTIER	787238,56	41693																							
20	GCM	290597,54				94,976	2271,741		160	1578,54					325,09						30					
21	GOLDEN AFRICAN	37775,68					449,5																			
22	GROUPE LA BONNE FIANCE																									
23	GTL																									
24	HUACHIN METAL LEACH	80754,04					892,412																			
25	HUACHIN MINING	22426,90		725		30																				
26	KAI PENG MINING	144185,37					1431,918																			
27	KATANGA METALS																									
28	KCC	1170059,21					10836,219													253,502	271,9					
29	KISANFU MINING/VENTE LOCALE	53120,86											15202,96													
30	LONG FEI/VENTE LOCALE																									

						[illegible]	[illegible]	50%Cu	(±80% Cu)	(89%Zn)		(Brut)	(Brut)	(±29%)	(Cu 95%)	(Cu 97%)	30%)	(±25%)	(±25%)	(±99%)	(Co 99%)	55,25%)	Cobel	Anod.Sol	(Cu±30%)	(96%
31	LUNA MINING/VENTE LOCALE																									
32	MAGMA																									
33	METALS MINES	26486,00		500			150																			
34	MIKAS VENTE LOVALE	7810,86	3245,51																							
35	MINES D'AFRIQUE																									
36	MJM																									
37	MKM	68762,89					627,984							171,622												
38	MMG KINSEVERE	522784,13					5775,739																			
39	MMR	22988,42																								
40	MUMI EXPORT	1840800,22					16630,179							6307,37												
41	MUMI/VENTE LOCALE																									
42	OM METAL																									
43	OPERA MINING																									
44	PANCOM																									
45	RUASHI MINING	436061,84					3081,764							2018,513												
46	RUBAMIN	30181,40				437,183																				
47	SEK	211661,55					2396,206																			
48	SHITURU MINING	149113,48					1841,714																			
49	SINO KATANGA (STK)	2893,02																								
50	SODIMICO				64																					
51	SOMIKA	152015,84					968							774,6												
52	TFM	3157565,73					17861,353							8880,773												
53	RUASHI MINING VENTE LOCALE																									
54	SEK/VENTE LOCALE																									
TOTAL		*10 880 914,66*	*57285,63*	*13538,58*	*4067,14*	*1739,17*	*74739,16*	*122,00*	*160,00*	*1578,54*	*32,40*	*0,00*	*15202,96*	*20614,58*	*325,09*	*0,00*	*0,00*	*92,48*	*0,00*	*287,25*	*301,90*	*0,00*	*0,00*	*0,00*	*0,00*	*0,0*

Le total général est de: Dix millions huit cent quatre vingt mille neuf cent quatorze, soixante six

N.B: 33,75 TONNES DE CUIVRE AUTREMENT PRESENTER EXPORTER PAR BOSS MINING
90 TONNES DE SULFURE DE DENICKELAGE EXPORTER PAR LA GCM

Légende: Concentré de cuivre: (Conc.cu); concentré de cobalt : (Conc.co); concentré cupro-cobaltifère: (Conc.CuCo); Cathode de cuivre (Cath.Cu); Matte de cuivre: (Matt.Cu); Hydroxyde de Cobalt: (Hydro.Co); Carbonate de cobalt: (Carb.Co); Poussière de Zinc: (Pouss.Zinc); Poussière de Plomb: (P.Plomb); Nodule du cuivre :(Nod.Cu); Alliage Rouge: (All.Rouge); Alliage Blanc : (All.Blanc); Cuivre Pulverisé: (Cu.Pul), Cuivre Bottom: (Cu.Bott); Hétérogénite de cobalt: (Hetero.Co); Minerais de cuivre: (Min.Cu)

Fait à Lubumbashi, le 05 Octobre 2015
Emmanuel KIANDA KIBAWA

PROVINCE DU KATANGA
DIVISION PROVINCIALE DES MINES
LUBUMBASHI
Le Chef de Division

STATISTIQUES DES NOTES DE DEBIT RELATIVES A LA REDEVANCE MINIERE EMISES MOIS D'OCTOBRE/2015

N°	SOCIETES	MONTANTS (USD)	Conc.Cu (10-30%)	Conc.Co (4-15%)	Conc.CuCo	Cu.Noir (80-98%)	Cath.Cu (99%)	All.Blanc (30%Co ≤ 30%Cu≤	All.Rouge (±80% Cu	P-Zinc (89%Zn)		Hetero Co (Brut)	Min Cu (Brut)	Hydro.Co (±29%)	Cu.Pul (Cu 95%)	Cu.Bott (Cu 97%)	P.Plomb (P 30%)	Carb.Co (±25%)	Carb.Cu (±25%)	Nod.Cu (±99%)	Co.Electro (Co 99%)	Co-sep Mag (Co 55,25%)	Sable. Cobel	Anod.Sol	Scraps (Cu±30%)	Co en Granule (96%)	Con[illegible]
1	ANVIL MINING CONGO (CMCC)																										
2	BOLFAST	41489,87		675		28	240																				
3	BOSS MINING	347024,32			4672,823		2278,5											12,915									
4	CAMIS																										
5	CCC/VENTE LOCALE	12285,84										244,931	337,86749														
6	CDM	393937,89		4452,3		1239,896	840,801							1483													
7	CEPRODEV																										
8	CHEMAF	368387,46					2237							1214,25													
9	CIMCO	147020,69					1905																				
10	CMSK																										
11	CNMC HUACHIN MINING MABENDE	225650,52					2496,134																				
12	COMIDE/VENTE LOCALE	34433,82											1297,46														
13	COMILU	126607,98					1466,811																				
14	CONGO JIN JU CHENG																										
15	CROWN MINING																										
16	DRAGON INTERNATIONAL																										
17	EAGLE GROUP																										
18	FEZA MINING	66618,10		2108																							
19	FRONTIER	167332,52	9223																								
20	GCM	140904,68					1217,063			2011,72																	
21	GOLDEN AFRICAN	29587,16					355																				
22	GROUPE LA BONNE FIANCE																										
23	GTL							2446,36																			
24	HUACHIN METAL LEACH	28236,47					310,138																				
25	HUACHIN MINING	2125,22		75																							
26	KAI PENG MINING	128222,67					1271,386																				
27	KATANGA METALS																										
28	KCC	64629,39					90,155													92,032	98,6						
29	KISANFU MINING/VENTE LOCALE	34082,85											99480,26														
30	LONG FEI/VENTE LOCALE																										
31	LUNA MINING/VENTE LOCALE																										

N°	SOCIETES	MONTANTS (USD)	Conc.Cu (10-30%)	Conc.Co (4-15%)	Conc.CuCo	Cu.Noir (80-98%)	Cath.Cu (99%)	All.Blanc (30%Co ≤ 30%Cu≤	All.Rouge (±80% Cu	P-Zinc (89%Zn)		Hetero Co (Brut)	Min Cu (Brut)	Hydro.Co (±29%)	Cu.Pul (Cu 95%)	Cu.Bott (Cu 97%)	P.Plomb (P 30%)	Carb.Co (±25%)	Carb.Cu (±25%)	Nod.Cu (±99%)	Co.Electro (Co 99%)	Co-sep Mag (Co 55,25%)	Sable. Cobel	Anod.Sol	Scraps (Cu±30%)	Co en Granule (96%)	Co[illegible]
32	MAGMA																										
33	METALS MINES	45046,48					525																				
34	MIKAS VENTE LOVALE	23471,63	7642,34																								
35	MINES D'AFRIQUE																										
36	MJM																										
37	MKM	288285,56					3120,495							241,733													
38	MMG KINSEVERE	833697,35					9134,6																				
39	MMR	46971,78																									
40	MUMI EXPORT	2221856,42					19906,77							6860,23													
41	MUMI/VENTE LOCALE																										
42	OM METAL																										
43	OPERA MINING																										
44	PANCOM																										
45	RUASHI MINING	433869,76					3234,527							1745,201													
46	RUBAMIN	49752,60				716,01																					
47	SEK	190813,24					2138,054																				
48	SHITURU MINING	236212,23					2848,097																				
49	SINO KATANGA (STK)	6498,42																									
50	SODIMICO																										5[illegible]
51	SOMIKA	134418,53					1166							345,6													
52	TFM	2936844,01					18692,43							7292,665													
53	RUASHI MINING VENTE LOCALE																										
54	SEK/VENTE LOCALE																										
TOTAL		*9 806 315,48*	*16865,34*	*7310,30*	*4672,82*	*1983,91*	*75473,96*	*2446,36*	*0,00*	*2011,72*	*0,00*	*244,93*	*101115,59*	*19182,68*	*0,00*	*0,00*	*0,00*	*12,92*	*0,00*	*92,03*	*98,60*	*0,00*	*0,00*	*0,00*	*0,00*	*0,00*	*28[illegible]*

Le total général est de: Neuf millions huit cent six mille trois cent quinze dollars, quarante huit centimes

N.B: 33,75 TONNES DE CUIVRE AUTREMENT PRESENTE EXPORTER PAR BOSS MINING

Légende: Concentré de cuivre: (Conc.cu); concentré de cobalt : (Conc.co); concentré cupro-cobaltifère: (Conc.CuCo); Cathode de cuivre: (Cath.Cu); Matte de cuivre: (Matt.Cu); Hydroxyde de Cobalt: (Hydro.Co); Carbonate de cobalt: (Carb.Co); Poussière de Zinc: (Pouss.Zinc); Poussière de Plomb: (P.Plomb); Nodule du cuivre :(Nod.Cu); Alliage Rouge: (All.Rouge); Alliage Blanc : (All.Blanc); Cuivre Pulverisé: (Cu.Pul); Cuivre Bottom: (Cu.Bott); Hétérogénite de cobalt: (Hetero.Co); Minerais de cuivre: (Min.Cu)

Fait à Lubumbashi, le 07 Novembre 2015
Emmanuel KIANDA KIBAWA

République Démocratique du Congo

PROVINCE DU HAU-KATANGA
DIVISION PROVINCIALE DES
MINES/KATANGA-SUD
LUBUMBASHI

STATISTIQUES DES NOTES DE DEBIT RELATIVES A LA REDEVANCE MINIERE EMISES MOIS DE NOVEMBRE/2015

N°	SOCIETES	MONTANTS (USD)	Conc.Cu (10-30%)	Conc.Co (4-15%)	Conc.CuCo	Cu.Noir (80-98%)	Cath.Cu (99%)	All.Blanc (30%Co ≤ 30%Cu≤	All.Rouge (±80% Cu	P-Zinc (89%Zn)		Hetero Co (Brut)	Min Cu (Brut)	Hydro.Co (±29%)	Cu.Pul (Cu 95%)	Cu.Bott (Cu 97%)	P.Plomb (P 30%)	Carb.Co (±25%)	Carb.Cu (±25%)	Nod.Cu (±99%)	Co.Electro (Co 99%)	Co-sep Mag (Co 55,25%)	Sable. Cobel	Anod.Sol	Scraps (Cu±30%)	Co en Granule (96%)
1	ANVIL MINING CONGO (CMCC)																									
2	BOLFAST	42406,88		850		30	210																			
3	BOSS MINING	347309,07		763,221	3584,039		2487											25,003								
4	CAMIS																									
5	CCC/VENTE LOCALE																									
6	CDM	454277,51		313,2		1553,08	650,601							3466,2												
7	CEPRODEV																									
8	CHEMAF	374475,31					2300							1513,5												
9	CIMCO	144924,75					1998,778																			
10	CMSK																									
11	CNMC HUACHIN MINING MABENDE	129579,45					1433,401																			
12	COMIDE/VENTE LOCALE	49951,21	1828,29																							
13	COMILU	32183,59					380,68																			
14	CONGO JIN JU CHENG																									
15	CROWN MINING	6376,80																								
16	DRAGON INTERNATIONAL	22623,60				320																				
17	EAGLE GROUP																									
18	FEZA MINING	69144,59		2236,44																						
19	FRONTIER	547471,59	27730																							
20	GCM	216810,75					1848,004		202	1343,52					300,13			150,7								
21	GOLDEN AFRICAN	10275,22					127,498																			
22	GROUPE LA BONNE FIANCE																									
23	GTL							2637,82																		
24	HUACHIN METAL LEACH	141130,14					1561,174																			
25	HUACHIN MINING																									
26	KAI PENG MINING	140863,88					1481,278																			
27	KATANGA METALS																									
28	KCC																									
29	KISANFU MINING/VENTE LOCALE																									
30	MANONO MINERALS	8155,39																								
31	LUNA MINING/VENTE LOCALE																									

N°	SOCIETES	MONTANTS (USD)	Conc.Cu (10-30%)	Conc.Co (4-15%)	Conc.CuCo	Cu.Noir (80-98%)	Cath.Cu (99%)	All.Blanc (30%Co ≤ 30%Cu≤	All.Rouge (±80% Cu	P-Zinc (89%Zn)		Hetero Co (Brut)	Min Cu (Brut)	Hydro.Co (±29%)	Cu.Pul (Cu 95%)	Cu.Bott (Cu 97%)	P.Plomb (P 30%)	Carb.Co (±25%)	Carb.Cu (±25%)	Nod.Cu (±99%)	Co.Electro (Co 99%)	Co-sep Mag (Co 55,25%)	Sable. Cobel	Anod.Sol	Scraps (Cu±30%)	Co en Granule (96%)
32	MAGMA																									
33	METALS MINES	61570,96		1400			375																			
34	MIKAS VENTE LOVALE	22202,19	6621,07																							
35	MINES D'AFRIQUE																									
36	MJM																									
37	MKM	306259,92					3053,839							549,219												
38	MMG KINSEVERE	507774,54					6023,665																			
39	MMR	27339,74																								
40	MUMI EXPORT	1904043,62					17971,41							5552,67												
41	MUMI/VENTE LOCALE																									
42	OM METAL																									
43	OPERA MINING																									
44	PANCOM																									
45	RUASHI MINING	358804,16					3299,705							973,18												
46	RUBAMIN	65929,67				941,796																				
47	SEK	172869,14					1976,941																			
48	SHITURU MINING	238174,73					2946,862																			
49	SINO KATANGA (STK)																									
50	SODIMICO																									
51	SOMIKA	129069,72					1250							197,7												
52	TFM	2627965,32					15296,6							6913,136												
53	RUASHI MINING VENTE LOCALE																									
54	SEK/VENTE LOCALE																									
TOTAL		*9 159 963,43*	*36179,36*	*5562,86*	*3584,04*	*2844,88*	*66672,43*	*2637,82*	*202,00*	*1343,52*	*0,00*	*0,00*	*0,00*	*19165,61*	*300,13*	*0,00*	*0,00*	*175,70*	*0,00*	*0,00*	*0,00*	*0,00*	*0,00*	*0,00*	*0,00*	*0,00*

Le total général est de: Neuf millions cent cinquante neuf mille neuf cent soixante trois dollars, quarante trois

N.B:

Légende: Concentré de cuivre: (Conc.cu); concentré de cobalt : (Conc.co); concentré cupro-cobaltifère: (Conc.CuCo); Cathode de cuivre: (Cath.Cu); Matte de cuivre: (Matt.Cu); Hydroxyde de Cobalt: (Hydro.Co); Carbonate de cobalt: (Carb.Co); Poussière de Zinc: (Pouss.Zinc), Poussière de Plomb: (P.Plomb); Nodule du cuivre :(Nod.Cu); Alliage Rouge: (All.Rouge); Alliage Blanc : (All.Blanc), Cuivre Pulverisé: (Cu.Pul); Cuivre Bottom: (Cu.Bott); Hétérogénite de cobalt: (Hetero.Co); Minerais de cuivre: (Min.Cu)

Fait à Lubumbashi, le 04 Décembre 2015
Emmanuel KIANDA KIBAWA

PROVINCE DU HAU-KATANGA
DIVISION PROVINCIALE DES MINES/KATANGA-SUD
LUBUMBASHI

STATISTIQUES DES NOTES DE DEBIT RELATIVES A LA REDEVANCE MINIERE EMISES MOIS DE DECEMBRE/2015

N°	SOCIETES	MONTANTS (USD)	Conc.Cu (10-30%)	Conc.Co (4-15%)	Conc.CuCo	Cu.Noir (80-98%)	Cath.Cu (99%)	All.Blanc (30%Co ≤ 30%Cus	All.Rouge (±80% Cu	P-Zinc (89%Zn)	Mat-Cu (±40%)	Hetero Co (Brut)	Min Cu (Brut)	Hydro.Co (±29%)	Cu.Pul (Cu 95%)	Cu.Bott (Cu 97%)	P.Plomb (P 30%)	Carb.Co (±25%)	Carb.Cu (±25%)	Nod.Cu (±99%)	Co.Electro (Co 99%)	Co-sep Mag (Co 55,25%)	Sable. Cobel	Anod.Sol	Scraps (Cu±30%)	Co en Granule (96%)
1	ANVIL MINING CONGO (CMCC)		22200																							
2	BOLFAST	20917,44		425			120																			
3	BOSS MINING	238384,71		2725,12	817,979		1950																			
4	CAMIS																	12,722								
5	CCC/VENTE LOCALE																									
6	CDM	304335,99		89,1		976,368	870,882				64,8			1973,5												
7	CEPRODEV																									
8	CHEMAF	255718,90					1908																			
9	CIMCO	41379,16					604,553							799,5												
10	CMSK																									
11	CNMC HUACHIN MINING MABENDE	169132,67					2002,241																			
12	COMIDE/VENTE LOCALE	37757,07	1487,23																							
13	COMILU	51207,16					655,891																			
14	CONGO JIN JU CHENG																									
15	CROWN MINING																									
16	DRAGON INTERNATIONAL	27091,77		252		360																				
17	NORD KAT GROUP	20367,82																								
18	FEZA MINING	33953,61		55,86				240																		
19	FRONTIER	637784,76	36844																							
20	GCM	134710,60					1242,164		306	285					60,004						10	20				
21	GOLDEN AFRICAN	8981,90					123,97																			
22	GROUPE LA BONNE FIANCE																									
23	GTL							3048,82																		
24	HUACHIN METAL LEACH	110947,46					1266,234																			
25	HUACHIN MINING																									
26	KAI PENG MINING	52543,75					595,717																			
27	KATANGA METALS																									
28	KCC	10533,86																								
29	KISANFU MINING/VENTE LOCALE																				25					
30	MANONO MINERALS																									
31	LUNA MINING/VENTE LOCALE																									

N°	SOCIETES	MONTANTS (USD)	Conc.Cu (10-30%)	Conc.Co (4-15%)	Conc.CuCo	Cu.Noir (80-98%)	Cath.Cu (99%)	All.Blanc (30%Co ≤ 30%Cu≤	All.Rouge (±80% Cu	P-Zinc (89%Zn)	Mat-Cu (±40%)	Hetero Co (Brut)	Min Cu (Brut)	Hydro.Co (±29%)	Cu.Pul (Cu 95%)	Cu.Bott (Cu 97%)	P.Plomb (P 30%)	Carb.Co (±25%)	Carb.Cu (±25%)	Nod.Cu (±99%)	Co.Electro (Co 99%)	Co-sep Mag (Co 55,25%)	Sable. Cobel	Anod.Sol	Scraps (Cu±30%)	Co en Granule (96%)
32	MAGMA																									
33	METALS MINES	84880,86		3225			225																			
34	MIKAS VENTE LOVALE	20105,59	6806,26																							
35	MINES D'AFRIQUE																									
36	MJM	27370,00		1288																						
37	MKM	140394,52					1639,07							70,026												
38	MMG KINSEVERE	357682,02					4487,632																			
39	MMR	31569,18																								
40	MUMI EXPORT	1721534,42					17728,2							6224,25												
41	MUMI/VENTE LOCALE																									
42	OM METAL																									
43	OPERA MINING																									
44	PANCOM																									
45	RUASHI MINING	391300,11					2899,679							2045,272												
46	RUBAMIN	62326,45				964,797																				
47	SEK	121350,92					1491,154																			
48	SHITURU MINING	178892,85					2484,949																			
49	SINO KATANGA (STK)	2873,92																								
50	SICOMINES		241,88																							
51	SOMIKA	127377,98					1292							303,3												
52	TFM	2536979,95					16567,41							8097,091												
53	RUASHI MINING VENTE LOCALE																									
54	SEK/VENTE LOCALE																									
TOTAL		*7 960 387,39*	*67579,37*	*8060,08*	*817,98*	*2301,17*	*60154,75*	*3288,82*	*306,00*	*285,00*	*64,80*	*0,00*	*0,00*	*19512,94*	*60,00*	*0,00*	*0,00*	*12,72*	*0,00*	*0,00*	*35,00*	*20,00*	*0,00*	*0,00*	*0,00*	*0,00*

Le total général est de: Sept millions neuf cent soixante mille trois cent quatre vingt sept dollars, trente neuf centimes

N.B:

Légende: Concentré de cuivre: (Conc.cu); concentré de cobalt : (Conc.co); concentré cupro-cobaltifère: (Conc.CuCo); Cathode de cuivre: (Cath.Cu); Matte de cuivre: (Matt.Cu); Hydroxyde de Cobalt: (Hydro.Co); Carbonate de cobalt: (Carb.Co); Poussière de Zinc: (Pouss.Zinc); Poussière de Plomb: (P.Plomb); Nodule du cuivre :(Nod.Cu); Alliage Rouge: (All.Rouge); Alliage Blanc : (All.Blanc); Cuivre Pulverisé: (Cu.Pul); Cuivre Bottom: (Cu.Bott), Hétérogénite de cobalt: (Hetero.Co); Minerais de cuivre: (Min.Cu)

Fait à Lubumbashi, le 04 Janvier 2016
Emmanuel KIANDA KIBAWA

République Démocratique du Congo

PROVINCE DU KATANGA
DIVISION PROVINCIALE DES MINES
LUBUMBASHI
Le Chef de Division

STATISTIQUES DES NOTES DE DEBIT RELATIVES A LA REDEVANCE MINIERE EMISES DE JANVIER A DECEMBRE/2015

Produits Miniers par tonnes

N°	SOCIETES	MONTANTS REDEVANCE MINIERE (USD)	Conc.Cu (10-30%)	Conc.Co (4-15%)	Conc.CuCo	Cu.Noir (80-98%)	Cath.Cu (99%)	All.Blanc (30%Co ≤ 30%Cu≤	All.Rouge (±80% Cu	P-Zinc (89%Zn)	Mat-Cu (±40%)	Hetero Co (Brut)	Min Cu (Brut)	Hydro.Co (29%)
1	ANVIL MINING CONGO (CMCC)	0,00	49 400,00											
2	BOLFAST	368 695,53		8 190,00	0,00	58,00	1 500,00							
3	BOSS MINING	4 932 272,84		30 941,13	47 600,52	0,00	28 659,90							8,18
4	CAMIS	1 116,70		0,00	0,00	0,00	0,00							0,00
5	CCC/VENTE LOCALE	45 781,21									0,00	1 228,28	666,34	0,00
6	CDM	4 892 617,94		52 419,60	0,00	19 392,87	9 717,24				935,55	0,00	0,00	12 610,50
7	CEPRODEV	2 372,82					0,00							0,00
8	CHEMAF	3 930 443,80					24 432,00							11 106,75
9	CIMCO	826 214,10					10 551,33							
10	CMSK	198 110,94			7 385,43	0,00	0,00							
11	CNMC HUACHIN MINING MABENDE	1 710 406,82			0,00	0,00	18 014,85							
12	COMIDE/VENTE LOCALE	631 021,99	17 846,68	0,00	0,00	0,00	0,00				0,00	0,00	2 637,89	
13	COMILU	944 380,64	0,00	0,00	0,00	0,00	10 309,12				0,00	0,00	0,00	
14	CONGO JIN JU CHENG	43 066,91				0,00	462,19							
15	CROWN MINING	48 318,74				0,00	0,00							
16	DRAGON INTERNATIONAL	112 985,29		252,00		1 453,00	0,00							
17	NORD KAT GOUP	20 367,82				0,00	0,00							
18	FEZA MINING	637 313,04	0,00	11 270,30				1 893,00	0,00	0,00	0,00			
19	FRONTIER	6 459 443,32	300 531,90	0,00				0,00	0,00	0,00	0,00			
20	GCM	2 439 904,78			8 429,45	168,67	15 346,55	0,00	2 610,00	19 336,58	0,00			
21	GOLDEN AFRICAN	280 507,85			0,00	0,00	3 230,47							
22	GROUPE LA BONNE FIANCE	12 616,00					0,00							
23	GTL	0,00					0,00	27 815,42	0,00	0,00	0,00			
24	HUACHIN METAL LEACH	1 201 075,90					12 547,98							
25	HUACHIN MINING	623 251,28	0,00	11 575,00	0,00	3 543,00	0,00							
26	KAI PENG MINING	847 750,71	0,00	0,00	0,00	0,00	8 180,99							
27	KATANGA METALS	24 967,64	0,00	0,00	0,00	274,73	0,00							
28	KCC	14 223 423,29	0,00	0,00	0,00	0,00	117 292,69							0,00
29	KISANFU MINING/VENTE LOCALE	293 925,76	0,00	0,00	0,00	0,00	0,00					0,00	167 306,85	0,00
30	MANONO MINERALS	8 155,39	0,00											
31	LUNA MINING/VENTE LOCALE	76 858,07	10 942,55											
32	MAGMA	109 425,60	0,00	0,00	0,00	1 350,00	0,00							

N°	SOCIETES	Cu.Pul (Cu 95%)	Cu.Bott (Cu 97%)	P.Plomb (P 30%)	Carb.Co (±25%)	Carb.Cu (±25%)	Nod.Cu (±97%)	Co.Electro (Co 99%)	Co-sep Mag (Co 55,25%)	Sable. Cobel	Anod.Sol	Scraps (Cu±30 %)	Conc.Wolframite
1	ANVIL MINING CONGO (CMCC)												
2	BOLFAST												
3	BOSS MINING	0,00	0,00	0,00	557,30	0,00	66,75						
4	CAMIS	0,00	0,00	0,00	0,00	0,00	0,00						
5	CCC/VENTE LOCALE												
6	CDM												
7	CEPRODEV												
8	CHEMAF												
9	CIMCO												
10	CMSK												
11	CNMC HUACHIN MINING MABENDE												
12	COMIDE/VENTE LOCALE												
13	COMILU												
14	CONGO JIN JU CHENG												
15	CROWN MINING												
16	DRAGON INTERNATIONAL												
17	NORD KAT GOUP												
18	FEZA MINING												
19	FRONTIER												
20	GCM	685,23	0,00	2 291,50	280,70	0,00	0,00	240,00	30,00	30,00	0,00	0,00	0,00
21	GOLDEN AFRICAN												
22	GROUPE LA BONNE FIANCE												
23	GTL												
24	HUACHIN METAL LEACH												
25	HUACHIN MINING												
26	KAI PENG MINING												
27	KATANGA METALS												
28	KCC						503,26	2 989,50	0,00	0,00	0,00	0,00	0,00
29	KISANFU MINING/VENTE LOCALE												
30	MANONO MINERALS												
31	LUNA MINING/VENTE LOCALE												
32	MAGMA												

N°	SOCIETES	MONTANTS REDEVANCE MINIERE (USD)	Conc.Cu (10-30%)	Conc.Co (4-15%)	Conc.CuCo	Cu.Noir (80-98%)	Cath.Cu (99%)	All.Blanc (30%Co ≤ 30%Cu≤	All.Rouge (±80% Cu	P-Zinc (89%Zn)	Mat-Cu (±40%)	Hetero Co (Brut)	Min Cu (Brut)	Hydro.Co (29%)	Cu.Pul (Cu 95%)	Cu.Bott (Cu 97%)	P.Plomb (P 30%)	Carb.Co (±25%)	Carb.Cu (±25%)	Nod.Cu (±97%)	Co.Electro (Co 99%)	Co-sep Mag (Co 55,25%)	Sable. Cobel	Anod.Sol	Scraps (Cu±30 %)	Conc.Wolframite	
33	METALS MINES	1 200 779,62	0,00	32 600,00			3 300,00																				
34	MIKAS VENTE LOVALE	220 852,34	70 633,43	0,00			0,00																				
35	MINES D'AFRIQUE	6 885,00																									
36	MJM	219 847,69	0,00	4 394,00	0,00	0,00	1 121,00							0,00													
37	MKM	2 215 181,89	0,00	0,00	0,00	0,00	21 984,44							2 216,77													
38	MMG KINSEVERE	8 036 224,71					81 931,30							0,00													
39	MMR	441 514,34					0,00							0,00													2
40	MUMI EXPORT	24 400 825,78					210 360,87							60 387,63	0,00	0,00	0,00	0,00	0,00	2 110,61	0,00	0,00	0,00	0,00	0,00	0,00	
41	MUMI/VENTE LOCALE	83 085,41	0,00	0,00	12 226,47	0,00	0,00																				
42	OM METAL	9 172,72	0,00	0,00	0,00	0,00	89,92																				
43	OPERA MINING	8 758,00				0,00	0,00																				
44	PANCOM	173 253,01				1 932,23	0,00																				
45	RUASHI MINING	5 058 405,35				0,00	35 064,21							19 750,19	0,00	0,00	0,00	0,00	0,00	0,00	0,00	0,00	0,00	0,00	0,00	0,00	
46	RUBAMIN	814 505,12				10 681,08	0,00																				
47	SEK EXPORT	2 602 492,98	2 947,50				26 672,71																				
48	SHITURU MINING	2 811 781,33					32 232,39																				
49	SINO KATANGA (STK)	55 618,43					0,00																				
50	SICOMINES	0,00	241,88	0,00	1 536,00	0,00	0,00																				
51	SOMIKA	1 813 981,42	0,00	1 202,40			13 808,00							4 553,50													
52	TFM	34 253 410,87	0,00	0,00			204 471,03							86 865,12													
53	RUASHI MINING VENTE LOCALE	168,00	0,00				2,50																				
54	SEK/VENTE LOCALE	678 961,71	89 675,00				0,00																				
TOTAL		131 052 498,44	542 218,94	152 844,43	77 177,88	38 853,58	891 283,67	29 708,42	2 610,00	19 336,58	935,55	1 228,28	170 611,08	197 498,63	685,23	0,00	2 291,50	838,00	0,00	2 680,62	3 229,50	30,00	30,00	0,00	0,00	0,00	4

Le total général est de: Cent trente un millions cinquante deux mille quatre cent nonante huit dollars, quarante quatre centimes

N.B: 514,32 Tonnes de Plomb Affiné à 99% exporter par KCC

67,5 TONNES DE CUIVRE AUTREMENT PRESENTER EXPORTER PAR BOSS MINING

90 TONNES DE SULFURE DE DENICKELAGE EXPORTER PAR LA GCM

Légende. Concentré de cuivre: (Conc.cu); concentré de cobalt : (Conc.co); concentré cupro-cobaltifère: (Conc.CuCo); Cathode de cuivre: (Cath.Cu); Matte de cuivre: (Matt.Cu); Hydroxyde de Cobalt: (Hydro.Co); Carbonate de cobalt: (Carb.Co); Poussière de Zinc. (Pouss.Zinc); Poussière de Plomb: (P.Plomb); Nodule du cuivre (Nod.Cu); Alliage Rouge: (All.Rouge); Alliage Blanc : (All.Blanc); Cuivre Pulverisé: (Cu.Pul); Cuivre Bottom: (Cu.Bott); Hétérogénite de cobalt: (Hetero.Co); Minerais de cuivre: (Min.Cu). Co en Granule: Cobalt en Granule; Cassi: Cassitérite

Fait à Lubumbashi, le 05 Janvier 2016
Emmanuel KIANDA KIBAWA

République Démocratique du Congo

DIVISION PROVINCIALE DES MINES/KATANGA-SUD
LUBUMBASHI
Le Chef de Division

STATISTIQUES DES NOTES DE DEBIT RELATIVES A LA REDEVANCE MINIERE EMISES DE JANVIER A DECEMBRE/2016

Produits Miniers par tonnes

N°	SOCIETES	MONTANTS REDEVANCE MINIERE (USD)	Conc.Cu (10-30%)	Conc.Co (4-15%)	Conc.CuCo	Cu.Noir (80-98%)	Cath.Cu (99%)	All.Blanc (30%Co ≤ 30%Cu≤	All.Rouge (±80% Cu	P-Zinc (89%Zn)	Mat-Cu (±40%)	Hetero Co (Brut)	Min Cu (Brut)	Hydro.Co (29%)	Cu.Pul (Cu 95%)	Cu.Bott (Cu 97%)	P.Plomb (P 30%)	Carb.Co (±25%)	Carb.Cu (±25%)	Nod.Cu (±97%)	Co.Electro (Co 99%)	Co-sep Mag (Co 55,25%)	Sable. Cobel	Anod.Sol	Scraps (Cu±30%)	Conc.Wolframite	Conc.Etain	Conc.Tantal
1	ANVIL MINING CONGO (CMCC)	0,00	11 030,00																								0,00	0,00
2	BEL AIR AFRICAN METALS	0,00		0,00	0,00	0,00	0,00																				49,62	0,00
3	BOLFAST	150 456,48		1 910,84	0,00	266,50	1 194,00																				0,00	0,00
4	BOSS MINING	4 034 735,56		7 837,63	58 854,45	0,00	26 308,25											517,63	0,00									
5	CAMIS	0,00		0,00	0,00	0,00	0,00											0,00	0,00									
6	CCC/VENTE LOCALE	223 288,41		0,00	0,00	0,00	0,00					2 322,77	5 918,31	0,00	0,00													
7	CDM	3 579 398,15		3 271,16	0,00	9 259,88	12 732,17					0,00	0,00	24 807,00	0,00													
8	CEPRODEV	0,00					0,00					0,00	0,00	0,00	0,00											0,00	25,01	0,00
9	CHEMAF	2 411 814,75					18 511,63					0,00	0,00	7 512,94	0,00											0,00	300,00	0,00
10	CIMCO	875 391,11					11 926,43					0,00	0,00	0,00	0,00											0,00	0,00	0,00
11	CNMC HUACHIN MINING MABENDE	1 988 707,02					23 696,69																			0,00		
12	COMIDE/VENTE LOCALE	291 118,79	11 070,32	0,00	1 593,76	0,00	0,00																			0,00		
13	COMILU	1 695 628,87	5 240,12	0,00	0,00	0,00	20 605,55																					
14	CONGO JIN JU CHENG	181 200,77				0,00	2 161,80																					
15	CROWN MINING	39 380,32				161,35	0,00																				125,00	78,00
16	DRAGON INTERNATIONAL	203 452,43				2 539,12	0,00																					
17	FEZA MINING	127 316,47	0,00	1 202,32			0,00	598,00	0,00	0,00	0,00	0,00			0,00						0,00							
18	FRONTIER	6 183 155,66	361 856,40	0,00			0,00	0,00	0,00	0,00	0,00	0,00			0,00						0,00							
19	GCM	1 274 452,36	0,00	0,00			10 038,86	0,00	315,00	19 202,17	150,00	0,00			552,09						50,00							
20	GOLDEN AFRICAN	259 174,20	0,00	0,00			3 345,12	0,00										32,00	0,00	0,00	0,00							
21	COMIKA	868 130,16	0,00	0,00	31 020,15	0,00	0,00	0,00																				
22	GTL	0,00					0,00	20 439,65																				
23	HUACHIN METAL LEACH	1 019 503,73					12 045,76	0,00																	0,00	0,00		
24	GRANDE CIMENTERIE DU KATANGA	2 026,68					0,00	0,00																	0,00	0,00		
25	KAI PENG MINING	1 250 854,48					12 329,67	0,00																	1 212,97	0,00		
26	KATANGA METALS	49 225,20	0,00	0,00	0,00	750,00	0,00	0,00																	0,00	0,00		
27	KCC	19 800,82	0,00	0,00	0,00	0,00	8,63	0,00										0,00	0,00	83,48	31,64	0,00	0,00	0,00	0,00	0,00		
28	KISANFU MINING/VENTE LOCALE	55 369,66											11 162,52	0,00														
29	LUNA MINING/VENTE LOCALE	0,00											0,00	0,00														
30	LUISHA MINING	152 435,75	0,00	0,00	0,00	2 178,14	0,00	0,00	0,00	0,00	0,00	0,00	0,00	0,00														

[illegible]	[illegible]	1 019 716,27	0,00	15 810,00			3 555,00						0,00	4 005,00														
33	[illegible]IKAS VENTE LOVALE	17 686,11	0,00	0,00			0,00						0,00	0,00														
34	IVERLAND MINING/VENTE LOCALE	113 670,96	0,00	0,00			0,00						183 680,05	0,00														
35	MJM	535 224,29	0,00	14 228,00			2 324,94				330,00	0,00	0,00	0,00														
36	MKM	1 765 362,14					19 430,64				0,00	0,00	0,00	2 855,36														
37	MMG KINSEVERE	7 019 746,47					81 246,06							0,00														
38	MMR	465 255,96					0,00							0,00				0,00	0,00	0,00	0,00	0,00	0,00	0,00	0,00	0,00	3 042,00	294,82
39	MUMI EXPORT	19 987 156,84					196 088,52							69 709,85				0,00	0,00	17 948,78	0,00	0,00	0,00	0,00	0,00	0,00	0,00	
40	MUMI/VENTE LOCALE	0,00					0,00							0,00													0,00	
41	NORD KAT GROUP	63 165,72					0,00							0,00													519,09	
42	OM METAL	0,00					0,00							0,00													0,00	
43	OPERA MINING	0,00					0,00							0,00													0,00	
44	PANCOM	0,00					0,00							0,00													0,00	
45	RUASHI MINING	3 957 103,74					32 510,97							14 288,86				0,00	0,00	20,79	0,00	0,00	0,00	0,00	0,00	0,00	0,00	
46	RUASHI MINING VENTE LOCALE	0,00	0,00	0,00	0,00	0,00	0,00																				0,00	
47	RUBAMIN	981 257,17	0,00	0,00	0,00	15 281,89	0,00																				0,00	
48	SEK	2 022 991,99	2 419,39	0,00	0,00	0,00	21 157,67																				0,00	
49	SEK/VENTE LOCALE	0,00	0,00				0,00																				0,00	
50	SHITURU MINING	2 563 257,71	0,00				33 582,73																				0,00	
51	SICOMINES	0,00	128 613,41				44 114,97																				0,00	
52	SINO KATANGA (STK)	75 534,45					0,00																				399,87	49,90
53	SOMIKA	1 290 588,85					15 478,00				0,00	0,00	0,00	0,00				0,00	0,00	0,00	0,00	0,00	0,00	0,00	0,00	0,00	0,00	0,00
54	TFM	29 309 960,37					214 498,26				0,00	0,00	0,00	95 217,20				0,00	0,00	0,00	0,00	0,00	0,00	0,00	0,00	0,00	0,00	0,00
TOTAL		*98 123 696,86*	*520 229,63*	*44 259,95*	*91 468,35*	*30 436,88*	*818 892,32*	*21 037,65*	*315,00*	*19 202,17*	*480,00*	*2 322,77*	*200 760,88*	*218 396,20*	*552,09*	*0,00*	*0,00*	*549,63*	*0,00*	*18 053,06*	*81,64*	*0,00*	*0,00*	*0,00*	*1 212,97*	*0,00*	*4 460,58*	*422,72*

Le total général est de: Nonante huit millions cent vingt trois mille six cent nonante six dollars, quatre vingt centimes

N.B: 447,25 Tonnes de Cuivre Autrement présenté exporté par BOSS MINING
3,961 Kg d'or exporté par le Comproir ROMINERAL
100,939 Tonnes de Plomb Affiné exporté par KCC
9.802,2 Tonnes de Calcalire à ciment vendu localement par GCK

Légende: Concentré de cuivre: (Conc.cu); concentré de cobalt : (Conc.co); concentré cupro-cobaltifère: (Conc.CuCo); Cathode de cuivre: (Cath.Cu); Matte de cuivre: (Matt.Cu); Hydroxyde de Cobalt: (Hydro.Co); Carbonate de cobalt: (Carb.Co); Poussière de Zinc: (Pouss.Zinc); Poussière de Plomb: (P.Plomb); Nodule du cuivre :(Nod.Cu); Alliage Rouge: (All.Rouge); Alliage Blanc : (All.Blanc); Cuivre Pulverisé: (Cu.Pul); Cuivre Bottom: (Cu.Bott); Hétérogénite de cobalt: (Hetero.Co); Minerais de cuivre: (Min.Cu), Co en Granule: Cobalt en Granule; Cassi: Cassitérite

Fait à Lubumbashi, le 06 Janvier 2017
Emmanuel KIANDA KIBAWA

République Démocratique du Congo

DIVISION PROVINCIALE DES MINES/KATANGA-SUD
LUBUMBASHI
Le Chef de Division

STATISTIQUES DES NOTES DE DEBIT RELATIVES A LA REDEVANCE MINIERE EMISES DE JANVIER A DECEMBRE 2017 (EX KATANGA DECLAREES A LUBUMBASHI)

Produits Miniers par tonnes

N°	SOCIETES	MONTANTS REDEVANCE MINIERE (USD)	Conc.Cu (10-30%)	Conc.Co (4-15%)	Conc.CuCo	Cu.Noir (80-98%)	Cath.Cu (99%)	All.Blanc (30%Co ≤ 30%Cu≤	All.Rouge (±80% Cu	P-Zinc (89%Zn)	Mat-Cu (±40%)	Hetero Co (Brut)	Min Cu (Brut)	Hydro.Co (29%)	Cu.Pul (Cu 95%)	Cu.Autrement présenté (Cu 99%)	P.Plomb (P 30%)	Carb.Co (±25%)	Carb.Cu (±25%)	Nod.Cu (±97%)	Co.Electro (Co 99%)	Cendre de Zinc (Zinc 32%)	CO.SEPT MAGNETIQUE	Anod.Sol	Scraps (Cu±99%)	Conc.Wolframite	Conc.Etain
1	KICC	517 912,60	11 145,86	0,00																							
2	COMMUS/ VENTE LOCALE	80 801,64	13 251,50	0,00																							
3	BOLFAST	21 719,20		200,00			180,00																				
4	BOSS MINING	6 459 144,51		45 532,77	5 038,04	0,00	37 295,00									240,25	0,00	3 074,04									
5	CCC/VENTE LOCALE	1 717,64		0,00		0,00	0,00				0,00	27,51	60,30	0,00													
6	CDM	7 026 234,81		2 583,49		8 386,38	17 752,85				0,00			25 117,97													
7	COMMUS	1 305 850,62	9 198,33	0,00		9 412,55	0,00				1 465,43			0,00													
8	CHEMAF	6 488 885,99					28 186,50							18 601,94													550,00
9	CIMCO	213 123,32					2 387,66							0,00													
10	CNMC HUACHIN MINING MABENDE	3 305 691,93					29 765,06																				
11	COMIDE/VENTE LOCALE	175 107,57	26 242,78																								
12	COMIKA	1 939 143,17	5 723,88	0,00	45 823,34		0,00																				
13	COMILU	1 976 669,76					21 358,37																				
14	CONGO JIN JU CHENG	324 733,74					2 961,22																				
15	CROWN MINING	41 073,60					0,00																				82,00
16	DRAGON INTERNATIONAL	56 884,14				403,85	241,96																				
17	FRONTIER	9 049 959,35	378 224,85				0,00		0,00	0,00					0,00										0,00		
18	GCM	1 921 744,24					11 757,18		410,39	17 665,30					1 390,04					0,00	120,00	2 492,49	100,00	0,00	87,08		
19	GOLDEN AFRICAN	511 583,04					4 621,90						0,00	272,00	0,00												
20	GRANDE CIMENTERIE DU KATANGA	5 749,10					0,00																				
21	GTL	0,00			0,00		0,00	14 272,14																			
22	HUACHIN METAL LEACH	1 481 237,37			0,00		13 460,13	0,00																			
23	IVERLAND MINING/VENTE LOCALE	431 951,05			37 816,74		1 753,83	0,00					401 650,55														
24	KAI PENG MINING	2 011 933,60			0,00		16 908,87	0,00					0,00														
25	KATANGA METALS	144 758,51			0,00	1 672,48	0,00																				
26	KCC	342 338,56			80 329,38																						
27	COPROCO GROUP	48 880,70																									350,82
28	LUISHA MINING	84 045,59		384,00		891,00	0,00																				
29	METALS MINES	2 495 492,02		19 734,32			5 318,00							6 042,69	0,00												
30	MJM	1 858 672,14		30 878,58			3 281,78							0,00	0,00												
31	MKM	3 341 037,39		0,00			25 723,49							4 511,30	0,00												
32	MMG KINSEVERE	8 934 404,49					79 504,36																				
33	MMR	594 121,39					0,00																				3 872,50
34	MUTANDA MINING	31 393 266,84					163 709,32							80 175,13	0,00				29 200,22					3 643,68	0,00		0,00
35	OM METAL	319 325,76					2 166,00								748,00												0,00
36	NORD KAT GROUP	52 831,86					0,00																				424,33

37	RUASHI MINING	7 386 220,44					31 285,67							18 386,16													
38	RUBAMIN	1 670 209,65				20 262,00	0,00	180,17																			
39	SEK	1 991 791,20				0,00	17 738,02																				
40	SHITURU MINING	3 638 486,77				0,00	35 646,89																				
41	SICOMINES	0,00	193 516,20				35 951,36							1 582,52													25,13
42	SINO KATANGA (STK)	76 375,47																									375,39
43	SOMIKA	3 291 882,18		23 571,96		0,00	18 352,00							2 618,00													
44	TFM	55 894 403,71		0,00		0,00	215 364,93							111 946,07													
45	TSM	685 562,78		1 997,50		5 428,49	825,58																				
46	SODIMIKA /VENTE LOCALE	9 379,00					0,00																				
47	RUASHI MINING/VENTE LOCALE	7,81					0,09																				
48	MINING PROGRESS	9 505,09		180,96																							
49	ROCBELT CONGO	30 852,78		0,00																							248,23
50	THOMAS MINING	399 385,78		825,00	0,00	3 068,48	0,00																				
51	CNMC CONGO COMPAGNIE MINIERE	220 503,36		0,00			1 728,78																				
52	BLUE STAR CAPITAL	39 603,74		660,00			0,00																				
53	SEK/VENTE LOCALE	976,80		0,00			0,00																	27,90		
54	KCC/EXPORT	172 473,00		0,00			1 345,19																	0,00		
TOTAL		***170 475 646,80***	***637303,41***	***126548,6***	***169007,5***	***49525,21***	***826571,99***	***14452,311***	***410,386***	***17665,3***	***1465,428***	***27,51***	***401710,85***	***269253,78***	***2138,04***	***240,25***	***0***	***3074,04***	***0***	***29200,22***	***120***	***2492,49***	***100***	***0***	***3758,662***	***0***	***5928,403***

Le total général est de: Cent septante millions quatre cent septante cinq mille six cent quarante six dollars, quatre vingt centi

NB: 47,157 Tonnes de Quartz exportés par le Comptoir CRISTAL QUARTZ
90 Tonnes de cobalt sulfuré exporté par la GCM
20.665,4 Tonnes de ciment à calaire vendu par GCK

Légende: Concentré de cuivre: (Conc.cu); concentré de cobalt : (Conc.co); concentré cupro-cobaltifère: (Conc.CuCo); Cathode de cuivre: (Cath.Cu); Matte de cuivre: (Matt.Cu); Hydroxyde de Cobalt: (Hydro.Co); Carbonate de cobalt: (Carb.Co); Poussière de Zinc: (Pouss.Zinc); Poussière de Plomb: (P.Plomb); Nodule du cuivre :(Nod.Cu); Alliage Rouge: (All.Rouge); Alliage Blanc : (All.Blanc); Cuivre Pulverisé: (Cu.Pul); Cuivre Bottom: (Cu.Bott); Hétérogénite de cobalt: (Hetero.Co); Minerais de cuivre: (Min.Cu), Co en Granule: Cobalt en Granule; Cassi: Cassitérite

Fait à Lubumbashi, le 06 janvier 2018
Emmanuel KIANDA KIBAWA

République Démocratique du Congo

DIVISION PROVINCIALE DES MINES
DU HAUT-KATANGA
LUBUMBASHI
Le Chef de Division

STATISTIQUES DE LA REDEVANCE MINIERE PAR QUOTITE EMISES
POUR LA PERIODE DE JANVIER A DECEMBRE 2018
(HAUT-KATANGA ,GUICHET-LUBUMBASHI ,LUALABA ET FUNGURUME)

			QUANTITES DES PRODUITS EXPORTES EN TONNES																							
	SOCIETE	REDEVENCE MINIERE 100% (USD)	ALL_BLANC	Carb_Co	Cath Co	CATH_CU	CENDRE DE ZINC	CO_CATH	CO_CATH_Fine	CO_ELECTRO	Conc_Mn	CONC_CO	CONC_CU	CONC_CUCO	CONC_ETAIN	CONC_TANTAL	CU_BLST	CU_NOIR	CU_PUL	Dechet_CU	HYDRO_CO	Matt_Cu	MIN_CO	MIN_CU	POUSS_ZINC	SCRAPS
1	ANVIL MINING	1 151 031,63											29941,76													
2	BOSS MINING	8 406 558,87		5970,533		26205,5						34647,432		2318,665												
3	CDM	14 240 990,88				11693,321											2413,503	4487,896			26272,206					
4	CHEMAF	15 647 571,49				26914									599	50					28055,651					
5	CNMC CONGO COMPAGNIE MINIERE	3 015 806,82				16232,9818																				
6	CNMC HUACHIN MABENDE	5 776 326,00				32318,092																				
7	COMIKA	3 388 128,48				2192,199							346,523	46170,416							1462,5					
8	COMILU	5 591 262,18				28028,542							18164,894													
9	COMMUS	5 627 946,87											17958,534					28551,811				5164,865				
10	CONGO JIN JU	714 489,48				3920,528																				
11	COPROCO	82 199,09													384											
12	CROWN MINING	13 295,40													82											
13	FRONTIER	8 147 245,75											239629													
14	GCM	1 801 562,09		179,6		7630,506	1898,785	10	10	40									956,848						467,471	30,95
15	GOLDEN AFRICA	1 331 388,78				5804,775															725,848					
16	HUACHIN METAL LEACH	2 731 869,95				13106,065															443,75					
17	IVERLAND (VENTE LOCAL)	374 891,82				1801,462																		178032,3		
18	KAI PENG	2 859 499,11				15872,708																				
19	KATANGA METALS	83 900,31																495,792								
20	KCC	34 413 780,23			8,264	111863,322															21632,64435					2346,419
21	KICC	4 639 528,42				89,707						178,48	64731,5472	277,745												
22	LONG FEI(VENTE LOCAL)	91 703,38																						35495,42		
23	LUALABA MINING(VENTE LOCAL)	121 027,60																					11535,606	73328,418		
24	METAL MINES	9 736 007,63				7988,989						14798,7018									19557,7864					
25	METALKOL	120 223,96				570,631																				
26	MIKAS	6 322 530,42				11116,473															9148,9					
27	MJM	2 903 327,70				4887						6197,76									3763,01788					
28	MKM	3 904 147,59				17543,754															3107,474					
29	MMG	14 692 678,88				80793,983																				
30	MMG (VENTE LOCAL)	3 217,36																		23,43						
31	MMR	740 888,34													2871	306,886										
32	MPC	2 179 961,44	228			275						21856														
33	MUMI	57 092 255,36				159288,567															65614,00736					8852,591
34	OM METAL	720 177,00		33		3092						665							285							
35	ROCBELT CONGO	27 324,90														33,5803										
36	RUASHI MINING	10 020 455,45				24042,308															14352,598					
37	RUBAMIN	2 149 641,31	837,066															15438,617								
37	RUBAMIN (VENTE LOCAL)	12 200,93																						6941,582		
38	SCM	692 829,34				58562,404						34,033	99169,4185								1627,874					
39	SCMK-Mn	21 422,59									14883,901															
40	SEK	3 486 521,50				19329,047																				
40	SEK (VENTE LOCAL)	4 126,20				15,896														17,871						
41	SHAMITUMBA (VENTE LOCAL)	78 318,72										458,95576	4082,00735													
42	SHITURU MINING	5 919 724,15				34591,678																				
43	SINO KATANGA	24 500,73													76,131											

34	SOMIKA	7 207 826,45				18880						5432									10414,637			40905,1		
34	SOMIKA(VENTE LOCAL)	149 153,22																					6960,97	81935,34		
35	TCC	145 950,79				676,59104																				
36	TFM	96 774 441,02				67150,4996															44832,62462					
37	THOMAS MINING	2 669 743,96				126,592						17696,855					2039,643	4903,722								
38	TSM	87 068,37													24,839			648,84								
	TOTAL	348 138 669,96	1 065,07	6 183,13	8,26	812 605,12	1 898,79	10,00	10,00	40,00	14 883,90	101 965,22	474 023,68	48 766,83	4 036,97	390,47	4 453,15	54 526,68	1 241,85	41,30	251 011,52	5 164,87	18 496,58	416 638,16	467,47	11 229,96

e total général est de: Trois cent quarante-huit million cent trente-huit mille six cent soixante neuf dollars, cinquante-quatre centimes

NB : Novembre et decembre: sans les données statistiques de Lualaba

Légende:

Carbonate de cobalt: (Carb.Co);Cathode de cuivre: (Cath.Cu); Cobalt Electrode (Co_ELECTRO);
Concentré de Manganèse: (Conc.Mn); concentré de cobalt : (Conc.co); Concentré de cuivre: (Conc.cu); concentré cupro-cobaltifère: (Conc.CuCo);
Concentré d'Etain (Conc.ETAIN); Concentré d'Etain (Conc.TANTALE); Cuvre Blister(CU_BLST); Cuivre noir(CU.NOIR); Cuivre Pulverisé: (Cu.Pul); Hydroxyde de Cobalt: (Hydro.Co);
Matte de cuivre: (Matt.Cu); Minerais de cuivre: (Min.Cu); Minerais de cobalt: (Min.Co); Craps de cuivre(craps.Cu)

République Démocratique du Congo

DIVISION PROVINCIALE DES MINES
DU HAUT-KATANGA
LUBUMBASHI
Le Chef de Division

STATISTIQUES DE LA REDEVANCE MINIERE PAR QUOTITE EMISES
POUR LA PERIODE DE JANVIER 2019
(HAUT-KATANGA)

N°	SOCIETE	REDEVANCE 100%	TERSOR 50%	PROVINCE 25%	TERRITOIRE 15%	FONDS MINIERS 10%	QUANTITES DES PRODUITS EXPORTES EN TONNES											
							CATH_CU	CO_SEPAR	CONC_CO	CONC_CU	CONC_ETAIN	CU_BLIST	CU_DEC	CU_NOIR	CU_PULV	HYDRO_CO	Min_Co	Min_Cu
1	CDM	2 714 298,29	1 357 149,15	678 574,57	407 144,74	271 429,83	1745,11					244,35				2654,606		
2	CHEMAF	2 783 790,68	1 391 895,34	695 947,67	417 568,60	278 379,07	3254				25					2403,6317		
3	CNMC CONGO COMPAGNIE	162 652,10	81 326,05	40 663,03	24 397,82	16 265,21	750,47											
4	CNMC HUACHIN MABENDE	695 388,40	347 694,20	173 847,10	104 308,26	69 538,84	3208,493											
5	COMILU	424 857,57	212 428,79	106 214,39	63 728,64	42 485,76	1957,869											
6	CONGO JIN JU	18 596,40	9 298,20	4 649,10	2 789,46	1 859,64	85,803											
7	GCM	426 910,19	213 455,09	106 727,55	64 036,53	42 691,02	456,93	120					109,03		82,988			
8	GOLDEN AFRICAN	62 444,76	31 222,38	15 611,19	9 366,71	6 244,48	288											
9	HUACHIN METAL LEACH	236 291,26	118 145,63	59 072,82	35 443,69	23 629,13	1090,238											
10	KAIPENG	435 464,93	217 732,47	108 866,23	65 319,74	43 546,49	2009,217											
11	KICC	412 784,00	206 392,00	103 196,00	61 917,60	41 278,40				5057,2839								
12	LONG FEI(VENTE LOCALE)	21 736,24	10 868,12	5 434,06	3 260,44	2 173,62												5014,8
13	LUALABA MINING(VENTE LOCALE)	43 925,18	21 962,59	10 981,29	6 588,78	4 392,52											5225,62	16735,24
14	METAL MINES	939 559,78	469 779,89	234 889,94	140 933,97	93 955,98	632,5		158,763							881,7725		
15	MIKAS	1 467 526,54	733 763,27	366 881,63	220 128,98	146 752,65	141,832									1667,9		
16	MJM	656 783,51	328 391,76	164 195,88	98 517,53	65 678,35	478,5									641,3968		
17	MMG	953 512,11	476 756,05	238 378,03	143 026,82	95 351,21	4399,465											
18	OM METAL	13 125,00	6 562,50	3 281,25	1 968,75	1 312,50	60											
19	RUASHI MINING	586 301,46	293 150,73	146 575,36	87 945,22	58 630,15	2705,171											
20	RUBAMIN	366 729,13	183 364,56	91 682,28	55 009,37	36 672,91								2349,982				
20	RUBAMIN(VENTE LOCALE)	32 978,21	16 489,10	8 244,55	4 946,73	3 297,82												22079,25
21	SEK	157 819,81	78 909,90	39 454,95	23 672,97	15 781,98	728,174											
23	SHITURU MINING	580 003,30	290 001,65	145 000,83	87 000,50	58 000,33	2676,111											
24	SOMIKA	1 116 604,36	558 302,18	279 151,09	167 490,65	111 660,44	1404									942,96		
	**TOTAL**	**15 310 083,19**	**7 655 041,60**	**3 827 520,80**	**2 296 512,48**	**1 531 008,32**	**28 071,88**	**120,00**	**158,76**	**5 057,28**	**25,00**	**244,35**	**109,03**	**2 349,98**	**82,99**	**9 192,27**	**5 225,62**	**43 829,3**

Le total général est de: Quinze million trois cent dix mille quatre-vingt-trois dollars, dix-neuf centimes

Fait à Lubumbashi, le 05/02/2019

Pierrot MALOBA KITUMBA

Légende:

Carbonate de cobalt: (Carb.Co);Cathode de cobalt (Cath.co) : (Cath.Cu); Cathode de cuivre: (Cath.Cu); Cobalt Electrode (Co_ELECTRO);
Concentré de Manganèse: (Conc.Mn); concentré de cobalt : (Conc.co); Concentré de cuivre: (Conc.cu); concentré cupro-cobaltifère: (Conc.CuCo);
Concentré d'Etain (Conc.ETAIN); Concentré d'Etain (Conc.TANTALE); Cuvre Blister(CU_BLST); Cuivre noir(CU.NOIR); Cuivre Pulverisé: (Cu.Pul); Hydroxyde de Cobalt: (Hydro.Co);
Matte de cuivre: (Matt.Cu); Minerais de cuivre: (Min.Cu); Minerais de cobalt: (Min.Co); Craps de cuivre(craps.Cu)

République Démocratique du Congo

DIVISION PROVINCIALE DES MINES
DU HAUT-KATANGA
LUBUMBASHI

Le Chef de Division

STATISTIQUES DE LA REDEVANCE MINIERE PAR QUOTITE EMISES
POUR LA PERIODE DE FEVRIER 2019
(HAUT-KATANGA)

N°	SOCIETE	REDEVANCE 100%	TERSOR 50%	PROVINCE 25%	TERRITOIRE 15%	FONDS MINIERS 10%	QUANTITES DES PRODUITS EXPORTES EN TONNES									
							CATH_CU	CONC_CU	CONC_CUCO	CONC_ETAIN	CU_AUTRE	CU_BLIST	CU_DEC	CU_NOIR	HYDRO_CO	Min_Cu
1	CDM	3 586 774,80	1 793 387,40	896 693,70	538 016,22	358 677,48	4965,437		3322,922			515,828			3444,157	
2	CHEMAF	3 546 271,67	1 773 135,83	886 567,92	531 940,75	354 627,17	2804			25					4735,45	
3	CNMC CONGO COMPAGNIE	348 989,75	174 494,88	87 247,44	52 348,46	34 898,98	1663,595									
4	CNMC HUACHIN MABENDE	795 330,28	397 665,14	198 832,57	119 299,54	79 533,03	3780,812									
5	COMILU	585 246,82	292 623,41	146 311,70	87 787,02	58 524,68	2815,04									
6	CONGO JIN JU	79 418,99	39 709,50	19 854,75	11 912,85	7 941,90	382,548									
7	FRONTIER	836 681,93	418 340,97	209 170,48	125 502,29	83 668,19		18432								
8	GCM	190 542,84	95 271,42	47 635,71	28 581,43	19 054,28	889,612						28,201			
9	GOLDEN AFRICAN	155 150,24	77 575,12	38 787,56	23 272,54	15 515,02	742									
10	HUACHIN METAL LEACH	205 962,48	102 981,24	51 490,62	30 894,37	20 596,25	975,53									
11	KAIPENG	558 564,68	279 282,34	139 641,17	83 784,70	55 856,47	2573,664									
12	KICC	759 092,46	379 546,23	189 773,12	113 863,87	75 909,25		8803,005								
13	LONG FEI(VENTE LOCALE)	54 262,35	27 131,18	13 565,59	8 139,35	5 426,24										12519
14	LUALABA MINING(VENTE LOCALE)	8 790,71	4 395,36	2 197,68	1 318,61	879,07										4821,532
15	METAL MINES	807 781,64	403 890,82	201 945,41	121 167,25	80 778,16	813								1023,6888	
16	MIKAS	886 356,00	443 178,00	221 589,00	132 953,40	88 635,60	689,62								1171,67	
17	MJM	683 433,95	341 716,98	170 858,49	102 515,09	68 343,40	990,5								765,4804	
18	MMG	950 479,55	475 239,78	237 619,89	142 571,93	95 047,96	4578,301									
19	OM METAL	120 113,70	60 056,85	30 028,43	18 017,06	12 011,37	300				276					
20	RUASHI MINING	941 472,55	470 736,28	235 368,14	141 220,88	94 147,26	2980,698								487,01	
21	RUBAMIN	340 110,66	170 055,33	85 027,67	51 016,60	34 011,07								2256,157		
21	RUBAMIN(VENTE LOCALE)	5 693,42	2 846,71	1 423,36	854,01	569,34										2711,154
22	SEK	242 240,17	121 120,08	60 560,04	36 336,02	24 224,02	1117,686									
23	SHITURU MINING	522 485,87	261 242,94	130 621,47	78 372,88	52 248,59	2485,971									
24	SOMIKA	747 379,50	373 689,75	186 844,88	112 106,93	74 737,95	1892								500,85	
	TOTAL	**17 958 627,04**	**8 979 313,52**	**4 489 656,76**	**2 693 794,06**	**1 795 862,70**	**37 440,01**	**27 235,01**	**3 322,92**	**25,00**	**276,00**	**515,83**	**28,20**	**2 256,16**	**12 128,31**	**20 051,69**

Le total général est de: Dix sept millions neuf cent cinquante-huit mille six cent vingt-sept dollars, zéro quatre centimes

Fait à Lubumbashi, le 05/03/2019

Pierrot MALOBA KITUMBA

Légende:

Carbonate de cobalt: (Carb.Co);Cathode de cobalt (Cath.co) : (Cath.Cu); Cathode de cuivre: (Cath.Cu); Cobalt Electrode (Co_ELECTRO);
Concentré de Manganèse: (Conc.Mn); concentré de cobalt : (Conc.co); Concentré de cuivre: (Conc.cu); concentré cupro-cobaltifère: (Conc.CuCo);
Concentré d'Etain (Conc.ETAIN); Concentré d'Etain (Conc.TANTALE); Cuvre Blister(CU_BLST); Cuivre noir(CU.NOIR); Cuivre Pulverisé: (Cu.Pul); Hydroxyde de Cobalt: (Hydro.Co);
Matte de cuivre: (Matt.Cu); Minerais de cuivre: (Min.Cu); Minerais de cobalt: (Min.Co); Craps de cuivre(craps.Cu)

République Démocratique du Congo

DIVISION PROVINCIALE DES MINES
DU HAUT-KATANGA
LUBUMBASHI
Le Chef de Division

STATISTIQUES DE LA REDEVANCE MINIERE PAR QUOTITE EMISES
POUR LA PERIODE DE MARS 2019
(HAUT-KATANGA)

							QUANTITES DES PRODUITS EXPORTES EN TONNES										
N°	SOCIETE	REDEVANCE 100%	TERSOR 50%	PROVINCE 25%	TERRITOIRE 15%	FONDS MINIERS 10%	CATH_CU	CONC_CU	CONC_CUCO	CONC_ETAIN	CONC_TANTALE	CU_AUTRE	CU_BLIST	CU_NOIR	CU_PULV	HYDRO_CO	Min_Cu
1	ANVIL MINING	405 621,21	202 810,61	101 405,30	60 843,18	40 562,12		10256									
2	CDM	2 859 256,93	1 429 628,46	714 814,23	428 888,54	285 925,69	4427,783		5354,469				729,789			1867,048	
3	CHEMAF	2 585 507,63	1 292 753,82	646 376,91	387 826,14	258 550,76	1946			25						4198,439	
4	CNMC CONGO COMPAGNIE	275 437,39	137 718,69	68 859,35	41 315,61	27 543,74	1262,083										
5	CNMC HUACHIN MABENDE	764 725,29	382 362,65	191 181,32	114 708,79	76 472,53	3503,934										
6	COMIKA	6 522,77	3 261,38	1 630,69	978,42	652,28			103,536								
7	COMILU	474 468,19	237 234,10	118 617,05	71 170,23	47 446,82	2158,636										
8	CONGO JIN JU	102 179,99	51 090,00	25 545,00	15 327,00	10 218,00	465,043										
9	FRONTIER	864 487,77	432 243,88	216 121,94	129 673,17	86 448,78		18350									
10	GCM	300 225,29	150 112,64	75 056,32	45 033,79	30 022,53	1144,424								171,676		
11	GOLDEN AFRICAN	120 170,40	60 085,20	30 042,60	18 025,56	12 017,04	540										
12	HUACHIN METAL LEACH	230 836,32	115 418,16	57 709,08	34 625,45	23 083,63	1068,194										
13	KAIPENG	567 447,67	283 723,84	141 861,92	85 117,15	56 744,77	2501,784										
14	KICC	464 713,41	232 356,70	116 178,35	69 707,01	46 471,34		5047,317									
15	LONG FEI(VENTE LOCALE)	17 845,02	8 922,51	4 461,26	2 676,75	1 784,50											4060,67
16	LUALABA MINING(VENTE LOCALE)	39 797,31	19 898,66	9 949,33	5 969,60	3 979,73											25226,403
17	METAL MINES	744 630,90	372 315,45	186 157,72	111 694,63	74 463,09	1136									967,0388	
18	MIKAS	49 516,99	24 758,50	12 379,25	7 427,55	4 951,70	226,45										
19	MJM	172 835,45	86 417,72	43 208,86	25 925,32	17 283,54	420									90,5994	
20	MMG	1 060 954,89	530 477,45	265 238,72	159 143,23	106 095,49	4837,264										
20	MMG(VENTE LOCALE)	7 005,78	3 502,89	1 751,44	1 050,87	700,58	32,909										
21	OM METAL	12 663,00	6 331,50	3 165,75	1 899,45	1 266,30						54					
22	RUASHI MINING	1 118 353,86	559 176,93	279 588,47	167 753,08	111 835,39	2874,272									709,6846	
23	RUBAMIN	307 898,40	153 949,20	76 974,60	46 184,76	30 789,84								1961,866			
23	RUBAMIN(VENTE LOCALE)	73 143,25	36 571,62	18 285,81	10 971,49	7 314,32											50038,111
24	SEK	115 087,76	57 543,88	28 771,94	17 263,16	11 508,78	523,789										
25	SHITURU MINING	751 137,61	375 568,81	187 784,40	112 670,64	75 113,76	3420,516										
26	SINO KATANGA	26 152,38	13 076,19	6 538,10	3 922,86	2 615,24				15,42	4,67						
27	SOMIKA	422 745,34	211 372,67	105 686,34	63 411,80	42 274,53	1924										
28	TSM	14 878,07	7 439,03	3 719,52	2 231,71	1 487,81				47,332							
	TOTAL	**14 956 246,27**	**7 478 123,13**	**3 739 061,57**	**2 243 436,94**	**1 495 624,63**	**34 413,08**	**33 653,32**	**5 458,01**	**87,75**	**4,67**	**54,00**	**729,79**	**1 961,87**	**171,68**	**7 832,81**	**79 325,18**

Le total général est de: Quatorze million neuf cent cinquante-six mille deux cent quarante-six dollars, vingt-sept centimes

Fait à Lubumbashi, le 05/04/2019

Pierrot MALOBA KITUMBA

Légende:

Carbonate de cobalt: (Carb.Co);Cathode de cobalt (Cath.co) : (Cath.Cu); Cathode de cuivre: (Cath.Cu); Cobalt Electrode (Co_ELECTRO);
Concentré de Manganèse: (Conc.Mn); concentré de cobalt : (Conc.co); Concentré de cuivre: (Conc.cu); concentré cupro-cobaltifère: (Conc.CuCo);
Concentré d'Etain (Conc.ETAIN); Concentré d'Etain (Conc.TANTALE); Cuivre Blister(CU_BLST); Cuivre noir(CU.NOIR); Cuivre Pulverisé: (Cu.Pul); Hydroxyde de Cobalt: (Hydro.Co);
Matte de cuivre: (Matt.Cu); Minerais de cuivre: (Min.Cu); Minerais de cobalt: (Min.Co); Craps de cuivre(craps.Cu)

République Démocratique du Congo

DIVISION PROVINCIALE DES MINES
DU HAUT-KATANGA
LUBUMBASHI
Le Chef de Division

STATISTIQUES DE LA REDEVANCE MINIERE PAR QUOTITE EMISES POUR LA PERIODE DE JANVIER A MARS 2019 (HAUT-KATANGA)

N°	SOCIETE	REDEVANCE 100%	TERSOR 50%	PROVINCE 25%	TERRITOIRE 15%	FONDS MINIERS 10%	QUANTITES DES PRODUITS EXPORTES EN TONNES													
							CATH_CU	CO_SEPAR	CONC_CO	CONC_CU	CONC_CUCO	CONC_ETAIN	CONC_TANTALE	CU_AUTRE	CU_BLIST	CU_DEC	CU_NOIR	CU_PULV	HYDRO_CO	Min_C
1	ANVIL MINING	405 621,21	202 810,61	101 405,30	60 843,18	40 562,12				10256										
2	CDM	9 160 330,02	4 580 165,01	2 290 082,50	1 374 049,50	916 033,00	11138,33				8677,391				1489,967				7965,811	
3	CHEMAF	8 915 569,97	4 457 784,99	2 228 892,49	1 337 335,50	891 557,00	8004					75							11337,521	
4	CNMC CONGO COMPAGNIE	787 079,24	393 539,62	196 769,81	118 061,89	78 707,92	3676,148													
5	CNMC HUACHIN MABENDE	2 255 443,97	1 127 721,98	563 860,99	338 316,60	225 544,40	10493,239													
6	COMIKA	6 522,77	3 261,38	1 630,69	978,42	652,28					103,536									
7	COMILU	1 484 572,58	742 286,29	371 143,15	222 685,89	148 457,26	6931,545													
8	CONGO JIN JU	200 195,38	100 097,69	50 048,85	30 029,31	20 019,54	933,394													
9	FRONTIER	1 701 169,70	850 584,85	425 292,43	255 175,46	170 116,97				36782										
10	GCM	917 678,32	458 839,16	229 419,58	137 651,75	91 767,83	2490,966	120								137,231		254,664		
11	GOLDEN AFRICAN	337 765,40	168 882,70	84 441,35	50 664,81	33 776,54	1570													
12	HUACHIN METAL LEACH	673 090,06	336 545,03	168 272,51	100 963,51	67 309,01	3133,962													
13	KAIPENG	1 561 477,29	780 738,64	390 369,32	234 221,59	156 147,73	7084,665													
14	KICC	1 636 589,87	818 294,93	409 147,47	245 488,48	163 658,99				18907,606										
15	LONG FEI(VENTE LOCALE)	93 843,61	46 921,81	23 460,90	14 076,54	9 384,36														
16	LUALABA MINING(VENTE LOCALE)	92 513,21	46 256,60	23 128,30	13 876,98	9 251,32														5225,
17	METAL MINES	2 491 972,32	1 245 986,16	622 993,08	373 795,85	249 197,23	2581,5		158,763										2872,5001	
18	MIKAS	2 403 399,53	1 201 699,77	600 849,88	360 509,93	240 339,95	1057,902												2839,57	
19	MJM	1 513 052,91	756 526,46	378 263,23	226 957,94	151 305,29	1889												1497,4766	
20	MMG	2 964 946,55	1 482 473,28	741 236,64	444 741,98	296 494,66	13815,03													
20	MMG(VENTE LOCALE)	7 005,78	3 502,89	1 751,44	1 050,87	700,58	32,909													
21	OM METAL	145 901,70	72 950,85	36 475,43	21 885,26	14 590,17	360							330						
22	RUASHI MINING	2 646 127,88	1 323 063,94	661 531,97	396 919,18	264 612,79	8560,141												1196,6946	
23	RUBAMIN	1 014 738,18	507 369,09	253 684,55	152 210,73	101 473,82											6568,005			
23	RUBAMIN(VENTE LOCALE)	111 814,88	55 907,44	27 953,72	16 772,23	11 181,49														
24	SEK	515 147,73	257 573,87	128 786,93	77 272,16	51 514,77	2369,649													
25	SHITURU MINING	1 853 626,79	926 813,40	463 406,70	278 044,02	185 362,68	8582,598													
26	SINO KATANGA	26 152,38	13 076,19	6 538,10	3 922,86	2 615,24						15,42	4,67							
27	SOMIKA	2 286 729,20	1 143 364,60	571 682,30	343 009,38	228 672,92	5220												1443,81	
28	TSM	14 878,07	7 439,03	3 719,52	2 231,71	1 487,81						47,332								
	TOTAL	**48 224 956,50**	**24 112 478,25**	**12 056 239,13**	**7 233 743,48**	**4 822 495,65**	**99 924,98**	**120,00**	**158,76**	**65 945,61**	**8 780,93**	**137,75**	**4,67**	**330,00**	**1 489,97**	**137,23**	**6 568,01**	**254,66**	**29 153,38**	**5 225,**

Le total général est de: Quarante-huit million deux cent vingt-quatre mille neuf cent cinquante-six dollars, cinquante centimes

Fait à Lubumbashi, le 10/04/2019

Légende:

Carbonate de cobalt: (Carb.Co);Cathode de cobalt (Cath.co) : (Cath.Cu); Cathode de cuivre: (Cath.Cu); Cobalt Electrode (Co_ELECTRO);
Concentré de Manganèse: (Conc.Mn); concentré de cobalt : (Conc.co); Concentré de cuivre: (Conc.cu); concentré cupro-cobaltifère: (Conc.CuCo);
Concentré d'Etain (Conc.ETAIN); Concentré d'Etain (Conc.TANTALE); Cuvre Blister(CU_BLST); Cuivre noir(CU.NOIR); Cuivre Pulverisé: (Cu.Pul); Hydroxyde de Cobalt: (Hydro.Co);
Matte de cuivre: (Matt.Cu); Minerais de cuivre: (Min.Cu); Minerais de cobalt: (Min.Co); Craps de cuivre(craps.Cu)

République Démocratique du Congo

DIVISION PROVINCIALE DES MINES
DU HAUT-KATANGA
LUBUMBASHI

Le Chef de Division

STATISTIQUES DE LA REDEVANCE MINIERE PAR QUOTITE EMISES POUR LA PERIODE DE MAI 2019 (HAUT-KATANGA)

							QUANTITES DES PRODUITS EXPORTES EN TONNES													
N°	SOCIETE	REDEVANCE 100%	TERSOR 50%	PROVINCE 25%	TERRITOIRE 15%	FONDS MINIERS 10%	Ano_Sol	Carb_Co	CATH_CU	CO_SEPAR	CONC_CU	CONC_CUCO	CONC_ETAIN	CU_AUTRE	CU_BLIST	CU_NOIR	CU_ROUGE	Déch_Ano	HYDRO_CO	Min_Cu
1	CDM	2 727 122,31	1 363 561,16	681 780,58	409 068,35	272 712,23			4199,915			7527,406			967,566				691,751	
2	CHEMAF	1 160 710,57	580 355,28	290 177,64	174 106,59	116 071,06			2249				15						1188,586	
3	CNMC CONGO COMPAGNIE	484 020,27	242 010,14	121 005,07	72 603,04	48 402,03			2145,5											
4	CNMC HUACHIN MABENDE	701 359,21	350 679,61	175 339,80	105 203,88	70 135,92			3108,938											
5	COMIKA	1 043 175,26	521 587,63	260 793,82	156 476,29	104 317,53						6452,6052								
6	COMILU	350 999,94	175 499,97	87 749,98	52 649,99	35 099,99			1554,817											
7	CONGO JIN JU	81 031,76	40 515,88	20 257,94	12 154,76	8 103,18			359,251											
8	FRONTIER	1 367 304,35	683 652,18	341 826,09	205 095,65	136 730,44					27699									
9	GCM	528 780,34	264 390,17	132 195,08	79 317,05	52 878,03	59,978	30	1276,747	110							141,015	90,076		
10	GOLDEN AFRICAN	145 710,15	72 855,07	36 427,54	21 856,52	14 571,01			646											
11	HUACHIN METAL LEACH	490 883,49	245 441,75	122 720,87	73 632,52	49 088,35			2176,312											
12	KAIPENG	614 562,06	307 281,03	153 640,51	92 184,31	61 456,21			2724,238											
13	KASTRO SARL	35 483,37	17 741,69	8 870,84	5 322,51	3 548,34													65	
14	KICC	573 761,70	286 880,85	143 440,42	86 064,25	57 376,17					6671,58									
15	LONG FEI(VENTE LOCALE)	55 613,37	27 806,69	13 903,34	8 342,01	5 561,34														12317,5
16	LUALABA MINING(VENTE LOCALE)	32 673,94	16 336,97	8 168,49	4 901,09	3 267,39														19283,783
17	METAL MINES	765 474,49	382 737,25	191 368,62	114 821,17	76 547,45			1123,5										938,0118	
18	MIKAS	574 577,77	287 288,89	143 644,44	86 186,67	57 457,78													1054,25	
19	MJM	151 349,08	75 674,54	37 837,27	22 702,36	15 134,91			671											
20	MMG	1 219 458,13	609 729,06	304 864,53	182 918,72	121 945,81			5406,418											
21	OM METAL	109 494,00	54 747,00	27 373,50	16 424,10	10 949,40			420					54						
22	RUASHI MINING	1 871 520,44	935 760,22	467 880,11	280 728,07	187 152,04			3586,159										1951,079	
23	RUBAMIN	384 174,54	192 087,27	96 043,64	57 626,18	38 417,45										2364,763				
23	RUBAMIN(VENTE LOCALE)	39 546,63	19 773,31	9 886,66	5 931,99	3 954,66														20810,376
24	SEK	183 435,09	91 717,54	45 858,77	27 515,26	18 343,51			813,252											
25	SEMHKAT(VENTE LOCALE)	4 515,00	2 257,50	1 128,75	677,25	451,50														1000
26	SHITURU MINING	708 994,70	354 497,35	177 248,68	106 349,21	70 899,47			3142,763											
27	SOMIKA	955 388,83	477 694,42	238 847,21	143 308,32	95 538,88			2678										643,46	
28	TSM	7 123,61	3 561,80	1 780,90	1 068,54	712,36							23,305							
	TOTAL	17 368 244,41	8 684 122,21	4 342 061,10	2 605 236,66	1 736 824,44	59,98	30,00	38 281,81	110,00	34 370,58	13 980,01	38,31	54,00	967,57	2 364,76	141,02	90,08	6 532,14	53 411,66

Le total général est de: Dix-sept million trois cent soixante-huit mille deux cent quarante-quatre dollars, quarante-un centimes

Fait à Lubumbashi, le 05/06/2019

Pierrot MALOBA KITUMBA

Légende:

Carbonate de cobalt: (Carb.Co);Cathode de cobalt (Cath.co) :Cobalt Electrode (Co_ELECTRO);Cuivre de Décuivrage (Cu.DEC); Cathode de cuivre: (Cath.Cu); Concentré de Manganèse: (Conc.Mn); concentré de cobalt : (Conc.co); Concentré de cuivre: (Conc.cu); concentré cupro-cobaltifère: (Conc.CuCo); Concentré d'Etain (Conc.ETAIN); Concentré d'Etain (Conc.TANTALE); Cuvre Blister(CU_BLST); Cuivre noir(CU.NOIR); Cuivre Pulverisé: (Cu.Pulv); Hydroxyde de Cobalt: (Hydro.Co); Cobalt separateur Magnétique(Co.SEPAR);Matte de cuivre: (Matt.Cu); Minerais de cuivre: (Min.Cu); Minerais de cobalt: (Min.Co); Craps de cuivre(craps.Cu); Cuivre Autrement présenté(Cu.AUTRE); Concentré de Cuivre et Argent (CONC.CU.Arg); Déchet d'Anodes(Déch.Ano); Anodes Solides(Ano.Sol)

République Démocratique du Congo

DIVISION PROVINCIALE DES MINES
DU HAUT-KATANGA
LUBUMBASHI

Le Chef de Division

STATISTIQUES DE LA REDEVANCE MINIERE PAR QUOTITE EMISES POUR LA PERIODE DE JUIN 2019 (HAUT-KATANGA)

							QUANTITES DES PRODUITS EXPORTES EN TONNES								
N°	SOCIETE	REDEVANCE 100%	TERSOR 50%	PROVINCE 25%	TERRITOIRE 15%	FONDS MINIERS 10%	CATH_CU	CONC_CU	CONC_CU_Arg	CONC_CUCO	CONC_ETAIN	CU_BLIST	CU_NOIR	HYDRO_CO	Min_Cu
1	AMICAL KAKANA MINING	726,25	363,13	181,56	108,94	72,63		25							
2	ANVIL MINING	665 485,37	332 742,69	166 371,34	99 822,81	66 548,54		10256	9148,352						
3	CDM	1 964 650,74	982 325,37	491 162,69	294 697,61	196 465,07	3460,437			3642,062		355,221		1029,391	
4	CHEMAF	899 287,20	449 643,60	224 821,80	134 893,08	89 928,72	679				35			1340,722	
5	CNMC CONGO COMPAGNIE	208 476,68	104 238,34	52 119,17	31 271,50	20 847,67	988,184								
6	CNMC HUACHIN MABENDE	407 312,28	203 656,14	101 828,07	61 096,84	40 731,23	1930,669								
7	COMIKA	1 525 823,42	762 911,71	381 455,86	228 873,51	152 582,34				9382,377					
8	COMILU	159 225,97	79 612,99	39 806,49	23 883,90	15 922,60	754,697								
9	CONGO JIN JU	55 157,76	27 578,88	13 789,44	8 273,66	5 515,78	261,449								
10	FRONTIER	7 609 652,93	3 804 826,47	1 902 413,23	1 141 447,94	760 965,29		166339							
11	GCM	207 269,94	103 634,97	51 817,48	31 090,49	20 726,99	982,464								
12	GOLDEN AFRICAN	101 270,40	50 635,20	25 317,60	15 190,56	10 127,04	480								
13	HUACHIN METAL LEACH	324 912,01	162 456,01	81 228,00	48 736,80	32 491,20	1540,09								
14	KAIPENG	455 344,33	227 672,16	113 836,08	68 301,65	45 534,43	2143,358								
15	KICC	458 964,07	229 482,03	114 741,02	68 844,61	45 896,41		5575,393							
16	LONG FEI(VENTE LOCALE)	52 257,22	26 128,61	13 064,30	7 838,58	5 225,72									12385,429
17	LUALABA MINING(VENTE LOCALE)	31 153,03	15 576,52	7 788,26	4 672,95	3 115,30									22272,161
18	METAL MINES	683 565,68	341 782,84	170 891,42	102 534,85	68 356,57	617							994,659	
19	MIKAS	107 550,77	53 775,39	26 887,69	16 132,62	10 755,08	509,793								
20	MJM	78 691,62	39 345,81	19 672,91	11 803,74	7 869,16	373								
21	MMG	1 270 398,62	635 199,31	317 599,66	190 559,79	127 039,86	6021,717								
22	OM METAL	59 640,00	29 820,00	14 910,00	8 946,00	5 964,00	270								
23	RUASHI MINING	577 762,58	288 881,29	144 440,64	86 664,39	57 776,26	2303,016							165,16	
24	RUBAMIN	247 765,06	123 882,53	61 941,26	37 164,76	24 776,51							1611,341		
24	RUBAMIN(VENTE LOCALE)	34 177,75	17 088,88	8 544,44	5 126,66	3 417,78									15302,048
25	SEK	115 597,99	57 799,00	28 899,50	17 339,70	11 559,80	547,937								
26	SEMHKAT(VENTE LOCALE)	8 470,00	4 235,00	2 117,50	1 270,50	847,00									2000
27	SHITURU MINING	620 296,45	310 148,22	155 074,11	93 044,47	62 029,64	2906,607								
28	SOMIKA	323 649,23	161 824,61	80 912,31	48 547,38	32 364,92	1534								
28	SOMIKA(VENTE LOCALE)	108 535,55	54 267,77	27 133,89	16 280,33	10 853,55									25724,05
	TOTAL	19 363 070,93	9 681 535,46	4 840 767,73	2 904 460,64	1 936 307,09	28 303,42	182 195,39	9 148,35	13 024,44	35,00	355,22	1 611,34	3 529,93	77 683,69

Le total général est de: Dix-neuf million trois cent soixante-trois mille septante dollars, nonante-trois centimes

Légende:
Carbonate de cobalt: (Carb.Co);Cathode de cobalt (Cath.co) :Cobalt Electrode (Co_ELECTRO);Cuivre de Décuivrage (Cu.DEC); Cathode de cuivre: (Cath.Cu); Concentré de Manganèse: (Conc.Mn); concentré de cobalt : (Conc.co); Concentré de cuivre: (Conc.cu); concentré cupro-cobaltifère: (Conc.CuCo);
Concentré d'Etain (Conc.ETAIN); Concentré d'Etain (Conc.TANTALE); Dechet de Cuivre(Dechet_Cu);Cuivre Blister(CU_BLST); Cuivre noir(CU.NOIR); Cuivre Pulverisé: (Cu.Pulv); Hydroxyde de Cobalt: (Hydro.Co);
Cobalt separateur Magnétique(Co.SEPAR);Matte de cuivre: (Matt.Cu); Minerais de cuivre: (Min.Cu); Minerais de cobalt: (Min.Co); Craps de cuivre(craps.Cu); Cuivre Autrement présenté(Cu.AUTRE); Concentré de Cuivre et Argent (CONC.CU.Arg); Déchet d'Anodes(Déch.Ano); Anodes Solides(Ano.Sol)

République Démocratique du Congo

DIVISION PROVINCIALE DES MINES
DU HAUT-KATANGA
LUBUMBASHI

Le Chef de Division

STATISTIQUES DE LA REDEVANCE MINIERE PAR QUOTITE EMISES
POUR LA PERIODE D'AVRIL 2019
(HAUT-KATANGA)

							QUANTITES DES PRODUITS EXPORTES EN TONNES											
N°	SOCIETE	REDEVANCE 100%	TERSOR 50%	PROVINCE 25%	TERRITOIRE 15%	FONDS MINIERS 10%	CATH_CU	CONC_CU	CONC_CU_Arg	CONC_CUCO	CONC_ETAIN	CU_AUTRE	CU_BLIST	CU_DEC	CU_NOIR	CU_PULV	HYDRO_CO	Min_Cu
1	ANVIL MINING	721671,3685	360 835,68	180 417,84	108 250,71	72 167,14			18231,066									
2	CDM	2281821,763	1 140 910,88	570 455,44	342 273,26	228 182,18	4354,955			4920,669			657,624				833,522	
3	CHEMAF	1376062,787	688 031,39	344 015,70	206 409,42	137 606,28	819				35						2353,6	
4	CNMC CONGO COMPAGNIE	138175,8057	69 087,90	34 543,95	20 726,37	13 817,58	617,4											
5	CNMC HUACHIN MABENDE	421119,1636	210 559,58	105 279,79	63 167,87	42 111,92	1865,333											
6	COMIKA	259070,8239	129 535,41	64 767,71	38 860,62	25 907,08				1718,704								
7	COMILU	376189,4759	188 094,74	94 047,37	56 428,42	37 618,95	1663,819											
8	CONGO JIN JU	54058,88705	27 029,44	13 514,72	8 108,83	5 405,89	239,452											
9	FRONTIER	1376485,186	688 242,59	344 121,30	206 472,78	137 648,52		27699										
10	GCM	350783,5966	175 391,80	87 695,90	52 617,54	35 078,36	1497,853							27,877		28,054		
11	GOLDEN AFRICAN	130038,2496	65 019,12	32 509,56	19 505,74	13 003,82	576											
12	HUACHIN METAL LEACH	501508,7644	250 754,38	125 377,19	75 226,31	50 150,88	2221,416											
13	KAIPENG	617873,8467	308 936,92	154 468,46	92 681,08	61 787,38	2733,038											
14	KICC	462548,6319	231 274,32	115 637,16	69 382,29	46 254,86		4941,547										
15	LONG FEI(VENTE LOCALE)	12695,3673	6 347,68	3 173,84	1 904,31	1 269,54												2811,82
16	LUALABA MINING(VENTE LOCALE)	12513,0488	6 256,52	3 128,26	1 876,96	1 251,30												7390,505
17	METAL MINES	936437,9741	468 218,99	234 109,49	140 465,70	93 643,80	976										1425,1875	
18	MIKAS	423604,2709	211 802,14	105 901,07	63 540,64	42 360,43											844,86	
19	MJM	155784,405	77 892,20	38 946,10	23 367,66	15 578,44	690											
20	MMG	909945,1005	454 972,55	227 486,28	136 491,77	90 994,51	4038,956											
21	OM METAL	18711	9 355,50	4 677,75	2 806,65	1 871,10	54					27						
22	RUASHI MINING	995308,717	497 654,36	248 827,18	149 296,31	99 530,87	2225,869										945,543	
23	RUBAMIN	281678,4829	140 839,24	70 419,62	42 251,77	28 167,85									1730,215			
23	RUBAMIN(VENTE LOCALE)	38796,2057	19 398,10	9 699,05	5 819,43	3 879,62												29525,413
24	SEK	137995,9498	68 997,97	34 498,99	20 699,39	13 799,59	612,004											
25	SHITURU MINING	645958,4578	322 979,23	161 489,61	96 893,77	64 595,85	2861,251											
26	SOMIKA	326001,0124	163 000,51	81 500,25	48 900,15	32 600,10	1444											
	TOTAL	13 962 838,34	6 981 419,17	3 490 709,59	2 094 425,75	1 396 283,83	29 490,35	32 640,55	18 231,07	6 639,37	35,00	27,00	657,62	27,88	1 730,22	28,05	6 402,71	39 727,74

Le total général est de: Treize million neuf cent soixante deux mille huit cent trente-huit dollars, trente-quatre centimes

Fait à Lubumbashi, le 06/05/2019

Pierrot MALOBA KITUMBA

Légende:

Carbonate de cobalt: (Carb.Co);Cathode de cobalt (Cath.co) :Cobalt Electrode (Co_ELECTRO);Cuivre de Décuivrage (Cu.DEC); Cathode de cuivre: (Cath.Cu); Concentré de Manganèse: (Conc.Mn);
concentré de cobalt : (Conc.co); Concentré de cuivre: (Conc.cu); concentré cupro-cobaltifère: (Conc.CuCo);
Concentré d'Etain (Conc.ETAIN); Concentré d'Etain (Conc.TANTALE); Cuvre Blister(CU_BLST); Cuivre noir(CU.NOIR); Cuivre Pulverisé: (Cu.Pulv); Hydroxyde de Cobalt: (Hydro.Co);
Cobalt separateur Magnétique(Co.SEPAR);Matte de cuivre: (Matt.Cu); Minerais de cuivre: (Min.Cu); Minerais de cobalt: (Min.Co); Craps de cuivre(craps.Cu); Cuivre Autrement présenté(Cu.AUTRE);
Concentré de Cuivre et Argent (CONC.CU.Arg); Déchet d'Anodes(Déch.Ano); Anodes Solides(Ano.Sol)

République Démocratique du Congo

DIVISION PROVINCIALE DES MINES
DU HAUT-KATANGA
LUBUMBASHI
Le Chef de Division

STATISTIQUES DE LA REDEVANCE MINIERE PAR QUOTITE EMISES POUR LA PERIODE DE JUILLET 2019 (HAUT-KATANGA)

							QUANTITES DES PRODUITS EXPORTES EN TONNES									
N°	SOCIETE	REDEVANCE 100%	TERSOR 50%	PROVINCE 25%	TERRITOIRE 15%	FONDS MINIERS 10%	ALL,BLANC	CATH_CU	CONC_CU	CONC_CUCO	CONC_ETAIN	CU_BLIST	CU_NOIR	Dechet_Cu	HYDRO_CO	Min_Cu
1	AMICAL KAKANA MINING	706,13	353,06	176,53	105,92	70,61			25							
2	CDM	1 871 655,53	935 827,76	467 913,88	280 748,33	187 165,55		3187,998		4005,147		1102,085			932,873	
3	CHEMAF	1 212 899,18	606 449,59	303 224,80	181 934,88	121 289,92		1574			15				1942,08	
4	CNMC CONGO COMPAGNIE	248 715,75	124 357,87	62 178,94	37 307,36	24 871,57		1197,206								
5	CNMC HUACHIN MABENDE	612 256,56	306 128,28	153 064,14	91 838,48	61 225,66		2957,081								
6	COMIKA	1 056 842,77	528 421,39	264 210,69	158 526,42	105 684,28		527,094		5198,381					517,759	
7	COMILU	323 239,61	161 619,81	80 809,90	48 485,94	32 323,96		1565,325								
8	CONGO JIN JU	82 462,82	41 231,41	20 615,70	12 369,42	8 246,28		401,514								
9	FRONTIER	832 643,38	416 321,69	208 160,85	124 896,51	83 264,34			18428							
10	GCM	313 851,77	156 925,89	78 462,94	47 077,77	31 385,18		1528,162								
11	GOLDEN AFRICAN	171 682,56	85 841,28	42 920,64	25 752,38	17 168,26		832								
12	HUACHIN METAL LEACH	582 379,22	291 189,61	145 594,80	87 356,88	58 237,92		2830,5								
13	KAIPENG	552 677,89	276 338,95	138 169,47	82 901,68	55 267,79		2649,225								
14	KASTRO SARL	54 245,98	27 122,99	13 561,49	8 136,90	5 424,60									97,5	
15	KICC	384 498,52	192 249,26	96 124,63	57 674,78	38 449,85			4512,66							
16	LONG FEI(VENTE LOCALE)	41 263,80	20 631,90	10 315,95	6 189,57	4 126,38										10042,298
17	LUALABA MINING(VENTE LOCALE)	18 319,19	9 159,60	4 579,80	2 747,88	1 831,92										14706,474
18	METAL MINES	671 225,20	335 612,60	167 806,30	100 683,78	67 122,52		933							1052,004	
19	MIKAS	255 939,32	127 969,66	63 984,83	38 390,90	25 593,93		1246,183								
20	MJM	195 480,00	97 740,00	48 870,00	29 322,00	19 548,00		951,8								
21	MMG	1 070 296,33	535 148,16	267 574,08	160 544,45	107 029,63		5209,306								
22	MMG(VENTE LOCALE)	5 610,12	2 805,06	1 402,53	841,52	561,01								27,316		
23	OM METAL	77 280,00	38 640,00	19 320,00	11 592,00	7 728,00		360								
24	RUASHI MINING	2 128 216,17	1 064 108,09	532 054,04	319 232,43	212 821,62		3002,248							2953,42	
25	RUBAMIN	348 391,25	174 195,63	87 097,81	52 258,69	34 839,13	107,44						2210,842			
25	RUBAMIN(VENTE LOCALE)	92 188,13	46 094,06	23 047,03	13 828,22	9 218,81										54528,435
26	SEK	157 881,82	78 940,91	39 470,46	23 682,27	15 788,18		767,32								
27	SHITURU MINING	820 776,79	410 388,40	205 194,20	123 116,52	82 077,68		3980,75								
28	SOMIKA	501 085,03	250 542,51	125 271,26	75 162,75	50 108,50		2436								
29	TSM	6 721,85	3 360,92	1 680,46	1 008,28	672,18					23,719					
	TOTAL	14 691 432,68	7 345 716,34	3 672 858,17	2 203 714,90	1 469 143,27	107,44	38 136,71	22 965,66	9 203,53	38,72	1 102,09	2 210,84	27,32	7 495,64	79 277,21

Le total général est de: Quatorze million six cent nonante-un mille quatre cent trente-deux dollars, soixante-huit centimes

Légende:

Carbonate de cobalt: (Carb.Co);Cathode de cobalt (Cath.co) :Cobalt Electrode (Co_ELECTRO);Cuivre de Décuivrage (Cu.DEC); Cathode de cuivre: (Cath.Cu); Concentré de Manganèse: (Conc.Mn); concentré de cobalt : (Conc.co); Concentré de cuivre: (Conc.cu); concentré cupro-cobaltifère: (Conc.CuCo);
Concentré d'Etain (Conc.ETAIN); Concentré d'Etain (Conc.TANTALE); Dechet de Cuivre(Dechet_Cu);Cuivre Blister(CU_BLST); Cuivre noir(CU.NOIR); Cuivre Pulverisé: (Cu.Pulv); Hydroxyde de Cobalt: (Hydro.Co);
Cobalt separateur Magnétique(Co.SEPAR);Matte de cuivre: (Matt.Cu); Minerais de cuivre: (Min.Cu); Minerais de cobalt: (Min.Co); Craps de cuivre(craps.Cu); Cuivre Autrement présenté(Cu.AUTRE); Concentré de Cuivre et Argent (CONC.CU.Arg);

République Démocratique du Congo

DIVISION PROVINCIALE DES MINES
DU HAUT-KATANGA
LUBUMBASHI
Le Chef de Division

STATISTIQUES DE LA REDEVANCE MINIERE PAR QUOTITE EMISES POUR LA PERIODE D'AOÛT 2019 (HAUT-KATANGA)

N°	SOCIETE	REDEVANCE 100%	TERSOR 50%	PROVINCE 25%	TERRITOIRE 15%	FONDS MINIERS 10%	QUANTITES DES PRODUITS EXPORTES EN TONNES								
							CATH_CU	CONC_CU	CONC_CUCO	CU_BLIST	CU_NOIR	Dechet_Cu	HYDRO_CO	Min_Co	Min_Cu
1	ANVIL MINING	383 790,34	191 895,17	95 947,59	57 568,55	38 379,03		10256							
2	CDM	1 542 060,31	771 030,16	385 515,08	231 309,05	154 206,03	3443,898		4154,425	492,647			380,92		
3	CHEMAF	824 176,74	412 088,37	206 044,18	123 626,51	82 417,67	1872						975,351		
4	CNMC CONGO COMPAGNIE	307 432,05	153 716,03	76 858,01	46 114,81	30 743,21	1478,787								
5	CNMC HUACHIN MABENDE	750 760,54	375 380,27	187 690,13	112 614,08	75 076,05	3611,253								
6	COMIKA	358 846,45	179 423,23	89 711,61	53 826,97	35 884,65	649,322						501,929		
7	COMILU	594 221,82	297 110,91	148 555,46	89 133,27	59 422,18	2858,282								
8	CONGO JIN JU	47 532,80	23 766,40	11 883,20	7 129,92	4 753,28	228,633								
9	FRONTIER	1 258 797,17	629 398,59	314 699,29	188 819,58	125 879,72		28397,52							
10	GCM	253 274,07	126 637,03	63 318,52	37 991,11	25 327,41	1221,146								
11	GOLDEN AFRICAN	144 698,40	72 349,20	36 174,60	21 704,76	14 469,84	696								
12	HUACHIN METAL LEACH	299 849,09	149 924,55	74 962,27	44 977,36	29 984,91	1442,312								
13	KAIPENG	601 031,29	300 515,65	150 257,82	90 154,69	60 103,13	2886,329								
14	KICC	287 105,05	143 552,53	71 776,26	43 065,76	28 710,51		3444,243							
15	LONG FEI(VENTE LOCALE)	32 703,89	16 351,94	8 175,97	4 905,58	3 270,39									7865,491
16	LUALABA MINING(VENTE LOCALE)	16 371,39	8 185,69	4 092,85	2 455,71	1 637,14									12881,514
17	METAL MINES	689 372,62	344 686,31	172 343,16	103 405,89	68 937,26	999,5						1079,768		
18	MIKAS	340 193,67	170 096,84	85 048,42	51 029,05	34 019,37	1046,167						275,12		
19	MJM	137 214,00	68 607,00	34 303,50	20 582,10	13 721,40	660								
20	MMG	1 152 484,83	576 242,41	288 121,21	172 872,72	115 248,48	5547,731								
20	MMG(VENTE LOCALE)	11 205,35	5 602,67	2 801,34	1 680,80	1 120,53						54,347			
21	OM METAL	76 440,00	38 220,00	19 110,00	11 466,00	7 644,00	360								
22	RUASHI MINING	1 948 604,85	974 302,43	487 151,21	292 290,73	194 860,49	2949,029						2694,08		
22	RUASHI MINING(VENTE LOCALE)	1 696,42	848,21	424,11	254,46	169,64	8,16								
23	RUBAMIN	456 087,27	228 043,64	114 021,82	68 413,09	45 608,73					3047,01				
23	RUBAMIN(VENTE LOCALE)	45 013,19	22 506,60	11 253,30	6 751,98	4 501,32									20481,444
24	SEK	141 633,70	70 816,85	35 408,43	21 245,06	14 163,37	681,276								
24	SEK(VENTE LOCALE)	10 668,41	5 334,21	2 667,10	1 600,26	1 066,84						68,422			
25	SEMHKAT(VENTE LOCALE)	12 474,00	6 237,00	3 118,50	1 871,10	1 247,40									3000
26	SHAMITUMBA (VENTE LOCAL)	287 893,03	143 946,52	71 973,26	43 183,95	28 789,30								9524,76	95592,95
27	SHITURU MINING	612 889,66	306 444,83	153 222,42	91 933,45	61 288,97	2956,913								
28	SOMIKA	353 453,80	176 726,90	88 363,45	53 018,07	35 345,38	1700								
	TOTAL	13 979 976,22	6 989 988,11	3 494 994,06	2 096 996,43	1 397 997,62	37 296,74	42 097,76	4 154,43	492,65	3 047,01	122,77	5 907,17	9 524,76	139 821,40

Le total général est de: Treize million neuf cent septante-neuf mille neuf cent septante-six dollars, Vingt-deux centimes

Légende:

Carbonate de cobalt: (Carb.Co);Cathode de cobalt (Cath.co) :Cobalt Electrode (Co_ELECTRO);Cuivre de Décuivrage (Cu.DEC);
Cathode de cuivre: (Cath.Cu); Concentré de Manganèse: (Conc.Mn); concentré de cobalt : (Conc.co); Concentré de cuivre: (Conc.cu); concentré cupro-cobaltifère: (Conc.CuCo);
Concentré d'Etain (Conc.ETAIN); Concentré d'Etain (Conc.TANTALE); Dechet de Cuivre(Dechet_Cu);Cuivre Blister(CU_BLST);
Cuivre noir(CU.NOIR); Cuivre Pulverisé: (Cu.Pulv); Hydroxyde de Cobalt: (Hydro.Co);
Cobalt separateur Magnétique(Co.SEPAR);Matte de cuivre: (Matt.Cu); Minerais de cuivre: (Min.Cu); Minerais de cobalt: (Min.Co); Craps de cuivre(craps.Cu); Cuivre Autrement présenté(Cu.AUTRE); Concentré de Cuivre et Argent (CONC.CU.Arg);

République Démocratique du Congo

DIVISION PROVINCIALE DES MINES
DU HAUT-KATANGA
LUBUMBASHI
Le Chef de Division

STATISTIQUES DE LA REDEVANCE MINIERE PAR QUOTITE EMISES POUR LA PERIODE SEPTEMBRE 2019 (HAUT-KATANGA)

N°	SOCIETE	REDEVANCE 100%	TERSOR 50%	PROVINCE 25%	TERRITOIRE 15%	FONDS MINIERS 10%	QUANTITES DES PRODUITS EXPORTES EN TONNES											
							CALCINE_ZINC	CATH_CU	CONC_CU	CONC_CU_Arg	CONC_CUCO	CONC_ETAIN	CU_AUTRE	CU_BLIST	CU_NOIR	HYDRO_CO	Min_Cu	SULFATE DE PLOMB
1	AMICAL KAKANA MINING	714,88	357,44	178,72	107,23	71,49												
2	ANVIL MINING	325 781,25	162 890,62	81 445,31	48 867,19	32 578,12			25									
3	CDM	1 637 496,77	818 748,38	409 374,19	245 624,52	163 749,68				9060,15								
4	CHEMAF	1 778 292,19	889 146,09	444 573,05	266 743,83	177 829,22		2967,196			5290,01			52,9		592,195		
5	CNMC CONGO COMPAGNIE	287 319,11	143 659,56	71 829,78	43 097,87	28 731,91		2990				10				2458,113		
6	CNMC HUACHIN MABENDE	464 287,89	232 143,94	116 071,97	69 643,18	46 428,79		1427,593										
7	COMIKA	358 458,53	179 229,27	89 614,63	53 768,78	35 845,85		2309,582										
8	COMILU	645 631,06	322 815,53	161 407,77	96 844,66	64 563,11		592,352								500,838		
9	CONGO JIN JU	64 350,14	32 175,07	16 087,53	9 652,52	6 435,01		3231,965										
10	FRONTIER	1 206 726,56	603 363,28	301 681,64	181 008,98	120 672,66		322,105										
11	GCM	486 286,42	243 143,21	121 571,60	72 942,96	48 628,64	466,44	2079,139	27741									
12	GOLDEN AFRICAN	186 979,34	93 489,67	46 744,83	28 046,90	18 697,93		936					234,104					278,38
13	HUACHIN METAL LEACH	481 163,83	240 581,91	120 290,96	72 174,57	48 116,38		2394,6										
14	KAIPENG	721 836,08	360 918,04	180 459,02	108 275,41	72 183,61		3531,989										
15	KICC	19 482,52	9 741,26	4 870,63	2 922,38	1 948,25			239,36									
16	LONG FEI(VENTE LOCALE)	6 831,54	3 415,77	1 707,88	1 024,73	683,15												
17	LUALABA MINING(VENTE LOCALE)	19 960,00	9 980,00	4 990,00	2 994,00	1 996,00											1709,9<br>14372,735	
18	METAL MINES	733 336,12	366 668,06	183 334,03	110 000,42	73 333,61		805										
19	MIKAS	307 077,19	153 538,60	76 769,30	46 061,58	30 707,72		1188,934								1193,9796		
20	MJM	123 064,48	61 532,24	30 766,12	18 459,67	12 306,45		616								142,73		
21	MMG	1 527 314,15	763 657,08	381 828,54	229 097,12	152 731,42		7640,028										
21	MMG(VENTE LOCALE)	15 172,29	7 586,15	3 793,07	2 275,84	1 517,23		75,951										
22	OM METAL	68 040,00	34 020,00	17 010,00	10 206,00	6 804,00		330										
23	RUASHI MINING	4 041 099,49	2 020 549,75	1 010 274,87	606 164,92	404 109,95		2607,46										
24	RUBAMIN	365 263,57	182 631,79	91 315,89	54 789,54	36 526,36										5879,34		
24	RUBAMIN(VENTE LOCALE)	23 144,95	11 572,47	5 786,24	3 471,74	2 314,49									2512,288			
25	SEK	183 862,52	91 931,26	45 965,63	27 579,38	18 386,25											11021,403	
26	SHITURU MINING	801 749,35	400 874,68	200 437,34	120 262,40	80 174,94		919,385										
27	SOMIKA	712 494,22	356 247,11	178 123,56	106 874,13	71 249,42		3971,68										
28	TSM	5 871,14	2 935,57	1 467,78	880,67	587,11		1923								679,875		
												23,96383						
	TOTAL	17 599 087,58	8 799 543,79	4 399 771,89	2 639 863,14	1 759 908,76	466,44	42 859,96	28 005,36	9 060,15	5 290,01	33,96	234,10	52,90	2 512,29	11 447,07	27 104,04	278,38

Le total général est de: Dix-sept million cinq cent nonante-neuf mille quatre-vingt-sept dollars, cinquante-huit centimes

<u>Légende:</u>

Carbonate de cobalt: (Carb.Co);Cathode de cobalt (Cath.co) :Cobalt Electrode (Co_ELECTRO);Cuivre de Décuivrage (Cu.DEC); Cathode de cuivre: (Cath.Cu); Concentré de Manganèse: (Conc.Mn); concentré de cobalt : (Conc.co); Concentré de cuivre: (Conc.cu); concentré cupro-cobaltifère: (Conc.CuCo);

Concentré d'Etain (Conc.ETAIN); Concentré d'Etain (Conc.TANTALE); Dechet de Cuivre(Dechet_Cu);Cuivre Blister(CU_BLST); Cuivre noir(CU.NOIR); Cuivre Pulverisé: (Cu.Pulv); Hydroxyde de Cobalt: (Hydro.Co);

Cobalt separateur Magnétique(Co.SEPAR);Matte de cuivre: (Matt.Cu); Minerais de cuivre: (Min.Cu); Minerais de cobalt: (Min.Co); Craps de cuivre(craps.Cu); Cuivre Autrement présenté(Cu.AUTRE); Concentré de Cuivre et Argent (CONC.CU.Arg); Déchet d'Anodes(Déch.Ano); Anodes Solides(Ano.Sol), Sulfate de Plomb (Sulf.Plomb)

République Démocratique du Congo

DIVISION PROVINCIALE DES MINES
DU HAUT-KATANGA
LUBUMBASHI

Le Chef de Division

STATISTIQUES DE LA REDEVANCE MINIERE PAR QUOTITE EMISES POUR LA PERIODE D'OCTOBRE 2019 (HAUT-KATANGA)

							QUANTITES DES PRODUITS EXPORTES EN TONNES								
N°	SOCIETE	REDEVANCE 100%	TERSOR 50%	PROVINCE 25%	TERRITOIRE 15%	FONDS MINIERS 10%	CATH_CU	CONC_CU	CONC_CUCO	CONC_ETAIN	CU_NOIR	CU_PULV	HYDRO_CO	Min_Cu	SULFATE DE PLOMB
1	AMICAL KAKANA MINING	4 147,50	2 073,75	1 036,88	622,13	414,75		150							
2	CDM	1 532 993,88	766 496,94	383 248,47	229 949,08	153 299,39	3485,629		2479,096				715,647		
3	CHEMAF	1 689 202,12	844 601,06	422 300,53	253 380,32	168 920,21	2245			10			2267,691		
4	CNMC CONGO COMPAGNIE	220 008,24	110 004,12	55 002,06	33 001,24	22 000,82	1094,162								
5	CNMC HUACHIN MABENDE	639 450,56	319 725,28	159 862,64	95 917,58	63 945,06	3180,165								
6	COMIKA	846 022,29	423 011,15	211 505,57	126 903,34	84 602,23	620,631		2234,748				585,704		
7	COMILU	344 247,44	172 123,72	86 061,86	51 637,12	34 424,74	1712,038								
8	CONGO JIN JU	44 868,08	22 434,04	11 217,02	6 730,21	4 486,81	223,141								
9	COPPERCO(VENTE LOCALE)	6 024,38	3 012,19	1 506,09	903,66	602,44		750							
10	FRONTIER	1 442 636,42	721 318,21	360 659,11	216 395,46	144 263,64		33858,8							
11	GCM	350 635,91	175 317,96	87 658,98	52 595,39	35 063,59	1481,828					150,056			636,3
12	GOLDEN AFRICAN	160 685,39	80 342,69	40 171,35	24 102,81	16 068,54	799								
13	HUACHIN METAL LEACH	557 360,03	278 680,01	139 340,01	83 604,00	55 736,00	2771,906								
14	KAIPENG	442 556,47	221 278,23	110 639,12	66 383,47	44 255,65	2194,223								
15	KICC(VENTE LOCALE)	24 669,94	12 334,97	6 167,49	3 700,49	2 466,99		320,959							
16	LONG FEI(VENTE LOCALE)	42 687,72	21 343,86	10 671,93	6 403,16	4 268,77								10614,894	
17	LUALABA MINING(VENTE LOCALE)	32 360,74	16 180,37	8 090,18	4 854,11	3 236,07								24168,492	
18	METAL MINES	785 199,21	392 599,61	196 299,80	117 779,88	78 519,92	893						1049,922		
19	MIKAS	112 994,64	56 497,32	28 248,66	16 949,20	11 299,46	501,81						58,85		
20	MJM	44 632,01	22 316,01	11 158,00	6 694,80	4 463,20	221,967								
21	MMG	1 495 316,17	747 658,08	373 829,04	224 297,42	149 531,62	7436,622								
22	OM METAL	93 030,00	46 515,00	23 257,50	13 954,50	9 303,00	450								
23	RUASHI MINING	2 943 855,54	1 471 927,77	735 963,89	441 578,33	294 385,55	3576,608						2794,07		
24	RUBAMIN	289 530,51	144 765,25	72 382,63	43 429,58	28 953,05					1999,884				
24	RUBAMIN(VENTE LOCALE)	8 201,43	4 100,71	2 050,36	1 230,21	820,14								9512,796	
25	SEK	268 389,21	134 194,61	67 097,30	40 258,38	26 838,92	1334,774								
26	SHITURU MINING	752 729,19	376 364,59	188 182,30	112 909,38	75 272,92	3743,531								
27	SOMIKA	525 992,76	262 996,38	131 498,19	78 898,91	52 599,28	2368						84,78		
28	T3M	5 674,71	2 837,36	1 418,68	851,21	567,47				23,1621					
	TOTAL	15 706 102,48	7 853 051,24	3 926 525,62	2 355 915,37	1 570 610,25	40 334,04	35 079,76	4 713,84	33,16	1 999,88	150,06	7 556,66	44 296,18	636,30

Le total général est de: Quinze million sept cent six mille cent et deux dollars, quarante-huit centimes

Légende:

Carbonate de cobalt: (Carb.Co);Cathode de cobalt (Cath.co) :Cobalt Electrode (Co_ELECTRO);Cuivre de Décuivrage (Cu.DEC); Cathode de cuivre: (Cath.Cu);   Concentré de Manganèse: (Conc.Mn); concentré de cobalt : (Conc.co); Concentré de cuivre: (Conc.cu); concentré cupro-cobaltifère: (Conc.CuCo);

Concentré d'Etain (Conc.ETAIN); Concentré d'Etain (Conc.TANTALE); Dechet de Cuivre(Dechet_Cu);Cuivre Blister(CU_BLST); Cuivre noir(CU.NOIR); Cuivre Pulverisé: (Cu.Pulv); Hydroxyde de Cobalt: (Hydro.Co);

Cobalt separateur Magnétique(Co.SEPAR);Matte de cuivre: (Matt.Cu);   Minerais de cuivre: (Min.Cu); Minerais de cobalt: (Min.Co); Craps de cuivre(craps.Cu); Cuivre Autrement présenté(Cu.AUTRE); Concentré de Cuivre et Argent (CONC.CU.Arg); Déchet d'Anodes(Déch.Ano); Anodes Solides(Ano.Sol), Sulfate de Plomb (Sulf.Plomb); Oxyde de Zinc (OXYDE.ZINC),

République Démocratique du Congo

DIVISION PROVINCIALE DES MINES
DU HAUT-KATANGA
LUBUMBASHI

Le Chef de Division

STATISTIQUES DE LA REDEVANCE MINIERE PAR QUOTITE EMISES POUR LA PERIODE DE NOVEMBRE 2019 (HAUT-KATANGA)

							QUANTITES DES PRODUITS COMMERCIALISES EN TONNES											
N°	SOCIETE	REDEVANCE 100%	TERSOR 50%	PROVINCE 25%	TERRITOIRE 15%	FONDS MINIERS 10%	ALL_BLANC	CATH_CU	CONC_CU	CONC_CUCO	CONC_ETAIN	CU_NOIR	CU_PULV	HYDRO_CO	Min_Co	Min_Cu	OXYDE_ZINC
1	ANVIL MINING	695 758,97	347 879,48	173 939,74	104 363,85	69 575,90			19232,05								
2	CDM	1 387 794,92	693 897,46	346 948,73	208 169,24	138 779,49		3936,515		1524,849				601,329			
3	CHEMAF	2 434 599,69	1 217 299,84	608 649,92	365 189,95	243 459,97		2931			40			3211,651			
4	CNMC CONGO COMPAGNIE	289 085,84	144 542,92	72 271,46	43 362,88	28 908,58		1438,355									
5	CNMC HUACHIN MABENDE	564 008,76	282 004,38	141 002,19	84 601,31	56 400,88		2806,242									
6	COMIKA	1 379 520,00	689 760,00	344 880,00	206 928,00	137 952,00		510,629		6686,814				303,493			
7	COMILU	402 281,61	201 140,81	100 570,40	60 342,24	40 228,16		2001,315									
8	CONGO JIN JU	39 014,27	19 507,13	9 753,57	5 852,14	3 901,43		194,096									
9	FRONTIER	1 577 781,98	788 890,99	394 445,50	236 667,30	157 778,20			36768,656								
10	GCM	955 982,33	477 991,16	238 995,58	143 397,35	95 598,23	1213,337	1486,207					89,944				255,371
11	GOLDEN AFRICAN	183 361,19	91 680,60	45 840,30	27 504,18	18 336,12		912									
12	HUACHIN METAL LEACH	440 845,18	220 422,59	110 211,29	66 126,78	44 084,52		2193,438									
13	KAIPENG	555 505,03	277 752,51	138 876,26	83 325,75	55 550,50		2708,967									
14	KICC(VENTE LOCALE)	350 984,72	175 492,36	87 746,18	52 647,71	35 098,47			4729,16								
15	LONG FEI(VENTE LOCALE)	55 833,91	27 916,96	13 958,48	8 375,09	5 583,39										13883,876	
16	LUALABA MINING(VENTE LOCALE)	36 316,41	18 158,20	9 079,10	5 447,46	3 631,64										28979,537	
17	METAL MINES	730 842,88	365 421,44	182 710,72	109 626,43	73 084,29		724						1023,9945			
18	MIKAS	682 471,87	341 235,93	170 617,97	102 370,78	68 247,19		807,599						908,44			
19	MJM	137 498,88	68 749,44	34 374,72	20 624,83	13 749,89		684,057									
20	MMG	1 061 822,55	530 911,27	265 455,64	159 273,38	106 182,25		5283,129									
21	OM METAL	48 720,00	24 360,00	12 180,00	7 308,00	4 872,00		240									
22	RUASHI MINING	3 789 064,38	1 894 532,19	947 266,09	568 359,66	378 906,44		2733,639						3745,31118			
22	RUASHI MINING(VENTE LOCALE)	1 585,76	792,88	396,44	237,86	158,58		7,89									
23	RUBAMIN	288 699,42	144 349,71	72 174,85	43 304,91	28 869,94						1994,953					
24	SEK	195 242,35	97 621,18	48 810,59	29 286,35	19 524,24		971,434									
25	SHAMITUMBA (VENTE LOCAL)	341 745,20	170 872,60	85 436,30	51 261,78	34 174,52									10096,15	99107,51	
26	SHITURU MINING	650 702,91	325 351,45	162 675,73	97 605,44	65 070,29		3237,374									
27	SOMIKA	366 312,24	183 256,12	91 628,06	54 976,84	36 651,22		1766		69,79							
	TOTAL	**19 643 583,23**	**9 821 791,61**	**4 910 895,81**	**2 946 537,48**	**1 964 358,32**	**1 213,34**	**37 573,89**	**60 729,87**	**8 281,45**	**40,00**	**1 994,95**	**89,94**	**9 794,22**	**10 096,15**	**141 970,92**	**255,37**

Le total général est de: Dix-neuf million six cent quarante-trois mille cinq cent quatre-vingt-trois dollars, vingt-trois centimes

<u>Légende:</u>

Carbonate de cobalt: (Carb.Co);Cathode de cobalt (Cath.co) :Cobalt Electrode (Co_ELECTRO);Cuivre de Décuivrage (Cu.DEC); Cathode de cuivre: (Cath.Cu); Concentré de Manganèse: (Conc.Mn); concentré de cobalt : (Conc.co); Concentré de cuivre: (Conc.cu); concentré cupro-cobaltifère: (Conc.CuCo);

Concentré d'Etain (Conc.ETAIN); Concentré d'Etain (Conc.TANTALE); Dechet de Cuivre(Dechet_Cu);Cuivre Blister(CU_BLST); Cuivre noir(CU.NOIR); Cuivre Pulverisé: (Cu.Pulv); Hydroxyde de Cobalt: (Hydro.Co);

Cobalt separateur Magnétique(Co.SEPAR);Matte de cuivre: (Matt.Cu); Minerais de cuivre: (Min.Cu); Minerais de cobalt: (Min.Co); Craps de cuivre(craps.Cu); Cuivre Autrement présenté(Cu.AUTRE); Concentré de Cuivre et Argent (CONC.CU.Arg); Déchet d'Anodes(Déch.Ano); Anodes

République Démocratique du Congo

DIVISION PROVINCIALE DES MINES
DU HAUT-KATANGA
LUBUMBASHI
Le Chef de Division

STATISTIQUES DE LA REDEVANCE MINIERE PAR QUOTITE EMISES POUR LA PERIODE DE DECEMBRE 2019 (HAUT-KATANGA)

							QUANTITES DES PRODUITS COMMERCIALISES EN TONNES										
N°	SOCIETE	REDEVANCE 100%	TERSOR 50%	PROVINCE 25%	TERRITOIRE 15%	FONDS MINIERS 10%	ALL_BLANC	CATH_CU	CONC_CU	CONC_CUCO	CONC_ETAIN	CU_NOIR	Dechet_Cu	HYDRO_CO	Matte_Cu	Min_Cu	OXYDE_ZINC
1	AMICAL KAKANA MINING	6 347,25	3 173,63	1 586,81	952,09	634,73			225								
2	ANVIL MINING	713 401,90	356 700,95	178 350,47	107 010,28	71 340,19			19404,352								
3	CDM	1 605 077,06	802 538,53	401 269,26	240 761,56	160 507,71		5672,691		1731,3				270,02			
3	CDM(VENTE LOCALE)	17 108,93	8 554,46	4 277,23	2 566,34	1 710,89									185,394		
4	CHEMAF	1 039 897,34	519 948,67	259 974,33	155 984,60	103 989,73		913			30			1475,884			
5	CNMC CONGO COMPAGNIE	395 443,45	197 721,73	98 860,86	59 316,52	39 544,35		1928,279									
5	CNMC CONGO COMPAGNIE(VENTE LOCALE)	58 976,44	29 488,22	14 744,11	8 846,47	5 897,64			1069,167								
6	CNMC HUACHIN MABENDE	563 303,58	281 651,79	140 825,89	84 495,54	56 330,36		2746,806									
7	COMIKA	790 498,41	395 249,21	197 624,60	118 574,76	79 049,84		871,707		866,285				816,907			
8	COMILU	324 581,96	162 290,98	81 145,49	48 687,29	32 458,20		1582,741									
9	CONGO JIN JU	32 735,60	16 367,80	8 183,90	4 910,34	3 273,56		159,608									
10	FRONTIER	1 922 669,71	961 334,85	480 667,43	288 400,46	192 266,97			45603								
11	GCM	1 752 498,28	876 249,14	438 124,57	262 874,74	175 249,83	2920,94	794,351									1791,259
12	GOLDEN AFRICAN	120 994,75	60 497,38	30 248,69	18 149,21	12 099,48		590									
13	HUACHIN METAL LEACH	554 165,40	277 082,70	138 541,35	83 124,81	55 416,54		2702,246									
14	KAIPENG	481 181,52	240 590,76	120 295,38	72 177,23	48 118,15		2290,239									
15	KICC(VENTE LOCALE)	796 561,66	398 280,83	199 140,41	119 484,25	79 656,17			10991,401								
16	LONG FEI(VENTE LOCALE)	21 105,05	10 552,53	5 276,26	3 165,76	2 110,51										5145,67	
17	LUALABA MINING(VENTE LOCALE)	14 805,22	7 402,61	3 701,30	2 220,78	1 480,52										12864,137	
18	METAL MINES	520 016,08	260 008,04	130 004,02	78 002,41	52 001,61		1347						425,5253			
19	MIKAS	716 469,48	358 234,74	179 117,37	107 470,42	71 646,95		1057,882						872,054			
20	MJM	123 889,42	61 944,71	30 972,36	18 583,41	12 388,94		604,044									
21	MMG	1 655 388,47	827 694,23	413 847,12	248 308,27	165 538,85		8088,63									
22	OM METAL	76 650,00	38 325,00	19 162,50	11 497,50	7 665,00		365									
23	PIMA MINING(VENTE LOCALE)	20 107,47	10 053,73	5 026,87	3 016,12	2 010,75										4000	
24	RUASHI MINING	1 937 746,59	968 873,30	484 436,65	290 661,99	193 774,66		3053,334						1537,61			
25	RUBAMIN	393 354,25	196 677,12	98 338,56	59 003,14	39 335,42						2664,018					
25	RUBAMIN(VENTE LOCALE)	32 968,57	16 484,29	8 242,14	4 945,29	3 296,86										13655,72	
26	SEK	229 935,68	114 967,84	57 483,92	34 490,35	22 993,57		1125,474									
26	SEK(VENTE LOCALE)	5 623,64	2 811,82	1 405,91	843,55	562,36							36,263				
27	SHITURU MINING	563 472,23	281 736,12	140 868,06	84 520,83	56 347,22		2749,501									
28	SOMIKA	437 067,35	218 533,68	109 266,84	65 560,10	43 706,74		2131									
28	SOMIKA(VENTE LOCALE)	103 211,46	51 605,73	25 802,86	15 481,72	10 321,15										25163,4	
	TOTAL	**18 027 254,18**	**9 013 627,09**	**4 506 813,54**	**2 704 088,13**	**1 802 725,42**	**2 920,94**	**40 773,53**	**77 292,92**	**2 597,59**	**30,00**	**2 664,02**	**36,26**	**5 398,00**	**185,39**	**60 828,93**	**1 791,26**

Le total général est de: Dix-huit million vingt-sept mille deux cent cinquante-quatre dollars, dix-huit centimes

Légende:

Carbonate de cobalt: (Carb.Co);Cathode de cobalt (Cath.co) :Cobalt Electrode (Co_ELECTRO);Cuivre de Décuivrage (Cu.DEC); Cathode de cuivre: (Cath.Cu); Concentré de Manganèse: (Conc.Mn); concentré de cobalt : (Conc.co); Concentré de cuivre: (Conc.cu); concentré cupro-cobaltifère: (Conc.CuCo);

Concentré d'Etain (Conc.ETAIN); Concentré d'Etain (Conc.TANTALE); Dechet de Cuivre(Dechet_Cu);Cuivre Blister(CU_BLST); Cuivre noir(CU.NOIR); Cuivre Pulverisé: (Cu.Pulv); Hydroxyde de Cobalt: (Hydro.Co);

Cobalt separateur Magnétique(Co.SEPAR);Matte de cuivre: (Matt.Cu); Minerais de cuivre: (Min.Cu); Minerais de cobalt: (Min.Co); Craps de cuivre(craps.Cu); Cuivre Autrement présenté(Cu.AUTRE); Concentré de Cuivre et Argent (CONC.CU.Arg); Déchet d'Anodes(Déch.Ano); Anodes

STATISTIQUES DE PRODUCTION DES PRODUITS MINIERS MARCHANDS POUR L'ANNEE 2019/PROVINCE DU LUALABA

	QUANTITE DES PRODUITS EXPORTES EN TONNES												
SOCIETES	Alliage Rouge	Carb Co	Cath. Cu	Conc Mn	Conc Co	Conc Cu	Conc CuCo	Cu Blst	Hyd Co	Matt Cu	MINERAI DE CUIVRE	MINERAI DE COBALT	SCRAPS Cu
BOSS MINING		1,774.980	4,366.350										
CCR			8,794.279						2,583.260				
CDM									833.000				
COMIKA			284.050						282.763				
COMMUS			25,687.031			28,942.470		35,597.594	1,675.924	5,880.098			
KATANGA METALS	198.827							1,994.623					
KCC			230,982.764						14,855.450				3,457.060
KIMIN									160.010				
METALKOL			50,815.032						18,656.862				
MINING PROGRESS					3,057.500								
MKM			28,527.499						5,803.615				
MUMI			105,987.056						82,391.860				837.866
SCM			64,486.307			72,011.455			2,341.344				
SCMK-Mn SA				11,347.315									
TAVIR								4,137.354					
TCC			11,452.759						3,490.002				
TFM			15,188.79						3,096.46				
THOMAS MINING								2,569.750					
**TOTAL**	*198.83*	*1774.98*	*546,571.917*	*11,347.315*	*3,057.500*	*100,953.925*		*44,299.321*	*136,170.551*	*5,880.098*			*4,294.926*

TABLEAU DE LA REDEVANCE MINIERE DE JANVIER A DECEMBRE 2019 EN APPLICATION DES DISPOSITIONS DU CODE MINIER REVISE

MONTANT (JANVIER A DECEMBRE 2019, en application des dipositions du code minier révisé et du décret n°18/042 du 24 Novembre 2019

MOIS	MONTANT REDEVANCE 100 % (USD)	TRESOR 50 % (USD)	PROVINCE 25 % (USD)	ETD 15 % (USD)	FONDS MINIER 10 % (USD)
JANVIER	26,353,763.76	13,176,881.91	6,588,440.93	3,953,064.58	2,635,375.38
FEVRIER	25,986,014.44	12,993,007.22	6,496,503.61	3,897,902.17	2,598,601.44
MARS	22,857,287.51	11,428,643.76	5,714,321.88	3,428,593.13	2,285,728.75
AVRIL	20,789,483.62	10,394,741.81	5,197,370.91	3,118,422.54	2,078,948.36
MAI	29,228,239.52	14,614,119.76	7,307,059.88	4,384,235.93	2,922,823.95
JUIN	24,883,361.35	12,441,680.67	6,220,840.34	3,732,504.20	2,488,336.13
JUILLET	24,021,053.10	12,010,526.55	6,005,263.27	3,603,157.96	2,402,105.31
AOUT	25,938,184.00	12,969,092.00	6,484,564.00	3,890,725.60	2,593,818.40
SEPTEMBRE	23,811,511.22	11,905,755.61	5,952,877.81	3,571,726.68	2,381,151.12
OCTOBRE	28,938,219.59	14,469,109.80	7,234,554.90	4,340,732.94	2,893,821.96
NOVEMBRE	24,647,381.85	12,323,690.92	6,161,845.46	3,697,107.28	2,464,738.18
DECEMBRE	23,044,751.06	11,522,375.53	5,761,187.77	3,456,712.66	2,304,475.11
**TOTAUX**	**300,499,251.02**	**150,249,625.54**	**75,124,830.76**	**45,074,885.67**	**30,049,924.09**

REPUBLIQUE DEMOCRATIQUE DU CONGO

DIVISION PROVINCIALE DES MINES
HAUT-KATANGA
LUBUMBASHI

STATISTIQUES DE LA REDEVANCE MINIERE CONSTATEE ET LIQUIDEE
CUMUL DE JANVIER A DECEMBRE 2020 (HAUT-KATANGA)

N°	SOCIETE	REDEVANCE 100%	CUMUL DE QUANTITES PAR PRODUIT (EXPORT ET VENTE LOCALE)																	
			ALL_BLANC	CALCINE_ZINC	CATH_CU	CONC_CO	Conc_Cu	CONC_CU_Arg	CONC_CUCO	CONC_ETAIN	CU_AUTRE_P	CU_NOIR	CU_PULV	Cu_Rejet	Dechet_cu	HYDRO_CO	Min_Cu	OXYDE_ZINC	Pierre_Couleur	Pouss_ZINC
1	AMICAL KAKANA MINING	108,194.44					3550													
2	ANVIL MINING	2,260,649.55					4427.5152	54687.8329												
2	ANVIL MINING(VENTE LOCALE)	318,342.01					9866.402													
3	CDM	19,801,376.60			66803.361				21094.573							5106.2403			6	
3	CDM(VENTE LOCALE)	132,867.00															3239.355			
4	CHEMAF	16,498,300.73			23770					247.5						21948.717				
5	CNMC CONGO COMPAGNIE	4,632,715.32			16844.807		1230.836									1332.6759				
5	CNMC CONGO COMPAGNIE(VENTE LOCALE)	418,216.40					8692.601													
6	CNMC HUACHIN MABENDE	7,796,722.82			36632.367															
7	COMIKA	6,837,371.90			16028.981											6218.4451				
8	COMILU	6,305,807.53			29150.539											298.481				
8	COMILU(VENTE LOCALE)	774,116.70					21168.577													
9	CONGO JIN JU	2,088,864.04			9302.0433															
10	DIVINE LAND MINING SARL	971,565.64			4342.4535															
11	EXCELLEN MINERALS SARL	3,848,007.47			16492.924															
12	FRONTIER	10,059,025.23					230319.5													
12	FRONTIER(VENTE LOCALE)	4,840,507.53					96713.134													
13	GCM	6,632,258.22	3337.733	529.478	21663.409								281.218					3810.987		
13	GCM(VENTE LOCALE)	1,978,584.02												392300			15000			
14	GOLDEN AFRICAN	2,132,463.27			10206.5															
14	GOLDEN AFRICAN (VENTE LOCALE)	46,382.60			214															
15	HUACHIN METAL LEACH	5,969,601.43			26738.345											408.39				
16	KAIPENG	7,333,285.43			33543.658															
17	KANSONGA(VENTE LOCALE)	11,409.24															2866.5			
18	KICC	0.00																		
18	KICC(VENTE LOCALE)	4,069,150.98					75639.193													
19	LONG FEI(VENTE LOCALE)	17,281.80															3956.742			
20	LUALABA MINING(VENTE LOCALE)	1,131,769.68															407772.152			
21	METAL MINES	8,426,787.75			13914											10589.3733				
22	MIKAS	3,945,929.92			5619.178											5286.1582				
23	MJM	3,084,140.93			10913.536											1582.1311				
24	MMG	15,035,937.17			71751.687															
24	MMG(VENTE LOCALE)	13,674.27													63.006					

N°	SOCIETE	REDEVANCE 100%	ALL_BLANC	CALCINE_ZINC	CATH_CU	CONC_CO	Conc_Cu	CONC_CU_Arg	CONC_CUCO	CONC_ETAIN	CU_AUTRE_P	CU_NOIR	CU_PULV	Cu_Rejet	Dechet_cu	HYDRO_CO	Min_Cu	OXYDE_ZINC	Pierre_Couleur	Pouss_ZINC
25	MMR	35,289.39								306.223										
26	MPC	766,115.00				2597														
27	OM METAL	905,766.40			4174						132									
28	RUASHI MINING	17,763,663.92			33483.5137											13940.61514				
29	RUBAMIN	3,556,757.59										23772.971								
29	RUBAMIN(VENTE LOCALE)	1,180,778.32															316323.45			
30	SEK	1,038,360.31			5018.349															
30	SEK(VENTE LOCALE)	855.42													5.612					
31	SEMHKAT(VENTE LOCALE)	112,415.27															26000			
32	SHAMITUMBA (VENTE LOCALE)	575,376.49															122154.12			
33	SHITURU MINING	6,480,906.55			30331.793															
34	SOCIETE MINIERE RESOURCES RENOUVELLABLE YING XING SARL(VENTE LOCALE)	42,673.78					2099.503													
35	SOMIKA	5,517,673.65			23320				2945.87							318.3				
35	SOMIKA(VENTE LOCALE)	143,266.74															33225.26			
36	STL	11,655,755.03	28884.077																	23664.991
	**TOTAL**	**197,296,961.47**	**32,221.81**	**529.48**	**510,259.44**	**2,597.00**	**453,707.26**	**54,687.83**	**24,040.44**	**553.72**	**132.00**	**23,772.97**	**281.22**	**392,300.00**	**68.62**	**67,029.53**	**930,537.58**	**3,810.99**	**6.00**	**23,664.99**

Le montant Total redevance 100% et de: Cent nonante-sept millions deux cent nonante-six milles neuf cent soixante-un dollars américains, quarante-sept centimes

Légende:

Carbonate de cobalt: (Carb.Co);Cathode de cobalt (Cath.co) :Cobalt Electrode (Co_ELECTRO);Cuivre de Décuivrage (Cu.DEC); Cathode de cuivre: (Cath.Cu); Concentré de Manganèse: (Conc.Mn); concentré de cobalt : (Conc.co); Concentré de cuivre: (Conc.cu); concentré cupro-cobaltifère: (Conc.CuCo);
Concentré d'Etain (Conc.ETAIN); Concentré d'Etain (Conc.TANTALE); Dechet de Cuivre(Dechet_Cu);Cuivre Blister(CU_BLST); Cuivre noir(CU.NOIR); Cuivre Pulverisé: (Cu.Pulv); Hydroxyde de Cobalt: (Hydro.Co);
Cobalt separateur Magnétique(Co.SEPAR);Matte de cuivre: (Matt.Cu); Minerais de cuivre: (Min.Cu); Minerais de cobalt: (Min.Co); Craps de cuivre(craps.Cu); Cuivre Autrement présenté(Cu.AUTRE); Concentré de Cuivre et Argent (CONC.CU.Arg); Déchet d'Anodes(Déch.Ano); Anodes Solides(Ano.Sol), Sulfate de Plomb (Sulf.Plomb); Oxyde de Zinc (OXYDE.ZINC), CALCINE.ZIN(Calcine de Zinc); Poussière de Zinc(Pouss.zinc)

STATISTIQUES DE PRODUCTION, REDEVANCE MINIERE (MONTANTS ATTENDUS) PAR ENTITE BENEFICIAIRE POUR LE MOIS DE JANVIER 2020/ GUICHET UNIQUE KOLWEZI

SOCIETES	REDEVANCE MINIERE 100% (USD)	TRESOR 50%	PROVINCE 25%	TERRITOIRE 15%	FONDS MINIER 10%
BOSS MINING	0,00	0,00	0,00	0,00	0,00
CCR	746.239,03	373.119,51	186.559,76	111.935,85	74.623,90
CDM	0,00	0,00	0,00	0,00	0,00
COMIKA	0,00	0,00	0,00	0,00	0,00
COMMUS	2.574.322,86	1.287.161,43	643.580,71	386.148,43	257.432,29
KATANGA METALS	47.975,66	23.987,83	11.993,91	7.196,35	4.797,57
KCC	7.022.838,11	3.511.419,06	1.755.709,53	1.053.425,72	702.283,81
LCS	209.293,48	104.646,74	52.323,37	31.394,02	20.929,35
METALKOL	3.889.392,68	1.944.696,34	972.348,17	583.408,90	388.939,27
MINING PROGRESS	0,00	0,00	0,00	0,00	0,00
MKM	428.643,52	214.321,76	107.160,88	64.296,53	42.864,35
MUMI	0,00	0,00	0,00	0,00	0,00
SCM	0,00	0,00	0,00	0,00	0,00
SCMK-Mn SA	0,00	0,00	0,00	0,00	0,00
TAVIR	0,00	0,00	0,00	0,00	0,00
TCC	407.861,17	203.930,58	101.965,29	61.179,17	40.786,12
THOMAS MINING	0,00	0,00	0,00	0,00	0,00
TOTAL	*15.326.566,50*	*7.663.283,25*	*3.831.641,62*	*2.298.984,97*	*1.532.656,65*

Le total est de: **Quinze Million Trois Cents Vingt-Six Mille Cinq Cents Soixante-Six et Cinquante Centimes**

STATISTIQUES DE PRODUCTION, REDEVANCE MINIERE PAR QUOTITES EMISES POUR LE MOIS DE DE JANVIER 2019/GUICHET UNIQUE FUNGURUME

SOCIETE	REDEVANCE MINIERE 100% (USD)	TRESOR 50%	PROVINCE 25%	TERRITOIRE 15%	FONDS MINIER 10%
TFM	*5.801.876,81*	*2.900.938,41*	*1.450.469,20*	*870.281,52*	*580.187,68*

Le total est de: **Cinq Million Huit Cents Un Mille Huit Cents Septante-Six et Quatre-Vingt-Un Centimes**

TOTAL GENERAL PAR QUOTITE POUR LE MOIS DE DECEMBRE 2019 / GUICHET UNIQUE KOLWEZI ET FUNGURUME

REDEVANCE MINIERE 100% (USD)	TRESOR 50%	PROVINCE 25%	TERRITOIRE 15%	FONDS MINIER 10%
21.128.443,31	10.564.221,66	5.282.110,83	3.169.266,50	2.112.844,33

Le ToTal est de : **Vingt et Un Million Cent Vingt-Huit Mille Quatre Cents Quarente-Trois et Trente et Un Centimes**

	QUANTITE DES PRODUITS EXPORTES EN TONNES												
SOCIETES	Alliage Rouge	Carb Co	Cath. Cu	Conc Mn	Conc Co	Conc Cu	Conc CuCo	Cu Blst	Hyd Co	Matt Cu	MINERAI DE CUIVRE	MINERAI DE COBALT	SCRAPS Cu
BOSS MINING													
CCR			1.396,76						840,00				
CDM													
COMIKA													
COMMUS			5.179,75			2.643,38		2.789,98	957,44	474,62			
KATANGA METALS								264,46					
KCC			22.657,16						2.136,81				279,88
LCS								1.036,24					
METALKOL			5.801,08						3.193,23				
MINING PROGRESS													
MKM			1.064,65						244,77				
MUMI													
SCM			7.757,94			4.819,47							
SCMK-Mn SA													
TAVIR													
TCC			1.508,02						200,50				
THOMAS MINING													
TOTAL		*0*	*45.365,35*	*0,00*		*7.462,85*		*4.090,68*	*7.572,75*	*474,62*			*279,88*

		QUANTITE DES PRODUITS EXPORTES EN TONNES											
SOCIETE	Alliage Rouge	Carb Co	Cath. Cu	Conc Mn	Conc Co	Conc Cu	Conc CuCo	Cu Blst	Hyd Co	Matt Cu	MINERAI DE CUIVRE	MINERAI DE COBALT	SCRAPS Cu
TFM			*13.724,01*						*3.149,76*				

STATISTIQUES DE PRODUCTION, REDEVANCE MINIERE (MONTANTS ATTENDUS) PAR ENTITE BENEFICIAIRE POUR LE MOIS DE FEVRIER 2020/ GUICHET UNIQUE KOLWEZI

SOCIETES	REDEVANCE MINIERE 100% (USD)	TRESOR 50%	PROVINCE 25%	TERRITOIRE 15%	FONDS MINIER 10%
BOSS MINING	0,00	0,00	0,00	0,00	0,00
CCR	405.354,72	202.677,36	101.338,68	60.803,21	40.535,47
CDM	0,00	0,00	0,00	0,00	0,00
COMIKA	0,00	0,00	0,00	0,00	0,00
COMMUS	1.881.486,70	940.743,35	470.371,68	282.223,01	188.148,67
KATANGA METALS	0,00	0,00	0,00	0,00	0,00
KCC	5.556.856,26	2.778.428,13	1.389.214,07	833.528,44	555.685,63
KIMIN	1.654.282,95	827.141,48	413.570,74	248.142,44	165.428,30
LCS	795.719,63	397.859,81	198.929,91	119.357,94	79.571,96
METALKOL	3.991.918,51	1.995.959,26	997.979,63	598.787,78	399.191,85
MINING PROGRESS	241.450,60	120.725,30	60.362,65	36.217,59	24.145,06
MKM	685.276,21	342.638,11	171.319,05	102.791,43	68.527,62
MUMI	5.254,67	2.627,34	1.313,67	788,20	525,47
SCM	0,00	0,00	0,00	0,00	0,00
SCMK-Mn SA	0,00	0,00	0,00	0,00	0,00
SM DEZIWA	602.415,21	301.207,60	150.603,80	90.362,28	60.241,52
TAVIR	0,00	0,00	0,00	0,00	0,00
TCC	388.213,34	194.106,67	97.053,33	58.232,00	38.821,33
THOMAS MINING	0,00	0,00	0,00	0,00	0,00
TOTAL	*16.208.228,79*	*8.104.114,40*	*4.052.057,20*	*2.431.234,32*	*1.620.822,88*

Le total est de: Seize Million Deux Cents Huit Mille Deux Cents Vingt-Huit et Septante-Neuf Centimes

STATISTIQUES DE PRODUCTION, REDEVANCE MINIERE PAR QUOTITES EMISES POUR LE MOIS DE DE FEVRIER 2019/GUICHET UNIQUE FUNGURUME

SOCIETE	REDEVANCE MINIERE 100% (USD)	TRESOR 50%	PROVINCE 25%	TERRITOIRE 15%	FONDS MINIER 10%
TFM	*5.853.547,10*	*2.926.773,55*	*1.463.386,78*	*878.032,07*	*585.354,71*

Le total est de: Cinq Million Huit Cents Cinquente-Trois Mille Cinque Cents Quarante-Sept et Dix Centimes

TOTAL GENERAL PAR QUOTITE POUR LE MOIS DE FEVRIER 2020 / GUICHET UNIQUE KOLWEZI ET FUNGURUME

REDEVANCE MINIERE 100% (USD)	TRESOR 50%	PROVINCE 25%	TERRITOIRE 15%	FONDS MINIER 10%
22.061.775,90	11.030.887,95	5.515.443,97	3.309.266,38	2.206.177,59

Le ToTal est de : Vingt-Deux Million Soixante et Un Mille Sept Cent Septente-Cinq et Nonante Centimes

LEGENDE :

1. Carbonate de Co (Carb Co);
2. Cathode de Cuivre (Cath Cu)
3. Concentré de Manganèse (Conc Mn)
4. Concentré de Cobalt (Conc Co)
5. Concentré de Cuivre (Conc Cu)
6. Concentré Cupro-Cobaltifère (Conc Cu Co)
7. Cuivre Blister (Cu-Blst)
8. Hydroxyde de Cobalt (Hyd Co)
9. Matte de Cuivre (Matt Cu)
10. Minerai de Cuivre (Min Cu)
11. Minerai de Cobalt (Min Co)
12. Scraps de Cuivre (Scraps Cu)

	QUANTITE DES PRODUITS EXPORTES EN TONNES												
	Alliage Rouge	Carb Co	Cath. Cu	Conc Mn	Conc Co	Conc Cu	Conc CuCo	Cu Blst	Hyd Co	Matt Cu	MINERAI DE CUIVRE	MINERAI DE COBALT	SCRAPS Cu
**BOSS MINING**			0,00		0,00	0		0	0	0	0	0	0
**CCR**			964,82		0,00	0		0	385	0	0	0	0
**CDM**			0,00		0,00	0		0	0	0	0	0	0
**COMIKA**			0,00		0,00	0		0	0	0	0	0	0
**COMMUS**			3.905,03		0,00	2.052,55		3.261,99	367,29812	677,447	0	0	0
**KATANGA METALS**			0,00		0,00	0		0	0	0	0	0	0
**KCC**			16.039,97		0,00	0		0	2119,19586	0	0	0	184,346
**KIMIN**					0,00	0		0	571,332844	0	0	0	
**LCS**			0,00		0,00	0		4201,753	0	0	0	0	0
**METALKOL**			5.139,42		0,00	0		0	3671,938	0	0	0	0
**MINING PROGRESS**			0,00		450,00	0		0	306	0	0	0	0
**MKM**			2.042,80		0,00	0		0	321,094	0	0	0	0
**MUMI**			0,00		0,00	0		0	0	0	0	0	27,518
**SCM**			5.827,74		0,00	8510,491		0	0	0	0	0	0
**SCMK-Mn SA**			0,00		0,00	0		0	0	0	0	0	0
**SM DEZIWA**			2.845,57		0,00	0		0	0	0	0	0	0
**TAVIR**			0,00		0,00	0		0	0	0	0	0	0
**TCC**			1.299,02		0,00	0		0	252,38657	0	0	0	0
**THOMAS MINING**			0,00		0,00	0		0	0	0	0	0	0
		0	*38.064,35*	*0,00*	*450,00*	*10.563,04*		*7.463,74*	*7.994,25*	*677,45*			*211,86*

		QUANTITE DES PRODUITS EXPORTES EN TONNES											
**SOCIETE**	**Alliage Rouge**	**Carb Co**	**Cath. Cu**	**Conc Mn**	**Conc Co**	**Conc Cu**	**Conc CuCo**	**Cu Blst**	**Hyd Co**	**Matt Cu**	**MINERAI DE CUIVRE**	**MINERAI DE COBALT**	**SCRAPS Cu**
**TFM**			***14.623,79***						***3.127,87***				

STATISTIQUES DE PRODUCTION, REDEVANCE MINIERE (MONTANTS ATTENDUS) PAR ENTITE BENEFICIAIRE POUR LE MOIS DE MARS 2020/ GUICHET UNIQUE KOLWEZI

SOCIETES	REDEVANCE MINIERE 100% (USD)	TRESOR 50%	PROVINCE 25%	TERRITOIRE 15%	FONDS MINIER 10%
BOSS MINING	0,00	0,00	0,00	0,00	0,00
CCR	440.020,44	220.010,22	110.005,11	66.003,07	44.002,04
CDM	0,00	0,00	0,00	0,00	0,00
COMIKA	0,00	0,00	0,00	0,00	0,00
COMMUS	1.771.395,13	885.697,57	442.848,78	265.709,27	177.139,51
KATANGA METALS	0,00	0,00	0,00	0,00	0,00
KCC	7.874.226,76	3.937.113,38	1.968.556,69	1.181.134,01	787.422,68
KIMIN	124.674,46	62.337,23	31.168,61	18.701,17	12.467,45
LCS	1.374.060,01	687.030,00	343.515,00	206.109,00	137.406,00
METALKOL	3.556.228,04	1.778.114,02	889.057,01	533.434,21	355.622,80
MINING PROGRESS	55.991,25	27.995,63	13.997,81	8.398,69	5.599,13
MKM	304.013,80	152.006,90	76.003,45	45.602,07	30.401,38
MUMI	0,00	0,00	0,00	0,00	0,00
SCM	0,00	0,00	0,00	0,00	0,00
SCMK-Mn SA	0,00	0,00	0,00	0,00	0,00
SM DEZIWA	2.685.475,15	1.342.737,57	671.368,79	402.821,27	268.547,51
TAVIR	0,00	0,00	0,00	0,00	0,00
TCC	525.369,22	262.684,61	131.342,31	78.805,38	52.536,92
THOMAS MINING	0,00	0,00	0,00	0,00	0,00
TOTAL	*18.711.454,24*	*9.355.727,12*	*4.677.863,56*	*2.806.718,14*	*1.871.145,42*

Le total est de: **Dix Huit Million Sept Cents Onze Mille Quatre Centes Cinquente-Quatre et Vingt-Quatre Centimes**

STATISTIQUES DE PRODUCTION, REDEVANCE MINIERE PAR QUOTITES EMISES POUR LE MOIS DE DE MARS 2019/GUICHET UNIQUE FUNGURUME

SOCIETE	REDEVANCE MINIERE 100% (USD)	TRESOR 50%	PROVINCE 25%	TERRITOIRE 15%	FONDS MINIER 10%
TFM	*4.854.697,75*	*2.427.348,88*	*1.213.674,44*	*728.204,66*	*485.469,78*

Le total est de: **Quatre Million Huit Cents Cinqente-Quatre Mille Six Cents Nonante-Sept et Septente-Cinq Centimes**

TOTAL GENERAL PAR QUOTITE POUR LE MOIS DE MARS 2020 / GUICHET UNIQUE KOLWEZI ET FUNGURUME

REDEVANCE MINIERE 100% (USD)	TRESOR 50%	PROVINCE 25%	TERRITOIRE 15%	FONDS MINIER 10%
23.566.152,00	11.783.076,00	5.891.538,00	3.534.922,80	2.356.615,20

Le ToTal est de : **Vingt-Trois Million Cinq Cents Soixante-Six Mille Cent Cinquente-Deux**

LEGENDE :

1. Carbonate de Co (Carb Co);
2. Cathode de Cuivre (Cath Cu)
3. Concentré de Manganèse (Conc Mn)
4. Concentré de Cobalt (Conc Co)
5. Concentré de Cuivre (Conc Cu)
6. Concentré Cupro-Cobaltifère (Conc Cu Co)
7. Cuivre Blister (Cu-Blst)
8. Hydroxyde de Cobalt (Hyd Co)
9. Matte de Cuivre (Matt Cu)
10. Minerai de Cuivre (Min Cu)
11. Minerai de Cobalt (Min Co)
12. Scraps de Cuivre (Scraps Cu)

	QUANTITE DES PRODUITS EXPORTES EN TONNES												
	Alliage Rouge	Carb Co	Cath. Cu	Conc Mn	Conc Co	Conc Cu	Conc CuCo	Cu Blst	Hyd Co	Matt Cu	MINERAI DE CUIVRE	MINERAI DE COBALT	SCRAPS Cu
**BOSS MINING**													
**CCR**			1.162,65						385,00				
**CDM**													
**COMIKA**													
**COMMUS**			4.197,55			1.926,84		4.074,40		479,04			
**KATANGA METALS**													
**KCC**			22.071,13						3.426,14				262,09
**KIMIN**									230,05				
**LCS**								7.419,14					
**METALKOL**			5.407,76						3.030,18				
**MINING PROGRESS**					420,00								
**MKM**			1.527,66										
**MUMI**													
**SCM**			7.905,44			6.831,62							
**SCMK-Mn SA**													
**SM DEZIWA**			5.484,74						1.973,26				
**TAVIR**													
**TCC**			1.963,84						248,28				
**THOMAS MINING**													
TOTAL		*0*	*49.720,79*	*0,00*	*420,00*	*8.758,46*		*11.493,54*	*9.292,92*	*479,04*			*262,09*

		QUANTITE DES PRODUITS EXPORTES EN TONNES											
SOCIETE	Alliage Rouge	Carb Co	Cath. Cu	Conc Mn	Conc Co	Conc Cu	Conc CuCo	Cu Blst	Hyd Co	Matt Cu	MINERAI DE CUIVRE	MINERAI DE COBALT	SCRAPS Cu
TFM			*10.705,07*						*2.922,11*				

STATISTIQUES DE PRODUCTION, REDEVANCE MINIERE (MONTANTS ATTENDUS) PAR ENTITE BENEFICIAIRE POUR LE MOIS D'AVRIL 2020/ GUICHET UNIQUE KOLWEZI

SOCIETES	REDEVANCE MINIERE 100% (USD)	TRESOR 50%	PROVINCE 25%	TERRITOIRE 15%	FONDS MINIER 10%	QUANTITE DES PRODUITS EXPORTES EN TONNES												
						Alliage Rouge	Carb Co	Cath. Cu	Conc Mn	Conc Co	Conc Cu	Conc CuCo	Cu Blst	Hyd Co	Matt Cu	MINERAI DE CUIVRE	MINERAI DE COBALT	SCRAPS Cu
BOSS MINING	0.00	0.00	0.00	0.00	0.00			0.00	0.00	0.00	0.00	0.00	0.00	0.00	0.00	0.00	0.00	0.00
CCR	907,397.74	453,698.87	226,849.43	136,109.66	90,739.77			1,931.53	0.00	0.00	0.00		0.00	1,128.40	0.00			0.00
CDM	0.00	0.00	0.00	0.00	0.00			0.00	0.00	0.00	0.00		0.00	0.00	0.00			0.00
COMIKA	0.00	0.00	0.00	0.00	0.00			0.00	0.00	0.00	0.00		0.00	0.00	0.00			0.00
COMMUS	1,638,381.43	819,190.71	409,595.36	245,757.21	163,838.14			4,543.26	0.00	0.00	2,359.97		3,594.54	0.00	598.40			0.00
KATANGA METALS	0.00	0.00	0.00	0.00	0.00			0.00	0.00	0.00	0.00		0.00	0.00	0.00			0.00
KCC	9,328,116.27	4,664,058.13	2,332,029.07	1,399,217.44	932,811.63			20,747.55	0.00	0.00	0.00		0.00	6,621.46	0.00			192.59
KIMIN	81,892.45	40,946.23	20,473.11	12,283.87	8,189.25			0.00	0.00	0.00	0.00		0.00	167.48	0.00			0.00
LCS	1,754,120.01	877,060.01	438,530.00	263,118.00	175,412.00			0.00	0.00	0.00	0.00		10,293.43	0.00	0.00			0.00
METALKOL	3,569,864.54	1,784,932.27	892,466.14	535,479.68	356,986.45			6,815.82	0.00	0.00	0.00		0.00	3,166.66	0.00			0.00
MINING PROGRESS	144,900.00	72,450.00	36,225.00	21,735.00	14,490.00			0.00	0.00	900.00	0.00		0.00	0.00	0.00			0.00
MKM	946,131.70	473,065.85	236,532.92	141,919.75	94,613.17			2,003.56	0.00	0.00	0.00		0.00	790.80	0.00			0.00
MUMI	0.00	0.00	0.00	0.00	0.00			0.00	0.00	0.00	0.00		0.00	0.00	0.00			0.00
SCM	0.00	0.00	0.00	0.00	0.00			7,024.20	0.00	0.00	6,669.98		0.00	0.00	0.00			0.00
SCMK-Mn SA	0.00	0.00	0.00	0.00	0.00			1,999.82	0.00	0.00	0.00		0.00	0.00	0.00			0.00
SM DEZIWA	2,407,399.01	1,203,699.51	601,849.75	361,109.85	240,739.90			6,121.73	0.00	0.00	0.00		0.00	1,268.84	0.00			0.00
TAVIR	0.00	0.00	0.00	0.00	0.00			0.00	0.00	0.00	0.00		0.00	0.00	0.00			0.00
TCC	470,333.74	235,166.87	117,583.43	70,550.06	47,033.37			1,952.30	0.00	0.00	0.00		0.00	238.32	0.00			0.00
THOMAS MINING	0.00	0.00	0.00	0.00	0.00			0.00	0.00	0.00	0.00		0.00	0.00	0.00			0.00
TOTAL	*21,248,536.89*	*10,624,268.44*	*5,312,134.22*	*3,187,280.53*	*2,124,853.69*		*0*	*53,139.77*	*0.00*	*900.00*	*9,029.96*		*13,887.97*	*13,381.97*	*598.40*			*192.59*

Le total est de: **Vingt et Un Million Deux Cents Quarente-Huit Mille Cinq Cents Trente-Six et Quatre-Vingt-Six Centimes**

STATISTIQUES DE PRODUCTION, REDEVANCE MINIERE PAR QUOTITES EMISES POUR LE MOIS D'AVRIL 2020/GUICHET UNIQUE FUNGURUME

SOCIETE	REDEVANCE MINIERE 100% (USD)	TRESOR 50%	PROVINCE 25%	TERRITOIRE 15%	FONDS MINIER 10%	Alliage Rouge	QUANTITE DES PRODUITS EXPORTES EN TONNES											
							Carb Co	Cath. Cu	Conc Mn	Conc Co	Conc Cu	Conc CuCo	Cu Blst	Hyd Co	Matt Cu	MINERAI DE CUIVRE	MINERAI DE COBALT	SCRAPS Cu
TFM	*5,921,500.30*	*2,960,750.15*	*1,480,375.07*	*888,225.04*	*592,150.03*			*16,238.85*						*2,849.09*				

Le total est de: **Cinq Million Neuf Cents Vingt et Un Mille Cinq Cents et Trente Centimes**

TOTAL GENERAL PAR QUOTITE POUR LE MOIS D'AVRIL 2020 / GUICHET UNIQUE KOLWEZI ET FUNGURUME

REDEVANCE MINIERE 100% (USD)	TRESOR 50%	PROVINCE 25%	TERRITOIRE 15%	FONDS MINIER 10%
27,170,037.19	13,585,018.59	6,792,509.30	4,075,505.58	2,717,003.72

Le ToTal est de : **Vingt-Sept Million Cent Septente Mille Trente-Sept et Dix-Neuf Centimes**

LEGENDE :

1. Carbonate de Co (Carb Co);
2. Cathode de Cuivre (Cath Cu)
3. Concentré de Manganèse (Conc Mn)
4. Concentré de Cobalt (Conc Co)
5. Concentré de Cuivre (Conc Cu)
6. Concentré Cupro-Cobaltifère (Conc Cu Co)
7. Cuivre Blister (Cu-Blst)
8. Hydroxyde de Cobalt (Hyd Co)
9. Matte de Cuivre (Matt Cu)
10. Minerai de Cuivre (Min Cu)
11. Minerai de Cobalt (Min Co)
12. Scraps de Cuivre (Scraps Cu)

STATISTIQUES DE PRODUCTION, REDEVANCE MINIERE (MONTANTS ATTENDUS) PAR ENTITE BENEFICIAIRE POUR LE MOIS DE MAI 2020/ GUICHET UNIQUE KOLWEZI

SOCIETES	REDEVANCE MINIERE 100% (USD)	TRESOR 50%	PROVINCE 25%	TERRITOIRE 15%	FONDS MINIER 10%	QUANTITE DES PRODUITS EXPORTES EN TONNES												
						Alliage Rouge	Carb Co	Cath. Cu	Conc Mn	Conc Co	Conc Cu	Conc CuCo	Cu Blst	Hyd Co	Matt Cu	MINERAI DE CUIVRE	MINERAI DE COBALT	SCRAPS Cu
BOSS MINING	0.00	0.00	0.00	0.00	0.00													
CCR	526,786.22	263,393.11	131,696.55	79,017.93	52,678.62			1,718.49						465.50				
CDM	0.00	0.00	0.00	0.00	0.00													
COMIKA	0.00	0.00	0.00	0.00	0.00													
COMMUS	1,795,722.28	897,861.14	448,930.57	269,358.34	179,572.23			6,422.40			2,264.04		2,968.38		119.68			
KATANGA METALS	0.00	0.00	0.00	0.00	0.00													
KCC	11,787,844.54	5,893,922.27	2,946,961.13	1,768,176.68	1,178,784.45			21,996.53						9,067.11				378.65
KIMIN	20,153.88	10,076.94	5,038.47	3,023.08	2,015.39									42.04				
LCS/CBSHI	1,121,519.72	560,759.86	280,379.93	168,227.96	112,151.97								6,424.66					
LCS/KICC	471,190.59	235,595.29	117,797.65	70,678.59	47,119.06								2,803.65					
METALKOL	5,408,200.46	2,704,100.23	1,352,050.11	811,230.07	540,820.05			6,068.37						5,999.29				
MINING PROGRESS	62,309.53	31,154.77	15,577.38	9,346.43	6,230.95					517.50								
MKM	928,645.83	464,322.91	232,161.46	139,296.87	92,864.58			2,178.40						750.26				
MUMI	0.00	0.00	0.00	0.00	0.00													
SCM	0.00	0.00	0.00	0.00	0.00			4,277.28			7,009.03			226.33				
SCMK-Mn SA	0.00	0.00	0.00	0.00	0.00													
SM DEZIWA	2,625,404.83	1,312,702.41	656,351.21	393,810.72	262,540.48			9,966.42						1,195.97				
TAVIR	0.00	0.00	0.00	0.00	0.00													
TCC	309,647.62	154,823.81	77,411.91	46,447.14	30,964.76			1,341.01						151.67				
THOMAS MINING	0.00	0.00	0.00	0.00	0.00													
TOTAL	*25,057,425.48*	*12,528,712.74*	*6,264,356.37*	*3,758,613.82*	*2,505,742.55*		*0*	*53,968.92*	*0.00*	*517.50*	*9,273.07*		*12,196.69*	*17,898.17*	*119.68*			*378.65*

Le total est de: **Vingt-Cinq Million Cinquente-Sept Mille Quatre Cents Vingt-Cinq et Quarent-Huit Centimes**

STATISTIQUES DE PRODUCTION, REDEVANCE MINIERE PAR QUOTITES EMISES POUR LE MOIS DE MAI 2020/GUICHET UNIQUE FUNGURUME

SOCIETE	REDEVANCE MINIERE 100% (USD)	TRESOR 50%	PROVINCE 25%	TERRITOIRE 15%	FONDS MINIER 10%	Alliage Rouge	QUANTITE DES PRODUITS EXPORTES EN TONNES											
							Carb Co	Cath. Cu	Conc Mn	Conc Co	Conc Cu	Conc CuCo	Cu Blst	Hyd Co	Matt Cu	MINERAI DE CUIVRE	MINERAI DE COBALT	SCRAPS Cu
TFM	*5,269,898.32*	*2,634,949.16*	*1,317,474.58*	*790,484.75*	*526,989.83*			*14,741.27*						*2,768.25*				

Le total est de: **Cinq Million Deux Cents Soixante-Neuf Mille Huit Cents Nonante-Huit et Trente-Deux Centimes**

TOTAL GENERAL PAR QUOTITE POUR LE MOIS DE MAI 2020 / GUICHET UNIQUE KOLWEZI ET FUNGURUME

REDEVANCE MINIERE 100% (USD)	TRESOR 50%	PROVINCE 25%	TERRITOIRE 15%	FONDS MINIER 10%
30,327,323.81	15,163,661.90	7,581,830.95	4,549,098.57	3,032,732.38

Le ToTal est de : **Trente Million Trois Cents Vingt-Sept Mille Trois Cents Vingt-Trois et Quatre-Vingt-Un Centimes**

LEGENDE :

1. Carbonate de Co (Carb Co);
2. Cathode de Cuivre (Cath Cu)
3. Concentré de Manganèse (Conc Mn)
4. Concentré de Cobalt (Conc Co)
5. Concentré de Cuivre (Conc Cu)
6. Concentré Cupro-Cobaltifère (Conc Cu Co)
7. Cuivre Blister (Cu-Blst)
8. Hydroxyde de Cobalt (Hyd Co)
9. Matte de Cuivre (Matt Cu)
10. Minerai de Cuivre (Min Cu)
11. Minerai de Cobalt (Min Co)
12. Scraps de Cuivre (Scraps Cu)

STATISTIQUES DE PRODUCTION, REDEVANCE MINIERE (MONTANTS ATTENDUS) PAR ENTITE BENEFICIAIRE POUR LE MOIS DE JUIN 2020/ GUICHET UNIQUE KOLWEZI

SOCIETES	REDEVANCE MINIERE 100% (USD)	TRESOR 50%	PROVINCE 25%	TERRITOIRE 15%	FONDS MINIER 10%	QUANTITE DES PRODUITS EXPORTES EN TONNES												
						Alliage Rouge	Carb Co	Cath. Cu	Conc Mn	Conc Co	Conc Cu	Conc CuCo	Cu Blst	Hyd Co	Matt Cu	MINERAI DE CUIVRE	MINERAI DE COBALT	SCRAPS Cu
BOSS MINING																		
CCR	953,836.58	476,918.29	238,459.14	143,075.49	95,383.66			2,490.38						1,038.10				
CDM																		
COMIKA																		
COMMUS	2,407,677.48	1,203,838.74	601,919.37	361,151.62	240,767.75			4,820.86			2,326.82		3,301.46	1,014.49	607.96			
HANRUI METAL CONGO SARL	408,070.21	204,035.10	102,017.55	61,210.53	40,807.02			2,234.44										
KATANGA METALS																		
KCC	14,152,051.11	7,076,025.56	3,538,012.78	2,122,807.67	1,415,205.11			20,783.63						11,357.20				741.27
KIMIN	110,144.65	55,072.33	27,536.16	16,521.70	11,014.47									229.77				
LCS	254,955.32	127,477.66	63,738.83	38,243.30	25,495.53								1,494.95					
LCS/CBSHI	1,195,857.08	597,928.54	298,964.27	179,378.56	119,585.71								6,528.19					
LCS/KICC	295,990.01	147,995.01	73,997.50	44,398.50	29,599.00								1,686.65					
METALKOL	3,822,650.12	1,911,325.06	955,662.53	573,397.52	382,265.01			7,183.45						3,468.36				
MINING PROGRESS																		
MKM	745,364.98	372,682.49	186,341.24	111,804.75	74,536.50			2,052.53						511.07				
MUMI																		
SCM								8,006.79			9,136.78			310.14				
SCMK-Mn SA																		
SM DEZIWA	2,240,402.43	1,120,201.21	560,100.61	336,060.36	224,040.24			7,012.63						1,322.45				
TAVIR																		
TCC	369,229.85	184,614.92	92,307.46	55,384.48	36,922.98			1,387.84						239.90				
THOMAS MINING																		
TOTAL	*26,956,229.81*	*13,478,114.91*	*6,739,057.45*	*4,043,434.47*	*2,695,622.98*		*0*	*55,972.55*	*0.00*	*0.00*	*11,463.60*		*13,011.24*	*19,491.47*	*607.96*			*741.27*

Le total est de: **Vingt-Six Million Neuf Cents Cinquante-Six Mille Deux Cents Vingt-Neuf et Quatre-vingt-Un Centimes**

STATISTIQUES DE PRODUCTION, REDEVANCE MINIERE PAR QUOTITES EMISES POUR LE MOIS DE JUIN 2020/GUICHET UNIQUE FUNGURUME

SOCIETE	REDEVANCE MINIERE 100% (USD)	TRESOR 50%	PROVINCE 25%	TERRITOIRE 15%	FONDS MINIER 10%	Alliage Rouge	QUANTITE DES PRODUITS EXPORTES EN TONNES											
							Carb Co	Cath. Cu	Conc Mn	Conc Co	Conc Cu	Conc CuCo	Cu Blst	Hyd Co	Matt Cu	MINERAI DE CUIVRE	MINERAI DE COBALT	SCRAPS Cu
TFM	*9,915,606.80*	*4,957,803.40*	*2,478,901.70*	*1,487,341.02*	*991,560.68*			*32,482.73*						*4,573.40*				

Le total est de: **Neuf Million Neuf Cents Quinze Mille Six Cents Six et Quatre-Vingt Centimes**

TOTAL GENERAL PAR QUOTITE POUR LE MOIS DE JUIN 2020 / GUICHET UNIQUE KOLWEZI ET FUNGURUME

REDEVANCE MINIERE 100% (USD)	TRESOR 50%	PROVINCE 25%	TERRITOIRE 15%	FONDS MINIER 10%
36,871,836.61	18,435,918.30	9,217,959.15	5,530,775.49	3,687,183.66

Le ToTal est de : **Trente Six Million Huit Cents Trente et Un Mille Huit Cents Trente-Six et Soixante et Un Centimes**

LEGENDE :

1. Carbonate de Co (Carb Co);
2. Cathode de Cuivre (Cath Cu)
3. Concentré de Manganèse (Conc Mn)
4. Concentré de Cobalt (Conc Co)
5. Concentré de Cuivre (Conc Cu)
6. Concentré Cupro-Cobaltifère (Conc Cu Co)
7. Cuivre Blister (Cu-Blst)
8. Hydroxyde de Cobalt (Hyd Co)
9. Matte de Cuivre (Matt Cu)
10. Minerai de Cuivre (Min Cu)
11. Minerai de Cobalt (Min Co)
12. Scraps de Cuivre (Scraps Cu)

STATISTIQUES DE PRODUCTION, REDEVANCE MINIERE (MONTANTS ATTENDUS) PAR ENTITE BENEFICIAIRE POUR LE MOIS DE JUILLET 2020/ GUICHET UNIQUE KOLWEZI

						QUANTITE DES PRODUITS EXPORTES EN TONNES												
SOCIETES	REDEVANCE MINIERE 100% (USD)	TRESOR 50%	PROVINCE 25%	TERRITOIRE 15%	FONDS MINIER 10%	Alliage Rouge	Carb Co	Cath. Cu	Conc Mn	Conc Co	Conc Cu	Conc CuCo	Cu Blst	Hyd Co	Matt Cu	MINERAI DE CUIVRE	MINERAI DE COBALT	SCRAPS Cu
BOSS MINING	0.00	0.00	0.00	0.00	0.00													
CCR	1,136,183.79	568,091.90	284,045.95	170,427.57	113,618.38			2,845.92			0.00		0.00	1,120.00	0.00			0.00
CDM	0.00	0.00	0.00	0.00	0.00			0.00			0.00		0.00	0.00	0.00			0.00
COMIKA	0.00	0.00	0.00	0.00	0.00			0.00			0.00		0.00	0.00	0.00			0.00
COMMUS	2,465,535.54	1,232,767.77	616,383.88	369,830.33	246,553.55			5,879.14			1,263.79		3,055.58	680.15	1,070.15			0.00
IANRUI METAL CONGO SAR	366,397.79	183,198.90	91,599.45	54,959.67	36,639.78			1,834.34			0.00		0.00	0.00	0.00			0.00
KATANGA METALS	0.00	0.00	0.00	0.00	0.00			0.00			0.00		0.00	0.00	0.00			0.00
KCC	14,450,896.10	7,225,448.05	3,612,724.02	2,167,634.41	1,445,089.61			20,917.16			0.00		0.00	11,758.39	0.00			711.13
KIMIN	263,960.16	131,980.08	65,990.04	39,594.02	26,396.02			0.00			0.00		0.00	523.99	0.00			0.00
LCS	1,115,865.02	557,932.51	278,966.26	167,379.75	111,586.50			0.00			0.00		5,791.66	0.00	0.00			0.00
LCS/CBSHI	111,368.46	55,684.23	27,842.11	16,705.27	11,136.85			0.00			0.00		554.12	0.00	0.00			0.00
LCS/KICC	877,043.33	438,521.67	219,260.83	131,556.50	87,704.33			0.00			0.00		4,531.96	0.00	0.00			0.00
METALKOL	5,920,330.43	2,960,165.21	1,480,082.61	888,049.56	592,033.04			8,239.69			0.00		0.00	5,614.60	0.00			0.00
MINING PROGRESS	11,613.00	5,806.50	2,903.25	1,741.95	1,161.30			0.00			420.00		0.00	0.00	0.00			0.00
MKM	892,647.07	446,323.54	223,161.77	133,897.06	89,264.71			2,364.74			0.00		0.00	512.90	0.00			0.00
MUMI	0.00	0.00	0.00	0.00	0.00			0.00			0.00		0.00	0.00	0.00			0.00
SCM	0.00	0.00	0.00	0.00	0.00			7,523.54			15,221.19		0.00	215.32	0.00			0.00
SCMK-Mn SA		0.00	0.00	0.00	0.00						0.00		0.00		0.00			0.00
SM DEZIWA	2,535,774.64	1,267,887.32	633,943.66	380,366.20	253,577.46			5,722.39			0.00		0.00	1,489.97	0.00			0.00
TAVIR	0.00	0.00	0.00	0.00	0.00			0.00			0.00		0.00	0.00	0.00			0.00
TCC	345,472.22	172,736.11	86,368.05	51,820.83	34,547.22			1,323.38			0.00		0.00	157.81	0.00			0.00
THOMAS MINING	0.00	0.00	0.00	0.00	0.00			0.00			0.00		0.00	0.00	0.00			0.00
TOTAL	*30,493,087.56*	*15,246,543.78*	*7,623,271.89*	*4,573,963.13*	*3,049,308.76*		*0*	*56,650.29*	*0.00*	*0.00*	*16,904.97*		*13,933.31*	*22,073.11*	*1,070.15*			*711.13*

Le total est de: **Trente Million Quatre Cents Nonente-Trois Quatre-Vingt-Sept et Cinquente-Six Centimes**

STATISTIQUES DE PRODUCTION, REDEVANCE MINIERE PAR QUOTITES EMISES POUR LE MOIS DE JUILLET 2020/GUICHET UNIQUE FUNGURUME

							QUANTITE DES PRODUITS EXPORTES EN TONNES											
SOCIETE	REDEVANCE MINIERE 100% (USD)	TRESOR 50%	PROVINCE 25%	TERRITOIRE 15%	FONDS MINIER 10%	Alliage Rouge	Carb Co	Cath. Cu	Conc Mn	Conc Co	Conc Cu	Conc CuCo	Cu Blst	Hyd Co	Matt Cu	MINERAI DE CUIVRE	MINERAI DE COBALT	SCRAPS Cu
TFM	*6,254,469.64*	*3,127,234.82*	*1,563,617.41*	*938,170.45*	*625,446.96*			*18,948.86*						*2,642.15*				

Le total est de: **Six Million Deux Cents Cinquente-Quatre Mille Quatre Cents Sixante-Neuf et Soixante-Quatre Centimes**

TOTAL GENERAL PAR QUOTITE POUR LE MOIS DE JUILLET 2020 / GUICHET UNIQUE KOLWEZI ET FUNGURUME

REDEVANCE MINIERE 100% (USD)	TRESOR 50%	PROVINCE 25%	TERRITOIRE 15%	FONDS MINIER 10%
36,747,557.20	18,373,778.60	9,186,889.30	5,512,133.58	3,674,755.72

Le Total est de : **Trente Six Million Sept Cents Quarente-Sept Mille Cinq Cents Cinquante-Sept et Vingt Centimes**

LEGENDE :

1. Carbonate de Co (Carb Co);
2. Cathode de Cuivre (Cath Cu)
3. Concentré de Manganèse (Conc Mn)
4. Concentré de Cobalt (Conc Co)
5. Concentré de Cuivre (Conc Cu)
6. Concentré Cupro-Cobaltifère (Conc Cu Co)
7. Cuivre Blister (Cu-Blst)
8. Hydroxyde de Cobalt (Hyd Co)
9. Matte de Cuivre (Matt Cu)
10. Minerai de Cuivre (Min Cu)
11. Minerai de Cobalt (Min Co)
12. Scraps de Cuivre (Scraps Cu)

STATISTIQUES DE PRODUCTION, REDEVANCE MINIERE (MONTANTS ATTENDUS) PAR ENTITE BENEFICIAIRE POUR LE MOIS D'AOUT 2020/ GUICHET UNIQUE KOLWEZI

SOCIETES	REDEVANCE MINIERE 100% (USD)	TRESOR 50%	PROVINCE 25%	TERRITOIRE 15%	FONDS MINIER 10%	QUANTITE DES PRODUITS EXPORTES EN TONNES												
						Alliage Rouge	Carb Co	Cath. Cu	Conc Mn	Conc Co	Conc Cu	Conc CuCo	Cu Blst	Hyd Co	Matt Cu	MINERAI DE CUIVRE	MINERAI DE COBALT	SCRAPS Cu
BOSS MINING	0.00	0.00	0.00	0.00	0.00													
CCR	971,261.38	485,630.69	242,815.34	145,689.21	97,126.14			2,616.80						840.00				
CDM	0.00	0.00	0.00	0.00	0.00													
COMIKA	0.00	0.00	0.00	0.00	0.00													
COMMUS	1,957,085.20	978,542.60	489,271.30	293,562.78	195,708.52			3,838.87			998.89		2,287.49	733.44	302.52			
ANRUI METAL CONGO SAR	331,007.02	165,503.51	82,751.75	49,651.05	33,100.70			1,487.68										
KATANGA METALS	0.00	0.00	0.00	0.00	0.00													
KCC	10,647,904.97	5,323,952.48	2,661,976.24	1,597,185.75	1,064,790.50			18,025.85						7,908.80				536.01
KIMIN	432,698.37	216,349.19	108,174.59	64,904.76	43,269.84			468.00						700.79				
LCS	0.00	0.00	0.00	0.00	0.00													
LCS/CBSHI	0.00	0.00	0.00	0.00	0.00													
LCS/KICC	135,847.95	67,923.98	33,961.99	20,377.19	13,584.80								650.97					
METALKOL	4,263,981.22	2,131,990.61	1,065,995.31	639,597.18	426,398.12			6,883.76						3,913.14				
MINING PROGRESS	20,167.52	10,083.76	5,041.88	3,025.13	2,016.75					90.00	240.00							
MKM	671,229.44	335,614.72	167,807.36	100,684.42	67,122.94			1,272.66						555.77				
MUMI	0.00	0.00	0.00	0.00	0.00													
SCM	0.00	0.00	0.00	0.00	0.00			9,554.44			9,368.61			251.95				
SCMK-Mn SA	2,461.56	1,230.78	615.39	369.23	246.16				1,758.26									
SM DEZIWA	2,981,628.97	1,490,814.48	745,407.24	447,244.35	298,162.90			5,256.67						2,023.18				
TAVIR	0.00	0.00	0.00	0.00	0.00													
TCC	467,115.01	233,557.51	116,778.75	70,067.25	46,711.50			1,412.14						330.18				
THOMAS MINING	0.00	0.00	0.00	0.00	0.00													
TOTAL	*22,882,388.62*	*11,441,194.31*	*5,720,597.15*	*3,432,358.29*	*2,288,238.86*		*0*	*50,816.86*	*1,758.26*	*90.00*	*10,607.50*		*2,938.46*	*17,257.23*	*302.52*			*536.01*

Le total est de: **Vingt-Deux Million Huit Cents Quatre-Vingt-Deux Mille Trois Cents Quatre-Vingt-Huit et Soixante-Deux Centimes**

STATISTIQUES DE PRODUCTION, REDEVANCE MINIERE PAR QUOTITES EMISES POUR LE MOIS D'AOUT 2020/GUICHET UNIQUE FUNGURUME

SOCIETE	REDEVANCE MINIERE 100% (USD)	TRESOR 50%	PROVINCE 25%	TERRITOIRE 15%	FONDS MINIER 10%	Alliage Rouge	QUANTITE DES PRODUITS EXPORTES EN TONNES											
							Carb Co	Cath. Cu	Conc Mn	Conc Co	Conc Cu	Conc CuCo	Cu Blst	Hyd Co	Matt Cu	MINERAI DE CUIVRE	MINERAI DE COBALT	SCRAPS Cu
TFM	*7,308,723.02*	*3,654,361.51*	*1,827,180.75*	*1,096,308.45*	*730,872.30*			*17,622.68*						*3,631.01*				

Le total est de: **Sept Million Trois Cents Huit Mille Sept Cents Vingt-Trois et Deux Centimes**

TOTAL GENERAL PAR QUOTITE POUR LE MOIS D'AOUT 2020 / GUICHET UNIQUE KOLWEZI ET FUNGURUME

REDEVANCE MINIERE 100% (USD)	TRESOR 50%	PROVINCE 25%	TERRITOIRE 15%	FONDS MINIER 10%
30,191,111.63	15,095,555.82	7,547,777.91	4,528,666.74	3,019,111.16

Le ToTal est de : **Trente Million Cent Nonante et Un Mille Cent onze et Soixante-Trois Centimes**

LEGENDE :

1. Carbonate de Co (Carb Co);
2. Cathode de Cuivre (Cath Cu)
3. Concentré de Manganèse (Conc Mn)
4. Concentré de Cobalt (Conc Co)
5. Concentré de Cuivre (Conc Cu)
6. Concentré Cupro-Cobaltifère (Conc Cu Co)
7. Cuivre Blister (Cu-Blst)
8. Hydroxyde de Cobalt (Hyd Co)
9. Matte de Cuivre (Matt Cu)
10. Minerai de Cuivre (Min Cu)
11. Minerai de Cobalt (Min Co)
12. Scraps de Cuivre (Scraps Cu)

STATISTIQUES DE PRODUCTION, REDEVANCE MINIERE (MONTANTS ATTENDUS) PAR ENTITE BENEFICIAIRE POUR LE MOIS DE SEPTEMBRE 2020/ GUICHET UNIQUE KOLWEZI

						QUANTITE DES PRODUITS EXPORTES EN TONNES												
SOCIETES	REDEVANCE MINIERE 100% (USD)	TRESOR 50%	PROVINCE 25%	TERRITOIRE 15%	FONDS MINIER 10%	Alliage Rouge	Carb Co	Cath. Cu	Conc Mn	Conc Co	Conc Cu	Conc CuCo	Cu Blst	Hyd Co	Matt Cu	MINERAI DE CUIVRE	MINERAI DE COBALT	SCRAPS Cu
ANVIL MINING	150,043.11	75,021.56	37,510.78	22,506.47	15,004.31								704.59					
CCR	919,220.01	459,610.01	229,805.00	137,883.00	91,922.00			2,067.28						840.00				
CDM		0.00	0.00	0.00	0.00													
COMIKA		0.00	0.00	0.00	0.00													
COMMUS	1,989,407.93	994,703.96	497,351.98	298,411.19	198,940.79			4,428.79					1,612.07	735.49	331.01			
ANRUI METAL CONGO SAF	465,505.41	232,752.71	116,376.35	69,825.81	46,550.54			2,047.22										
KATANGA METALS		0.00	0.00	0.00	0.00													
KCC	14,677,398.21	7,338,699.11	3,669,349.55	2,201,609.73	1,467,739.82			23,617.19						8,854.66				2,173.64
KIMIN	279,135.25	139,567.63	69,783.81	41,870.29	27,913.53			1,042.00						78.92				
LCS	1,199,394.41	599,697.20	299,848.60	179,909.16	119,939.44								5,571.40					
KINGSAIL	20,099.20	10,049.60	5,024.80	3,014.88	2,009.92								88.39					
KICC	1,495,446.14	747,723.07	373,861.53	224,316.92	149,544.61								6,880.61					
METALKOL	3,545,965.04	1,772,982.52	886,491.26	531,894.76	354,596.50			6,073.85						2,685.36				
MINING PROGRESS		0.00	0.00	0.00	0.00													
MKM	879,390.51	439,695.25	219,847.63	131,908.58	87,939.05			2,007.09						524.71				
MUMI		0.00	0.00	0.00	0.00													
SICOMINES		0.00	0.00	0.00	0.00			5,672.98						200.61				
SCMK-Mn SA	503.65	251.83	125.91	75.55	50.37				359.75									
SM DEZIWA	4,147,475.65	2,073,737.83	1,036,868.91	622,121.35	414,747.57			5,972.49						2,825.73				
TAVIR		0.00	0.00	0.00	0.00													
TCC	568,615.86	284,307.93	142,153.97	85,292.38	56,861.59			1,856.97						273.74				
THOMAS MINING		0.00	0.00	0.00	0.00													
TOTAL	*30,337,600.38*	*15,168,800.19*	*7,584,400.10*	*4,550,640.06*	*3,033,760.04*		*0*	*54,785.85*	*359.75*	*0.00*	*0.00*		*14,857.06*	*17,019.23*	*331.01*			*2,173.64*

Le total est de: **Trente Million Trois Cents Trente-Sept Mille Six Cents et Trente-Huit Centimes**

STATISTIQUES DE PRODUCTION, REDEVANCE MINIERE PAR QUOTITES EMISES POUR LE MOIS DE SEPTEMBRE 2020/GUICHET UNIQUE FUNGURUME

							QUANTITE DES PRODUITS EXPORTES EN TONNES											
SOCIETE	REDEVANCE MINIERE 100% (USD)	TRESOR 50%	PROVINCE 25%	TERRITOIRE 15%	FONDS MINIER 10%	Alliage Rouge	Carb Co	Cath. Cu	Conc Mn	Conc Co	Conc Cu	Conc CuCo	Cu Blst	Hyd Co	Matt Cu	MINERAI DE CUIVRE	MINERAI DE COBALT	SCRAPS Cu
TFM	*7,564,523.61*	*3,782,261.80*	*1,891,130.90*	*1,134,678.54*	*756,452.36*			*17,159.54*						*3,938.07*				

Le total est de: **Sept Million Cinq Cents Soixante-Quatre Mille Cinq Cents Vingt-Trois et Soixante et Un Centimes**

TOTAL GENERAL PAR QUOTITE POUR LE MOIS DE SEPTEMBRE 2020 / GUICHET UNIQUE KOLWEZI ET FUNGURUME

REDEVANCE MINIERE 100% (USD)	TRESOR 50%	PROVINCE 25%	TERRITOIRE 15%	FONDS MINIER 10%
37,902,123.99	18,951,061.99	9,475,531.00	5,685,318.60	3,790,212.40

Le ToTal est de : **Trente-Sept Million Neuf Cents Deux Mille Cent Vingt-Trois et Nonente-Neuf Centimes**

LEGENDE :

1. Carbonate de Co (Carb Co);
2. Cathode de Cuivre (Cath Cu)
3. Concentré de Manganèse (Conc Mn)
4. Concentré de Cobalt (Conc Co)
5. Concentré de Cuivre (Conc Cu)
6. Concentré Cupro-Cobaltifère (Conc Cu Co)
7. Cuivre Blister (Cu-Blst)
8. Hydroxyde de Cobalt (Hyd Co)
9. Matte de Cuivre (Matt Cu)
10. Minerai de Cuivre (Min Cu)
11. Minerai de Cobalt (Min Co)
12. Scraps de Cuivre (Scraps Cu)
